# Patterns of sequence conservation in presynaptic neural genes

**DOI:** 10.1186/gb-2006-7-11-r105

**Published:** 2006-11-10

**Authors:** Dexter Hadley, Tara Murphy, Otto Valladares, Sridhar Hannenhalli, Lyle Ungar, Junhyong Kim, Maja Bućan

**Affiliations:** 1 Penn Center for Bioinformatics, 423 Guardian Drive, University of Pennsylvania, Philadelphia, Pennsylvania 19104, USA; 2 Genomics and Computational Biology Graduate Group, 423 Guardian Drive, University of Pennsylvania, Philadelphia, Pennsylvania 19104, USA; 3 Department of Genetics in the School of Medicine, University of Pennsylvania, 415 Curie Boulevard Philadelphia, Pennsylvania 19104, USA; 4 UCLA Neuroscience Graduate Office, 695 Young Drive South, Los Angeles, California 90095, USA; 5 Department of Computer & Information Sciences in School of Engineering and Applied Sciences, 3330 Walnut Street, University of Pennsylvania, Philadelphia, Pennsylvania 19104, USA; 6 Department of Biology in the School of Arts and Sciences, 433 S University Avenue, University of Pennsylvania, Philadelphia, Pennsylvania 19104, USA

## Abstract

**Background:**

The neuronal synapse is a fundamental functional unit in the central nervous system of animals. Because synaptic function is evolutionarily conserved, we reasoned that functional sequences of genes and related genomic elements known to play important roles in neurotransmitter release would also be conserved.

**Results:**

Evolutionary rate analysis revealed that presynaptic proteins evolve slowly, although some members of large gene families exhibit accelerated evolutionary rates relative to other family members. Comparative sequence analysis of 46 megabases spanning 150 presynaptic genes identified more than 26,000 elements that are highly conserved in eight vertebrate species, as well as a small subset of sequences (6%) that are shared among unrelated presynaptic genes. Analysis of large gene families revealed that upstream and intronic regions of closely related family members are extremely divergent. We also identified 504 exceptionally long conserved elements (≥360 base pairs, ≥80% pair-wise identity between human and other mammals) in intergenic and intronic regions of presynaptic genes. Many of these elements form a highly stable stem-loop RNA structure and consequently are candidates for novel regulatory elements, whereas some conserved noncoding elements are shown to correlate with specific gene expression profiles. The SynapseDB online database integrates these findings and other functional genomic resources for synaptic genes.

**Conclusion:**

Highly conserved elements in nonprotein coding regions of 150 presynaptic genes represent sequences that may be involved in the transcriptional or post-transcriptional regulation of these genes. Furthermore, comparative sequence analysis will facilitate selection of genes and noncoding sequences for future functional studies and analysis of variation studies in neurodevelopmental and psychiatric disorders.

## Abstract 2

Comparative sequence analysis and annotation of genomic regions surrounding 150 presynaptic genes identified over 26,000 elements highly conserved in eight vertebrate species; these results are made available in the SynapseDB database.

## Background

The neuronal synapse is composed of presynaptic and postsynaptic components, and communication across these components is mediated by the release of neurotransmitters from synaptic vesicles. This process is initiated in the presynaptic terminal when an action potential opens voltage-gated Ca^2+ ^channels and a Ca^2+ ^influx triggers intracellular membrane fusion between the synaptic vesicles and plasma membrane. Before fusion, synaptic vesicles are targeted to dock at the active zone of the presynaptic membrane in a pathway that is mediated by the formation and regulation of SNARE complexes. These multiprotein complexes are composed of proteins that are bound constitutively or transiently to the synaptic vesicles or plasma membrane. Among them are synaptotagmins, the vesicular Ca^2+ ^sensors that trigger the Ca^2+ ^release. RAB proteins, at least *RAB3*, *RAB5 *and *RAB11 *family members, form a large set of GTP-binding proteins that regulate vesicle transport, docking, and late steps in exocytosis. RAB effectors include rabphilin, RIMs, RAB GDP dissociation inhibitor (*RABGDI*), RAB GTPase activating protein (*RAB3GAP*), RAB GDP/GTP exchange protein (*RAB3GEP*) and guanine nucleotide exchange factors (GEFs), among others. There is a substantial volume of data on the biochemical and physiological roles for a large number of presynaptic genes, although their role with respect to behavior and human disease is largely unknown [1].

Studies of neuronal synapses provide an excellent framework for the analysis of regulatory elements involved in all major levels of gene regulation. Although many genes involved in synaptic function are expressed during the early stages of development, an increase in their expression during development and in early postnatal stages, as well as the intricate complexity of their temporal and spatial patterns of expression in the adult brain, implicate the role of transcriptional control in their regulation [2,3]. Alternative transcription start sites and splicing of pre-mRNA represents another versatile mechanism for cell-type specificity in the brain [4,5]. For example, the trans-synaptic interaction of neurexins on the presynaptic terminal with neuroligins on the postsynaptic terminal is thought to coordinate synaptic connectivity, and this interaction is regulated by alternative splicing of both neuroligin and neurexin genes [4-6].

To facilitate identification of regulatory elements that are involved in the transcriptional and post-transcriptional control of gene expression in the neuronal synapse, we initiated a large-scale comparative analysis of genomic sequence for genes implicated in presynaptic function. Comparative sequence analysis of rodent (mouse and rat) and human genomes estimates that approximately 5% of small segments of sequence (50-100 base pairs [bp]) are under negative or purifying selection [7]; that is, nucleotide changes are occurring slower that would be expected given the underlying neutral mutation rate. Although a portion of this sequence can be accounted for by protein-coding regions of the genome (1.5%) and untranslated regions of protein-coding genes (1%), the function of the remaining 2.5% of conserved sequence remains elusive. Experimental studies support claims that a portion of these conserved noncoding sequences in intergenic and intronic regions represent *cis*-regulatory elements [8,9]. Furthermore, recent evidence points to an important role that short nonprotein coding RNAs, micro RNAs (miRNA) and small interfering RNAs (siRNAs), play in gene regulation [10,11].

Despite efforts to elucidate the function of noncoding conserved elements at the level of the entire genome, the identification, functional annotation, and systematic classification of the elements *vis à vis *a specific pathway remains incomplete. The synapse, involving both the presynaptic and postsynaptic cellular compartments, forms a distinct functional unit within a neuronal cell, and the associated molecular processes are parts of distinct localized pathways [12,13]. Our goal is to use the neuronal synapse as a model for comparative and integrative sequence analysis in order to generate systematically an inventory of putative functional genomic elements in a subcellular compartment by dissecting patterns of molecular evolution for subsequences surrounding presynaptic genes both within and between species.

In this study we conducted analyses of the genomic neighborhoods surrounding presynaptic genes from whole-genome multiple alignments of human with seven other vertebrate genomes. We find that genes that are involved in presynaptic transmission exhibit stronger evidence of purifying selection than do vertebrate genes as a whole. Interestingly, however, in large gene families at least one member often shows unusually relaxed purifying selection with a higher accumulation of amino acid changes compared with the other members of the family. Overall, there are many segments of noncoding regions that are well conserved across orthologous genomic segments but show divergence within paralogous regions of the same genome, suggesting an ancestral pattern of *cis*-regulatory functional divergence and stabilization within the vertebrate lineages. Furthermore, our studies provide a catalog of exceptionally long (≥360 bp) highly conserved sequences (>80% pair-wise identity from humans to mammals and >70% pair-wise identity from humans to nonmammals). In some cases, identified elements map in the vicinity of exon-intron boundaries of experimentally validated functional and developmentally regulated splice forms. Therefore, by classifying a large number of these discrete elements with respect to their relative genic position (intergenic, intronic, 5'- and 3'-untranslated region [UTR], and intron-exon boundary) and their potential to encode RNA or form stable RNA structure, we provide a foundation for more informed functional studies.

## Results

### Presynaptic gene index

Our analysis focuses on a set of 150 proteins mainly in the presynaptic nerve terminal known to participate in synaptogenesis or neurotransmitter release (Table 1). Using literature searches we first compiled a list of human genes implicated in synaptic vesicle exocytosis based on biochemical and functional studies [1,14]. We then established SynapseDB [15], which is a database of synaptic process genes/proteins in the human genome and their orthologs in multiple species such as the mouse (*Mus musculus*), rat (*Rattus norvegicus*), dog (*Canis familiaris*), chicken (*Gallus gallus*), zebrafish (*Danio renio*), puffer fish (*Takifugu rubripes*), fruitfly (*Drosophila melanogaster*), and worm (*Caenorhabditis elegans*). For the majority of presynaptic genes we established orthology by a straightforward mapping of the pair-wise reciprocal best BLAST (basic local alignment search tool) hits [16]. In addition to the nucleotide and protein sequence alignment, the establishment of paralogy/orthology relationships for large gene families required comparison of syntenic gene order to unambiguously identify orthologs and species-specific paralogs derived from gene duplication. In cases in which presynaptic genes belong to large gene families, we generally included all known paralogs regardless of their function in the presynaptic neuron. We also considered in our analysis neuroligins, a family of trans-synaptic proteins on the postsynaptic terminal known to interact with neurexins on the presynaptic terminal.

For 144 genes in the dataset, expression patterns from microarray analysis of 79 human nonredundant tissues and cell lines were available, courtesy of the Genomics Institute of the Novartis Research Foundation [17,18]. Furthermore, *in situ *hybridization patterns in adult brain are available for 91 selected genes from the Allen Brain Atlas [19]. To examine patterns of conservation in the genomic neighborhood of 150 presynaptic genes, we defined genomic regions of interest (gROIs) for each gene. The gROIs include protein-coding regions with 5'-UTR and 3'-UTR, intronic sequences, and the upstream and downstream regions as defined by the two neighboring genes on the chromosome regardless of strand. The gROIs for the 150 presynaptic genes encompass a total of 46 megabases (Mb) dispersed throughout the genome (Additional data file 1). Four pairs of genes had overlapping gROIs (*EPIM*-*RIMBP2*, *STX1B2*-*STX4A*, *GZMB*-*STXBP6*, and *VAMP5*-*VAMP8*) because of spatial proximity. Presynaptic genes had an average (mean ± standard deviation) size of 145.1 ± 240.0 kilobases (kb), with a median size of 51.2 kb and a range of 850 bp (*CALML5*) to 1.6 Mb (*NRXN3*). The gROIs are on average 311.5 ± 531.7 kb, with a median size of 126.3 kb, and gROI sizes range from 2.3 kb (*CAMK2N2*) to 4.5 Mb (*NRXN1*). The average size of the upstream regions is 115.9 ± 282.6 kb, with a median size of 29.9 kb and a maximum of 2.6 Mb (*NRXN1*). The average downstream size is 72.1 ± 152.9 kb with a median of 15.0 kb and a maximum size of 1.0 Mb (*NLGN4Y*). Nine presynaptic genes in our dataset were separated by more than 500 kb (within 'gene deserts') from any neighboring genes (*CAMK1G*, *NBEA*, *NCAM1*, *NLGN1*, *NLGN4Y*, *NRXN1*, *SYT1*, *SYT10*, and *UNC13C*).

### Molecular evolution of presynaptic genes and gene families

Before initiating systematic comparative analysis, we conducted a focused study of the molecular evolution of 150 presynaptic genes, including several large gene families. There are 10 large gene families containing five or more members such as calcium/calmodulin-dependent protein kinase (CAMK), exocyst complex (EXOC), neuroligins (NLGN), secretory carrier membrane protein (SCAMP), synaptotagmins (SYT), syntaxins (STX), syntaxin binding protein (STXBP), RAB GTPases (RAB), and vesicle associated membrane proteins (VAMP), as well as 15 smaller gene families containing between two and five paralogs. The RAB family is the largest family and evolutionary analysis for over 60 members has previously been reported [20,21]. We selected four members from the RAB3 family and three from the RAB5 family because members of these subfamilies are thought to be particularly important in the molecular dynamics of synaptic transmission [22,23]. In other families we consider all known paralogs. Two families, namely the SYTs and STXs, are considerably large, having 15 and 17 paralogs, respectively. All of the members of each family have orthologs in the human, the mouse, and the rat with one exception. Based on BLAST analysis and syntenic mapping, STX10 appears to have no mouse or rat ortholog.

**Figure 1:**
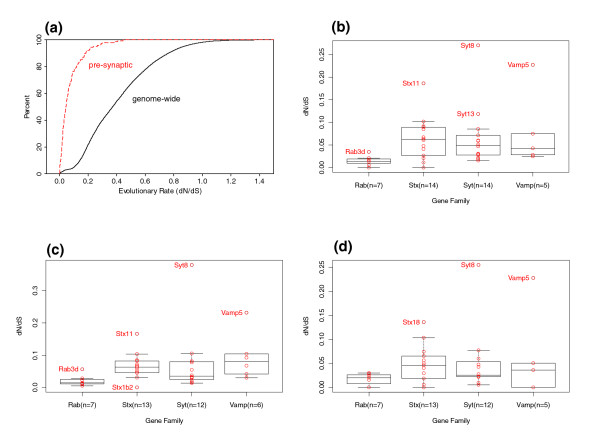
Evolutionary analysis of proteins involved in synaptic transmission. **(a) **The empirical cumulative distribution of protein evolutionary rate, measured by d_N_/d_S_, was calculated for human-mouse orthologs. Data for 139 human-mouse orthologs of mainly presynaptic genes is shown in red whereas a comprehensive survey of more than 15,000 homologous pairs of human-mouse orthologs is shown in black. **(b) **The distribution of d_N_/d_S _calculated for human-mouse orthologs was grouped by gene family. All family members are shown in red and extreme members outside whiskers are labeled. Black boxes showing the 25% quantile, the median, and 75% quantile are superimposed, and whiskers extend to the most extreme data point that is no more than the interquartile range in both directions from the median in the box. **(c) **The distribution of d_N_/d_S _calculated for human-rat orthologs was grouped by gene family. **(d) **The distribution of d_N_/d_S _calculated for mouse-rat orthologs grouped by gene family. d_N_, nonsynonymous rate of change; d_S_, synonymous rate of change.

To assess the rate of molecular evolution we computed the ratio of the nonsynonymous (amino acid replacing) rate of change to the synonymous (silent) rate of change (d_N_/d_S_) for pair-wise comparison of orthologs between human, mouse, and rat. d_N _is the relative rate of nonsynonymous mutations, and d_S _is the relative rate of synonymous mutations, and their ratio d_N_/d_S _indicates the direction of selection pressure acting on the proteins. Therefore, d_N_/d_S _< 1 suggests purifying selection, d_N_/d_S _= 1 suggests neutral selection, and d_N_/d_S _> 1 suggests positive selection. We were able to calculate d_N_/d_S _for 139 presynaptic genes and their average d_N_/d_S _is fivefold lower than that of a comprehensive genomic survey of 15,398 homologous pairs of human-mouse transcripts (0.072 versus 0.413; Figure 1a), which suggests purifying selection has broadly acted on genes known to be involved in synaptic transmission, as previously reported [24]. For presynaptic genes relative to the genomic survey, the average d_N _was almost 20-fold lower (0.043 ± 0.005 versus 0.848 ± 0.004; *P *< 0.001), and interestingly the average d_S _was almost fourfold lower (0.558 ± 0.016 versus 2.171 ± 0.008; *P *< 0.001).

When we focused only on largest four gene families (RABs, STXs, SYTs, and VAMPs), at least one family member exhibited elevated d_N_/d_S _compared with the remaining members; the most extreme members were *RAB3D*, *STX11*, *SYT8*, and *VAMP5 *in both human-mouse and human-rat comparisons (Figure 1b,c). Thus, in each large gene family one member is showing elevated levels of amino acid substitution relative to the overall substitution rate of the family. To investigate the human specificity of such outliers, we compared mouse-rat divergence of the same genes (Figure 1d). Interestingly, *SYT8 *and *VAMP5 *appeared as outliers in the mouse-rat comparisons, suggesting that these genes are under less pressure for purifying selection relative to other family members in all three species considered. In the syntaxins, *STX11 *is the most extreme outlier in both human-rodent comparisons, whereas *STX18 *is the most extreme outlier in mouse-rat comparisons. Similarly in the RAB family, *RAB3D *exhibits greater amino acid evolution in human-rodent comparisons but not in mouse-rat comparisons. Thus, this initial sequence analysis of large gene families suggests both *STX11 *and *RAB3D *have undergone human-specific patterns of faster amino acid fixations. The d_N_/d_S _ratio is still less than 1.0; therefore, this may be due to more relaxed functional constraints on these genes and less purifying selection. However, it is also possible that small domains might be undergoing positive selection whose rate is obscured by stabilizing selection on the remaining parts of the molecule. For instance, a current comparative analysis of human and great ape sequences found evidence for positive selection on sequences encoding a protein domain of unknown function (DUF1220), and these unknown domains are highly expressed in brain regions associated with higher cognitive function, and in brain they show neuron-specific expression preferentially in cell bodies and dendrites [25].

Phylogenetic analysis of gene families was performed for synaptotagmins (SYTs), syntaxins (STXs), RABs, and vesicle-associated membrane proteins (VAMPs) using the protein-coding sequence of all known human paralogs and their mouse orthologs. We included homologs from *Drosophila *outgroups whenever available. The VAMPs comprised the smallest family, with six members (*VAMP1*, *VAMP2*, *VAMP3*, *VAMP4*, *VAMP5*, and *VAMP8*), and all mammalian orthologous copies of this family form monophyletic groups (Additional data file 2), suggesting that the gene family diversified before the current eutherian species diversification. Rooting the tree from the two *Drosophila *homologs, *dVAMP1 *and *dVAMP2*, separates two clades each with three members: *VAMP1 *+ *VAMP2 *+ *VAMP3 *and *VAMP4 *+ *VAMP5 *+ *VAMP8*. (We note that the *Drosophila *nomenclature does not reflect homology relationships.) The split into these two clades was robust across different phylogeny estimation techniques, with a single variation in which the two different *Drosophila *homologs either formed a monophyletic root or a paraphyletic group rooting the respective VAMP subfamilies.

The family of RAB GTPases contains more than 60 members, from which we selected seven closely related members in the RAB3 and RAB5 subfamilies for analysis (*RAB3A*, *RAB3B*, *RAB3C*, *RAB3D*, *RAB5A*, *RAB5B*, and *RAB5C*). The resulting tree placed all orthologous copies in monophyletic clades, indicating the RABs also diversified before the human-rodent split (Additional data file 3). All orthologs separate into the two subfamilies similar to the VAMP diversification with *Drosophila RAB3 *and *RAB5 *homologs, respectively *dRAB3 *and *dRAB5*, forming the root of each subfamily. This pattern of two invertebrate homologs forming the roots of two subfamilies is identical to the pattern seen in the neighbor-joining estimate of the VAMP phylogeny, suggesting an ancestral two-gene family that respectively diversified in the vertebrates. In the RAB3 subfamily, mammalian *RAB3D *was consistently placed adjacent to *dRAB3 *with high significance, a finding that was robust to different tree estimation techniques, which suggests that *RAB3D *diversified from the ancestral vertebrate gene before *RAB3A*, *RAB3B*, and *RAB3C*. Interestingly, *RAB3D *also exhibits an unusual pattern of greater amino acid changes with high d_N_/d_S _ratios in both human-mouse and human-rat comparisons, but not in the mouse-rat comparison, suggesting a human-specific pattern.

In the STX family, all 14 protein-coding members analyzed (*STX1a*, *STX1b*, *STX2*, *STX3*, *STX4a*, *STX5a*, *STX6*, *STX7*, *STX8*, *STX10*, *STX11*, *STX12*, *STX16*, and *STX18*) formed orthologous monophyletic groups with some notable features (Additional data file 4). First, *STX10*, which is human specific, is placed basal to the mammalian *STX6 *clade (100% bootstrap support), suggesting that *STX10 *diversified before *STX6 *in the most recent common ancestor of human and mouse, and then the copy was lost in the rodent lineage. Interestingly, all *Drosophila *homologs are placed basal to their mammalian counterparts either as sister taxa (*STX1A*, *STX5*, *STX16*, and *STX18*) or at the base of an inclusive clade (*STX7*). Thus, STXs appear to have diversified early in the metazoan evolution with multiple ancestral copies, which subsequently diversified further in the vertebrate or mammalian lineage. The absence of *Drosophila *homologs for well supported clades such as *hSTX10 *+ *hSTX6 *+ *mSTX6 *suggests loss of ancestral copies in flies. The structure of the phylogenetic tree suggests at least two additional ancestral copies may have been lost in the invertebrate lineage.

In the SYT family, we analyzed 17 members with copies in human and mouse along with four *Drosophila *homologs (Additional data file 5). Again, all mammalian orthologous genes formed monophyletic groups, suggesting that this family also diverged at the base of the mammalian lineage. The only four *Drosophila *homologs identified were placed basal to the mammalian clades of *SYT7*, *STY4 *+ *STY11*, *STY1*, and *STY14 *+ *SYT16*, and given the size of the SYT family we may be missing other putative ancestral copies for the other lineages. Being conservative and collapsing branches supported by bootstrap values less than 65%, we predict that we are missing the invertebrate homolog for the *STY9 *+ *STY10 *+ *STY6 *+ *STY3 *clade and the remaining paraphyletic group of *STY8 *+ *STY13 *+ *STY15 *+ *STY17 *+ *STY12*. Thus, again for the SYTs, there may have been six ancestral copies in the metazoan lineage.

**Figure 2:**
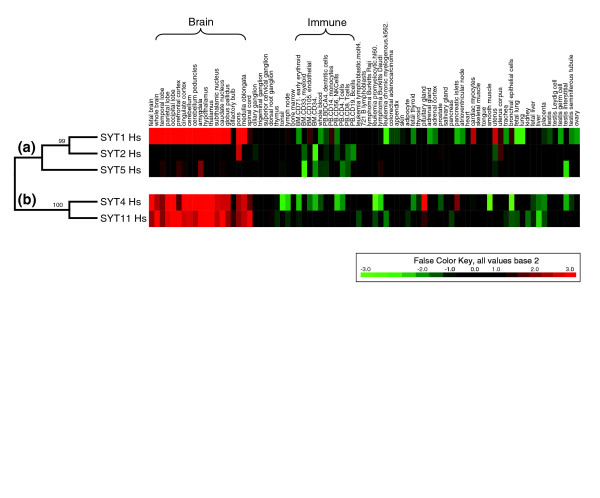
*SYT *protein trees with superimposed expression profiles. **(a) **The *SYT1*-*SYT2*-*SYT5 *clade of the SYT protein tree is shown for human and mouse orthologs with the expression profile for human genes superimposed. **(b) **Two closely related paralogs of the SYT family (*SYT4 *and *SYT11*) are shown with superimposed expression profiles.

Finally, to compare gene expression across tissues in a gene family context, we superimposed expression profiles obtained by microarray analysis of 79 human nonredundant tissues and cell lines [18] on the phylogenetic trees described above. Among paralogs closely related by coding sequence, there is considerable variation in patterns of gene expression. We found the best correlation between protein sequence similarity and expression similarity in the RAB subfamilies (Additional data file 6). Phylogenetic analysis of synaptotagmins and comparison with expression profiles illustrate two possible scenarios (Figure 2). On one hand, closely related paralogs *SYT4*-*SYT11 *within the same clade share a remarkably similar brain-enriched pattern of expression. On the other hand, the *SYT1*-*SYT2 *pair within the same clade exhibit different expression profiles, with *SYT1 *showing strong enrichment across multiple brain tissues whereas *SYT2 *shows strong enrichment in only 1 out of 18 brain tissues. Although *SYT5 *is placed immediately basal to the *SYT1*-*SYT2 *clade, it shares a similar broad brain-enrichment expression pattern as *SYT1*. Close inspection of alignment of the *SYT1*, *SYT2*, and *SYT5 *gROIs did not reveal nucleotide sequence homology outside of exons (see Duplicated MCEs among gROIs, below). Thus, the more narrow tissue specificity of *SYT2 *seems to be an evolutionarily derived condition that is likely due to rapid functional diversification of noncoding sequence after the *SYT1*-*SYT2 *evolutionary split.

### Comparative analysis of presynaptic genes

**Figure 3:**
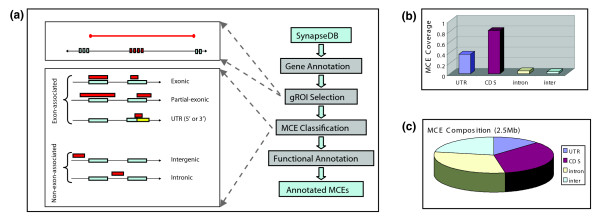
Comparative analysis of presynaptic genes. **(a) **Gene names from SynapseDB were used to query RefSeq and ENSEMBL transcript annotations, which were then clustered into gene models defined as groups of overlapping transcripts in the same orientation. The region around the synaptic gene model was extended up to the next annotated upstream and downstream gene models to define gROIs. MCEs were selected and characterized based on their relative genic position into exon-associated and non-exon-associated elements. Exon-associated elements were further subdivided into those that are completely exonic, those that are partially exonic and span exon-intron boundries, and those associated with UTRs; whereas non-exon-associated elements were divided into those that are intergenic and those that are intronic. **(b) **Individual bases were annotated as CDS, UTR sequence (UTR), intronic (intron), or intergenic (inter) based on gene model annotations. The coverage of MCEs (the proportion of most conserved bases in a gROI) across different annotations is shown. **(c) **The composition of MCEs (the proportion of MCEs with a given annotation) across CDS, UTR, intronic, and intergenic annotations is shown. CDS, coding sequence; gROI, genomic region of interest; MCR, most conserved element; UTR, untranslated region.

To automate comparative sequence analysis of gROIs we established a computational pipeline (Figure 3a) to select and analyze the most conserved elements (MCEs) from genome-wide alignments of human with seven other vertebrate genomes (the chimpanzee *Pan troglodytes*, the dog *Canis familiaris*, the mouse *Mus musculus*, the rat *Rattus norvegicus*, the chicken *Gallus gallus*, the zebra fish *Danio renio*, and the puffer fish *Fugu rubripes*) provided by the UCSC Genome Browser [26,27]. MCEs were identified using phastCons, a phylogenetic hidden Markov model that considers nucleotide substitutions in a phylogenetic context. This algorithm is suited to problems in which aligned sequences are to be parsed into segments of different classes, such as 'conserved' and 'nonconserved' [28]. By submitting 150 presynaptic gROIs (covering more than 46 Mb) to the pipeline, we identified about 26,000 (26,197) MCEs for analysis, spanning approximately 5% (2.5 Mb) of all gROI regions, corresponding to the portion of the human genome that is under selective pressure [7,29]. MCEs were on average (mean ± standard deviation) 86 ± 90 bp, with median size 54 bp (see Additional data file 7 for a distribution of MCE lengths).

We classified each nucleotide in the gROI input sequence as 'coding', 'intronic', 'intergenic', or 'UTR', based on a combination of RefSeq and Ensembl annotation. For each gROI considered, we calculated the proportion of each class covered by MCEs (see Additional data file 1). Across all gROIs, MCEs cover about 81% of coding sequence, 37% of UTR sequence (16-fold and 7-fold enrichments, respectively, compared with the expected coverage if the predicted conserved elements were distributed randomly across 5% of the genome), 5% of intronic sequence and 4% of intergenic sequences (Figure 3b). Considering the other direction, among the 2.5 Mb of MCEs identified, the majority mapped to coding regions (34%) and introns (31%), with smaller proportions mapping to intergenic (22%) and UTR (13%) regions (Figure 3c). For further analysis, we classified MCEs by their 'relative genic position' (Figure 3a) in the automated pipeline. We divided exon-associated conserved elements into those that are completely exonic, those that are partially exonic and span exon-intron boundaries, and those that are associated with UTRs; whereas non-exon-associated elements were divided into those that are intergenic and those that are intronic.

### Duplicated MCEs among gROIs

The MCEs represent conserved genomic segments found across different species. It is also common to find duplicated genomic segments within the same genome. These duplicated segments can arise through a multitude of genomic events including chromosome duplication, gene duplication, retroviral elements, among others. It is possible that these duplicated genomic segments may also be conserved across different species, forming what we refer to as 'duplicated MCE' (dMCE) subsequences. The dMCEs represent ancestrally duplicated genomic elements that have been independently conserved in disparate species, most likely due to stabilizing selection. Such elements are unusual in that duplicated genomic segments typically diverge, either through neutral degeneration or through positive selection for functional diversification [30,31]. Thus, dMCEs may represent small parts of ancient duplications that are preserved because of their core functional importance, for example as regulatory elements that interact with a common trans-regulator.

To investigate the dMCE pairs we used BLASTN [32] for comparison of all 26,000 MCEs with themselves. We identified 2365 significant (E value ≤ 10^-2^) high scoring dMCE pairs within 6% (1723/26,000) of all MCEs. We classified the genomic subsequences comprising dMCEs by their relative genic position (Table 2). The vast majority of dMCE pairs share broad relative genic position; 88% (895/1016) of pairs involve one exon-associated dMCE paired to another exon-associated dMCE, and similarly 88% (1193/1349) of pairs involve one non-exon-associated MCE paired to another nonexon-associated MCE. There were only 1,087 MCEs in the non-exon-associated group, and although small in number (1,087/26,000) this subset of MCEs represents a particularly important group of sequences because they may correspond to potential functional regulatory motifs (see below).

**Figure 4:**
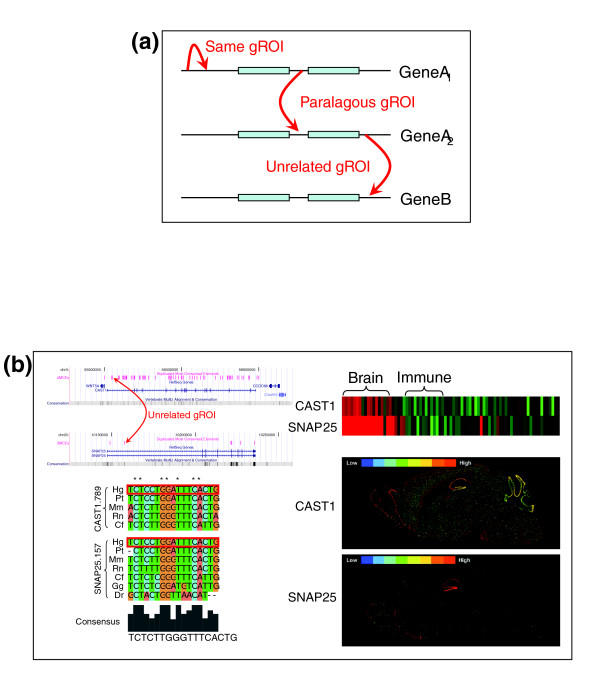
Duplicated most conserved elements. **(a) **A schematic illustration of three classes of dMCEs in a hypothetical two-exon gene is shown. The blue rectangles represent exons of three different two-exon genes, and the red arrows represent the relationship between pairs of duplicated MCEs relative to their gROIs. GeneA_1 _and GeneA_2 _are paralogs in the same gene family, whereas GeneB represents an unrelated gene. The figure shows a local dMCE pair in the same gROI upstream from GeneA_1_, an intronic pair of dMCE elements between the paralagous gROI of GeneA_1 _and GeneA_2_, and an intergenic pair of dMCE elements downstream unrelated genes GeneA_2 _and GeneB. **(b) **Example of a dMCE pair between unrelated genes *CAST1 *(chromosome 3) and *SNAP25 *(chromosome 20) is shown. The pair involves an element in the first intron of *CAST1*(.789) and an element in the last intron of *SNAP25*(.157). Orthologous species shown in the alignments include chimpanzee (*Pan troglodytes *[pt]), dog (*Canis familiaris *[cf]), mouse (*Mus musculus *[mm]), rat (*Rattus norvegicus *[rn]), chicken (*Gallus gallus *[gg]), and zebra fish (*Danio renio *[dr]). Both elements are conserved in mammals, and SNAP25 element exhibits conservation in chicken and zebrafish. Both genes related to these elements exhibit increased expression in brain tissues, and reduced expression in immune tissues and cell types. Both genes also show increased expression in hippocampus and throughout the cortex, although they differ in cerebellum expression as shown by *in situ *expression patterns courtesy of Allen Brain Atlas [19]. dMCE, duplicated most conserved element; gROI, genomic region of interest.

We classified all significant dMCE pairs as mapping to the same gROI, mapping to paralagous gROIs, or mapping to unrelated gROIs (Figure 4a and Table 3). In addition, we also searched for palindromic matches to the same MCE (regions in which the sequence is equivalent when read in either direction). The majority of exon-associated dMCE pairs mapped in and around exons of paralogous gROIs, whereas most non-exon-associated duplicated MCE modules mapped to unrelated gROIs. We found a small number of dMCE pairs shared by paralogous genes. The small proportion of intronic and intergenic dMCE pairs that map to the same gROI reveal that local segmental duplications and palindromes contributed to the evolutionary history of 35 presynaptic genes. Palindromic sequences accounted for 23 of these presynaptic genes (as shown in Additional data file 8).

To test the hypothesis that dMCEs are preserved because of their core functional importance, we compared members of dMCE pairs with the same relative genic position (exonic - exonic, exon-intron boundaries - exon-intron boundaries, UTR - UTR, intergenic - intergenic, and intronic - intronic MCEs) with a set of control unique MCEs (from all gROIs) outside of any dMCE pair. We annotated the MCEs and dMCEs according to the following: whether they mapped to protein domains from ENSEMBL, whether they possessed significant RNA secondary structure, and whether they mapped to public mRNA expressed sequence tags (ESTs) and transcripts clustered by the Database of Transcribed Sequences [33]. The proportion of dMCEs associated with annotated protein domains is significantly greater than that of controls (924/3091 [30%] versus 166/306 [54%]; *P *< 0.001). This is somewhat expected as many presynaptic genes form large gene families that share sequence encoding protein domains. We found the proportion of MCEs associated with the 3'-UTR portion of genes to be significantly enriched for significant RNA secondary structure in dMCE pairs versus unique MCEs (20/65 [31%] versus 18/215 [8%]; *P *< 0.001). The proportion of intergenic dMCE pairs that exhibit evidence of transcription is significantly greater than that of controls (46/3666 [13%] versus 279/6562 [4%]; *P *< 0.001). Thus, members of dMCE pairs, when found in the same relative genic position, exhibit greater evidence of functional association than in control MCEs.

**Figure 5:**
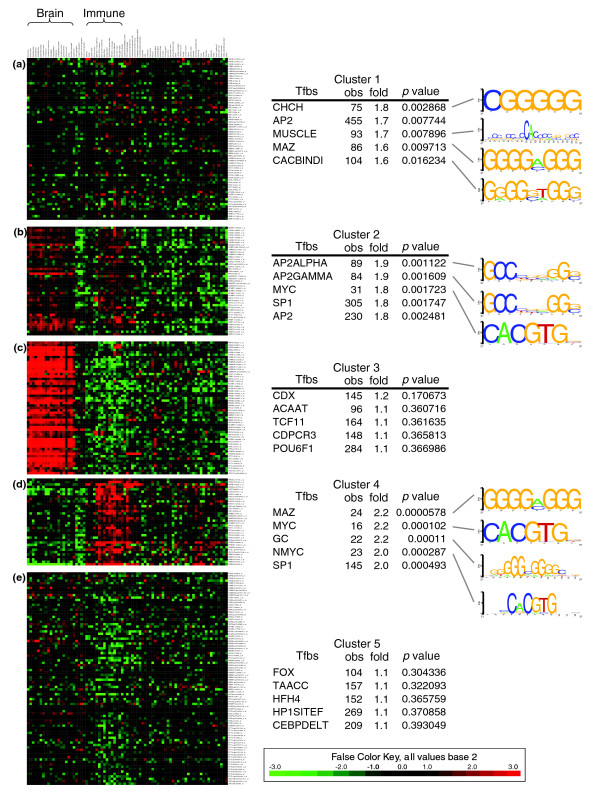
Analysis of coexpressed sets of genes across human tissues and cell lines. The figure shows five clusters of genes with distinct expression profiles from Genomics Institute of the Novartis Research Foundation SymAtlas [17]: **(a) **transcripts with widespread and low-level expression in most tissues/cell types; **(b) **transcripts expressed in brain and immune tissues and cell types but under-expressed in other tissues; **(c) **transcripts with enriched expression in brain tissues and low levels of expression in other tissues; **(d) **transcripts or splice forms enriched in hematopoietic derived immune cell types; and **(e) **transcripts or splice forms under-expressed in immune tissues and cell types. The tables to the right of each expression cluster shows the five most enriched TFBSs found in that cluster, and lists the TFBS name, the observed count number of hits of that TFBS in intergenic and intronic MCEs, the fold increase over that expected by chance, and the significance of enrichment in the cluster. Available PWM logos for all significantly enriched TFBSs (*P *< 0.05) are also displayed. MCE, most conserved element; PWM, positional weight matrix; TFBS, transcription factor binding site.

To investigate potential co-regulation among the (581) presynaptic gene pairs defined by 1,087 intronic and intergenic dMCEs, we analyzed data from a microarray analysis of 79 human nonredundant tissues and cell lines [18] (Figure 5). Expression clustering of transcripts detected by 291 unique oligonucleotide probes on a chip corresponding to 144 presynaptic genes in our dataset identified five distinct expression profiles: transcripts with widespread and low levels of expression in most tissues/cell types; transcripts expressed in brain and immune tissues and cell types but under-expressed in other tissues; transcripts with enriched expression in brain tissues and low levels of expression in other tissues; transcripts or splice forms enriched in hematopoietic derived immune cell types; and transcripts or splice forms under-expressed in immune tissues and cell types. In about one-third of presynaptic genes with expression data (50/144), selected gene probes/oligonucleotides detected different transcripts or expression profiles (Additional data file 9). Nonetheless, in every cluster there is a statistically significant over-representation of pairs of genes sharing at least one common dMCE subsequence (*P *values ≤ 1.4 × 10^-7^). The over-representation ranged from a 7.7-fold enrichment of gene pairs sharing dMCEs in cluster 3 (with 158 gene pairs; Figure 4b and Figure 5c) to a 2.6-fold enrichment in cluster 4 (with 39 gene pairs; Table 4). Thus, the most significantly enriched gene pairs were found in clusters with clear expression in brain tissues (clusters 3 and 4).

### Transcription factor binding sites in MCEs

The MCEs in intergenic and intronic regions of presynaptic genes are candidates for regulatory elements. Therefore, we used 546 positional weight matricies (PWMs) in the TRANSFAC database [34] to search all 26,000 MCEs, annotated by their relative genic position. We found more than 200,000 hits to 338 different transcription factor binding sites (TFBSs). To investigate which TFBS might be over-represented in presynaptic MCEs, we compared the relative occurrence of TFBSs in the subset of intronic and intergenic presynaptic MCEs (which comprise 88% of all MCEs) to a genome-wide randomly sampled set of MCEs. We found enrichment of 16 TFBSs (CRX, LHX3, HNF-6, OCT-1, HFH-8, POU6F1, MEF-2, EVI-1, NKX3A, TTF1, HOXA4, GATA-X, SMAD, BRN-2, RFX1, and TST) in intronic and intergenic presynaptic MCEs. Closer inspection revealed ten enriched TFBSs (OCT-1, LHX3, GATA-X, MEF-2, NKX3A, GR, HNF-6, SMAD, POU6F1, and FOXP3) in the intronic MCEs, ten enriched TFBSs (CRX, LHX3, AP-1, HFH-8, RFX1, OCT-1, MEIS1B:HOXA9, TCF-4, PBX-1, and TST-1) in the upstream intergenic MCEs, and only two enriched TFBSs (RFX1 and S8) in the downstream intergenic MCEs of presynaptic genes. Thus, there is a significant enrichment in upstream and intronic MCEs but not in MCEs downstream of presynaptic genes. Several of these transcription factors are known to be involved in synaptogenesis or neuronal function such as Crx [35], Lh3x [36], GR[37,38], SMAD [39,40], and OCT-1 [41]. Interestingly, of the 16 enriched TFBSs, 11 (CRX, FOXP3, GR, HOXA4, LHX3, NKX3A, POU6F1, RFX1, S8, SMAD, and TTF1) were located within duplicated MCEs discussed above.

To investigate whether particular TFBSs are differentially associated with the five expression clusters of presynaptic genes, we calculated the frequency of occurrence of each TFBS within intronic and intergenic MCEs associated with genes in each of the five clusters. We then quantified statistical differences in the frequency of each TFBS across the five clusters (Figure 5a-e) to identify 32 TFBSs with a statistically significant differential frequency of occurrence across all the expression clusters (*P *< 0.05 by χ^2 ^distribution after correcting for multiple tests; Additional data file 10). For each of these 32 TFBSs, we carried out a *post hoc *contrast between each cluster and the remaining clusters to assess whether any of the TFBSs were particularly associated with a single cluster (Figure 5). The most statistically significantly over-represented TFBS were found in MCEs in genes from the three clusters exhibiting over-expression in immune tissues/cell types (clusters 4, 2 and 1, in order of decreasing significance). Furthermore, the top five enriched TFBSs in all three of these clusters have statistically significant differences in their frequencies (*P *< 0.05 by Normal distribution) across the five expression clusters. We did not detect a significant enrichment of TFBSs in expression clusters with transcripts under-expressed in immune tissues/cell types (clusters 3 and 4). Thus, the statistical significance of TFBS enrichment in presynaptic genes appears to be correlated with over-expression in immune tissues/cell types.

### Analysis of large MCEs

A genome-wide study found that the most highly conserved elements in vertebrates are hundreds or thousands of bases long and show extreme levels of conservation [28,42]. Our in-depth analysis focused on 88 genes that harbor the longest 504 MCEs (Additional data file 11) ranging from 360 bp (eight elements from *CAMK2G*, *NCAM1*, *NLGN1*, *NRXN1*, and *RAB6IP2*) to over 1.2 kb (*CASK*), which we refer to as 'large MCEs' (LMCEs). These elements encompass exons (37 exonic and 23 partial exonic) and UTRs (22 UTR-5' and 38 UTR-3'), with a majority found in intergenic (204) and intronic (180) genomic regions. We found that 35% (8/23) of partial exonic LMCEs that span intron-exon boundaries map to known alternatively spliced exons in *CAMK2G *(.113 and .140), *CASK *(.333), *NLGN3 *(.19), *NRXN2 *(.143), *RIMS1 *(.437), and *RIMS2 *(.193). As expected, the number of LMCEs identified is proportional to the size of gROI analyzed.

**Figure 6:**
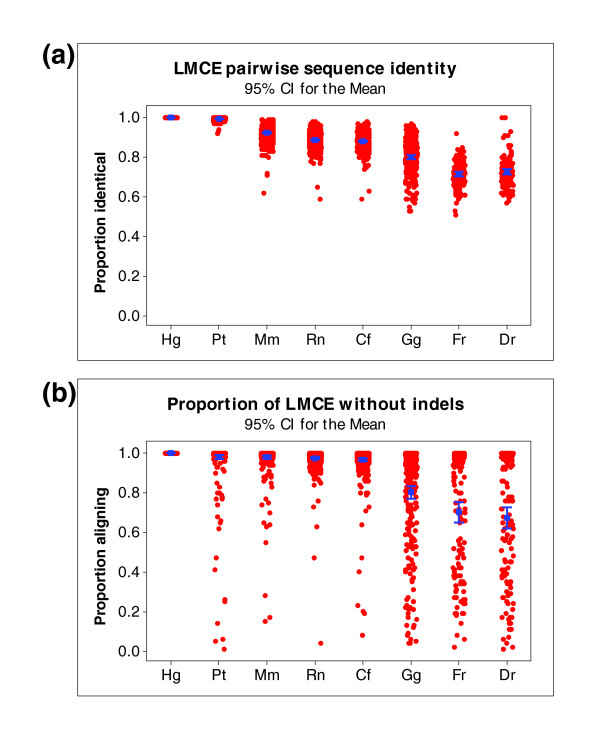
Conservation of large most conserved elements across species. **(a) **The red data points show conservation in the LMCEs (defined as MCEs ≥360 base pairs) plotted as pair-wise identities across all species in the underlying seven-way vertebrate whole-genome alignments with human. The blue lines indicate the mean and standard errors of the mean for each species relative to human. Orthologous species include chimpanzee (*Pan troglodytes *[pt]), dog (*Canis familiaris *[cf]), mouse (*Mus musculus *[mm]), rat (*Rattus norvegicus *[rn]), chicken (*Gallus gallus *[gg]), zebra fish (*Danio renio *[dr]), and puffer fish (*Fugu rubripes *[fr]). **(b) **Species are plotted against the proportion of total LMCE length showing homologous sequence devoid of insertions or deletions in the underlying whole-genome multiple alignment. LMCE, large most conserved element.

The average pair-wise identity between human LMCEs and other species (Figure 6a) was highest in mammals (99.3 ± 0.0% in chimpanzee, followed by 92.2 ± 3.9% in mouse, 88.5 ± 4.2% in rat, and 88.0 ± 4.4% in dog), followed by chicken (80.0 ± 8.6%), and was lowest in fish (72.4 ± 7.6% in zebra fish and 71.3 ± 6.5% in puffer fish). Although the average percent identity of aligned sequence between humans and other species is high (≥88% in mammals and ≥71% in nonmammals, excluding indels), the proportion of putative homologous sites was much more variable (Figure 6b) and diverged more between mammals and nonmammals (≥93% in mammals and ≥69% in nonmammals). In mammals the percentage of well aligned putative homologous sites is higher on average than the percent identity of the nucleotides within the homologous sites (97.9 ± 11.0% for chimpanzee, 98.0 ± 7.5% in mouse, 97.4 ± 5.9% in rat, and 96.6 ± 9.0% in dog). However, for nonmammals the percentage of well aligned putative homologous sites is on average less than the percent identity of nucleotides within the homologous sites except in chicken (80.1 ± 26.0% in chicken, 70.1 ± 30.3% in puffer fish, and 67.3 ± 31.2% in zebra fish). Thus, the percentage of well aligned putative homologous sites seems to be directly proportional to the evlolutionary distance to humans which may suggest that indels become an increasingly important divergence mechanism at longer evolutionary times.

We defined elements as conserved if more than half of their sequence length exhibited significant site homology to some subsequence in available vertebrate genomes (Additional data file 11). Although virtually all (503/504) LMCEs were conserved in mammals, only 48% (241/504) were conserved in nonmammals. Among exon-associated elements, 67% (80/119) were conserved in nonmammals, which decomposed to 97% (35/36), 57% (13/23), and 53%(32/60) for exonic, partial exonic, and UTR LMCEs, respectively. Among elements associated with UTRs, 68% (26/38) are conserved in the 3'-UTR of nonmammals as compared with only 27% (6/22) in the 5'-UTR, highlighting the relative functional importance of the 3'-UTR in this set of genes. Among non-exon-associated elements, 42% (161/385) were conserved in nonmammals. As fish-mammal genomic comparisons have proved to be powerful in identifying conserved noncoding elements that are likely to be *cis*-regulatory in nature [43], we specifically examined elements conserved in fish. Out of all the LMCEs, 21% (106/504) were conserved in fish with approximately equal proportions being exon-associated (55/106) and non-exon-associated (51/106).

We performed BLAST analysis (E value ≤ 10^-4^) of all intronic and intergenic LMCEs to search for orthologous sequence in the *Drosophila *genome. In general, mammalian LMCEs are not present in the *Drosophila *genome, except in a few cases (CALM3.19, CAMK1G.865, NSF.209, SV2C.10, NLGN1.716, and SYN.152) when these LMCEs represent short fragments of *Drosophila *protein coding genes, such as ribosomal proteins (NLGN1.716 and NSF.209), cytoskeletal actin proteins (SYN2.152), and the metabolic glyceraldehyde 3 phosphate dehydrogenase enzyme (CALM3.19).

**Figure 7:**
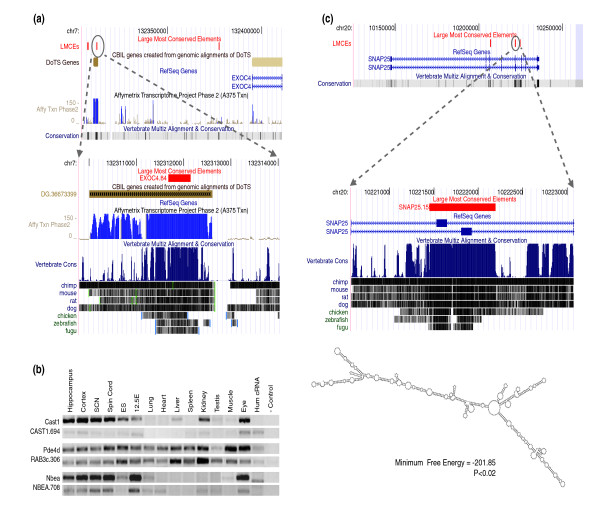
Evidence for transcription and RNA stability in LMCEs. A complete representation of the positions and analysis of MCEs within all 46 megabases analyzed is available via custom tracks in the UCSC Genome Browser [26] through supplemental data. **(a) **A view depicting a transcribed LMCE identified by both DoTS and tiling array data upstream from *EXOC4*(.84) is shown. The LMCE is shown by the red track towards the top and is highly conserved to zebra and puffer fish, whereas DoTS transcripts from clustered mRNA and EST sequences is shown as the next brown track below, and evidence for significant transcription by tiling array data is shown by blue bars on the next track down. **(b) **Expression patterns of LMCEs across tissues were compared with patterns obtained for RT-PCR products generated by priming at exons upstream and downstream of the LMCE. The PCR products were visualized by gel electrophoresis to show similar patterns of expression to the nearby genes. Shown are elements upstream from *CAST*(.694), downstream from *RAB3C*(.306) in the neighboring *PDE4D *gene, and in an internal intron of *NBEA*(.708). **(c) **A view depicting a LMCE with significant stable RNA secondary structure spanning alternatively spliced exons of *SNAP25*(.159) is shown along with the optimal minimum free energy RNA structure. Intronic portion of this LMCE are also highly conserved to zebra and puffer fish. DoTS, Database of Transcribed Sequences; EST, expressed sequence tag; LMCE, large most conserved element; RT-PCR, reverse transcription polymerase chain reaction.

We found evidence that a subset of LMCEs represent transcribed elements (nonannotated exons of known synaptic genes or novel transcripts; Additional data file 11). This evidence comes from high-density tiling array data and the Database of Transcribed Sequences (DoTS) [33]. Expression data in eight cell lines established by hybridization to high-density oligonucleotide arrays are available for ten human chromosomes [44]. There were 148 LMCEs transcribed, and among these 87% (103/119) of the exon-associated LMCEs were annotated as transcribed. We confirmed transcription in all (36/36) exonic and most (56/60) UTR elements. Less than half (11/23) of the partial exonic LMCEs at intron-exon boundaries (which by definition have large nontranscribed intronic portions) were annotated as transcribed. We found that 45 (out of 385) non-exon-associated LMCEs had evidence for transcription, of which 15 (12 intergenic and 3 intronic) correspond to known genes in other species, and three intronic elements (EXOC4.381, NLGN1.503, and SNAP25.168) may be missed exons of their respective synaptic genes. We also found six intergenic elements that do not map to any known protein-coding genes (both human and nonhuman). Only one of these elements shows any protein coding potential (SYT13.83), suggesting that the rest may be novel non-protein-coding genes (EXOC5.359, RAB3C.156, SYN2.235, SYT16.215, and UNC13A.87), one of which (SYN2.235) identifies transcripts nested antisense to the known presynaptic gene. Finally, we provide evidence for transcription for five additional non-exon-associated LMCEs out of 20 we tested by reverse transcription polymerase chain reaction (RT-PCR; Figure 7b).

Highly conserved sequences that form RNA secondary structures may participate in the regulation of gene expression, splicing, cleavage, or post-transcriptional control [45,46]. We investigated the significance of predicted RNA minimum free energy (MFE) for MCEs surrounding 150 synaptic genes. Among 504 LMCEs, we identified 139 significant thermodynamically stable secondary structures (Figure 7c). These structures were found in and around exons (38/139) and in intronic and intergenic highly conserved elements (101/139). We found 15 non-exon-associated elements predicted to be transcribed that were also predicted to have significant secondary structure.

## Discussion

We conducted a comprehensive analysis of sequence conservation patterns in gROIs of 150 genes involved in synaptic function. This analysis resulted in the identification of a significant number of novel highly conserved sequence elements that are likely to regulate the expression, translation, or function of these genes. Our inventory of conserved genomic elements is compiled in the SynapseDB, a database that contains the genome sequence and expression data for synaptic genes along with results of comparative sequence analysis reported here. Among other information, this database includes the following: phylogenetic analysis of several large gene families; expression patterns obtained by *in situ *hybridization and microarray analysis of multiple splice forms per gene; conserved regions in the entire gROI, including intronic regions and intergenic regions; classification of conserved elements based on their relative genic position and other functionally significant features; and classification of elements based on their homology to other presynaptic genes or paralogous gene regions. The identification of a large number of putative novel regulatory elements in a subset of synaptic genes provides an important list of novel functional 'targets' for gene regulation during nervous system development and for dysregulation in disease. To our knowledge, this is the first report on large-scale identification and computational characterization of genomic elements in synaptic genes. However, our effort complements a more comprehensive attempt of the Genes to Cognition (G2C) program to establish a framework for studying genes, brain, and behavior in order to link basic molecular and proteomic research from genomes and experimental genetic organisms with human clinical studies of cognition [47,48].

### Protein sequence conservation

Our evolutionary analysis of human-mouse-rat orthologs, measured by the d_N_/d_S _ratio, showed that the average d_N_/d_S _for 139 presynaptic genes is fivefold lower than that of a comprehensive genomic survey of over 15,000 homologous pairs of human-mouse transcripts (0.072 versus 0.413). This is consistent with a previous proposal [49] and findings that brain-specific and neuron-specific proteins are under stronger purifying selection pressure than genes expressed, for example, in the liver [24]. The average rate (mean ± standard error) of relative synonymous substitution (d_S_) for 139 synaptic genes is 0.558 ± 0.016, which represents a statistically significant (*P *< 0.001), almost fourfold reduction in the genome-wide average d_S_. Therefore, the reduced d_N_/d_S _ratio is not due to an increased neutral mutation rate in presynaptic genes. The presynaptic genes are found on almost all human chromosomes (except chromosome 21), and in 85% (128/150) of genes the distance to the nearest synaptic gene exceeds 500 kb. Thus, it is highly unlikely there might be a chromosomally localized mutational bias specific to presynaptic genes.

The significant reduction in the rate of synonymous substitutions is unusual and suggests that selective forces act at the level of the mRNA, such as selection for translational efficiency or accuracy through biased codon usage, selection for regulation involving the primary transcript such as by miRNA-type mechanisms, or selection for other primary transcript function such as binding to RNA binding proteins (RBPs). We found no difference in the 'effective number of codons' statistic [50] as a measure of codon bias between presynaptic genes and the genomic survey (data not shown), and so there is no evidence that presynaptic genes are preferentially selected for translational efficiency or accuracy than other genes. The reported mechanisms of primary transcript-dependent regulation (for example, by miRNAs or RBPs) is too sparsely annotated in the genome to assess whether presynaptic genes may be unusual in this regard. However, numerous miRNAs are thought to function in neuronal regulation (for review, see Kosik and Krichevsky [11]), and many studies have implicated RBPs in regulation of synaptic function (for review, see Ule and Darnell [51]) in phenomena such as selective translation of mRNAs at synapses [52] and brain-specific alternative splicing [12]. In addition, supporting the idea that presynaptic genes are under purifying selection for primary transcripts, we found an unusual number of potential RNA secondary structures with significant evidence for stability. The potential for functionally important secondary structure is consistent with the idea that the primary transcripts of the genes involved in synaptic transmission have important functional significance, either in regulation or in interaction with RBPs.

### Duplicated MCE modules

The analysis of more than 26,000 MCEs identified in our set of 150 synaptic transmission pathway genes showed that these sequences are nearly unique and mostly do not represent groups of elements with similar sequence. Nevertheless, the MCEs showed a more pronounced pattern of similarities when restricted to genomic regions around paralogous members of a gene family. Paralogous genes that arise from gene duplication events often diverge from each other either because one copy is redundant, and therefore undergoes neutral drift, or because there is selection for new and divergent function. Paralogs that contain between-species conserved sequences most likely have experienced positive selection for new function in an ancestral genome and were subsequently conserved through purifying selection [53], but such paralogs are likely to have diverged from each other in the ancestral genome due to neo-functionalization, subfunctionalization, or degeneration to pseudogenes [30,31]. Thus, there is no *a priori *expectation that paralogous genes will contain MCEs that are also similar to each other within the same genome.

Our results show that exonic dMCEs are enriched for annotated protein domains; within coding sequences, dMCEs may indicate a 'functional backbone' of a protein, such as transmembrane domains or DNA binding domains. The 1,087 cases of intronic and intergenic dMCEs may represent common regulatory elements of shared trans factors. Indeed, we found significant enrichment for gene pairs that were over-expressed in brain tissues as well as for gene pairs under expressed in immune tissues and cells, which indicates that these noncoding dMCEs may have regulatory potential. Our findings suggest that these dMCEs arise only in the rare cases (only 6% of all MCEs) in which the shared conserved elements represent some core function that cannot diverge from each other in the initial positive selection for new function because of biophysical or chemical constraints.

### Comparison with ultraconserved elements

Our selection and in-depth sequence analysis of exceptionally long conserved sequence elements can be compared with the identification of ultraconserved elements identified by a genome-wide search for regions longer than 200 bp with 100% identity between the human, mouse, and rat genomes [42]. LMCEs selected in our study are less perfectly conserved than ultraconserved elements, exhibiting 80% or greater pair-wise identity between humans and mammals and 70% or greater identity between human and nonmammalian sequences. About 10% (51/504) of our LMCEs are annotated as non-exon-associated and conserved over the majority of their length in fish, suggesting a functional basis for their phylogenetically broad conservation. We found that LMCEs fall within all three categories relative to annotated genic structure (exonic, intronic, and intergenic). Several LMCEs correspond to large exons such as the particularly long exonic MCEs ranging from 600 to 800 bp in the NLGN family (NLGN1.667, NLGN1.668, NLGN2.37, NLGN3.61, NLGN4X.24), and in *BSN*(.98), *EXOC8*(.5), *PCLO*(.119, .162), and *RAB6IP2*(.20). We found that 35% (8/23) of partial exonic LMCEs that span intron-exon boundaries map to known alternatively spliced exons in *CAMK2G*, *CASK*, *NLGN3*, *NRXN2*, *RIMS1*, and *RIMS2*. In the case of *SNAP25*, the LMCE spans the exon-intron boundaries of two tandemly arranged exons (Figure 7c). A molecular switch between these two exons at three weeks of age is important for survival and synapse remodeling after neural injury [54]. It is striking that conserved elements surrounding alternatively spliced exons are also marked as elements with significant thermodynamic stability for predicted RNA secondary structure. Finally, for the most abundant class of LMCEs, those in the intergenic and intronic regions, we provide evidence that they correspond to missed exons of alternative splice forms of two synaptic genes (*EXOC4*, *NLGN1*, and *SNAP25*) and novel putative non-protein-coding genes around six synaptic genes (*CASK*, *EXOC5*, *RAB3C*, *SYN2*, *SYT13*, and *SYT16*). The function of these transcribed elements and remaining LMCEs with no evidence for transcription remains elusive, although we assume that a high selective pressure may reflect their regulatory or structural roles.

### Noncoding elements, single nucleotide polymorphisms, and neurologic and psychiatric illness

Comparative sequence analysis and identification of highly conserved elements in noncoding regions of presynaptic genes will have immediate application to ongoing human genetics studies. We believe that such comparative analysis provides a more comprehensive inventory of genomics elements that are functionally active in a pathway or a compartment such as the synapse. An extended inventory is especially important in providing functional targets for disease-gene association studies, which often discover functional variants in non-protein-coding regions. For example, several proteins of the *N*-methyl-D-aspartate receptor complex have been linked to cognitive dysfunction or associated with mental illness [48]. However, in several cases single nucleotide polymorphisms with the strongest association fall within the intergenic or intronic gene regions [55,56]. Systematic identification and computational analysis of highly conserved elements surrounding these and other synaptic genes can uncover either an adjacent novel gene or *cis*-acting polymorphisms resulting in the modulation of synaptic function in cognition and mental illness. Therefore, we suggest that a comprehensive comparative analysis may be an essential complement of genome-wide association studies of complex diseases.

## Materials and methods

### Ortholog identification, phylogenetic analysis, and tests for protein evolution

Orthologs for human gene family members were found using a combination ENSEMBL's Compara database (v37.35j) and the best reciprocal BLAST hit procedure, as described by Tatusov and coworkers [16]. Clustal W [57] was used to align coding sequence (CDS) from corresponding RefSeq annotations of all transcripts between *Homo sapiens *(human; May 2004, NCBI Build 35, UCSC hg17), *Mus musculus *(mouse; May 2004, NCBI Build 33, UCSC mm5), and *Rattus norvegicus *(rat; June 2003, Baylor College of Medicine HGSC v3.1, UCSC rn3). From these multiple alignments, we calculated the ratio of synonymous to nonsynonymous substitutions (d_N_/d_S_) [58] between human-mouse and human-rat pair-wise alignments as a measure of selection pressure. d_N _is the relative rate of nonsynonymous mutations per nonsynonymous site, whereas d_S _is the relative rate of synonymous mutations per synonymous site. Their ratio indicates the degree of selection pressure. Multiple alignments were also used for both the neighbor-joining and parsimony methods for phylogeny reconstruction on 1,000 bootstrap permutations using the MEGA3 program for molecular evolutionary genetic analysis [59]. The trees shown in Additional data files 2 to 5 are drawn after collapsing branches supported by bootstrap values less than 65%, and each tree is labeled as either 'neighbor-joining' or 'maximum parsimony'.

### Comparative analysis pipeline

We used genomic sequence from human (May 2004, NCBI Build 35, UCSC hg17) and gene annotations from RefSeq (both human and nonhuman), ENSEMBL, and 'known genes' available via the UCSC Genome Browser database as of 1 May 2006. This set of annotations was used to identify conservatively upstream and downstream regions around 150 presynaptic genes in order to define gROIs. For 11 genes we could not identify a unique upstream region because an adjacent nonpresynaptic gene overlapped the beginning of the presynaptic gene, and similarly for 22 genes we could not identify a unique downstream gene because of overlapping adjacent nonpresynaptic genes. Furthermore, these multiple gene annotations were merged into a common annotation such that inconsistencies were resolved by giving priority to UTRs, coding sequence (CDS), and intron classes in the order listed; for example, if a base was annotated as both CDS and UTR, then it was counted as belonging to the UTR class. This common annotation was used for relative genic characterization of the MCEs. Broadly, we divide elements into exon-associated elements and non-exon-associated elements. We further divided exon-associated elements into three putative functional groups: 'exonic', those completely contained within an exon; 'partial exonic', those that span an intron-exons boundrary; and 'UTR', those that include the 3'-UTR or 5'-UTR regions. We classified non-exon-associated MCEs into two subgroups: 'intergenic', those located outside any annotated gene; and 'intronic', those contained in the intron of an annotated gene. To identify sequence elements with possible novel regulatory roles, we removed about 3000 exon-associated MCEs that corresponded to exons of neighboring transcripts overlapping and in trans to the presynaptic gene, leaving a set of about 26,000 MCEs on which the analysis is based.

We sourced our MCEs from the 'most conserved' track from UCSC genome browser. These elements are based on analysis of an eight-genome-wide multiple alignment by phastCons, a phylogenetic hidden Markov model that identifies conserved elements in multiply aligned sequences based on the process by which nucleotide substitutions occur at each site in a genome and how this process changes from one site to the next [28]. Genome sequence of human (*Homo sapiens*), chimpanzee (*Pan troglodytes*), dog (*Canis familiaris*), mouse (*Mus musculus*), rat (*Rattus norvegicus*), chicken (*Gallus gallus*), zebra fish (*Danio renio*), and puffer fish (*Fugu rubripes*) were used to generate alignments. Annotations of MCEs included transcription from the Database of Transcribed Sequences (DoTS) [33], which clusters public mRNA and EST data as well as high-density tiling array data (as described by Cheng and coworkers [44]) available through the 'Affymetrix Transcriptome Project phase 2' track from Genome Browser. In addition, we annotated protein domains from the ENSEMBL core database, coding potential from predicted evolutionarily conserved protein-coding exons from multiple alignments available through the 'exoniphy human/mouse/rat/dog' track on Genome Browser, and statistically significant stable RNA secondary structure as described in Results, above.

### Identification of duplicated MCE pairs and expression clustering

We used BLASTN [32] to conduct a comparison of all 26,000 MCEs with themselves using a significance threshold of E value ≤ 10^-2 ^after masking all known repeats with RepeatMasker [60]. We refer to the duplicated MCE subsequence comprising significant high scoring BLAST pairs as duplicated MCE (dMCE) modules. To investigate whether dMCE modules are preserved because of some functional importance, we compared members of dMCE pairs whose members are in the same relative genic position with a set of control unique MCEs (from all gROIs) outside any dMCE pair. While controlling for relative genic position, the significance of the proportion of annotations among duplicated versus unique MCEs was calculated by Fisher's exact test and the 2-proportion test using the MINITAB v14 statistical package. Annotations of MCEs included transcription from DoTS and high-density tiling array, domains from the ENSEMBL core database, and significant RNA secondary structure.

For clustering of significantly regulated genes, we used Michael Eisen's Cluster v2.11 [61] implementation in the BioPython 1.42 distribution [62], and we used Java TreeView 1.0.13 [63] to visualize the data. We used expression values from the Genomics Institute of the Novartis Research Foundation (GNF) data from two replicates across 79 human tissues and cell lines [18] on Affymetrix microarrays, which are scaled and normalized in the 'GNF Expression Atlas 2' track from Genome Browser. Although we identified 359 different probes that overlapped our genes, we excluded 12 probes that cross-hybridize with paralogs and 56 that detect antisense transcripts. We clustered 291 unique probe sets that interrogated 144 different genes into five distinct clusters, as described. Genes were then mapped directly to expression clusters with most genes assigned to more than one expression cluster. We used dMCEs to define unique pairs of genes that are in the same expression cluster, and for each cluster we calculated the binomial probability for the observed number of gene pairs. When different probesets for the same gene show divergent results we assume that they interrogate different splice forms, and treat each probeset as a distinct transcript (we double count) for statistical consistency.

### Analysis of transcription factor binding sites

We searched the MCEs in the human genome for putative transcription factor binding sites (TFBSs) using the 546 vertebrate positional weight matrices (PWMs) in the TRANSFAC database v8.4 [34]. We identified the putative TFBSs using our previously described search tool PWM_SCAN [64] with a *P *value threshold of 10^-5 ^(we expect a better match once every 10 kb on average in the genome). In order to assess whether specific TFBSs are enriched in the MCEs near presynaptic genes, we used as control a length-matched set of MCEs from the human genome, not overlapping the presynaptic MCEs. For each presynaptic MCE, we randomly selected 10 regions of the same length contained in other nonpresynaptic MCEs in the genome. For each PWM, we randomly select 1,000 samples from the control in which each sample consists of 1-1 length-matched MCEs. The fraction of 1,000 samples in which the PWM frequency exceeds that in the presynaptic MCEs provides an estimate of significance of PWM enrichment in the presynaptic MCEs. We use a *P *value threshold of 0.002 for enrichment significance, and we filter the enriched PWMs to ensure that we only report PWMs that are sufficiently distinct from each other.

To analyze enrichment of TFBSs within clusters of expressed genes, for each TFBS we first used a χ^2 ^test to identify TFBSs whose relative frequencies across clusters are significantly different. As we are testing multiple TFBSs, we use a Bonferroni correction [65] at a significance level of α = 0.05. Subsequently, for the five most over-represented TFBSs in each cluster, we test for statistical significance of by calculating a *Z *score for TFBS enrichment. We assume that the relative enrichment of TFBSs in clusters is normally distributed and we justify this assumption because the natural log of the relative enrichment (log likelihood) of every TFBS across every cluster is normal (*P *< 0.005 by Anderson-Darling test). Again, when different probes for the same gene fall into divergent expression clusters, we assume that they interrogate different splice forms and treat each probeset as a distinct transcript and double count for statistical consistency. Finally, PWM logos generated from equal-length Transfac hits found within clusters were generated via WebLogo [66] for all statistically significant cluster-specific enriched TFBSs.

### Significant RNA secondary structure

The RNAfold program [67] was used to perform a screen of secondary structures by calculating the minimal free energy for all MCEs longer than 50 bp. The minimal free energy for each MCE was compared with those of 1,000 random permutations of the original sequence to evaluate its potential to form stable secondary structures by a permutation test.

### RT-PCR analysis

RT-PCR analysis was carried out on ABI Prism 7900HT sequence detection system (Applied Biosystems, Foster City, CA, USA). Five micrograms of total RNA from multiple tissues were converted to first-strand cDNA using Superscript II, (Invitrogen Corporation, Carlsbad, California 92008, USA), reverse transcriptase primed using random oligomers. PCR primers were selected to cover LMCEs. Expression patterns of these LMCEs across tissues was compared with patterns obtained for RT-PCR products generated by priming at exons upstream and downstream of the LMCE. The PCR products were visualized by gel electrophoresis.

## Additional data files

The following additional data are available with the online version of this paper. Additional data file 1 is a document providing an overview of gROIs. Additional data file 2 is a figure illustrating VAMP phylogeny. Additional data file 3 is a figure illustrating RAB phylogeny. Additional data file 4 is a figure illustrating STX phylogeny. Additional data file 5 is a figure illustrating SYT phylogeny. Additional data file 6 is a figure including a RAB tree with superimposed expression profiles. Additional data file 7 is a figure showing the distribution of most MCE lengths. Additional data file 8 tabulates palindromes found within MCE subsequences. Additional data file 9 tabulates the differential expression of genes. Additional data file 10 tabuilates the frequency of TFBSs across clusters of coexpressed genes. Additional data file 11 tabulates the large MCEs (LMCEs) identified.

## Supplementary Material

Additional data file 1 Overview of genomic regions of interest (gROIs) Click here for file

Additional data file 2 VAMP phylogeny Click here for file

Additional data file 3 RAB phylogeny Click here for file

Additional data file 4 STX phylogeny Click here for file

Additional data file 5 SYT phylogeny Click here for file

Additional data file 6 RAB tree with superimposed expression profiles Click here for file

Additional data file 7 Distribution of most conserved element (MCE) lengths Click here for file

Additional data file 8 Palindromes found within most conserved element (MCE) subsequences Click here for file

Additional data file 9 Differential expression of genes Click here for file

Additional data file 10 The spreadsheet contains two worksheets. The 'chisq' worksheet lists transcription factor binding sites (TFBSs) differentially associated with the five expression clusters of presynaptic genes in decreasing order of statistical significance. The worksheet lists the TFBSs, the counts of occurrence of the TFBS in clusters 1 to 5, the expected number of TFBSs in clusters 1 to 5, the nominal c^2 ^*P *value of observing such TFBS frequencies, and the Bonferroni-corrected *P *value. The 'z score' worksheet lists the TFBSs associated with a given expression profile in order of decreasing enrichment. The table lists the expression cluster number (1 to 5), the count of TFBSs across all clusters, the input size (base pairs) of genome queried for TFBS hits in the given cluster, the expected number of TFBS hits in the given cluster, enrichment, the natural log of the enrichment (the log likelihood), the z score, the *P *value associated with seeing up to the observed number of TFBSs in the given cluster, and the *P *value associated with seeing at least the observed TFBS in the given cluster (1 - *P *value). Click here for file

Additional data file 11 Large most conserved elements (LMCEs) identified Click here for file

## Figures and Tables

**Table 1:** All genes analyzed

#	Gene	#	Gene	#	Gene	#	Gene	#	Gene
1	*AMPH*	31	*EXOC1*	61	*RAB3GAP1*	91	*STX5A*	121	*SYT2*
2	*APBA1*	32	*EXOC2*	62	*RAB5A*	92	*STX6*	122	*SYT3*
3	*APBA2*	33	*EXOC3*	63	*RAB5B*	93	*STX7*	123	*SYT4*
4	*APBA3*	34	*EXOC4*	64	*RAB5C*	94	*STX8*	124	*SYT5*
5	*ASPM*	35	*EXOC5*	65	*RAB6IP2*	95	*STX10*	125	*SYT6*
6	*BSN*	36	*EXOC6*	66	*RABAC1*	96	*STX11*	126	*SYT7*
7	*BZRAP1*	37	*EXOC7*	67	*RABGEF1*	97	*STX12*	127	*SYT8*
8	*CALM1*	38	*EXOC8*	68	*RABGGTA*	98	*STX16*	128	*SYT9*
9	*CALM2*	39	*GDI1*	69	*RABGGTB*	99	*STX17*	129	*SYT10*
10	*CALM3*	40	*GDI2*	70	*RABIF*	100	*STX18*	130	*SYT11*
11	*CALML3*	41	*GZMB*	71	*RIMBP2*	101	*STX19*	131	*SYT12*
12	*CALML4*	42	*NAPA*	72	*RIMS1*	102	*STXBP1*	132	*SYT13*
13	*CALML5*	43	*NAPB*	73	*RIMS2*	103	*STXBP2*	133	*SYT14*
14	*CALML6*	44	*NAPG*	74	*RIMS3*	104	*STXBP3*	134	*SYT15*
15	*CAMK1*	45	*NBEA*	75	*RIMS4*	105	*STXBP4*	135	*SYT16*
16	*CAMK1D*	46	*NCAM1*	76	*RPH3A*	106	*STXBP5*	136	*SYT17*
17	*CAMK1G*	47	*NLGN1*	77	*SCAMP1*	107	*STXBP6*	137	*SYTL1*
18	*CAMK2A*	48	*NLGN2*	78	*SCAMP2*	108	*SV2A*	138	*SYTL2*
19	*CAMK2B*	49	*NLGN3*	79	*SCAMP3*	109	*SV2B*	139	*SYTL3*
20	*CAMK2D*	50	*NLGN4X*	80	*SCAMP4*	110	*SV2C*	140	*SYTL4*
21	*CAMK2G*	51	*NLGN4Y*	81	*SCAMP5*	111	*SVOP*	141	*UNC13A*
22	*CAMK2N1*	52	*NRXN1*	82	*SLC30A3*	112	*SYN1*	142	*UNC13B*
23	*CAMK2N2*	53	*NRXN2*	83	*SLC30A4*	113	*SYN2*	143	*UNC13C*
24	*CAMK4*	54	*NRXN3*	84	*SNAP25*	114	*SYN3*	144	*UNC13D*
25	*CASK*	55	*NSF*	85	*SNAPAP*	115	*SYNGR1*	145	*VAMP1*
26	*CAST*	56	*PCLO*	86	*SNCA*	116	*SYNGR2*	146	*VAMP2*
27	*CAST1*	57	*RAB3A*	87	*STX1A*	117	*SYNGR3*	147	*VAMP3*
28	*DMXL2*	58	*RAB3B*	88	*STX1B2*	118	*SYNGR4*	148	*VAMP4*
29	*DNM1*	59	*RAB3C*	89	*STX3A*	119	*SYP*	149	*VAMP5*
30	*EPIM*	60	*RAB3D*	90	*STX4A*	120	*SYT1*	150	*VAMP8*

The table lists the gene names for all 150 genes analyzed.

**Table 2:** Distribution of dMCEs by paired relative genic structure

Type/type	Exonic	Partial exonic	UTR (5')	UTR (3')	Intergenic (5')	Intergenic (3')	Intronic	Grand total
Exonic	651	3	43	51	20	6	42	816
Partial exonic	2	9	2			1	7	21
UTR (5')	45	2	13	1	2	1	5	69
UTR (3')	54		1	18	10	7	20	110
Intergenic (5')	22	1	2	12	183	57	159	436
Intergenic (3')	7		4	6	48	53	72	190
Intronic	64	6	3	29	196	74	351	723
Grand total	845	21	68	117	459	199	656	2,365

Counts by relative genic structure of members of paired dMCEs are shown. Exon-associated elements are type 1 and non-exon-associated elements are type2. Type 1 MCEs are further decomposed into three putative functional groups: type 1a (exonic), those completely contained within an exon; type 1b (partial exonic), those that span an intron-exons boundrary; and type 1c (UTR), those that include the 3'-UTR or 5'-UTR regions. Type 2 MCEs are divided into two subgroups: type 2a (intergenic), those located outside any annotated gene; and type 2b (intronic), those contained in the intron of an annotated gene. dMCE, duplicated most conserved element; UTR, untranslated region.

**Table 3:** Distribution of dMCEs by gROI relation

Type/gROI	Same	Paralagous	Unrelated	Grand total
Exonic	26	666	124	816
Partial exonic	4	13	4	21
UTR (5')	3	41	25	69
UTR (3')	3	70	37	110
Intergenic (5')	37	7	392	436
Intergenic (3')	33	14	143	190
Intronic	120	60	543	723
Grand total	226	871	1,268	2,365

The relationship between genic structure of and the gROI relation of dMCE pair members is shown. The genic structure of the (BLAST) reference member of significant dMCE pairs is shown. The gROI relation of dMCE pairs was classified as mapping to the same gROI (same), mapping to paralagous gROIs (paralagous), or mapping to unrelated gROIs (unrelated). dMCE, duplicated most conserved element; gROI, genomic region of interest.

**Table 4:** Enrichment of gene pairs defined by dMCEs across gene expression clusters

Cluster #	Observed gene pairs	Expected gene pairs	Enrichment	*P *value
3	268	34.7	7.7	2.99E-154
2	91	18.6	4.9	1.16E-34
5	321	71.9	4.5	2.50E-121
1	158	49.6	3.2	1.12E-37
4	39	15.1	2.6	1.41E-07

The enrichment of the number of gene pairs defined by intergenic and intronic dMCEs in different clusters is shown in order of decreasing enrichment. The table lists cluster # (see Figure 5 for cluster expression patterns), the observed number of gene pairs, the expected number of gene pairs by chance, the enrichment of observed gene pairs, and the one-tailed binomial *P *value for the observed enrichment. dMCE, duplicated most conserved element.
