# Supplementary material for: Patterns of sequence conservation in presynaptic neural genes
Source: Genome Biol. 2006 Nov 10;7(11):R105. doi: 10.1186/gb-2006-7-11-r105 (PMC1794582; doi:10.1186/gb-2006-7-11-r105)
Supplement: Additional data file 1 — Overview of genomic regions of interest (gROIs) [file gb-2006-7-11-r105-S1.doc]

# Overview of genomic regions of interest (gROI)

An overview of gROI and most conserved elements (MCEs) for all synaptic transmission genes analyzed. gROI include the genomic region spanning a synaptic gene as well as the surrounding sequence up to the next annotated *cis*-gene in either (5’ or 3’) direction. In respective order, the table lists the gene symbol, the corresponding chromosome, the gROI length, the length of the upstream region, the length of the synaptic gene, the length of the downstream reagion, the proportion of gROI spanned by the synaptic gene, the relative distance from the synaptic gene to the nearest annotated gene (- and + are upstream and downstream of the synaptic gene respectively), the number of MCEs, the total sequence spanned by MCEs, the proportion of gROI spanned by MCEs, and the number of LMCEs (large MCEs; defined as MCEs ≥ 360bp in length).

| **#** | **sym** | **chr** | **gROI(Kb)** | **up(Kb)** | **gene(Kb)** | **down(Kb)** | **p(gene)** | **near(Kb)** | **#MCEs** | **MCE(Kb)** | **p(MCE)** | **#LMCEs** |
| --- | --- | --- | --- | --- | --- | --- | --- | --- | --- | --- | --- | --- |
| 1 | AMPH | 7 | [325.78](http://genome.ucsc.edu/cgi-bin/hgTracks?db=hg17&position=chr7:38172004-38497784&hgt.customText=http://www.neurogenome.org/mcs/tracks/chr7.txt) | [53.52](http://genome.ucsc.edu/cgi-bin/hgTracks?db=hg17&position=chr7:38444261-38497784&hgt.customText=http://www.neurogenome.org/mcs/tracks/chr7.txt) | [247.72](http://genome.ucsc.edu/cgi-bin/hgTracks?db=hg17&position=chr7:38196546-38444260&hgt.customText=http://www.neurogenome.org/mcs/tracks/chr7.txt) | [24.54](http://genome.ucsc.edu/cgi-bin/hgTracks?db=hg17&position=chr7:38172004-38196545&hgt.customText=http://www.neurogenome.org/mcs/tracks/chr7.txt) | 76% | +24.54 | 123 | 8.26 | 3% | 1 |
| 2 | APBA1 | 9 | [317.06](http://genome.ucsc.edu/cgi-bin/hgTracks?db=hg17&position=chr9:69236932-69553995&hgt.customText=http://www.neurogenome.org/mcs/tracks/chr9.txt) | [37.22](http://genome.ucsc.edu/cgi-bin/hgTracks?db=hg17&position=chr9:69516777-69553995&hgt.customText=http://www.neurogenome.org/mcs/tracks/chr9.txt) | [242.02](http://genome.ucsc.edu/cgi-bin/hgTracks?db=hg17&position=chr9:69274756-69516776&hgt.customText=http://www.neurogenome.org/mcs/tracks/chr9.txt) | [37.82](http://genome.ucsc.edu/cgi-bin/hgTracks?db=hg17&position=chr9:69236932-69274755&hgt.customText=http://www.neurogenome.org/mcs/tracks/chr9.txt) | 76% | -37.22 | 189 | 13.6 | 4% | 3 |
| 3 | APBA2 | 15 | [366.07](http://genome.ucsc.edu/cgi-bin/hgTracks?db=hg17&position=chr15:26833674-27199748&hgt.customText=http://www.neurogenome.org/mcs/tracks/chr15.txt) | [167.47](http://genome.ucsc.edu/cgi-bin/hgTracks?db=hg17&position=chr15:26833674-27001144&hgt.customText=http://www.neurogenome.org/mcs/tracks/chr15.txt) | [196.66](http://genome.ucsc.edu/cgi-bin/hgTracks?db=hg17&position=chr15:27001145-27197807&hgt.customText=http://www.neurogenome.org/mcs/tracks/chr15.txt) | [1.94](http://genome.ucsc.edu/cgi-bin/hgTracks?db=hg17&position=chr15:27197808-27199748&hgt.customText=http://www.neurogenome.org/mcs/tracks/chr15.txt) | 54% | +1.94 | 119 | 8.46 | 2% | 1 |
| 4 | APBA3 | 19 | [11.9](http://genome.ucsc.edu/cgi-bin/hgTracks?db=hg17&position=chr19:3701771-3713671&hgt.customText=http://www.neurogenome.org/mcs/tracks/chr19.txt) | [1](http://genome.ucsc.edu/cgi-bin/hgTracks?db=hg17&position=chr19:3712674-3713671&hgt.customText=http://www.neurogenome.org/mcs/tracks/chr19.txt) | [10.9](http://genome.ucsc.edu/cgi-bin/hgTracks?db=hg17&position=chr19:3701771-3712673&hgt.customText=http://www.neurogenome.org/mcs/tracks/chr19.txt) |  | 92% | -1 | 20 | 1.29 | 11% | 0 |
| 5 | ASPM | 1 | [86.45](http://genome.ucsc.edu/cgi-bin/hgTracks?db=hg17&position=chr1:193768024-193854470&hgt.customText=http://www.neurogenome.org/mcs/tracks/chr1.txt) | [7.25](http://genome.ucsc.edu/cgi-bin/hgTracks?db=hg17&position=chr1:193847225-193854470&hgt.customText=http://www.neurogenome.org/mcs/tracks/chr1.txt) | [62.3](http://genome.ucsc.edu/cgi-bin/hgTracks?db=hg17&position=chr1:193784921-193847224&hgt.customText=http://www.neurogenome.org/mcs/tracks/chr1.txt) | [16.9](http://genome.ucsc.edu/cgi-bin/hgTracks?db=hg17&position=chr1:193768024-193784920&hgt.customText=http://www.neurogenome.org/mcs/tracks/chr1.txt) | 72% | -7.25 | 117 | 4.8 | 6% | 0 |
| 6 | BSN | 3 | [138.39](http://genome.ucsc.edu/cgi-bin/hgTracks?db=hg17&position=chr3:49548049-49686438&hgt.customText=http://www.neurogenome.org/mcs/tracks/chr3.txt) | [18.88](http://genome.ucsc.edu/cgi-bin/hgTracks?db=hg17&position=chr3:49548049-49566925&hgt.customText=http://www.neurogenome.org/mcs/tracks/chr3.txt) | [117.06](http://genome.ucsc.edu/cgi-bin/hgTracks?db=hg17&position=chr3:49566926-49683980&hgt.customText=http://www.neurogenome.org/mcs/tracks/chr3.txt) | [2.46](http://genome.ucsc.edu/cgi-bin/hgTracks?db=hg17&position=chr3:49683981-49686438&hgt.customText=http://www.neurogenome.org/mcs/tracks/chr3.txt) | 85% | +2.46 | 206 | 16.95 | 12% | 4 |
| 7 | BZRAP1 | 17 | [64.24](http://genome.ucsc.edu/cgi-bin/hgTracks?db=hg17&position=chr17:53713296-53777537&hgt.customText=http://www.neurogenome.org/mcs/tracks/chr17.txt) | [16.42](http://genome.ucsc.edu/cgi-bin/hgTracks?db=hg17&position=chr17:53761121-53777537&hgt.customText=http://www.neurogenome.org/mcs/tracks/chr17.txt) | [27.53](http://genome.ucsc.edu/cgi-bin/hgTracks?db=hg17&position=chr17:53733593-53761120&hgt.customText=http://www.neurogenome.org/mcs/tracks/chr17.txt) | [20.3](http://genome.ucsc.edu/cgi-bin/hgTracks?db=hg17&position=chr17:53713296-53733592&hgt.customText=http://www.neurogenome.org/mcs/tracks/chr17.txt) | 43% | -16.42 | 148 | 10.05 | 16% | 0 |
| 8 | CALM1 | 14 | [173.41](http://genome.ucsc.edu/cgi-bin/hgTracks?db=hg17&position=chr14:89903277-90076685&hgt.customText=http://www.neurogenome.org/mcs/tracks/chr14.txt) | [29.85](http://genome.ucsc.edu/cgi-bin/hgTracks?db=hg17&position=chr14:89903277-89933125&hgt.customText=http://www.neurogenome.org/mcs/tracks/chr14.txt) | [11.24](http://genome.ucsc.edu/cgi-bin/hgTracks?db=hg17&position=chr14:89933126-89944363&hgt.customText=http://www.neurogenome.org/mcs/tracks/chr14.txt) | [132.32](http://genome.ucsc.edu/cgi-bin/hgTracks?db=hg17&position=chr14:89944364-90076685&hgt.customText=http://www.neurogenome.org/mcs/tracks/chr14.txt) | 6% | -29.85 | 123 | 9.26 | 5% | 1 |
| 9 | CALM2 | 2 | [214.02](http://genome.ucsc.edu/cgi-bin/hgTracks?db=hg17&position=chr2:47294078-47508100&hgt.customText=http://www.neurogenome.org/mcs/tracks/chr2.txt) | [192.79](http://genome.ucsc.edu/cgi-bin/hgTracks?db=hg17&position=chr2:47315307-47508100&hgt.customText=http://www.neurogenome.org/mcs/tracks/chr2.txt) | [16.35](http://genome.ucsc.edu/cgi-bin/hgTracks?db=hg17&position=chr2:47298956-47315306&hgt.customText=http://www.neurogenome.org/mcs/tracks/chr2.txt) | [4.88](http://genome.ucsc.edu/cgi-bin/hgTracks?db=hg17&position=chr2:47294078-47298955&hgt.customText=http://www.neurogenome.org/mcs/tracks/chr2.txt) | 8% | +4.88 | 107 | 9.53 | 4% | 1 |
| 10 | CALM3 | 19 | [123.97](http://genome.ucsc.edu/cgi-bin/hgTracks?db=hg17&position=chr19:51691596-51815565&hgt.customText=http://www.neurogenome.org/mcs/tracks/chr19.txt) | [104.65](http://genome.ucsc.edu/cgi-bin/hgTracks?db=hg17&position=chr19:51691596-51796246&hgt.customText=http://www.neurogenome.org/mcs/tracks/chr19.txt) | [9.63](http://genome.ucsc.edu/cgi-bin/hgTracks?db=hg17&position=chr19:51796247-51805878&hgt.customText=http://www.neurogenome.org/mcs/tracks/chr19.txt) | [9.69](http://genome.ucsc.edu/cgi-bin/hgTracks?db=hg17&position=chr19:51805879-51815565&hgt.customText=http://www.neurogenome.org/mcs/tracks/chr19.txt) | 8% | +9.69 | 61 | 5.88 | 5% | 3 |
| 11 | CALML3 | 10 | [165.69](http://genome.ucsc.edu/cgi-bin/hgTracks?db=hg17&position=chr10:5555141-5720835&hgt.customText=http://www.neurogenome.org/mcs/tracks/chr10.txt) | [1.78](http://genome.ucsc.edu/cgi-bin/hgTracks?db=hg17&position=chr10:5555141-5556923&hgt.customText=http://www.neurogenome.org/mcs/tracks/chr10.txt) | [1.3](http://genome.ucsc.edu/cgi-bin/hgTracks?db=hg17&position=chr10:5556924-5558225&hgt.customText=http://www.neurogenome.org/mcs/tracks/chr10.txt) | [162.61](http://genome.ucsc.edu/cgi-bin/hgTracks?db=hg17&position=chr10:5558226-5720835&hgt.customText=http://www.neurogenome.org/mcs/tracks/chr10.txt) | 1% | -1.78 | 46 | 3.27 | 2% | 1 |
| 12 | CALML4 | 15 | [18.93](http://genome.ucsc.edu/cgi-bin/hgTracks?db=hg17&position=chr15:66267457-66286385&hgt.customText=http://www.neurogenome.org/mcs/tracks/chr15.txt) | [0.88](http://genome.ucsc.edu/cgi-bin/hgTracks?db=hg17&position=chr15:66285503-66286385&hgt.customText=http://www.neurogenome.org/mcs/tracks/chr15.txt) | [15.02](http://genome.ucsc.edu/cgi-bin/hgTracks?db=hg17&position=chr15:66270480-66285502&hgt.customText=http://www.neurogenome.org/mcs/tracks/chr15.txt) | [3.02](http://genome.ucsc.edu/cgi-bin/hgTracks?db=hg17&position=chr15:66267457-66270479&hgt.customText=http://www.neurogenome.org/mcs/tracks/chr15.txt) | 79% | -0.88 | 28 | 2.1 | 11% | 0 |
| 13 | CALML5 | 10 | [55.8](http://genome.ucsc.edu/cgi-bin/hgTracks?db=hg17&position=chr10:5490402-5546206&hgt.customText=http://www.neurogenome.org/mcs/tracks/chr10.txt) | [14.7](http://genome.ucsc.edu/cgi-bin/hgTracks?db=hg17&position=chr10:5531511-5546206&hgt.customText=http://www.neurogenome.org/mcs/tracks/chr10.txt) | [0.85](http://genome.ucsc.edu/cgi-bin/hgTracks?db=hg17&position=chr10:5530661-5531510&hgt.customText=http://www.neurogenome.org/mcs/tracks/chr10.txt) | [40.26](http://genome.ucsc.edu/cgi-bin/hgTracks?db=hg17&position=chr10:5490402-5530660&hgt.customText=http://www.neurogenome.org/mcs/tracks/chr10.txt) | 2% | -14.7 | 10 | 0.44 | 1% | 0 |
| 14 | CALML6 | 1 | [26.53](http://genome.ucsc.edu/cgi-bin/hgTracks?db=hg17&position=chr1:1854658-1881190&hgt.customText=http://www.neurogenome.org/mcs/tracks/chr1.txt) | [23.77](http://genome.ucsc.edu/cgi-bin/hgTracks?db=hg17&position=chr1:1854658-1878427&hgt.customText=http://www.neurogenome.org/mcs/tracks/chr1.txt) | [2.47](http://genome.ucsc.edu/cgi-bin/hgTracks?db=hg17&position=chr1:1878428-1880895&hgt.customText=http://www.neurogenome.org/mcs/tracks/chr1.txt) | [0.29](http://genome.ucsc.edu/cgi-bin/hgTracks?db=hg17&position=chr1:1880896-1881190&hgt.customText=http://www.neurogenome.org/mcs/tracks/chr1.txt) | 9% | +0.29 | 27 | 0.92 | 3% | 0 |
| 15 | CAMK1 | 3 | [22.62](http://genome.ucsc.edu/cgi-bin/hgTracks?db=hg17&position=chr3:9774032-9796653&hgt.customText=http://www.neurogenome.org/mcs/tracks/chr3.txt) | [9.99](http://genome.ucsc.edu/cgi-bin/hgTracks?db=hg17&position=chr3:9786662-9796653&hgt.customText=http://www.neurogenome.org/mcs/tracks/chr3.txt) | [12.63](http://genome.ucsc.edu/cgi-bin/hgTracks?db=hg17&position=chr3:9774032-9786661&hgt.customText=http://www.neurogenome.org/mcs/tracks/chr3.txt) |  | 56% | -9.99 | 34 | 2.58 | 11% | 0 |
| 16 | CAMK1D | 10 | [646.04](http://genome.ucsc.edu/cgi-bin/hgTracks?db=hg17&position=chr10:12332594-12978630&hgt.customText=http://www.neurogenome.org/mcs/tracks/chr10.txt) | [99](http://genome.ucsc.edu/cgi-bin/hgTracks?db=hg17&position=chr10:12332594-12431588&hgt.customText=http://www.neurogenome.org/mcs/tracks/chr10.txt) | [480.15](http://genome.ucsc.edu/cgi-bin/hgTracks?db=hg17&position=chr10:12431589-12911737&hgt.customText=http://www.neurogenome.org/mcs/tracks/chr10.txt) | [66.89](http://genome.ucsc.edu/cgi-bin/hgTracks?db=hg17&position=chr10:12911738-12978630&hgt.customText=http://www.neurogenome.org/mcs/tracks/chr10.txt) | 74% | +66.89 | 208 | 13.86 | 2% | 0 |
| 17 | CAMK1G | 1 | [1371.1](http://genome.ucsc.edu/cgi-bin/hgTracks?db=hg17&position=chr1:204805512-206176613&hgt.customText=http://www.neurogenome.org/mcs/tracks/chr1.txt) | [1339.93](http://genome.ucsc.edu/cgi-bin/hgTracks?db=hg17&position=chr1:204805512-206145439&hgt.customText=http://www.neurogenome.org/mcs/tracks/chr1.txt) | [30.24](http://genome.ucsc.edu/cgi-bin/hgTracks?db=hg17&position=chr1:206145440-206175678&hgt.customText=http://www.neurogenome.org/mcs/tracks/chr1.txt) | [0.94](http://genome.ucsc.edu/cgi-bin/hgTracks?db=hg17&position=chr1:206175679-206176613&hgt.customText=http://www.neurogenome.org/mcs/tracks/chr1.txt) | 2% | +0.94 | 1146 | 115.32 | 8% | 37 |
| 18 | CAMK2A | 5 | [92.68](http://genome.ucsc.edu/cgi-bin/hgTracks?db=hg17&position=chr5:149563424-149656100&hgt.customText=http://www.neurogenome.org/mcs/tracks/chr5.txt) | [6.57](http://genome.ucsc.edu/cgi-bin/hgTracks?db=hg17&position=chr5:149649530-149656100&hgt.customText=http://www.neurogenome.org/mcs/tracks/chr5.txt) | [86.11](http://genome.ucsc.edu/cgi-bin/hgTracks?db=hg17&position=chr5:149563424-149649529&hgt.customText=http://www.neurogenome.org/mcs/tracks/chr5.txt) |  | 93% | -6.57 | 121 | 9.59 | 10% | 0 |
| 19 | CAMK2B | 7 | [167.99](http://genome.ucsc.edu/cgi-bin/hgTracks?db=hg17&position=chr7:44027133-44195122&hgt.customText=http://www.neurogenome.org/mcs/tracks/chr7.txt) | [56.66](http://genome.ucsc.edu/cgi-bin/hgTracks?db=hg17&position=chr7:44138465-44195122&hgt.customText=http://www.neurogenome.org/mcs/tracks/chr7.txt) | [106.33](http://genome.ucsc.edu/cgi-bin/hgTracks?db=hg17&position=chr7:44032137-44138464&hgt.customText=http://www.neurogenome.org/mcs/tracks/chr7.txt) | [5](http://genome.ucsc.edu/cgi-bin/hgTracks?db=hg17&position=chr7:44027133-44032136&hgt.customText=http://www.neurogenome.org/mcs/tracks/chr7.txt) | 63% | +5 | 95 | 7.84 | 5% | 1 |
| 20 | CAMK2D | 4 | [516.55](http://genome.ucsc.edu/cgi-bin/hgTracks?db=hg17&position=chr4:114662496-115179042&hgt.customText=http://www.neurogenome.org/mcs/tracks/chr4.txt) | [138.71](http://genome.ucsc.edu/cgi-bin/hgTracks?db=hg17&position=chr4:115040333-115179042&hgt.customText=http://www.neurogenome.org/mcs/tracks/chr4.txt) | [309.16](http://genome.ucsc.edu/cgi-bin/hgTracks?db=hg17&position=chr4:114731177-115040332&hgt.customText=http://www.neurogenome.org/mcs/tracks/chr4.txt) | [68.68](http://genome.ucsc.edu/cgi-bin/hgTracks?db=hg17&position=chr4:114662496-114731176&hgt.customText=http://www.neurogenome.org/mcs/tracks/chr4.txt) | 60% | +68.68 | 246 | 17.85 | 3% | 2 |
| 21 | CAMK2G | 10 | [98.19](http://genome.ucsc.edu/cgi-bin/hgTracks?db=hg17&position=chr10:75241548-75339739&hgt.customText=http://www.neurogenome.org/mcs/tracks/chr10.txt) | [35.39](http://genome.ucsc.edu/cgi-bin/hgTracks?db=hg17&position=chr10:75304350-75339739&hgt.customText=http://www.neurogenome.org/mcs/tracks/chr10.txt) | [62.09](http://genome.ucsc.edu/cgi-bin/hgTracks?db=hg17&position=chr10:75242265-75304349&hgt.customText=http://www.neurogenome.org/mcs/tracks/chr10.txt) | [0.72](http://genome.ucsc.edu/cgi-bin/hgTracks?db=hg17&position=chr10:75241548-75242264&hgt.customText=http://www.neurogenome.org/mcs/tracks/chr10.txt) | 63% | +0.72 | 158 | 16.9 | 17% | 6 |
| 22 | CAMK2N1 | 1 | [145.2](http://genome.ucsc.edu/cgi-bin/hgTracks?db=hg17&position=chr1:20426051-20571248&hgt.customText=http://www.neurogenome.org/mcs/tracks/chr1.txt) | [13.67](http://genome.ucsc.edu/cgi-bin/hgTracks?db=hg17&position=chr1:20557579-20571248&hgt.customText=http://www.neurogenome.org/mcs/tracks/chr1.txt) | [3.39](http://genome.ucsc.edu/cgi-bin/hgTracks?db=hg17&position=chr1:20554191-20557578&hgt.customText=http://www.neurogenome.org/mcs/tracks/chr1.txt) | [128.14](http://genome.ucsc.edu/cgi-bin/hgTracks?db=hg17&position=chr1:20426051-20554190&hgt.customText=http://www.neurogenome.org/mcs/tracks/chr1.txt) | 2% | -13.67 | 135 | 9.66 | 7% | 0 |
| 23 | CAMK2N2 | 3 | [2.25](http://genome.ucsc.edu/cgi-bin/hgTracks?db=hg17&position=chr3:185459705-185461953&hgt.customText=http://www.neurogenome.org/mcs/tracks/chr3.txt) |  | [2.25](http://genome.ucsc.edu/cgi-bin/hgTracks?db=hg17&position=chr3:185459705-185461953&hgt.customText=http://www.neurogenome.org/mcs/tracks/chr3.txt) |  | 100% | -0 | 3 | 0.4 | 18% | 0 |
| 24 | CAMK4 | 5 | [367.82](http://genome.ucsc.edu/cgi-bin/hgTracks?db=hg17&position=chr5:110494100-110861920&hgt.customText=http://www.neurogenome.org/mcs/tracks/chr5.txt) | [93.88](http://genome.ucsc.edu/cgi-bin/hgTracks?db=hg17&position=chr5:110494100-110587980&hgt.customText=http://www.neurogenome.org/mcs/tracks/chr5.txt) | [260.67](http://genome.ucsc.edu/cgi-bin/hgTracks?db=hg17&position=chr5:110587981-110848645&hgt.customText=http://www.neurogenome.org/mcs/tracks/chr5.txt) | [13.28](http://genome.ucsc.edu/cgi-bin/hgTracks?db=hg17&position=chr5:110848646-110861920&hgt.customText=http://www.neurogenome.org/mcs/tracks/chr5.txt) | 71% | +13.28 | 158 | 11.07 | 3% | 1 |
| 25 | CASK | X | [809.42](http://genome.ucsc.edu/cgi-bin/hgTracks?db=hg17&position=chrX:41091218-41900635&hgt.customText=http://www.neurogenome.org/mcs/tracks/chrX.txt) | [362.11](http://genome.ucsc.edu/cgi-bin/hgTracks?db=hg17&position=chrX:41538523-41900635&hgt.customText=http://www.neurogenome.org/mcs/tracks/chrX.txt) | [403.81](http://genome.ucsc.edu/cgi-bin/hgTracks?db=hg17&position=chrX:41134718-41538522&hgt.customText=http://www.neurogenome.org/mcs/tracks/chrX.txt) | [43.5](http://genome.ucsc.edu/cgi-bin/hgTracks?db=hg17&position=chrX:41091218-41134717&hgt.customText=http://www.neurogenome.org/mcs/tracks/chrX.txt) | 50% | +43.5 | 729 | 61.3 | 8% | 16 |
| 26 | CAST | 5 | [341.43](http://genome.ucsc.edu/cgi-bin/hgTracks?db=hg17&position=chr5:95794709-96136139&hgt.customText=http://www.neurogenome.org/mcs/tracks/chr5.txt) | [228.82](http://genome.ucsc.edu/cgi-bin/hgTracks?db=hg17&position=chr5:95794709-96023532&hgt.customText=http://www.neurogenome.org/mcs/tracks/chr5.txt) | [112.61](http://genome.ucsc.edu/cgi-bin/hgTracks?db=hg17&position=chr5:96023533-96136139&hgt.customText=http://www.neurogenome.org/mcs/tracks/chr5.txt) |  | 33% | -228.82 | 120 | 8.39 | 2% | 1 |
| 27 | CAST1 | 3 | [1069.86](http://genome.ucsc.edu/cgi-bin/hgTracks?db=hg17&position=chr3:55496372-56566230&hgt.customText=http://www.neurogenome.org/mcs/tracks/chr3.txt) | [88.8](http://genome.ucsc.edu/cgi-bin/hgTracks?db=hg17&position=chr3:56477432-56566230&hgt.customText=http://www.neurogenome.org/mcs/tracks/chr3.txt) | [960.06](http://genome.ucsc.edu/cgi-bin/hgTracks?db=hg17&position=chr3:55517376-56477431&hgt.customText=http://www.neurogenome.org/mcs/tracks/chr3.txt) | [21](http://genome.ucsc.edu/cgi-bin/hgTracks?db=hg17&position=chr3:55496372-55517375&hgt.customText=http://www.neurogenome.org/mcs/tracks/chr3.txt) | 90% | +21 | 996 | 101.14 | 9% | 33 |
| 28 | DMXL2 | 15 | [273.35](http://genome.ucsc.edu/cgi-bin/hgTracks?db=hg17&position=chr15:49487494-49760841&hgt.customText=http://www.neurogenome.org/mcs/tracks/chr15.txt) | [58.6](http://genome.ucsc.edu/cgi-bin/hgTracks?db=hg17&position=chr15:49702242-49760841&hgt.customText=http://www.neurogenome.org/mcs/tracks/chr15.txt) | [174.06](http://genome.ucsc.edu/cgi-bin/hgTracks?db=hg17&position=chr15:49528181-49702241&hgt.customText=http://www.neurogenome.org/mcs/tracks/chr15.txt) | [40.69](http://genome.ucsc.edu/cgi-bin/hgTracks?db=hg17&position=chr15:49487494-49528180&hgt.customText=http://www.neurogenome.org/mcs/tracks/chr15.txt) | 64% | +40.69 | 158 | 12.94 | 5% | 2 |
| 29 | DNM1 | 9 | [52.45](http://genome.ucsc.edu/cgi-bin/hgTracks?db=hg17&position=chr9:128045212-128097661&hgt.customText=http://www.neurogenome.org/mcs/tracks/chr9.txt) |  | [51.87](http://genome.ucsc.edu/cgi-bin/hgTracks?db=hg17&position=chr9:128045212-128097081&hgt.customText=http://www.neurogenome.org/mcs/tracks/chr9.txt) | [0.58](http://genome.ucsc.edu/cgi-bin/hgTracks?db=hg17&position=chr9:128097082-128097661&hgt.customText=http://www.neurogenome.org/mcs/tracks/chr9.txt) | 99% | +0.58 | 108 | 9.38 | 18% | 2 |
| 30 | EPIM | 12 | [155.74](http://genome.ucsc.edu/cgi-bin/hgTracks?db=hg17&position=chr12:129725707-129881447&hgt.customText=http://www.neurogenome.org/mcs/tracks/chr12.txt) | [32.76](http://genome.ucsc.edu/cgi-bin/hgTracks?db=hg17&position=chr12:129848692-129881447&hgt.customText=http://www.neurogenome.org/mcs/tracks/chr12.txt) | [49.66](http://genome.ucsc.edu/cgi-bin/hgTracks?db=hg17&position=chr12:129799028-129848691&hgt.customText=http://www.neurogenome.org/mcs/tracks/chr12.txt) | [73.32](http://genome.ucsc.edu/cgi-bin/hgTracks?db=hg17&position=chr12:129725707-129799027&hgt.customText=http://www.neurogenome.org/mcs/tracks/chr12.txt) | 32% | -32.76 | 30 | 1.96 | 1% | 0 |
| 31 | EXOC1 | 4 | [312.57](http://genome.ucsc.edu/cgi-bin/hgTracks?db=hg17&position=chr4:56343394-56655964&hgt.customText=http://www.neurogenome.org/mcs/tracks/chr4.txt) | [217.35](http://genome.ucsc.edu/cgi-bin/hgTracks?db=hg17&position=chr4:56343394-56560743&hgt.customText=http://www.neurogenome.org/mcs/tracks/chr4.txt) | [51.43](http://genome.ucsc.edu/cgi-bin/hgTracks?db=hg17&position=chr4:56560744-56612172&hgt.customText=http://www.neurogenome.org/mcs/tracks/chr4.txt) | [43.79](http://genome.ucsc.edu/cgi-bin/hgTracks?db=hg17&position=chr4:56612173-56655964&hgt.customText=http://www.neurogenome.org/mcs/tracks/chr4.txt) | 16% | +43.79 | 142 | 10.73 | 3% | 0 |
| 32 | EXOC2 | 6 | [281.67](http://genome.ucsc.edu/cgi-bin/hgTracks?db=hg17&position=chr6:356443-638109&hgt.customText=http://www.neurogenome.org/mcs/tracks/chr6.txt) |  | [207.97](http://genome.ucsc.edu/cgi-bin/hgTracks?db=hg17&position=chr6:430139-638109&hgt.customText=http://www.neurogenome.org/mcs/tracks/chr6.txt) | [73.7](http://genome.ucsc.edu/cgi-bin/hgTracks?db=hg17&position=chr6:356443-430138&hgt.customText=http://www.neurogenome.org/mcs/tracks/chr6.txt) | 74% | +73.7 | 129 | 9.32 | 3% | 1 |
| 33 | EXOC3 | 5 | [28.79](http://genome.ucsc.edu/cgi-bin/hgTracks?db=hg17&position=chr5:496259-525052&hgt.customText=http://www.neurogenome.org/mcs/tracks/chr5.txt) | [0.07](http://genome.ucsc.edu/cgi-bin/hgTracks?db=hg17&position=chr5:496259-496333&hgt.customText=http://www.neurogenome.org/mcs/tracks/chr5.txt) | [28.72](http://genome.ucsc.edu/cgi-bin/hgTracks?db=hg17&position=chr5:496334-525052&hgt.customText=http://www.neurogenome.org/mcs/tracks/chr5.txt) |  | 100% | -0.07 | 28 | 2.69 | 9% | 1 |
| 34 | EXOC4 | 7 | [1045.25](http://genome.ucsc.edu/cgi-bin/hgTracks?db=hg17&position=chr7:132224106-133269359&hgt.customText=http://www.neurogenome.org/mcs/tracks/chr7.txt) | [170.97](http://genome.ucsc.edu/cgi-bin/hgTracks?db=hg17&position=chr7:132224106-132395077&hgt.customText=http://www.neurogenome.org/mcs/tracks/chr7.txt) | [812.69](http://genome.ucsc.edu/cgi-bin/hgTracks?db=hg17&position=chr7:132395078-133207767&hgt.customText=http://www.neurogenome.org/mcs/tracks/chr7.txt) | [61.59](http://genome.ucsc.edu/cgi-bin/hgTracks?db=hg17&position=chr7:133207768-133269359&hgt.customText=http://www.neurogenome.org/mcs/tracks/chr7.txt) | 78% | +61.59 | 810 | 78.72 | 8% | 28 |
| 35 | EXOC5 | 14 | [458.44](http://genome.ucsc.edu/cgi-bin/hgTracks?db=hg17&position=chr14:56346941-56805379&hgt.customText=http://www.neurogenome.org/mcs/tracks/chr14.txt) | [0](http://genome.ucsc.edu/cgi-bin/hgTracks?db=hg17&position=chr14:56805377-56805379&hgt.customText=http://www.neurogenome.org/mcs/tracks/chr14.txt) | [66.43](http://genome.ucsc.edu/cgi-bin/hgTracks?db=hg17&position=chr14:56738949-56805376&hgt.customText=http://www.neurogenome.org/mcs/tracks/chr14.txt) | [392.01](http://genome.ucsc.edu/cgi-bin/hgTracks?db=hg17&position=chr14:56346941-56738948&hgt.customText=http://www.neurogenome.org/mcs/tracks/chr14.txt) | 14% | -0 | 364 | 47.23 | 10% | 35 |
| 36 | EXOC6 | 10 | [365.62](http://genome.ucsc.edu/cgi-bin/hgTracks?db=hg17&position=chr10:94445387-94811010&hgt.customText=http://www.neurogenome.org/mcs/tracks/chr10.txt) | [139.06](http://genome.ucsc.edu/cgi-bin/hgTracks?db=hg17&position=chr10:94445387-94584449&hgt.customText=http://www.neurogenome.org/mcs/tracks/chr10.txt) | [224.79](http://genome.ucsc.edu/cgi-bin/hgTracks?db=hg17&position=chr10:94584450-94809240&hgt.customText=http://www.neurogenome.org/mcs/tracks/chr10.txt) | [1.77](http://genome.ucsc.edu/cgi-bin/hgTracks?db=hg17&position=chr10:94809241-94811010&hgt.customText=http://www.neurogenome.org/mcs/tracks/chr10.txt) | 61% | +1.77 | 218 | 18.55 | 5% | 7 |
| 37 | EXOC7 | 17 | [55.33](http://genome.ucsc.edu/cgi-bin/hgTracks?db=hg17&position=chr17:71588682-71644011&hgt.customText=http://www.neurogenome.org/mcs/tracks/chr17.txt) | [32.55](http://genome.ucsc.edu/cgi-bin/hgTracks?db=hg17&position=chr17:71611464-71644011&hgt.customText=http://www.neurogenome.org/mcs/tracks/chr17.txt) | [22.78](http://genome.ucsc.edu/cgi-bin/hgTracks?db=hg17&position=chr17:71588682-71611463&hgt.customText=http://www.neurogenome.org/mcs/tracks/chr17.txt) |  | 41% | -32.55 | 55 | 3.73 | 7% | 0 |
| 38 | EXOC8 | 1 | [60.04](http://genome.ucsc.edu/cgi-bin/hgTracks?db=hg17&position=chr1:227720271-227780313&hgt.customText=http://www.neurogenome.org/mcs/tracks/chr1.txt) |  | [5.1](http://genome.ucsc.edu/cgi-bin/hgTracks?db=hg17&position=chr1:227775218-227780313&hgt.customText=http://www.neurogenome.org/mcs/tracks/chr1.txt) | [54.95](http://genome.ucsc.edu/cgi-bin/hgTracks?db=hg17&position=chr1:227720271-227775217&hgt.customText=http://www.neurogenome.org/mcs/tracks/chr1.txt) | 8% | +54.95 | 11 | 2.29 | 4% | 2 |
| 39 | GDI1 | X | [52.56](http://genome.ucsc.edu/cgi-bin/hgTracks?db=hg17&position=chrX:153183642-153236205&hgt.customText=http://www.neurogenome.org/mcs/tracks/chrX.txt) | [50.52](http://genome.ucsc.edu/cgi-bin/hgTracks?db=hg17&position=chrX:153183642-153234158&hgt.customText=http://www.neurogenome.org/mcs/tracks/chrX.txt) | [1.36](http://genome.ucsc.edu/cgi-bin/hgTracks?db=hg17&position=chrX:153234159-153235516&hgt.customText=http://www.neurogenome.org/mcs/tracks/chrX.txt) | [0.69](http://genome.ucsc.edu/cgi-bin/hgTracks?db=hg17&position=chrX:153235517-153236205&hgt.customText=http://www.neurogenome.org/mcs/tracks/chrX.txt) | 3% | +0.69 | 8 | 1.01 | 2% | 0 |
| 40 | GDI2 | 10 | [97.2](http://genome.ucsc.edu/cgi-bin/hgTracks?db=hg17&position=chr10:5845702-5942897&hgt.customText=http://www.neurogenome.org/mcs/tracks/chr10.txt) | [47.52](http://genome.ucsc.edu/cgi-bin/hgTracks?db=hg17&position=chr10:5895380-5942897&hgt.customText=http://www.neurogenome.org/mcs/tracks/chr10.txt) | [48.19](http://genome.ucsc.edu/cgi-bin/hgTracks?db=hg17&position=chr10:5847192-5895379&hgt.customText=http://www.neurogenome.org/mcs/tracks/chr10.txt) | [1.49](http://genome.ucsc.edu/cgi-bin/hgTracks?db=hg17&position=chr10:5845702-5847191&hgt.customText=http://www.neurogenome.org/mcs/tracks/chr10.txt) | 50% | +1.49 | 35 | 3.18 | 3% | 0 |
| 41 | GZMB | 14 | [199.96](http://genome.ucsc.edu/cgi-bin/hgTracks?db=hg17&position=chr14:24148746-24348707&hgt.customText=http://www.neurogenome.org/mcs/tracks/chr14.txt) | [175.39](http://genome.ucsc.edu/cgi-bin/hgTracks?db=hg17&position=chr14:24173314-24348707&hgt.customText=http://www.neurogenome.org/mcs/tracks/chr14.txt) | [3.31](http://genome.ucsc.edu/cgi-bin/hgTracks?db=hg17&position=chr14:24170004-24173313&hgt.customText=http://www.neurogenome.org/mcs/tracks/chr14.txt) | [21.26](http://genome.ucsc.edu/cgi-bin/hgTracks?db=hg17&position=chr14:24148746-24170003&hgt.customText=http://www.neurogenome.org/mcs/tracks/chr14.txt) | 2% | +21.26 | 57 | 3.97 | 2% | 1 |
| 42 | NAPA | 19 | [36.47](http://genome.ucsc.edu/cgi-bin/hgTracks?db=hg17&position=chr19:52679289-52715758&hgt.customText=http://www.neurogenome.org/mcs/tracks/chr19.txt) | [5.45](http://genome.ucsc.edu/cgi-bin/hgTracks?db=hg17&position=chr19:52710310-52715758&hgt.customText=http://www.neurogenome.org/mcs/tracks/chr19.txt) | [27.61](http://genome.ucsc.edu/cgi-bin/hgTracks?db=hg17&position=chr19:52682703-52710309&hgt.customText=http://www.neurogenome.org/mcs/tracks/chr19.txt) | [3.41](http://genome.ucsc.edu/cgi-bin/hgTracks?db=hg17&position=chr19:52679289-52682702&hgt.customText=http://www.neurogenome.org/mcs/tracks/chr19.txt) | 76% | +3.41 | 31 | 2.06 | 6% | 0 |
| 43 | NAPB | 20 | [53.97](http://genome.ucsc.edu/cgi-bin/hgTracks?db=hg17&position=chr20:23301683-23355650&hgt.customText=http://www.neurogenome.org/mcs/tracks/chr20.txt) | [5.57](http://genome.ucsc.edu/cgi-bin/hgTracks?db=hg17&position=chr20:23350084-23355650&hgt.customText=http://www.neurogenome.org/mcs/tracks/chr20.txt) | [46.92](http://genome.ucsc.edu/cgi-bin/hgTracks?db=hg17&position=chr20:23303164-23350083&hgt.customText=http://www.neurogenome.org/mcs/tracks/chr20.txt) | [1.48](http://genome.ucsc.edu/cgi-bin/hgTracks?db=hg17&position=chr20:23301683-23303163&hgt.customText=http://www.neurogenome.org/mcs/tracks/chr20.txt) | 87% | +1.48 | 30 | 2.26 | 4% | 0 |
| 44 | NAPG | 18 | [182.16](http://genome.ucsc.edu/cgi-bin/hgTracks?db=hg17&position=chr18:10478698-10660859&hgt.customText=http://www.neurogenome.org/mcs/tracks/chr18.txt) | [37.33](http://genome.ucsc.edu/cgi-bin/hgTracks?db=hg17&position=chr18:10478698-10516030&hgt.customText=http://www.neurogenome.org/mcs/tracks/chr18.txt) | [24.34](http://genome.ucsc.edu/cgi-bin/hgTracks?db=hg17&position=chr18:10516031-10540371&hgt.customText=http://www.neurogenome.org/mcs/tracks/chr18.txt) | [120.49](http://genome.ucsc.edu/cgi-bin/hgTracks?db=hg17&position=chr18:10540372-10660859&hgt.customText=http://www.neurogenome.org/mcs/tracks/chr18.txt) | 13% | -37.33 | 44 | 4.68 | 3% | 2 |
| 45 | NBEA | 13 | [1804.78](http://genome.ucsc.edu/cgi-bin/hgTracks?db=hg17&position=chr13:33438695-35243477&hgt.customText=http://www.neurogenome.org/mcs/tracks/chr13.txt) | [975.76](http://genome.ucsc.edu/cgi-bin/hgTracks?db=hg17&position=chr13:33438695-34414455&hgt.customText=http://www.neurogenome.org/mcs/tracks/chr13.txt) | [730.42](http://genome.ucsc.edu/cgi-bin/hgTracks?db=hg17&position=chr13:34414456-35144872&hgt.customText=http://www.neurogenome.org/mcs/tracks/chr13.txt) | [98.61](http://genome.ucsc.edu/cgi-bin/hgTracks?db=hg17&position=chr13:35144873-35243477&hgt.customText=http://www.neurogenome.org/mcs/tracks/chr13.txt) | 40% | +98.61 | 889 | 82.61 | 5% | 20 |
| 46 | NCAM1 | 11 | [1053.13](http://genome.ucsc.edu/cgi-bin/hgTracks?db=hg17&position=chr11:111637328-112690460&hgt.customText=http://www.neurogenome.org/mcs/tracks/chr11.txt) | [700.04](http://genome.ucsc.edu/cgi-bin/hgTracks?db=hg17&position=chr11:111637328-112337367&hgt.customText=http://www.neurogenome.org/mcs/tracks/chr11.txt) | [316.41](http://genome.ucsc.edu/cgi-bin/hgTracks?db=hg17&position=chr11:112337368-112653781&hgt.customText=http://www.neurogenome.org/mcs/tracks/chr11.txt) | [36.68](http://genome.ucsc.edu/cgi-bin/hgTracks?db=hg17&position=chr11:112653782-112690460&hgt.customText=http://www.neurogenome.org/mcs/tracks/chr11.txt) | 30% | +36.68 | 906 | 83.45 | 8% | 19 |
| 47 | NLGN1 | 3 | [1718.1](http://genome.ucsc.edu/cgi-bin/hgTracks?db=hg17&position=chr3:174341709-176059812&hgt.customText=http://www.neurogenome.org/mcs/tracks/chr3.txt) | [257.24](http://genome.ucsc.edu/cgi-bin/hgTracks?db=hg17&position=chr3:174341709-174598945&hgt.customText=http://www.neurogenome.org/mcs/tracks/chr3.txt) | [884.87](http://genome.ucsc.edu/cgi-bin/hgTracks?db=hg17&position=chr3:174598946-175483818&hgt.customText=http://www.neurogenome.org/mcs/tracks/chr3.txt) | [575.99](http://genome.ucsc.edu/cgi-bin/hgTracks?db=hg17&position=chr3:175483819-176059812&hgt.customText=http://www.neurogenome.org/mcs/tracks/chr3.txt) | 52% | -257.24 | 854 | 78.99 | 5% | 25 |
| 48 | NLGN2 | 17 | [16.22](http://genome.ucsc.edu/cgi-bin/hgTracks?db=hg17&position=chr17:7248143-7264366&hgt.customText=http://www.neurogenome.org/mcs/tracks/chr17.txt) | [4.08](http://genome.ucsc.edu/cgi-bin/hgTracks?db=hg17&position=chr17:7248143-7252225&hgt.customText=http://www.neurogenome.org/mcs/tracks/chr17.txt) | [11.68](http://genome.ucsc.edu/cgi-bin/hgTracks?db=hg17&position=chr17:7252226-7263903&hgt.customText=http://www.neurogenome.org/mcs/tracks/chr17.txt) | [0.46](http://genome.ucsc.edu/cgi-bin/hgTracks?db=hg17&position=chr17:7263904-7264366&hgt.customText=http://www.neurogenome.org/mcs/tracks/chr17.txt) | 72% | +0.46 | 56 | 8.25 | 51% | 5 |
| 49 | NLGN3 | X | [72.79](http://genome.ucsc.edu/cgi-bin/hgTracks?db=hg17&position=chrX:70145319-70218109&hgt.customText=http://www.neurogenome.org/mcs/tracks/chrX.txt) | [2.41](http://genome.ucsc.edu/cgi-bin/hgTracks?db=hg17&position=chrX:70145319-70147731&hgt.customText=http://www.neurogenome.org/mcs/tracks/chrX.txt) | [26.34](http://genome.ucsc.edu/cgi-bin/hgTracks?db=hg17&position=chrX:70147732-70174070&hgt.customText=http://www.neurogenome.org/mcs/tracks/chrX.txt) | [44.04](http://genome.ucsc.edu/cgi-bin/hgTracks?db=hg17&position=chrX:70174071-70218109&hgt.customText=http://www.neurogenome.org/mcs/tracks/chrX.txt) | 36% | -2.41 | 116 | 12.77 | 18% | 8 |
| 50 | NLGN4X | X | [730.58](http://genome.ucsc.edu/cgi-bin/hgTracks?db=hg17&position=chrX:5275859-6006442&hgt.customText=http://www.neurogenome.org/mcs/tracks/chrX.txt) |  | [338.62](http://genome.ucsc.edu/cgi-bin/hgTracks?db=hg17&position=chrX:5667821-6006442&hgt.customText=http://www.neurogenome.org/mcs/tracks/chrX.txt) | [391.96](http://genome.ucsc.edu/cgi-bin/hgTracks?db=hg17&position=chrX:5275859-5667820&hgt.customText=http://www.neurogenome.org/mcs/tracks/chrX.txt) | 46% | +391.96 | 56 | 7.77 | 1% | 4 |
| 51 | NLGN4Y | Y | [1829.79](http://genome.ucsc.edu/cgi-bin/hgTracks?db=hg17&position=chrY:14606970-16436756&hgt.customText=http://www.neurogenome.org/mcs/tracks/chrY.txt) | [465.79](http://genome.ucsc.edu/cgi-bin/hgTracks?db=hg17&position=chrY:14606970-15072762&hgt.customText=http://www.neurogenome.org/mcs/tracks/chrY.txt) | [321.22](http://genome.ucsc.edu/cgi-bin/hgTracks?db=hg17&position=chrY:15072763-15393979&hgt.customText=http://www.neurogenome.org/mcs/tracks/chrY.txt) | [1042.78](http://genome.ucsc.edu/cgi-bin/hgTracks?db=hg17&position=chrY:15393980-16436756&hgt.customText=http://www.neurogenome.org/mcs/tracks/chrY.txt) | 18% | -465.79 | 96 | 7.43 | 0% | 1 |
| 52 | NRXN1 | 2 | [4515.49](http://genome.ucsc.edu/cgi-bin/hgTracks?db=hg17&position=chr2:49293282-53808768&hgt.customText=http://www.neurogenome.org/mcs/tracks/chr2.txt) | [2641.51](http://genome.ucsc.edu/cgi-bin/hgTracks?db=hg17&position=chr2:51167255-53808768&hgt.customText=http://www.neurogenome.org/mcs/tracks/chr2.txt) | [1108.12](http://genome.ucsc.edu/cgi-bin/hgTracks?db=hg17&position=chr2:50059139-51167254&hgt.customText=http://www.neurogenome.org/mcs/tracks/chr2.txt) | [765.86](http://genome.ucsc.edu/cgi-bin/hgTracks?db=hg17&position=chr2:49293282-50059138&hgt.customText=http://www.neurogenome.org/mcs/tracks/chr2.txt) | 25% | +765.86 | 1664 | 156.16 | 3% | 41 |
| 53 | NRXN2 | 11 | [124.56](http://genome.ucsc.edu/cgi-bin/hgTracks?db=hg17&position=chr11:64126397-64250958&hgt.customText=http://www.neurogenome.org/mcs/tracks/chr11.txt) | [3.72](http://genome.ucsc.edu/cgi-bin/hgTracks?db=hg17&position=chr11:64247237-64250958&hgt.customText=http://www.neurogenome.org/mcs/tracks/chr11.txt) | [117.02](http://genome.ucsc.edu/cgi-bin/hgTracks?db=hg17&position=chr11:64130222-64247236&hgt.customText=http://www.neurogenome.org/mcs/tracks/chr11.txt) | [3.83](http://genome.ucsc.edu/cgi-bin/hgTracks?db=hg17&position=chr11:64126397-64130221&hgt.customText=http://www.neurogenome.org/mcs/tracks/chr11.txt) | 94% | -3.72 | 257 | 23.71 | 19% | 8 |
| 54 | NRXN3 | 14 | [2263.57](http://genome.ucsc.edu/cgi-bin/hgTracks?db=hg17&position=chr14:77470049-79733622&hgt.customText=http://www.neurogenome.org/mcs/tracks/chr14.txt) | [236.63](http://genome.ucsc.edu/cgi-bin/hgTracks?db=hg17&position=chr14:77470049-77706680&hgt.customText=http://www.neurogenome.org/mcs/tracks/chr14.txt) | [1693.83](http://genome.ucsc.edu/cgi-bin/hgTracks?db=hg17&position=chr14:77706681-79400511&hgt.customText=http://www.neurogenome.org/mcs/tracks/chr14.txt) | [333.11](http://genome.ucsc.edu/cgi-bin/hgTracks?db=hg17&position=chr14:79400512-79733622&hgt.customText=http://www.neurogenome.org/mcs/tracks/chr14.txt) | 75% | -236.63 | 1983 | 171.7 | 8% | 34 |
| 55 | NSF | 17 | [395.13](http://genome.ucsc.edu/cgi-bin/hgTracks?db=hg17&position=chr17:41794866-42189993&hgt.customText=http://www.neurogenome.org/mcs/tracks/chr17.txt) | [11.08](http://genome.ucsc.edu/cgi-bin/hgTracks?db=hg17&position=chr17:41794866-41805942&hgt.customText=http://www.neurogenome.org/mcs/tracks/chr17.txt) | [646.17](http://genome.ucsc.edu/cgi-bin/hgTracks?db=hg17&position=chr17:41805943-42452116&hgt.customText=http://www.neurogenome.org/mcs/tracks/chr17.txt) |  | 164% | -11.08 | 249 | 22.29 | 6% | 5 |
| 56 | PCLO | 7 | [920.19](http://genome.ucsc.edu/cgi-bin/hgTracks?db=hg17&position=chr7:81717683-82637872&hgt.customText=http://www.neurogenome.org/mcs/tracks/chr7.txt) | [201.31](http://genome.ucsc.edu/cgi-bin/hgTracks?db=hg17&position=chr7:82436560-82637872&hgt.customText=http://www.neurogenome.org/mcs/tracks/chr7.txt) | [340.48](http://genome.ucsc.edu/cgi-bin/hgTracks?db=hg17&position=chr7:82096076-82436559&hgt.customText=http://www.neurogenome.org/mcs/tracks/chr7.txt) | [378.39](http://genome.ucsc.edu/cgi-bin/hgTracks?db=hg17&position=chr7:81717683-82096075&hgt.customText=http://www.neurogenome.org/mcs/tracks/chr7.txt) | 37% | -201.31 | 375 | 29.39 | 3% | 8 |
| 57 | RAB3A | 19 | [12.9](http://genome.ucsc.edu/cgi-bin/hgTracks?db=hg17&position=chr19:18168611-18181514&hgt.customText=http://www.neurogenome.org/mcs/tracks/chr19.txt) | [5.67](http://genome.ucsc.edu/cgi-bin/hgTracks?db=hg17&position=chr19:18175840-18181514&hgt.customText=http://www.neurogenome.org/mcs/tracks/chr19.txt) | [7.23](http://genome.ucsc.edu/cgi-bin/hgTracks?db=hg17&position=chr19:18168611-18175839&hgt.customText=http://www.neurogenome.org/mcs/tracks/chr19.txt) |  | 56% | -5.67 | 13 | 1.22 | 9% | 0 |
| 58 | RAB3B | 1 | [141.38](http://genome.ucsc.edu/cgi-bin/hgTracks?db=hg17&position=chr1:52056444-52197826&hgt.customText=http://www.neurogenome.org/mcs/tracks/chr1.txt) | [29.46](http://genome.ucsc.edu/cgi-bin/hgTracks?db=hg17&position=chr1:52168370-52197826&hgt.customText=http://www.neurogenome.org/mcs/tracks/chr1.txt) | [71.51](http://genome.ucsc.edu/cgi-bin/hgTracks?db=hg17&position=chr1:52096858-52168369&hgt.customText=http://www.neurogenome.org/mcs/tracks/chr1.txt) | [40.41](http://genome.ucsc.edu/cgi-bin/hgTracks?db=hg17&position=chr1:52056444-52096857&hgt.customText=http://www.neurogenome.org/mcs/tracks/chr1.txt) | 51% | -29.46 | 97 | 6.16 | 4% | 0 |
| 59 | RAB3C | 5 | [474.52](http://genome.ucsc.edu/cgi-bin/hgTracks?db=hg17&position=chr5:57827943-58302467&hgt.customText=http://www.neurogenome.org/mcs/tracks/chr5.txt) | [86.75](http://genome.ucsc.edu/cgi-bin/hgTracks?db=hg17&position=chr5:57827943-57914695&hgt.customText=http://www.neurogenome.org/mcs/tracks/chr5.txt) | [268.47](http://genome.ucsc.edu/cgi-bin/hgTracks?db=hg17&position=chr5:57914696-58183162&hgt.customText=http://www.neurogenome.org/mcs/tracks/chr5.txt) | [119.31](http://genome.ucsc.edu/cgi-bin/hgTracks?db=hg17&position=chr5:58183163-58302467&hgt.customText=http://www.neurogenome.org/mcs/tracks/chr5.txt) | 57% | -86.75 | 194 | 13.79 | 3% | 2 |
| 60 | RAB3D | 19 | [18.36](http://genome.ucsc.edu/cgi-bin/hgTracks?db=hg17&position=chr19:11296094-11314451&hgt.customText=http://www.neurogenome.org/mcs/tracks/chr19.txt) | [3.13](http://genome.ucsc.edu/cgi-bin/hgTracks?db=hg17&position=chr19:11311322-11314451&hgt.customText=http://www.neurogenome.org/mcs/tracks/chr19.txt) | [15.23](http://genome.ucsc.edu/cgi-bin/hgTracks?db=hg17&position=chr19:11296094-11311321&hgt.customText=http://www.neurogenome.org/mcs/tracks/chr19.txt) |  | 83% | -3.13 | 15 | 0.97 | 5% | 0 |
| 61 | RAB3GAP1 | 2 | [145.61](http://genome.ucsc.edu/cgi-bin/hgTracks?db=hg17&position=chr2:135615981-135761589&hgt.customText=http://www.neurogenome.org/mcs/tracks/chr2.txt) | [27.6](http://genome.ucsc.edu/cgi-bin/hgTracks?db=hg17&position=chr2:135615981-135643584&hgt.customText=http://www.neurogenome.org/mcs/tracks/chr2.txt) | [118](http://genome.ucsc.edu/cgi-bin/hgTracks?db=hg17&position=chr2:135643585-135761589&hgt.customText=http://www.neurogenome.org/mcs/tracks/chr2.txt) |  | 81% | -27.6 | 78 | 6.24 | 4% | 0 |
| 62 | RAB5A | 3 | [38.14](http://genome.ucsc.edu/cgi-bin/hgTracks?db=hg17&position=chr3:19963506-20001647&hgt.customText=http://www.neurogenome.org/mcs/tracks/chr3.txt) | [0.24](http://genome.ucsc.edu/cgi-bin/hgTracks?db=hg17&position=chr3:19963506-19963748&hgt.customText=http://www.neurogenome.org/mcs/tracks/chr3.txt) | [37.9](http://genome.ucsc.edu/cgi-bin/hgTracks?db=hg17&position=chr3:19963749-20001647&hgt.customText=http://www.neurogenome.org/mcs/tracks/chr3.txt) |  | 99% | -0.24 | 17 | 2.16 | 6% | 1 |
| 63 | RAB5B | 12 | [24.48](http://genome.ucsc.edu/cgi-bin/hgTracks?db=hg17&position=chr12:54652835-54677309&hgt.customText=http://www.neurogenome.org/mcs/tracks/chr12.txt) | [1.19](http://genome.ucsc.edu/cgi-bin/hgTracks?db=hg17&position=chr12:54652835-54654026&hgt.customText=http://www.neurogenome.org/mcs/tracks/chr12.txt) | [20.73](http://genome.ucsc.edu/cgi-bin/hgTracks?db=hg17&position=chr12:54654027-54674753&hgt.customText=http://www.neurogenome.org/mcs/tracks/chr12.txt) | [2.56](http://genome.ucsc.edu/cgi-bin/hgTracks?db=hg17&position=chr12:54674754-54677309&hgt.customText=http://www.neurogenome.org/mcs/tracks/chr12.txt) | 85% | -1.19 | 42 | 4.32 | 18% | 0 |
| 64 | RAB5C | 17 | [33.54](http://genome.ucsc.edu/cgi-bin/hgTracks?db=hg17&position=chr17:37528896-37562438&hgt.customText=http://www.neurogenome.org/mcs/tracks/chr17.txt) | [1.89](http://genome.ucsc.edu/cgi-bin/hgTracks?db=hg17&position=chr17:37560549-37562438&hgt.customText=http://www.neurogenome.org/mcs/tracks/chr17.txt) | [30.02](http://genome.ucsc.edu/cgi-bin/hgTracks?db=hg17&position=chr17:37530524-37560548&hgt.customText=http://www.neurogenome.org/mcs/tracks/chr17.txt) | [1.63](http://genome.ucsc.edu/cgi-bin/hgTracks?db=hg17&position=chr17:37528896-37530523&hgt.customText=http://www.neurogenome.org/mcs/tracks/chr17.txt) | 90% | +1.63 | 71 | 5.53 | 16% | 0 |
| 65 | RAB6IP2 | 12 | [575.94](http://genome.ucsc.edu/cgi-bin/hgTracks?db=hg17&position=chr12:969481-1545420&hgt.customText=http://www.neurogenome.org/mcs/tracks/chr12.txt) | [1.18](http://genome.ucsc.edu/cgi-bin/hgTracks?db=hg17&position=chr12:969481-970664&hgt.customText=http://www.neurogenome.org/mcs/tracks/chr12.txt) | [502.29](http://genome.ucsc.edu/cgi-bin/hgTracks?db=hg17&position=chr12:970665-1472958&hgt.customText=http://www.neurogenome.org/mcs/tracks/chr12.txt) | [72.46](http://genome.ucsc.edu/cgi-bin/hgTracks?db=hg17&position=chr12:1472959-1545420&hgt.customText=http://www.neurogenome.org/mcs/tracks/chr12.txt) | 87% | -1.18 | 231 | 19.21 | 3% | 3 |
| 66 | RABAC1 | 19 | [59.13](http://genome.ucsc.edu/cgi-bin/hgTracks?db=hg17&position=chr19:47103444-47162575&hgt.customText=http://www.neurogenome.org/mcs/tracks/chr19.txt) | [7.25](http://genome.ucsc.edu/cgi-bin/hgTracks?db=hg17&position=chr19:47155321-47162575&hgt.customText=http://www.neurogenome.org/mcs/tracks/chr19.txt) | [2.64](http://genome.ucsc.edu/cgi-bin/hgTracks?db=hg17&position=chr19:47152678-47155320&hgt.customText=http://www.neurogenome.org/mcs/tracks/chr19.txt) | [49.23](http://genome.ucsc.edu/cgi-bin/hgTracks?db=hg17&position=chr19:47103444-47152677&hgt.customText=http://www.neurogenome.org/mcs/tracks/chr19.txt) | 4% | -7.25 | 69 | 7.31 | 12% | 3 |
| 67 | RABGEF1 | 7 | [280.42](http://genome.ucsc.edu/cgi-bin/hgTracks?db=hg17&position=chr7:65549968-65830383&hgt.customText=http://www.neurogenome.org/mcs/tracks/chr7.txt) | [99.9](http://genome.ucsc.edu/cgi-bin/hgTracks?db=hg17&position=chr7:65549968-65649869&hgt.customText=http://www.neurogenome.org/mcs/tracks/chr7.txt) | [70.73](http://genome.ucsc.edu/cgi-bin/hgTracks?db=hg17&position=chr7:65649870-65720596&hgt.customText=http://www.neurogenome.org/mcs/tracks/chr7.txt) | [109.79](http://genome.ucsc.edu/cgi-bin/hgTracks?db=hg17&position=chr7:65720597-65830383&hgt.customText=http://www.neurogenome.org/mcs/tracks/chr7.txt) | 25% | -99.9 | 115 | 8.75 | 3% | 0 |
| 68 | RABGGTA | 14 | [27.43](http://genome.ucsc.edu/cgi-bin/hgTracks?db=hg17&position=chr14:23802213-23829645&hgt.customText=http://www.neurogenome.org/mcs/tracks/chr14.txt) | [19](http://genome.ucsc.edu/cgi-bin/hgTracks?db=hg17&position=chr14:23810644-23829645&hgt.customText=http://www.neurogenome.org/mcs/tracks/chr14.txt) | [6.06](http://genome.ucsc.edu/cgi-bin/hgTracks?db=hg17&position=chr14:23804586-23810643&hgt.customText=http://www.neurogenome.org/mcs/tracks/chr14.txt) | [2.37](http://genome.ucsc.edu/cgi-bin/hgTracks?db=hg17&position=chr14:23802213-23804585&hgt.customText=http://www.neurogenome.org/mcs/tracks/chr14.txt) | 22% | +2.37 | 44 | 2.87 | 10% | 0 |
| 69 | RABGGTB | 1 | [33.38](http://genome.ucsc.edu/cgi-bin/hgTracks?db=hg17&position=chr1:75941205-75974587&hgt.customText=http://www.neurogenome.org/mcs/tracks/chr1.txt) | [22.7](http://genome.ucsc.edu/cgi-bin/hgTracks?db=hg17&position=chr1:75941205-75963906&hgt.customText=http://www.neurogenome.org/mcs/tracks/chr1.txt) | [8.88](http://genome.ucsc.edu/cgi-bin/hgTracks?db=hg17&position=chr1:75963907-75972785&hgt.customText=http://www.neurogenome.org/mcs/tracks/chr1.txt) | [1.8](http://genome.ucsc.edu/cgi-bin/hgTracks?db=hg17&position=chr1:75972786-75974587&hgt.customText=http://www.neurogenome.org/mcs/tracks/chr1.txt) | 27% | +1.8 | 18 | 1.71 | 5% | 0 |
| 70 | RABIF | 1 | [15.86](http://genome.ucsc.edu/cgi-bin/hgTracks?db=hg17&position=chr1:199576027-199591886&hgt.customText=http://www.neurogenome.org/mcs/tracks/chr1.txt) | [1.97](http://genome.ucsc.edu/cgi-bin/hgTracks?db=hg17&position=chr1:199589921-199591886&hgt.customText=http://www.neurogenome.org/mcs/tracks/chr1.txt) | [10.18](http://genome.ucsc.edu/cgi-bin/hgTracks?db=hg17&position=chr1:199579743-199589920&hgt.customText=http://www.neurogenome.org/mcs/tracks/chr1.txt) | [3.72](http://genome.ucsc.edu/cgi-bin/hgTracks?db=hg17&position=chr1:199576027-199579742&hgt.customText=http://www.neurogenome.org/mcs/tracks/chr1.txt) | 64% | -1.97 | 8 | 0.51 | 3% | 0 |
| 71 | RIMBP2 | 12 | [417.27](http://genome.ucsc.edu/cgi-bin/hgTracks?db=hg17&position=chr12:129381753-129799027&hgt.customText=http://www.neurogenome.org/mcs/tracks/chr12.txt) | [73.32](http://genome.ucsc.edu/cgi-bin/hgTracks?db=hg17&position=chr12:129725707-129799027&hgt.customText=http://www.neurogenome.org/mcs/tracks/chr12.txt) | [320.14](http://genome.ucsc.edu/cgi-bin/hgTracks?db=hg17&position=chr12:129405562-129725706&hgt.customText=http://www.neurogenome.org/mcs/tracks/chr12.txt) | [23.81](http://genome.ucsc.edu/cgi-bin/hgTracks?db=hg17&position=chr12:129381753-129405561&hgt.customText=http://www.neurogenome.org/mcs/tracks/chr12.txt) | 77% | +23.81 | 114 | 6.72 | 2% | 1 |
| 72 | RIMS1 | 6 | [1201.38](http://genome.ucsc.edu/cgi-bin/hgTracks?db=hg17&position=chr6:72187170-73388554&hgt.customText=http://www.neurogenome.org/mcs/tracks/chr6.txt) | [465.96](http://genome.ucsc.edu/cgi-bin/hgTracks?db=hg17&position=chr6:72187170-72653126&hgt.customText=http://www.neurogenome.org/mcs/tracks/chr6.txt) | [516.1](http://genome.ucsc.edu/cgi-bin/hgTracks?db=hg17&position=chr6:72653127-73169228&hgt.customText=http://www.neurogenome.org/mcs/tracks/chr6.txt) | [219.33](http://genome.ucsc.edu/cgi-bin/hgTracks?db=hg17&position=chr6:73169229-73388554&hgt.customText=http://www.neurogenome.org/mcs/tracks/chr6.txt) | 43% | +219.33 | 621 | 50.88 | 4% | 5 |
| 73 | RIMS2 | 8 | [896.85](http://genome.ucsc.edu/cgi-bin/hgTracks?db=hg17&position=chr8:104524377-105421227&hgt.customText=http://www.neurogenome.org/mcs/tracks/chr8.txt) | [57.77](http://genome.ucsc.edu/cgi-bin/hgTracks?db=hg17&position=chr8:104524377-104582151&hgt.customText=http://www.neurogenome.org/mcs/tracks/chr8.txt) | [751.78](http://genome.ucsc.edu/cgi-bin/hgTracks?db=hg17&position=chr8:104582152-105333929&hgt.customText=http://www.neurogenome.org/mcs/tracks/chr8.txt) | [87.3](http://genome.ucsc.edu/cgi-bin/hgTracks?db=hg17&position=chr8:105333930-105421227&hgt.customText=http://www.neurogenome.org/mcs/tracks/chr8.txt) | 84% | -57.77 | 329 | 28.68 | 3% | 12 |
| 74 | RIMS3 | 1 | [142.21](http://genome.ucsc.edu/cgi-bin/hgTracks?db=hg17&position=chr1:40682933-40825144&hgt.customText=http://www.neurogenome.org/mcs/tracks/chr1.txt) | [24.72](http://genome.ucsc.edu/cgi-bin/hgTracks?db=hg17&position=chr1:40800422-40825144&hgt.customText=http://www.neurogenome.org/mcs/tracks/chr1.txt) | [44.98](http://genome.ucsc.edu/cgi-bin/hgTracks?db=hg17&position=chr1:40755445-40800421&hgt.customText=http://www.neurogenome.org/mcs/tracks/chr1.txt) | [72.51](http://genome.ucsc.edu/cgi-bin/hgTracks?db=hg17&position=chr1:40682933-40755444&hgt.customText=http://www.neurogenome.org/mcs/tracks/chr1.txt) | 32% | -24.72 | 127 | 9.19 | 6% | 1 |
| 75 | RIMS4 | 20 | [134.68](http://genome.ucsc.edu/cgi-bin/hgTracks?db=hg17&position=chr20:42813081-42947757&hgt.customText=http://www.neurogenome.org/mcs/tracks/chr20.txt) | [75.43](http://genome.ucsc.edu/cgi-bin/hgTracks?db=hg17&position=chr20:42872327-42947757&hgt.customText=http://www.neurogenome.org/mcs/tracks/chr20.txt) | [58.46](http://genome.ucsc.edu/cgi-bin/hgTracks?db=hg17&position=chr20:42813863-42872326&hgt.customText=http://www.neurogenome.org/mcs/tracks/chr20.txt) | [0.78](http://genome.ucsc.edu/cgi-bin/hgTracks?db=hg17&position=chr20:42813081-42813862&hgt.customText=http://www.neurogenome.org/mcs/tracks/chr20.txt) | 43% | +0.78 | 112 | 9.39 | 7% | 1 |
| 76 | RPH3A | 12 | [397.02](http://genome.ucsc.edu/cgi-bin/hgTracks?db=hg17&position=chr12:111410438-111807458&hgt.customText=http://www.neurogenome.org/mcs/tracks/chr12.txt) | [281.95](http://genome.ucsc.edu/cgi-bin/hgTracks?db=hg17&position=chr12:111410438-111692392&hgt.customText=http://www.neurogenome.org/mcs/tracks/chr12.txt) | [107.01](http://genome.ucsc.edu/cgi-bin/hgTracks?db=hg17&position=chr12:111692393-111799402&hgt.customText=http://www.neurogenome.org/mcs/tracks/chr12.txt) | [8.06](http://genome.ucsc.edu/cgi-bin/hgTracks?db=hg17&position=chr12:111799403-111807458&hgt.customText=http://www.neurogenome.org/mcs/tracks/chr12.txt) | 27% | +8.06 | 150 | 9.44 | 2% | 1 |
| 77 | SCAMP1 | 5 | [190.53](http://genome.ucsc.edu/cgi-bin/hgTracks?db=hg17&position=chr5:77626285-77816809&hgt.customText=http://www.neurogenome.org/mcs/tracks/chr5.txt) | [65.83](http://genome.ucsc.edu/cgi-bin/hgTracks?db=hg17&position=chr5:77626285-77692109&hgt.customText=http://www.neurogenome.org/mcs/tracks/chr5.txt) | [118.53](http://genome.ucsc.edu/cgi-bin/hgTracks?db=hg17&position=chr5:77692110-77810634&hgt.customText=http://www.neurogenome.org/mcs/tracks/chr5.txt) | [6.17](http://genome.ucsc.edu/cgi-bin/hgTracks?db=hg17&position=chr5:77810635-77816809&hgt.customText=http://www.neurogenome.org/mcs/tracks/chr5.txt) | 62% | +6.17 | 75 | 4.89 | 3% | 0 |
| 78 | SCAMP2 | 15 | [46.7](http://genome.ucsc.edu/cgi-bin/hgTracks?db=hg17&position=chr15:72922741-72969435&hgt.customText=http://www.neurogenome.org/mcs/tracks/chr15.txt) | [16.71](http://genome.ucsc.edu/cgi-bin/hgTracks?db=hg17&position=chr15:72952729-72969435&hgt.customText=http://www.neurogenome.org/mcs/tracks/chr15.txt) | [28.48](http://genome.ucsc.edu/cgi-bin/hgTracks?db=hg17&position=chr15:72924249-72952728&hgt.customText=http://www.neurogenome.org/mcs/tracks/chr15.txt) | [1.51](http://genome.ucsc.edu/cgi-bin/hgTracks?db=hg17&position=chr15:72922741-72924248&hgt.customText=http://www.neurogenome.org/mcs/tracks/chr15.txt) | 61% | +1.51 | 45 | 2.84 | 6% | 0 |
| 79 | SCAMP3 | 1 | [7.38](http://genome.ucsc.edu/cgi-bin/hgTracks?db=hg17&position=chr1:152038348-152045732&hgt.customText=http://www.neurogenome.org/mcs/tracks/chr1.txt) | [0.46](http://genome.ucsc.edu/cgi-bin/hgTracks?db=hg17&position=chr1:152045269-152045732&hgt.customText=http://www.neurogenome.org/mcs/tracks/chr1.txt) | [6.43](http://genome.ucsc.edu/cgi-bin/hgTracks?db=hg17&position=chr1:152038843-152045268&hgt.customText=http://www.neurogenome.org/mcs/tracks/chr1.txt) | [0.49](http://genome.ucsc.edu/cgi-bin/hgTracks?db=hg17&position=chr1:152038348-152038842&hgt.customText=http://www.neurogenome.org/mcs/tracks/chr1.txt) | 87% | -0.46 | 22 | 1.5 | 20% | 0 |
| 80 | SCAMP4 | 19 | [55.69](http://genome.ucsc.edu/cgi-bin/hgTracks?db=hg17&position=chr19:1836501-1892187&hgt.customText=http://www.neurogenome.org/mcs/tracks/chr19.txt) | [19.87](http://genome.ucsc.edu/cgi-bin/hgTracks?db=hg17&position=chr19:1836501-1856370&hgt.customText=http://www.neurogenome.org/mcs/tracks/chr19.txt) | [20.64](http://genome.ucsc.edu/cgi-bin/hgTracks?db=hg17&position=chr19:1856371-1877011&hgt.customText=http://www.neurogenome.org/mcs/tracks/chr19.txt) | [15.18](http://genome.ucsc.edu/cgi-bin/hgTracks?db=hg17&position=chr19:1877012-1892187&hgt.customText=http://www.neurogenome.org/mcs/tracks/chr19.txt) | 37% | +15.18 | 30 | 1.68 | 3% | 0 |
| 81 | SCAMP5 | 15 | [66.15](http://genome.ucsc.edu/cgi-bin/hgTracks?db=hg17&position=chr15:73036829-73102979&hgt.customText=http://www.neurogenome.org/mcs/tracks/chr15.txt) | [38.1](http://genome.ucsc.edu/cgi-bin/hgTracks?db=hg17&position=chr15:73036829-73074928&hgt.customText=http://www.neurogenome.org/mcs/tracks/chr15.txt) | [25.96](http://genome.ucsc.edu/cgi-bin/hgTracks?db=hg17&position=chr15:73074929-73100889&hgt.customText=http://www.neurogenome.org/mcs/tracks/chr15.txt) | [2.09](http://genome.ucsc.edu/cgi-bin/hgTracks?db=hg17&position=chr15:73100890-73102979&hgt.customText=http://www.neurogenome.org/mcs/tracks/chr15.txt) | 39% | +2.09 | 57 | 4.55 | 7% | 1 |
| 82 | SLC30A3 | 2 | [31.63](http://genome.ucsc.edu/cgi-bin/hgTracks?db=hg17&position=chr2:27378306-27409939&hgt.customText=http://www.neurogenome.org/mcs/tracks/chr2.txt) | [12.33](http://genome.ucsc.edu/cgi-bin/hgTracks?db=hg17&position=chr2:27397612-27409939&hgt.customText=http://www.neurogenome.org/mcs/tracks/chr2.txt) | [9.41](http://genome.ucsc.edu/cgi-bin/hgTracks?db=hg17&position=chr2:27388203-27397611&hgt.customText=http://www.neurogenome.org/mcs/tracks/chr2.txt) | [9.9](http://genome.ucsc.edu/cgi-bin/hgTracks?db=hg17&position=chr2:27378306-27388202&hgt.customText=http://www.neurogenome.org/mcs/tracks/chr2.txt) | 30% | +9.9 | 44 | 4.09 | 13% | 1 |
| 83 | SLC30A4 | 15 | [40.33](http://genome.ucsc.edu/cgi-bin/hgTracks?db=hg17&position=chr15:43561970-43602294&hgt.customText=http://www.neurogenome.org/mcs/tracks/chr15.txt) |  | [40.33](http://genome.ucsc.edu/cgi-bin/hgTracks?db=hg17&position=chr15:43561970-43602294&hgt.customText=http://www.neurogenome.org/mcs/tracks/chr15.txt) |  | 100% | -0 | 37 | 2.64 | 7% | 0 |
| 84 | SNAP25 | 20 | [348.43](http://genome.ucsc.edu/cgi-bin/hgTracks?db=hg17&position=chr20:9985406-10333832&hgt.customText=http://www.neurogenome.org/mcs/tracks/chr20.txt) | [162.07](http://genome.ucsc.edu/cgi-bin/hgTracks?db=hg17&position=chr20:9985406-10147476&hgt.customText=http://www.neurogenome.org/mcs/tracks/chr20.txt) | [88.59](http://genome.ucsc.edu/cgi-bin/hgTracks?db=hg17&position=chr20:10147477-10236065&hgt.customText=http://www.neurogenome.org/mcs/tracks/chr20.txt) | [97.77](http://genome.ucsc.edu/cgi-bin/hgTracks?db=hg17&position=chr20:10236066-10333832&hgt.customText=http://www.neurogenome.org/mcs/tracks/chr20.txt) | 25% | +97.77 | 241 | 22.13 | 6% | 4 |
| 85 | SNAPAP | 1 | [15.73](http://genome.ucsc.edu/cgi-bin/hgTracks?db=hg17&position=chr1:150431855-150447586&hgt.customText=http://www.neurogenome.org/mcs/tracks/chr1.txt) | [12.37](http://genome.ucsc.edu/cgi-bin/hgTracks?db=hg17&position=chr1:150431855-150444225&hgt.customText=http://www.neurogenome.org/mcs/tracks/chr1.txt) | [3.15](http://genome.ucsc.edu/cgi-bin/hgTracks?db=hg17&position=chr1:150444226-150447378&hgt.customText=http://www.neurogenome.org/mcs/tracks/chr1.txt) | [0.21](http://genome.ucsc.edu/cgi-bin/hgTracks?db=hg17&position=chr1:150447379-150447586&hgt.customText=http://www.neurogenome.org/mcs/tracks/chr1.txt) | 20% | +0.21 | 11 | 0.97 | 6% | 0 |
| 86 | SNCA | 4 | [587.06](http://genome.ucsc.edu/cgi-bin/hgTracks?db=hg17&position=chr4:90586123-91173180&hgt.customText=http://www.neurogenome.org/mcs/tracks/chr4.txt) | [57.87](http://genome.ucsc.edu/cgi-bin/hgTracks?db=hg17&position=chr4:91115312-91173180&hgt.customText=http://www.neurogenome.org/mcs/tracks/chr4.txt) | [111.43](http://genome.ucsc.edu/cgi-bin/hgTracks?db=hg17&position=chr4:91003883-91115311&hgt.customText=http://www.neurogenome.org/mcs/tracks/chr4.txt) | [417.76](http://genome.ucsc.edu/cgi-bin/hgTracks?db=hg17&position=chr4:90586123-91003882&hgt.customText=http://www.neurogenome.org/mcs/tracks/chr4.txt) | 19% | -57.87 | 132 | 8.92 | 2% | 2 |
| 87 | STX10 | 19 | [9.32](http://genome.ucsc.edu/cgi-bin/hgTracks?db=hg17&position=chr19:13112959-13122281&hgt.customText=http://www.neurogenome.org/mcs/tracks/chr19.txt) | [0.12](http://genome.ucsc.edu/cgi-bin/hgTracks?db=hg17&position=chr19:13122161-13122281&hgt.customText=http://www.neurogenome.org/mcs/tracks/chr19.txt) | [6.29](http://genome.ucsc.edu/cgi-bin/hgTracks?db=hg17&position=chr19:13115873-13122160&hgt.customText=http://www.neurogenome.org/mcs/tracks/chr19.txt) | [2.91](http://genome.ucsc.edu/cgi-bin/hgTracks?db=hg17&position=chr19:13112959-13115872&hgt.customText=http://www.neurogenome.org/mcs/tracks/chr19.txt) | 67% | -0.12 | 21 | 0.67 | 7% | 0 |
| 88 | STX11 | 6 | [104.83](http://genome.ucsc.edu/cgi-bin/hgTracks?db=hg17&position=chr6:144458448-144563282&hgt.customText=http://www.neurogenome.org/mcs/tracks/chr6.txt) | [54.91](http://genome.ucsc.edu/cgi-bin/hgTracks?db=hg17&position=chr6:144458448-144513361&hgt.customText=http://www.neurogenome.org/mcs/tracks/chr6.txt) | [37.83](http://genome.ucsc.edu/cgi-bin/hgTracks?db=hg17&position=chr6:144513362-144551194&hgt.customText=http://www.neurogenome.org/mcs/tracks/chr6.txt) | [12.09](http://genome.ucsc.edu/cgi-bin/hgTracks?db=hg17&position=chr6:144551195-144563282&hgt.customText=http://www.neurogenome.org/mcs/tracks/chr6.txt) | 36% | +12.09 | 28 | 1.97 | 2% | 0 |
| 89 | STX12 | 1 | [68.82](http://genome.ucsc.edu/cgi-bin/hgTracks?db=hg17&position=chr1:27772618-27841434&hgt.customText=http://www.neurogenome.org/mcs/tracks/chr1.txt) | [11.31](http://genome.ucsc.edu/cgi-bin/hgTracks?db=hg17&position=chr1:27772618-27783924&hgt.customText=http://www.neurogenome.org/mcs/tracks/chr1.txt) | [51.03](http://genome.ucsc.edu/cgi-bin/hgTracks?db=hg17&position=chr1:27783925-27834956&hgt.customText=http://www.neurogenome.org/mcs/tracks/chr1.txt) | [6.48](http://genome.ucsc.edu/cgi-bin/hgTracks?db=hg17&position=chr1:27834957-27841434&hgt.customText=http://www.neurogenome.org/mcs/tracks/chr1.txt) | 74% | +6.48 | 53 | 3.8 | 6% | 0 |
| 90 | STX16 | 20 | [56.26](http://genome.ucsc.edu/cgi-bin/hgTracks?db=hg17&position=chr20:56644196-56700459&hgt.customText=http://www.neurogenome.org/mcs/tracks/chr20.txt) | [15.54](http://genome.ucsc.edu/cgi-bin/hgTracks?db=hg17&position=chr20:56644196-56659733&hgt.customText=http://www.neurogenome.org/mcs/tracks/chr20.txt) | [28.25](http://genome.ucsc.edu/cgi-bin/hgTracks?db=hg17&position=chr20:56659734-56687986&hgt.customText=http://www.neurogenome.org/mcs/tracks/chr20.txt) | [12.47](http://genome.ucsc.edu/cgi-bin/hgTracks?db=hg17&position=chr20:56687987-56700459&hgt.customText=http://www.neurogenome.org/mcs/tracks/chr20.txt) | 50% | +12.47 | 69 | 7.68 | 14% | 3 |
| 91 | STX17 | 9 | [112.29](http://genome.ucsc.edu/cgi-bin/hgTracks?db=hg17&position=chr9:99708730-99821015&hgt.customText=http://www.neurogenome.org/mcs/tracks/chr9.txt) | [39.78](http://genome.ucsc.edu/cgi-bin/hgTracks?db=hg17&position=chr9:99708730-99748506&hgt.customText=http://www.neurogenome.org/mcs/tracks/chr9.txt) | [63.67](http://genome.ucsc.edu/cgi-bin/hgTracks?db=hg17&position=chr9:99748507-99812176&hgt.customText=http://www.neurogenome.org/mcs/tracks/chr9.txt) | [8.84](http://genome.ucsc.edu/cgi-bin/hgTracks?db=hg17&position=chr9:99812177-99821015&hgt.customText=http://www.neurogenome.org/mcs/tracks/chr9.txt) | 57% | +8.84 | 347 | 18.69 | 17% | 3 |
| 92 | STX18 | 4 | [440.7](http://genome.ucsc.edu/cgi-bin/hgTracks?db=hg17&position=chr4:4538770-4979470&hgt.customText=http://www.neurogenome.org/mcs/tracks/chr4.txt) | [317.62](http://genome.ucsc.edu/cgi-bin/hgTracks?db=hg17&position=chr4:4661848-4979470&hgt.customText=http://www.neurogenome.org/mcs/tracks/chr4.txt) | [123.08](http://genome.ucsc.edu/cgi-bin/hgTracks?db=hg17&position=chr4:4538770-4661847&hgt.customText=http://www.neurogenome.org/mcs/tracks/chr4.txt) |  | 28% | -317.62 | 268 | 26.41 | 6% | 11 |
| 93 | STX19 | 3 | [14.24](http://genome.ucsc.edu/cgi-bin/hgTracks?db=hg17&position=chr3:95215905-95230144&hgt.customText=http://www.neurogenome.org/mcs/tracks/chr3.txt) |  | [14.24](http://genome.ucsc.edu/cgi-bin/hgTracks?db=hg17&position=chr3:95215905-95230144&hgt.customText=http://www.neurogenome.org/mcs/tracks/chr3.txt) |  | 100% | -0 | 11 | 0.61 | 4% | 0 |
| 94 | STX1A | 7 | [37.88](http://genome.ucsc.edu/cgi-bin/hgTracks?db=hg17&position=chr7:72557193-72595077&hgt.customText=http://www.neurogenome.org/mcs/tracks/chr7.txt) | [16.44](http://genome.ucsc.edu/cgi-bin/hgTracks?db=hg17&position=chr7:72578641-72595077&hgt.customText=http://www.neurogenome.org/mcs/tracks/chr7.txt) | [20.45](http://genome.ucsc.edu/cgi-bin/hgTracks?db=hg17&position=chr7:72558191-72578640&hgt.customText=http://www.neurogenome.org/mcs/tracks/chr7.txt) | [1](http://genome.ucsc.edu/cgi-bin/hgTracks?db=hg17&position=chr7:72557193-72558190&hgt.customText=http://www.neurogenome.org/mcs/tracks/chr7.txt) | 54% | +1 | 48 | 3.44 | 9% | 0 |
| 95 | STX1B2 | 16 | [44.43](http://genome.ucsc.edu/cgi-bin/hgTracks?db=hg17&position=chr16:30907973-30952403&hgt.customText=http://www.neurogenome.org/mcs/tracks/chr16.txt) | [23.13](http://genome.ucsc.edu/cgi-bin/hgTracks?db=hg17&position=chr16:30929277-30952403&hgt.customText=http://www.neurogenome.org/mcs/tracks/chr16.txt) | [21.2](http://genome.ucsc.edu/cgi-bin/hgTracks?db=hg17&position=chr16:30908080-30929276&hgt.customText=http://www.neurogenome.org/mcs/tracks/chr16.txt) | [0.11](http://genome.ucsc.edu/cgi-bin/hgTracks?db=hg17&position=chr16:30907973-30908079&hgt.customText=http://www.neurogenome.org/mcs/tracks/chr16.txt) | 48% | +0.11 | 39 | 4.44 | 10% | 2 |
| 96 | STX3A | 11 | [92.29](http://genome.ucsc.edu/cgi-bin/hgTracks?db=hg17&position=chr11:59237895-59330183&hgt.customText=http://www.neurogenome.org/mcs/tracks/chr11.txt) | [41.21](http://genome.ucsc.edu/cgi-bin/hgTracks?db=hg17&position=chr11:59237895-59279107&hgt.customText=http://www.neurogenome.org/mcs/tracks/chr11.txt) | [47.65](http://genome.ucsc.edu/cgi-bin/hgTracks?db=hg17&position=chr11:59279108-59326752&hgt.customText=http://www.neurogenome.org/mcs/tracks/chr11.txt) | [3.43](http://genome.ucsc.edu/cgi-bin/hgTracks?db=hg17&position=chr11:59326753-59330183&hgt.customText=http://www.neurogenome.org/mcs/tracks/chr11.txt) | 52% | +3.43 | 72 | 4.07 | 4% | 0 |
| 97 | STX4A | 16 | [50.4](http://genome.ucsc.edu/cgi-bin/hgTracks?db=hg17&position=chr16:30929277-30979672&hgt.customText=http://www.neurogenome.org/mcs/tracks/chr16.txt) | [23.13](http://genome.ucsc.edu/cgi-bin/hgTracks?db=hg17&position=chr16:30929277-30952403&hgt.customText=http://www.neurogenome.org/mcs/tracks/chr16.txt) | [6.58](http://genome.ucsc.edu/cgi-bin/hgTracks?db=hg17&position=chr16:30952404-30958985&hgt.customText=http://www.neurogenome.org/mcs/tracks/chr16.txt) | [20.69](http://genome.ucsc.edu/cgi-bin/hgTracks?db=hg17&position=chr16:30958986-30979672&hgt.customText=http://www.neurogenome.org/mcs/tracks/chr16.txt) | 13% | +20.69 | 24 | 2.28 | 5% | 0 |
| 98 | STX5A | 11 | [27.43](http://genome.ucsc.edu/cgi-bin/hgTracks?db=hg17&position=chr11:62329530-62356959&hgt.customText=http://www.neurogenome.org/mcs/tracks/chr11.txt) | [0.82](http://genome.ucsc.edu/cgi-bin/hgTracks?db=hg17&position=chr11:62356137-62356959&hgt.customText=http://www.neurogenome.org/mcs/tracks/chr11.txt) | [25.19](http://genome.ucsc.edu/cgi-bin/hgTracks?db=hg17&position=chr11:62330946-62356136&hgt.customText=http://www.neurogenome.org/mcs/tracks/chr11.txt) | [1.42](http://genome.ucsc.edu/cgi-bin/hgTracks?db=hg17&position=chr11:62329530-62330945&hgt.customText=http://www.neurogenome.org/mcs/tracks/chr11.txt) | 92% | -0.82 | 27 | 2.53 | 9% | 0 |
| 99 | STX6 | 1 | [89.96](http://genome.ucsc.edu/cgi-bin/hgTracks?db=hg17&position=chr1:177644832-177734792&hgt.customText=http://www.neurogenome.org/mcs/tracks/chr1.txt) | [11.09](http://genome.ucsc.edu/cgi-bin/hgTracks?db=hg17&position=chr1:177723704-177734792&hgt.customText=http://www.neurogenome.org/mcs/tracks/chr1.txt) | [78.87](http://genome.ucsc.edu/cgi-bin/hgTracks?db=hg17&position=chr1:177644832-177723703&hgt.customText=http://www.neurogenome.org/mcs/tracks/chr1.txt) |  | 88% | -11.09 | 94 | 6.17 | 7% | 2 |
| 100 | STX7 | 6 | [136.79](http://genome.ucsc.edu/cgi-bin/hgTracks?db=hg17&position=chr6:132764326-132901119&hgt.customText=http://www.neurogenome.org/mcs/tracks/chr6.txt) | [25.26](http://genome.ucsc.edu/cgi-bin/hgTracks?db=hg17&position=chr6:132875860-132901119&hgt.customText=http://www.neurogenome.org/mcs/tracks/chr6.txt) | [53.01](http://genome.ucsc.edu/cgi-bin/hgTracks?db=hg17&position=chr6:132822848-132875859&hgt.customText=http://www.neurogenome.org/mcs/tracks/chr6.txt) | [58.52](http://genome.ucsc.edu/cgi-bin/hgTracks?db=hg17&position=chr6:132764326-132822847&hgt.customText=http://www.neurogenome.org/mcs/tracks/chr6.txt) | 39% | -25.26 | 51 | 3.35 | 2% | 0 |
| 101 | STX8 | 17 | [336.61](http://genome.ucsc.edu/cgi-bin/hgTracks?db=hg17&position=chr17:9084079-9420683&hgt.customText=http://www.neurogenome.org/mcs/tracks/chr17.txt) | [0.68](http://genome.ucsc.edu/cgi-bin/hgTracks?db=hg17&position=chr17:9420001-9420683&hgt.customText=http://www.neurogenome.org/mcs/tracks/chr17.txt) | [325.49](http://genome.ucsc.edu/cgi-bin/hgTracks?db=hg17&position=chr17:9094514-9420000&hgt.customText=http://www.neurogenome.org/mcs/tracks/chr17.txt) | [10.44](http://genome.ucsc.edu/cgi-bin/hgTracks?db=hg17&position=chr17:9084079-9094513&hgt.customText=http://www.neurogenome.org/mcs/tracks/chr17.txt) | 97% | -0.68 | 211 | 18.37 | 5% | 5 |
| 102 | STXBP1 | 9 | [128](http://genome.ucsc.edu/cgi-bin/hgTracks?db=hg17&position=chr9:127420823-127548821&hgt.customText=http://www.neurogenome.org/mcs/tracks/chr9.txt) | [33.3](http://genome.ucsc.edu/cgi-bin/hgTracks?db=hg17&position=chr9:127420823-127454121&hgt.customText=http://www.neurogenome.org/mcs/tracks/chr9.txt) | [80.43](http://genome.ucsc.edu/cgi-bin/hgTracks?db=hg17&position=chr9:127454122-127534549&hgt.customText=http://www.neurogenome.org/mcs/tracks/chr9.txt) | [14.27](http://genome.ucsc.edu/cgi-bin/hgTracks?db=hg17&position=chr9:127534550-127548821&hgt.customText=http://www.neurogenome.org/mcs/tracks/chr9.txt) | 63% | +14.27 | 78 | 5.86 | 5% | 1 |
| 103 | STXBP2 | 19 | [35.34](http://genome.ucsc.edu/cgi-bin/hgTracks?db=hg17&position=chr19:7604637-7639971&hgt.customText=http://www.neurogenome.org/mcs/tracks/chr19.txt) | [3.38](http://genome.ucsc.edu/cgi-bin/hgTracks?db=hg17&position=chr19:7604637-7608012&hgt.customText=http://www.neurogenome.org/mcs/tracks/chr19.txt) | [10.75](http://genome.ucsc.edu/cgi-bin/hgTracks?db=hg17&position=chr19:7608013-7618758&hgt.customText=http://www.neurogenome.org/mcs/tracks/chr19.txt) | [21.21](http://genome.ucsc.edu/cgi-bin/hgTracks?db=hg17&position=chr19:7618759-7639971&hgt.customText=http://www.neurogenome.org/mcs/tracks/chr19.txt) | 30% | -3.38 | 34 | 2.86 | 8% | 0 |
| 104 | STXBP3 | 1 | [73.15](http://genome.ucsc.edu/cgi-bin/hgTracks?db=hg17&position=chr1:108997408-109070557&hgt.customText=http://www.neurogenome.org/mcs/tracks/chr1.txt) | [3.94](http://genome.ucsc.edu/cgi-bin/hgTracks?db=hg17&position=chr1:108997408-109001349&hgt.customText=http://www.neurogenome.org/mcs/tracks/chr1.txt) | [62.84](http://genome.ucsc.edu/cgi-bin/hgTracks?db=hg17&position=chr1:109001350-109064190&hgt.customText=http://www.neurogenome.org/mcs/tracks/chr1.txt) | [6.37](http://genome.ucsc.edu/cgi-bin/hgTracks?db=hg17&position=chr1:109064191-109070557&hgt.customText=http://www.neurogenome.org/mcs/tracks/chr1.txt) | 86% | -3.94 | 58 | 3.07 | 4% | 0 |
| 105 | STXBP4 | 17 | [296.32](http://genome.ucsc.edu/cgi-bin/hgTracks?db=hg17&position=chr17:50401054-50697369&hgt.customText=http://www.neurogenome.org/mcs/tracks/chr17.txt) | [0.07](http://genome.ucsc.edu/cgi-bin/hgTracks?db=hg17&position=chr17:50401054-50401124&hgt.customText=http://www.neurogenome.org/mcs/tracks/chr17.txt) | [195.32](http://genome.ucsc.edu/cgi-bin/hgTracks?db=hg17&position=chr17:50401125-50596448&hgt.customText=http://www.neurogenome.org/mcs/tracks/chr17.txt) | [100.92](http://genome.ucsc.edu/cgi-bin/hgTracks?db=hg17&position=chr17:50596449-50697369&hgt.customText=http://www.neurogenome.org/mcs/tracks/chr17.txt) | 66% | -0.07 | 182 | 13.12 | 4% | 1 |
| 106 | STXBP5 | 6 | [693.47](http://genome.ucsc.edu/cgi-bin/hgTracks?db=hg17&position=chr6:147178289-147871755&hgt.customText=http://www.neurogenome.org/mcs/tracks/chr6.txt) | [388.28](http://genome.ucsc.edu/cgi-bin/hgTracks?db=hg17&position=chr6:147178289-147566567&hgt.customText=http://www.neurogenome.org/mcs/tracks/chr6.txt) | [182.02](http://genome.ucsc.edu/cgi-bin/hgTracks?db=hg17&position=chr6:147566568-147748584&hgt.customText=http://www.neurogenome.org/mcs/tracks/chr6.txt) | [123.17](http://genome.ucsc.edu/cgi-bin/hgTracks?db=hg17&position=chr6:147748585-147871755&hgt.customText=http://www.neurogenome.org/mcs/tracks/chr6.txt) | 26% | +123.17 | 336 | 27.2 | 4% | 1 |
| 107 | STXBP6 | 14 | [632.8](http://genome.ucsc.edu/cgi-bin/hgTracks?db=hg17&position=chr14:24173314-24806109&hgt.customText=http://www.neurogenome.org/mcs/tracks/chr14.txt) | [217](http://genome.ucsc.edu/cgi-bin/hgTracks?db=hg17&position=chr14:24589111-24806109&hgt.customText=http://www.neurogenome.org/mcs/tracks/chr14.txt) | [240.4](http://genome.ucsc.edu/cgi-bin/hgTracks?db=hg17&position=chr14:24348708-24589110&hgt.customText=http://www.neurogenome.org/mcs/tracks/chr14.txt) | [175.39](http://genome.ucsc.edu/cgi-bin/hgTracks?db=hg17&position=chr14:24173314-24348707&hgt.customText=http://www.neurogenome.org/mcs/tracks/chr14.txt) | 38% | +175.39 | 314 | 23.2 | 4% | 4 |
| 108 | SV2A | 1 | [22.86](http://genome.ucsc.edu/cgi-bin/hgTracks?db=hg17&position=chr1:146685421-146708282&hgt.customText=http://www.neurogenome.org/mcs/tracks/chr1.txt) | [5.78](http://genome.ucsc.edu/cgi-bin/hgTracks?db=hg17&position=chr1:146702508-146708282&hgt.customText=http://www.neurogenome.org/mcs/tracks/chr1.txt) | [14.56](http://genome.ucsc.edu/cgi-bin/hgTracks?db=hg17&position=chr1:146687949-146702507&hgt.customText=http://www.neurogenome.org/mcs/tracks/chr1.txt) | [2.53](http://genome.ucsc.edu/cgi-bin/hgTracks?db=hg17&position=chr1:146685421-146687948&hgt.customText=http://www.neurogenome.org/mcs/tracks/chr1.txt) | 64% | +2.53 | 47 | 4.65 | 20% | 1 |
| 109 | SV2B | 15 | [263.51](http://genome.ucsc.edu/cgi-bin/hgTracks?db=hg17&position=chr15:89376672-89640184&hgt.customText=http://www.neurogenome.org/mcs/tracks/chr15.txt) | [67.88](http://genome.ucsc.edu/cgi-bin/hgTracks?db=hg17&position=chr15:89376672-89444548&hgt.customText=http://www.neurogenome.org/mcs/tracks/chr15.txt) | [195.1](http://genome.ucsc.edu/cgi-bin/hgTracks?db=hg17&position=chr15:89444549-89639652&hgt.customText=http://www.neurogenome.org/mcs/tracks/chr15.txt) | [0.53](http://genome.ucsc.edu/cgi-bin/hgTracks?db=hg17&position=chr15:89639653-89640184&hgt.customText=http://www.neurogenome.org/mcs/tracks/chr15.txt) | 74% | +0.53 | 153 | 10.4 | 4% | 1 |
| 110 | SV2C | 5 | [643.81](http://genome.ucsc.edu/cgi-bin/hgTracks?db=hg17&position=chr5:75064449-75708255&hgt.customText=http://www.neurogenome.org/mcs/tracks/chr5.txt) | [350.61](http://genome.ucsc.edu/cgi-bin/hgTracks?db=hg17&position=chr5:75064449-75415060&hgt.customText=http://www.neurogenome.org/mcs/tracks/chr5.txt) | [246.59](http://genome.ucsc.edu/cgi-bin/hgTracks?db=hg17&position=chr5:75415061-75661645&hgt.customText=http://www.neurogenome.org/mcs/tracks/chr5.txt) | [46.61](http://genome.ucsc.edu/cgi-bin/hgTracks?db=hg17&position=chr5:75661646-75708255&hgt.customText=http://www.neurogenome.org/mcs/tracks/chr5.txt) | 38% | +46.61 | 246 | 20.41 | 3% | 6 |
| 111 | SVOP | 12 | [155.95](http://genome.ucsc.edu/cgi-bin/hgTracks?db=hg17&position=chr12:107797164-107953110&hgt.customText=http://www.neurogenome.org/mcs/tracks/chr12.txt) | [31.55](http://genome.ucsc.edu/cgi-bin/hgTracks?db=hg17&position=chr12:107921566-107953110&hgt.customText=http://www.neurogenome.org/mcs/tracks/chr12.txt) | [114.44](http://genome.ucsc.edu/cgi-bin/hgTracks?db=hg17&position=chr12:107807124-107921565&hgt.customText=http://www.neurogenome.org/mcs/tracks/chr12.txt) | [9.96](http://genome.ucsc.edu/cgi-bin/hgTracks?db=hg17&position=chr12:107797164-107807123&hgt.customText=http://www.neurogenome.org/mcs/tracks/chr12.txt) | 73% | +9.96 | 33 | 1.87 | 1% | 0 |
| 112 | SYN1 | X | [52.32](http://genome.ucsc.edu/cgi-bin/hgTracks?db=hg17&position=chrX:47187558-47239878&hgt.customText=http://www.neurogenome.org/mcs/tracks/chrX.txt) | [4.37](http://genome.ucsc.edu/cgi-bin/hgTracks?db=hg17&position=chrX:47235512-47239878&hgt.customText=http://www.neurogenome.org/mcs/tracks/chrX.txt) | [47.95](http://genome.ucsc.edu/cgi-bin/hgTracks?db=hg17&position=chrX:47187558-47235511&hgt.customText=http://www.neurogenome.org/mcs/tracks/chrX.txt) |  | 92% | -4.37 | 76 | 6.19 | 12% | 2 |
| 113 | SYN2 | 3 | [441](http://genome.ucsc.edu/cgi-bin/hgTracks?db=hg17&position=chr3:11863356-12304358&hgt.customText=http://www.neurogenome.org/mcs/tracks/chr3.txt) | [157.51](http://genome.ucsc.edu/cgi-bin/hgTracks?db=hg17&position=chr3:11863356-12020864&hgt.customText=http://www.neurogenome.org/mcs/tracks/chr3.txt) | [187.02](http://genome.ucsc.edu/cgi-bin/hgTracks?db=hg17&position=chr3:12020865-12207885&hgt.customText=http://www.neurogenome.org/mcs/tracks/chr3.txt) | [96.47](http://genome.ucsc.edu/cgi-bin/hgTracks?db=hg17&position=chr3:12207886-12304358&hgt.customText=http://www.neurogenome.org/mcs/tracks/chr3.txt) | 42% | +96.47 | 372 | 29.26 | 7% | 5 |
| 114 | SYN3 | 22 | [638.24](http://genome.ucsc.edu/cgi-bin/hgTracks?db=hg17&position=chr22:31219372-31857609&hgt.customText=http://www.neurogenome.org/mcs/tracks/chr22.txt) | [78.73](http://genome.ucsc.edu/cgi-bin/hgTracks?db=hg17&position=chr22:31778884-31857609&hgt.customText=http://www.neurogenome.org/mcs/tracks/chr22.txt) | [545.79](http://genome.ucsc.edu/cgi-bin/hgTracks?db=hg17&position=chr22:31233094-31778883&hgt.customText=http://www.neurogenome.org/mcs/tracks/chr22.txt) | [13.72](http://genome.ucsc.edu/cgi-bin/hgTracks?db=hg17&position=chr22:31219372-31233093&hgt.customText=http://www.neurogenome.org/mcs/tracks/chr22.txt) | 86% | +13.72 | 378 | 30.8 | 5% | 5 |
| 115 | SYNGR1 | 22 | [79.35](http://genome.ucsc.edu/cgi-bin/hgTracks?db=hg17&position=chr22:38040897-38120245&hgt.customText=http://www.neurogenome.org/mcs/tracks/chr22.txt) | [29.56](http://genome.ucsc.edu/cgi-bin/hgTracks?db=hg17&position=chr22:38040897-38070453&hgt.customText=http://www.neurogenome.org/mcs/tracks/chr22.txt) | [35.63](http://genome.ucsc.edu/cgi-bin/hgTracks?db=hg17&position=chr22:38070454-38106080&hgt.customText=http://www.neurogenome.org/mcs/tracks/chr22.txt) | [14.16](http://genome.ucsc.edu/cgi-bin/hgTracks?db=hg17&position=chr22:38106081-38120245&hgt.customText=http://www.neurogenome.org/mcs/tracks/chr22.txt) | 45% | +14.16 | 37 | 2.43 | 3% | 0 |
| 116 | SYNGR2 | 17 | [7.94](http://genome.ucsc.edu/cgi-bin/hgTracks?db=hg17&position=chr17:73673832-73681775&hgt.customText=http://www.neurogenome.org/mcs/tracks/chr17.txt) | [2.43](http://genome.ucsc.edu/cgi-bin/hgTracks?db=hg17&position=chr17:73673832-73676265&hgt.customText=http://www.neurogenome.org/mcs/tracks/chr17.txt) | [4.34](http://genome.ucsc.edu/cgi-bin/hgTracks?db=hg17&position=chr17:73676266-73680604&hgt.customText=http://www.neurogenome.org/mcs/tracks/chr17.txt) | [1.17](http://genome.ucsc.edu/cgi-bin/hgTracks?db=hg17&position=chr17:73680605-73681775&hgt.customText=http://www.neurogenome.org/mcs/tracks/chr17.txt) | 55% | +1.17 | 17 | 0.52 | 7% | 0 |
| 117 | SYNGR3 | 16 | [10.02](http://genome.ucsc.edu/cgi-bin/hgTracks?db=hg17&position=chr16:1977751-1987768&hgt.customText=http://www.neurogenome.org/mcs/tracks/chr16.txt) | [2.22](http://genome.ucsc.edu/cgi-bin/hgTracks?db=hg17&position=chr16:1977751-1979968&hgt.customText=http://www.neurogenome.org/mcs/tracks/chr16.txt) | [4.31](http://genome.ucsc.edu/cgi-bin/hgTracks?db=hg17&position=chr16:1979969-1984276&hgt.customText=http://www.neurogenome.org/mcs/tracks/chr16.txt) | [3.49](http://genome.ucsc.edu/cgi-bin/hgTracks?db=hg17&position=chr16:1984277-1987768&hgt.customText=http://www.neurogenome.org/mcs/tracks/chr16.txt) | 43% | -2.22 | 20 | 1.41 | 14% | 0 |
| 118 | SYNGR4 | 19 | [18.63](http://genome.ucsc.edu/cgi-bin/hgTracks?db=hg17&position=chr19:53559010-53577638&hgt.customText=http://www.neurogenome.org/mcs/tracks/chr19.txt) | [0.46](http://genome.ucsc.edu/cgi-bin/hgTracks?db=hg17&position=chr19:53559010-53559468&hgt.customText=http://www.neurogenome.org/mcs/tracks/chr19.txt) | [11.98](http://genome.ucsc.edu/cgi-bin/hgTracks?db=hg17&position=chr19:53559469-53571446&hgt.customText=http://www.neurogenome.org/mcs/tracks/chr19.txt) | [6.19](http://genome.ucsc.edu/cgi-bin/hgTracks?db=hg17&position=chr19:53571447-53577638&hgt.customText=http://www.neurogenome.org/mcs/tracks/chr19.txt) | 64% | -0.46 | 25 | 1.21 | 6% | 0 |
| 119 | SYP | X | [18.7](http://genome.ucsc.edu/cgi-bin/hgTracks?db=hg17&position=chrX:48799194-48817893&hgt.customText=http://www.neurogenome.org/mcs/tracks/chrX.txt) | [4.86](http://genome.ucsc.edu/cgi-bin/hgTracks?db=hg17&position=chrX:48813033-48817893&hgt.customText=http://www.neurogenome.org/mcs/tracks/chrX.txt) | [12.4](http://genome.ucsc.edu/cgi-bin/hgTracks?db=hg17&position=chrX:48800636-48813032&hgt.customText=http://www.neurogenome.org/mcs/tracks/chrX.txt) | [1.44](http://genome.ucsc.edu/cgi-bin/hgTracks?db=hg17&position=chrX:48799194-48800635&hgt.customText=http://www.neurogenome.org/mcs/tracks/chrX.txt) | 66% | +1.44 | 42 | 3.62 | 19% | 0 |
| 120 | SYT1 | 12 | [1345.79](http://genome.ucsc.edu/cgi-bin/hgTracks?db=hg17&position=chr12:77109257-78455050&hgt.customText=http://www.neurogenome.org/mcs/tracks/chr12.txt) | [650.98](http://genome.ucsc.edu/cgi-bin/hgTracks?db=hg17&position=chr12:77109257-77760240&hgt.customText=http://www.neurogenome.org/mcs/tracks/chr12.txt) | [588.01](http://genome.ucsc.edu/cgi-bin/hgTracks?db=hg17&position=chr12:77760241-78348254&hgt.customText=http://www.neurogenome.org/mcs/tracks/chr12.txt) | [106.8](http://genome.ucsc.edu/cgi-bin/hgTracks?db=hg17&position=chr12:78348255-78455050&hgt.customText=http://www.neurogenome.org/mcs/tracks/chr12.txt) | 44% | +106.8 | 820 | 70.26 | 5% | 10 |
| 121 | SYT10 | 12 | [1125.44](http://genome.ucsc.edu/cgi-bin/hgTracks?db=hg17&position=chr12:32941042-34066482&hgt.customText=http://www.neurogenome.org/mcs/tracks/chr12.txt) | [582.46](http://genome.ucsc.edu/cgi-bin/hgTracks?db=hg17&position=chr12:33484022-34066482&hgt.customText=http://www.neurogenome.org/mcs/tracks/chr12.txt) | [64.41](http://genome.ucsc.edu/cgi-bin/hgTracks?db=hg17&position=chr12:33419615-33484021&hgt.customText=http://www.neurogenome.org/mcs/tracks/chr12.txt) | [478.57](http://genome.ucsc.edu/cgi-bin/hgTracks?db=hg17&position=chr12:32941042-33419614&hgt.customText=http://www.neurogenome.org/mcs/tracks/chr12.txt) | 6% | +478.57 | 162 | 14.04 | 1% | 1 |
| 122 | SYT11 | 1 | [42.88](http://genome.ucsc.edu/cgi-bin/hgTracks?db=hg17&position=chr1:152640082-152682962&hgt.customText=http://www.neurogenome.org/mcs/tracks/chr1.txt) | [2.29](http://genome.ucsc.edu/cgi-bin/hgTracks?db=hg17&position=chr1:152640082-152642372&hgt.customText=http://www.neurogenome.org/mcs/tracks/chr1.txt) | [25.54](http://genome.ucsc.edu/cgi-bin/hgTracks?db=hg17&position=chr1:152642373-152667910&hgt.customText=http://www.neurogenome.org/mcs/tracks/chr1.txt) | [15.05](http://genome.ucsc.edu/cgi-bin/hgTracks?db=hg17&position=chr1:152667911-152682962&hgt.customText=http://www.neurogenome.org/mcs/tracks/chr1.txt) | 60% | -2.29 | 35 | 3.63 | 8% | 1 |
| 123 | SYT12 | 11 | [80.6](http://genome.ucsc.edu/cgi-bin/hgTracks?db=hg17&position=chr11:66500299-66580896&hgt.customText=http://www.neurogenome.org/mcs/tracks/chr11.txt) | [30.53](http://genome.ucsc.edu/cgi-bin/hgTracks?db=hg17&position=chr11:66500299-66530824&hgt.customText=http://www.neurogenome.org/mcs/tracks/chr11.txt) | [44.08](http://genome.ucsc.edu/cgi-bin/hgTracks?db=hg17&position=chr11:66530825-66574905&hgt.customText=http://www.neurogenome.org/mcs/tracks/chr11.txt) | [5.99](http://genome.ucsc.edu/cgi-bin/hgTracks?db=hg17&position=chr11:66574906-66580896&hgt.customText=http://www.neurogenome.org/mcs/tracks/chr11.txt) | 55% | +5.99 | 52 | 3.73 | 5% | 0 |
| 124 | SYT13 | 11 | [146.04](http://genome.ucsc.edu/cgi-bin/hgTracks?db=hg17&position=chr11:45203480-45349523&hgt.customText=http://www.neurogenome.org/mcs/tracks/chr11.txt) | [85.08](http://genome.ucsc.edu/cgi-bin/hgTracks?db=hg17&position=chr11:45264447-45349523&hgt.customText=http://www.neurogenome.org/mcs/tracks/chr11.txt) | [46.02](http://genome.ucsc.edu/cgi-bin/hgTracks?db=hg17&position=chr11:45218428-45264446&hgt.customText=http://www.neurogenome.org/mcs/tracks/chr11.txt) | [14.95](http://genome.ucsc.edu/cgi-bin/hgTracks?db=hg17&position=chr11:45203480-45218427&hgt.customText=http://www.neurogenome.org/mcs/tracks/chr11.txt) | 32% | +14.95 | 89 | 9.63 | 7% | 6 |
| 125 | SYT14 | 1 | [375.28](http://genome.ucsc.edu/cgi-bin/hgTracks?db=hg17&position=chr1:206419306-206794589&hgt.customText=http://www.neurogenome.org/mcs/tracks/chr1.txt) | [80.69](http://genome.ucsc.edu/cgi-bin/hgTracks?db=hg17&position=chr1:206419306-206499990&hgt.customText=http://www.neurogenome.org/mcs/tracks/chr1.txt) | [226.03](http://genome.ucsc.edu/cgi-bin/hgTracks?db=hg17&position=chr1:206499991-206726025&hgt.customText=http://www.neurogenome.org/mcs/tracks/chr1.txt) | [68.56](http://genome.ucsc.edu/cgi-bin/hgTracks?db=hg17&position=chr1:206726026-206794589&hgt.customText=http://www.neurogenome.org/mcs/tracks/chr1.txt) | 60% | +68.56 | 158 | 14.02 | 4% | 2 |
| 126 | SYT15 | 10 | [18.89](http://genome.ucsc.edu/cgi-bin/hgTracks?db=hg17&position=chr10:46372517-46391406&hgt.customText=http://www.neurogenome.org/mcs/tracks/chr10.txt) |  | [18.89](http://genome.ucsc.edu/cgi-bin/hgTracks?db=hg17&position=chr10:46372517-46391406&hgt.customText=http://www.neurogenome.org/mcs/tracks/chr10.txt) |  | 100% | -0 | 38 | 3.51 | 19% | 1 |
| 127 | SYT16 | 14 | [321.05](http://genome.ucsc.edu/cgi-bin/hgTracks?db=hg17&position=chr14:61332899-61653949&hgt.customText=http://www.neurogenome.org/mcs/tracks/chr14.txt) | [199.4](http://genome.ucsc.edu/cgi-bin/hgTracks?db=hg17&position=chr14:61332899-61532293&hgt.customText=http://www.neurogenome.org/mcs/tracks/chr14.txt) | [105.89](http://genome.ucsc.edu/cgi-bin/hgTracks?db=hg17&position=chr14:61532294-61638180&hgt.customText=http://www.neurogenome.org/mcs/tracks/chr14.txt) | [15.77](http://genome.ucsc.edu/cgi-bin/hgTracks?db=hg17&position=chr14:61638181-61653949&hgt.customText=http://www.neurogenome.org/mcs/tracks/chr14.txt) | 33% | +15.77 | 218 | 19.44 | 6% | 4 |
| 128 | SYT17 | 16 | [169.66](http://genome.ucsc.edu/cgi-bin/hgTracks?db=hg17&position=chr16:19034944-19204601&hgt.customText=http://www.neurogenome.org/mcs/tracks/chr16.txt) | [52.2](http://genome.ucsc.edu/cgi-bin/hgTracks?db=hg17&position=chr16:19034944-19087138&hgt.customText=http://www.neurogenome.org/mcs/tracks/chr16.txt) | [98.92](http://genome.ucsc.edu/cgi-bin/hgTracks?db=hg17&position=chr16:19087139-19186055&hgt.customText=http://www.neurogenome.org/mcs/tracks/chr16.txt) | [18.55](http://genome.ucsc.edu/cgi-bin/hgTracks?db=hg17&position=chr16:19186056-19204601&hgt.customText=http://www.neurogenome.org/mcs/tracks/chr16.txt) | 58% | +18.55 | 101 | 7.9 | 5% | 1 |
| 129 | SYT2 | 1 | [132.88](http://genome.ucsc.edu/cgi-bin/hgTracks?db=hg17&position=chr1:199295305-199428188&hgt.customText=http://www.neurogenome.org/mcs/tracks/chr1.txt) | [16.99](http://genome.ucsc.edu/cgi-bin/hgTracks?db=hg17&position=chr1:199411203-199428188&hgt.customText=http://www.neurogenome.org/mcs/tracks/chr1.txt) | [114.66](http://genome.ucsc.edu/cgi-bin/hgTracks?db=hg17&position=chr1:199296545-199411202&hgt.customText=http://www.neurogenome.org/mcs/tracks/chr1.txt) | [1.24](http://genome.ucsc.edu/cgi-bin/hgTracks?db=hg17&position=chr1:199295305-199296544&hgt.customText=http://www.neurogenome.org/mcs/tracks/chr1.txt) | 86% | +1.24 | 82 | 5.77 | 4% | 0 |
| 130 | SYT3 | 19 | [110.33](http://genome.ucsc.edu/cgi-bin/hgTracks?db=hg17&position=chr19:55753133-55863466&hgt.customText=http://www.neurogenome.org/mcs/tracks/chr19.txt) |  | [94.96](http://genome.ucsc.edu/cgi-bin/hgTracks?db=hg17&position=chr19:55817048-55912007&hgt.customText=http://www.neurogenome.org/mcs/tracks/chr19.txt) | [63.91](http://genome.ucsc.edu/cgi-bin/hgTracks?db=hg17&position=chr19:55753133-55817047&hgt.customText=http://www.neurogenome.org/mcs/tracks/chr19.txt) | 86% | +63.91 | 87 | 8.2 | 7% | 2 |
| 131 | SYT4 | 18 | [358.8](http://genome.ucsc.edu/cgi-bin/hgTracks?db=hg17&position=chr18:38974340-39333135&hgt.customText=http://www.neurogenome.org/mcs/tracks/chr18.txt) | [221.71](http://genome.ucsc.edu/cgi-bin/hgTracks?db=hg17&position=chr18:39111431-39333135&hgt.customText=http://www.neurogenome.org/mcs/tracks/chr18.txt) | [9.58](http://genome.ucsc.edu/cgi-bin/hgTracks?db=hg17&position=chr18:39101855-39111430&hgt.customText=http://www.neurogenome.org/mcs/tracks/chr18.txt) | [127.52](http://genome.ucsc.edu/cgi-bin/hgTracks?db=hg17&position=chr18:38974340-39101854&hgt.customText=http://www.neurogenome.org/mcs/tracks/chr18.txt) | 3% | +127.52 | 86 | 8.03 | 2% | 2 |
| 132 | SYT5 | 19 | [13.63](http://genome.ucsc.edu/cgi-bin/hgTracks?db=hg17&position=chr19:60369831-60383458&hgt.customText=http://www.neurogenome.org/mcs/tracks/chr19.txt) |  | [7.18](http://genome.ucsc.edu/cgi-bin/hgTracks?db=hg17&position=chr19:60376276-60383458&hgt.customText=http://www.neurogenome.org/mcs/tracks/chr19.txt) | [6.45](http://genome.ucsc.edu/cgi-bin/hgTracks?db=hg17&position=chr19:60369831-60376275&hgt.customText=http://www.neurogenome.org/mcs/tracks/chr19.txt) | 53% | +6.45 | 26 | 1.74 | 13% | 0 |
| 133 | SYT6 | 1 | [410.52](http://genome.ucsc.edu/cgi-bin/hgTracks?db=hg17&position=chr1:114236918-114647442&hgt.customText=http://www.neurogenome.org/mcs/tracks/chr1.txt) | [238.93](http://genome.ucsc.edu/cgi-bin/hgTracks?db=hg17&position=chr1:114408515-114647442&hgt.customText=http://www.neurogenome.org/mcs/tracks/chr1.txt) | [62.09](http://genome.ucsc.edu/cgi-bin/hgTracks?db=hg17&position=chr1:114346429-114408514&hgt.customText=http://www.neurogenome.org/mcs/tracks/chr1.txt) | [109.51](http://genome.ucsc.edu/cgi-bin/hgTracks?db=hg17&position=chr1:114236918-114346428&hgt.customText=http://www.neurogenome.org/mcs/tracks/chr1.txt) | 15% | +109.51 | 351 | 26.31 | 6% | 2 |
| 134 | SYT7 | 11 | [134.06](http://genome.ucsc.edu/cgi-bin/hgTracks?db=hg17&position=chr11:61014976-61149032&hgt.customText=http://www.neurogenome.org/mcs/tracks/chr11.txt) | [44.16](http://genome.ucsc.edu/cgi-bin/hgTracks?db=hg17&position=chr11:61104875-61149032&hgt.customText=http://www.neurogenome.org/mcs/tracks/chr11.txt) | [65.51](http://genome.ucsc.edu/cgi-bin/hgTracks?db=hg17&position=chr11:61039362-61104874&hgt.customText=http://www.neurogenome.org/mcs/tracks/chr11.txt) | [24.39](http://genome.ucsc.edu/cgi-bin/hgTracks?db=hg17&position=chr11:61014976-61039361&hgt.customText=http://www.neurogenome.org/mcs/tracks/chr11.txt) | 49% | +24.39 | 227 | 22.44 | 17% | 6 |
| 135 | SYT8 | 11 | [35.38](http://genome.ucsc.edu/cgi-bin/hgTracks?db=hg17&position=chr11:1781911-1817294&hgt.customText=http://www.neurogenome.org/mcs/tracks/chr11.txt) | [26.98](http://genome.ucsc.edu/cgi-bin/hgTracks?db=hg17&position=chr11:1781911-1808892&hgt.customText=http://www.neurogenome.org/mcs/tracks/chr11.txt) | [6.43](http://genome.ucsc.edu/cgi-bin/hgTracks?db=hg17&position=chr11:1808893-1815326&hgt.customText=http://www.neurogenome.org/mcs/tracks/chr11.txt) | [1.97](http://genome.ucsc.edu/cgi-bin/hgTracks?db=hg17&position=chr11:1815327-1817294&hgt.customText=http://www.neurogenome.org/mcs/tracks/chr11.txt) | 18% | +1.97 | 43 | 1.9 | 5% | 0 |
| 136 | SYT9 | 11 | [394.33](http://genome.ucsc.edu/cgi-bin/hgTracks?db=hg17&position=chr11:7068956-7463288&hgt.customText=http://www.neurogenome.org/mcs/tracks/chr11.txt) | [147.63](http://genome.ucsc.edu/cgi-bin/hgTracks?db=hg17&position=chr11:7068956-7216584&hgt.customText=http://www.neurogenome.org/mcs/tracks/chr11.txt) | [230.26](http://genome.ucsc.edu/cgi-bin/hgTracks?db=hg17&position=chr11:7216585-7446844&hgt.customText=http://www.neurogenome.org/mcs/tracks/chr11.txt) | [16.44](http://genome.ucsc.edu/cgi-bin/hgTracks?db=hg17&position=chr11:7446845-7463288&hgt.customText=http://www.neurogenome.org/mcs/tracks/chr11.txt) | 58% | +16.44 | 245 | 18.83 | 5% | 3 |
| 137 | SYTL1 | 1 | [18.78](http://genome.ucsc.edu/cgi-bin/hgTracks?db=hg17&position=chr1:27347033-27365811&hgt.customText=http://www.neurogenome.org/mcs/tracks/chr1.txt) | [5.62](http://genome.ucsc.edu/cgi-bin/hgTracks?db=hg17&position=chr1:27347033-27352654&hgt.customText=http://www.neurogenome.org/mcs/tracks/chr1.txt) | [11.91](http://genome.ucsc.edu/cgi-bin/hgTracks?db=hg17&position=chr1:27352655-27364563&hgt.customText=http://www.neurogenome.org/mcs/tracks/chr1.txt) | [1.25](http://genome.ucsc.edu/cgi-bin/hgTracks?db=hg17&position=chr1:27364564-27365811&hgt.customText=http://www.neurogenome.org/mcs/tracks/chr1.txt) | 63% | +1.25 | 29 | 2.19 | 12% | 0 |
| 138 | SYTL2 | 11 | [166.28](http://genome.ucsc.edu/cgi-bin/hgTracks?db=hg17&position=chr11:85074969-85241247&hgt.customText=http://www.neurogenome.org/mcs/tracks/chr11.txt) | [94.56](http://genome.ucsc.edu/cgi-bin/hgTracks?db=hg17&position=chr11:85146693-85241247&hgt.customText=http://www.neurogenome.org/mcs/tracks/chr11.txt) | [63.78](http://genome.ucsc.edu/cgi-bin/hgTracks?db=hg17&position=chr11:85082912-85146692&hgt.customText=http://www.neurogenome.org/mcs/tracks/chr11.txt) | [7.94](http://genome.ucsc.edu/cgi-bin/hgTracks?db=hg17&position=chr11:85074969-85082911&hgt.customText=http://www.neurogenome.org/mcs/tracks/chr11.txt) | 38% | +7.94 | 156 | 10.43 | 6% | 1 |
| 139 | SYTL3 | 6 | [121.01](http://genome.ucsc.edu/cgi-bin/hgTracks?db=hg17&position=chr6:159036181-159157186&hgt.customText=http://www.neurogenome.org/mcs/tracks/chr6.txt) | [5.33](http://genome.ucsc.edu/cgi-bin/hgTracks?db=hg17&position=chr6:159036181-159041508&hgt.customText=http://www.neurogenome.org/mcs/tracks/chr6.txt) | [114.8](http://genome.ucsc.edu/cgi-bin/hgTracks?db=hg17&position=chr6:159041509-159156312&hgt.customText=http://www.neurogenome.org/mcs/tracks/chr6.txt) | [0.87](http://genome.ucsc.edu/cgi-bin/hgTracks?db=hg17&position=chr6:159156313-159157186&hgt.customText=http://www.neurogenome.org/mcs/tracks/chr6.txt) | 95% | +0.87 | 72 | 3.16 | 3% | 0 |
| 140 | SYTL4 | X | [149.06](http://genome.ucsc.edu/cgi-bin/hgTracks?db=hg17&position=chrX:99732437-99881492&hgt.customText=http://www.neurogenome.org/mcs/tracks/chrX.txt) | [88.24](http://genome.ucsc.edu/cgi-bin/hgTracks?db=hg17&position=chrX:99793254-99881492&hgt.customText=http://www.neurogenome.org/mcs/tracks/chrX.txt) | [57.62](http://genome.ucsc.edu/cgi-bin/hgTracks?db=hg17&position=chrX:99735634-99793253&hgt.customText=http://www.neurogenome.org/mcs/tracks/chrX.txt) | [3.2](http://genome.ucsc.edu/cgi-bin/hgTracks?db=hg17&position=chrX:99732437-99735633&hgt.customText=http://www.neurogenome.org/mcs/tracks/chrX.txt) | 39% | +3.2 | 121 | 10.74 | 7% | 1 |
| 141 | UNC13A | 19 | [105.05](http://genome.ucsc.edu/cgi-bin/hgTracks?db=hg17&position=chr19:17554968-17660014&hgt.customText=http://www.neurogenome.org/mcs/tracks/chr19.txt) | [42.2](http://genome.ucsc.edu/cgi-bin/hgTracks?db=hg17&position=chr19:17617816-17660014&hgt.customText=http://www.neurogenome.org/mcs/tracks/chr19.txt) | [42.19](http://genome.ucsc.edu/cgi-bin/hgTracks?db=hg17&position=chr19:17575622-17617815&hgt.customText=http://www.neurogenome.org/mcs/tracks/chr19.txt) | [20.65](http://genome.ucsc.edu/cgi-bin/hgTracks?db=hg17&position=chr19:17554968-17575621&hgt.customText=http://www.neurogenome.org/mcs/tracks/chr19.txt) | 40% | +20.65 | 87 | 8.48 | 8% | 1 |
| 142 | UNC13B | 9 | [256.16](http://genome.ucsc.edu/cgi-bin/hgTracks?db=hg17&position=chr9:35139170-35395331&hgt.customText=http://www.neurogenome.org/mcs/tracks/chr9.txt) | [12.88](http://genome.ucsc.edu/cgi-bin/hgTracks?db=hg17&position=chr9:35139170-35152054&hgt.customText=http://www.neurogenome.org/mcs/tracks/chr9.txt) | [243.28](http://genome.ucsc.edu/cgi-bin/hgTracks?db=hg17&position=chr9:35152055-35395331&hgt.customText=http://www.neurogenome.org/mcs/tracks/chr9.txt) |  | 95% | -12.88 | 270 | 20.06 | 8% | 2 |
| 143 | UNC13C | 15 | [1266.6](http://genome.ucsc.edu/cgi-bin/hgTracks?db=hg17&position=chr15:51994202-53260804&hgt.customText=http://www.neurogenome.org/mcs/tracks/chr15.txt) | [179.18](http://genome.ucsc.edu/cgi-bin/hgTracks?db=hg17&position=chr15:51994202-52173378&hgt.customText=http://www.neurogenome.org/mcs/tracks/chr15.txt) | [534.72](http://genome.ucsc.edu/cgi-bin/hgTracks?db=hg17&position=chr15:52173379-52708094&hgt.customText=http://www.neurogenome.org/mcs/tracks/chr15.txt) | [552.71](http://genome.ucsc.edu/cgi-bin/hgTracks?db=hg17&position=chr15:52708095-53260804&hgt.customText=http://www.neurogenome.org/mcs/tracks/chr15.txt) | 42% | -179.18 | 325 | 24.1 | 2% | 3 |
| 144 | UNC13D | 17 | [19.9](http://genome.ucsc.edu/cgi-bin/hgTracks?db=hg17&position=chr17:71333475-71353375&hgt.customText=http://www.neurogenome.org/mcs/tracks/chr17.txt) | [0.98](http://genome.ucsc.edu/cgi-bin/hgTracks?db=hg17&position=chr17:71352394-71353375&hgt.customText=http://www.neurogenome.org/mcs/tracks/chr17.txt) | [17.46](http://genome.ucsc.edu/cgi-bin/hgTracks?db=hg17&position=chr17:71334934-71352393&hgt.customText=http://www.neurogenome.org/mcs/tracks/chr17.txt) | [1.46](http://genome.ucsc.edu/cgi-bin/hgTracks?db=hg17&position=chr17:71333475-71334933&hgt.customText=http://www.neurogenome.org/mcs/tracks/chr17.txt) | 88% | -0.98 | 47 | 3.48 | 17% | 0 |
| 145 | VAMP1 | 12 | [24.61](http://genome.ucsc.edu/cgi-bin/hgTracks?db=hg17&position=chr12:6425495-6450104&hgt.customText=http://www.neurogenome.org/mcs/tracks/chr12.txt) |  | [24.61](http://genome.ucsc.edu/cgi-bin/hgTracks?db=hg17&position=chr12:6425495-6450104&hgt.customText=http://www.neurogenome.org/mcs/tracks/chr12.txt) |  | 100% | -0 | 45 | 3.23 | 13% | 0 |
| 146 | VAMP2 | 17 | [31.04](http://genome.ucsc.edu/cgi-bin/hgTracks?db=hg17&position=chr17:7986242-8017284&hgt.customText=http://www.neurogenome.org/mcs/tracks/chr17.txt) | [9.7](http://genome.ucsc.edu/cgi-bin/hgTracks?db=hg17&position=chr17:8007590-8017284&hgt.customText=http://www.neurogenome.org/mcs/tracks/chr17.txt) | [23.07](http://genome.ucsc.edu/cgi-bin/hgTracks?db=hg17&position=chr17:7984515-8007589&hgt.customText=http://www.neurogenome.org/mcs/tracks/chr17.txt) |  | 74% | -9.7 | 85 | 8.3 | 27% | 1 |
| 147 | VAMP3 | 1 | [22.44](http://genome.ucsc.edu/cgi-bin/hgTracks?db=hg17&position=chr1:7764030-7786465&hgt.customText=http://www.neurogenome.org/mcs/tracks/chr1.txt) | [1.56](http://genome.ucsc.edu/cgi-bin/hgTracks?db=hg17&position=chr1:7764030-7765594&hgt.customText=http://www.neurogenome.org/mcs/tracks/chr1.txt) | [73.91](http://genome.ucsc.edu/cgi-bin/hgTracks?db=hg17&position=chr1:7765595-7839503&hgt.customText=http://www.neurogenome.org/mcs/tracks/chr1.txt) |  | 329% | -1.56 | 27 | 1.47 | 7% | 0 |
| 148 | VAMP4 | 1 | [109.36](http://genome.ucsc.edu/cgi-bin/hgTracks?db=hg17&position=chr1:168353509-168462870&hgt.customText=http://www.neurogenome.org/mcs/tracks/chr1.txt) | [20](http://genome.ucsc.edu/cgi-bin/hgTracks?db=hg17&position=chr1:168442872-168462870&hgt.customText=http://www.neurogenome.org/mcs/tracks/chr1.txt) | [41.91](http://genome.ucsc.edu/cgi-bin/hgTracks?db=hg17&position=chr1:168400962-168442871&hgt.customText=http://www.neurogenome.org/mcs/tracks/chr1.txt) | [47.45](http://genome.ucsc.edu/cgi-bin/hgTracks?db=hg17&position=chr1:168353509-168400961&hgt.customText=http://www.neurogenome.org/mcs/tracks/chr1.txt) | 38% | -20 | 60 | 5.1 | 5% | 2 |
| 149 | VAMP5 | 2 | [13.68](http://genome.ucsc.edu/cgi-bin/hgTracks?db=hg17&position=chr2:85720812-85734494&hgt.customText=http://www.neurogenome.org/mcs/tracks/chr2.txt) | [2.38](http://genome.ucsc.edu/cgi-bin/hgTracks?db=hg17&position=chr2:85720812-85723188&hgt.customText=http://www.neurogenome.org/mcs/tracks/chr2.txt) | [8.98](http://genome.ucsc.edu/cgi-bin/hgTracks?db=hg17&position=chr2:85723189-85732169&hgt.customText=http://www.neurogenome.org/mcs/tracks/chr2.txt) | [2.33](http://genome.ucsc.edu/cgi-bin/hgTracks?db=hg17&position=chr2:85732170-85734494&hgt.customText=http://www.neurogenome.org/mcs/tracks/chr2.txt) | 66% | +2.33 | 21 | 2.11 | 15% | 0 |
| 150 | VAMP8 | 2 | [22.95](http://genome.ucsc.edu/cgi-bin/hgTracks?db=hg17&position=chr2:85700238-85723188&hgt.customText=http://www.neurogenome.org/mcs/tracks/chr2.txt) | [16.14](http://genome.ucsc.edu/cgi-bin/hgTracks?db=hg17&position=chr2:85700238-85716374&hgt.customText=http://www.neurogenome.org/mcs/tracks/chr2.txt) | [4.44](http://genome.ucsc.edu/cgi-bin/hgTracks?db=hg17&position=chr2:85716375-85720811&hgt.customText=http://www.neurogenome.org/mcs/tracks/chr2.txt) | [2.38](http://genome.ucsc.edu/cgi-bin/hgTracks?db=hg17&position=chr2:85720812-85723188&hgt.customText=http://www.neurogenome.org/mcs/tracks/chr2.txt) | 19% | +2.38 | 18 | 1.15 | 5% | 0 |
