# Supplementary figures and images for: Patterns of sequence conservation in presynaptic neural genes
Source: Genome Biol. 2006 Nov 10;7(11):R105. doi: 10.1186/gb-2006-7-11-r105 (PMC1794582; doi:10.1186/gb-2006-7-11-r105)

Neighbor-joining

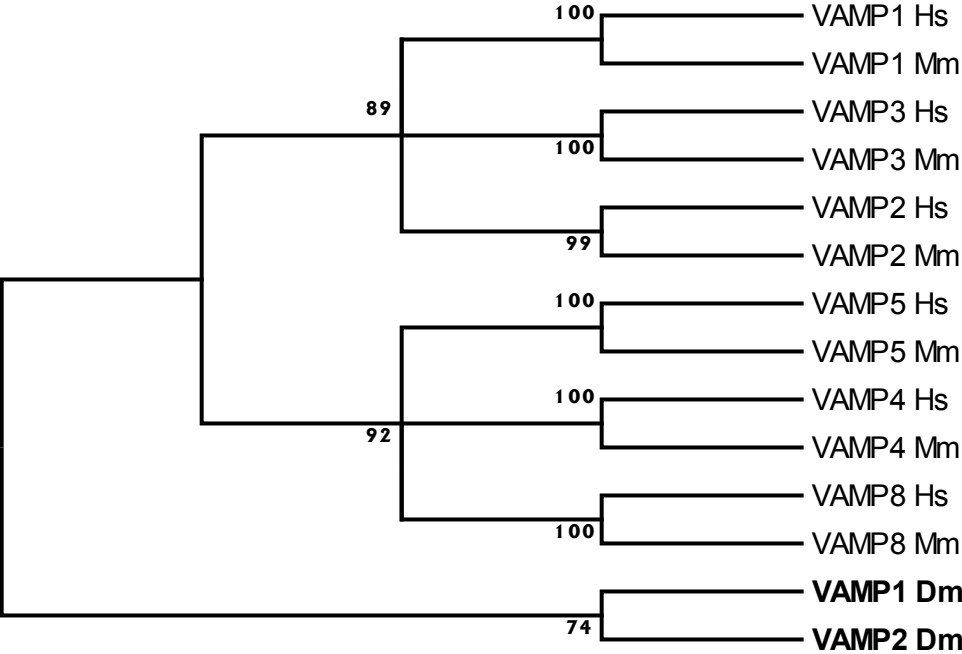

Maximum Parsimony

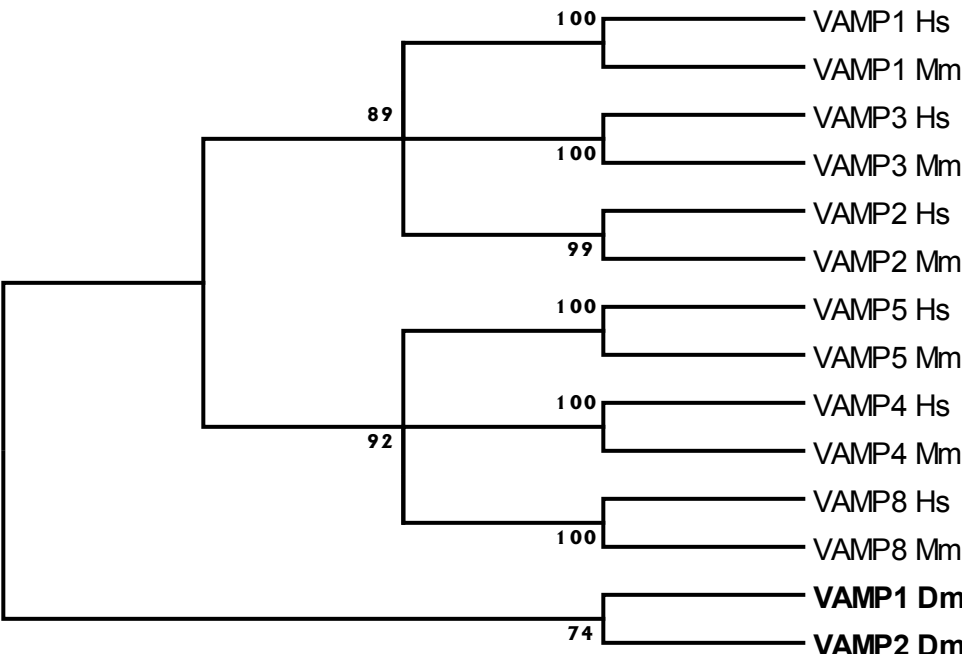

Supplement: Additional data file 2 — VAMP phylogeny [file gb-2006-7-11-r105-S2.pdf]

Neighbor-Joining

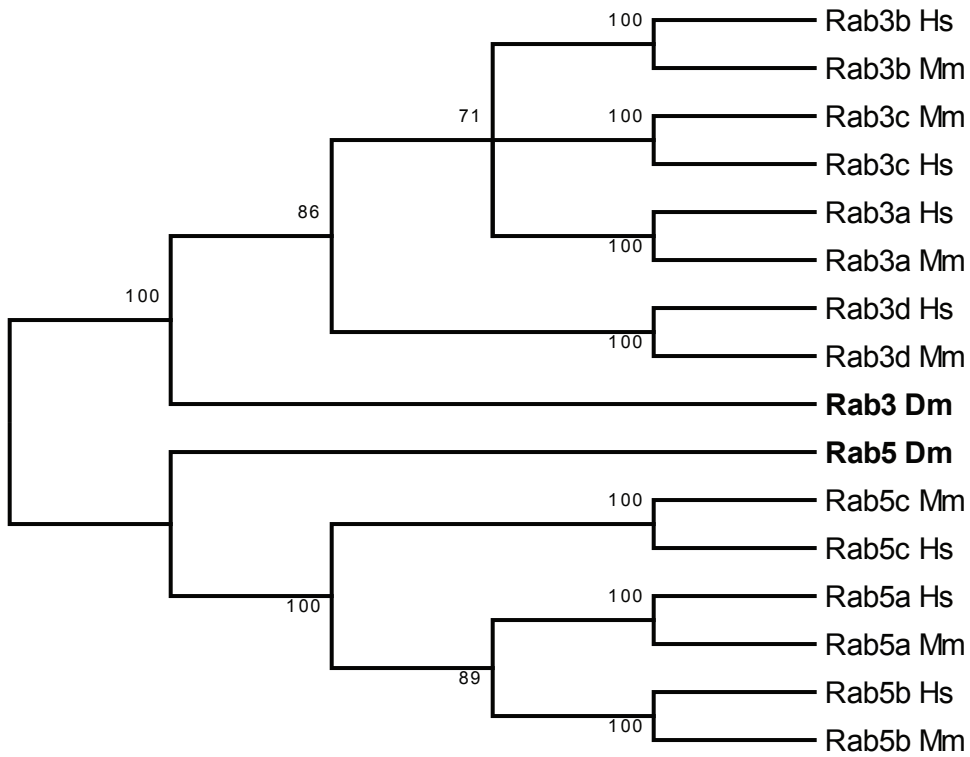

Maximum Parsimony

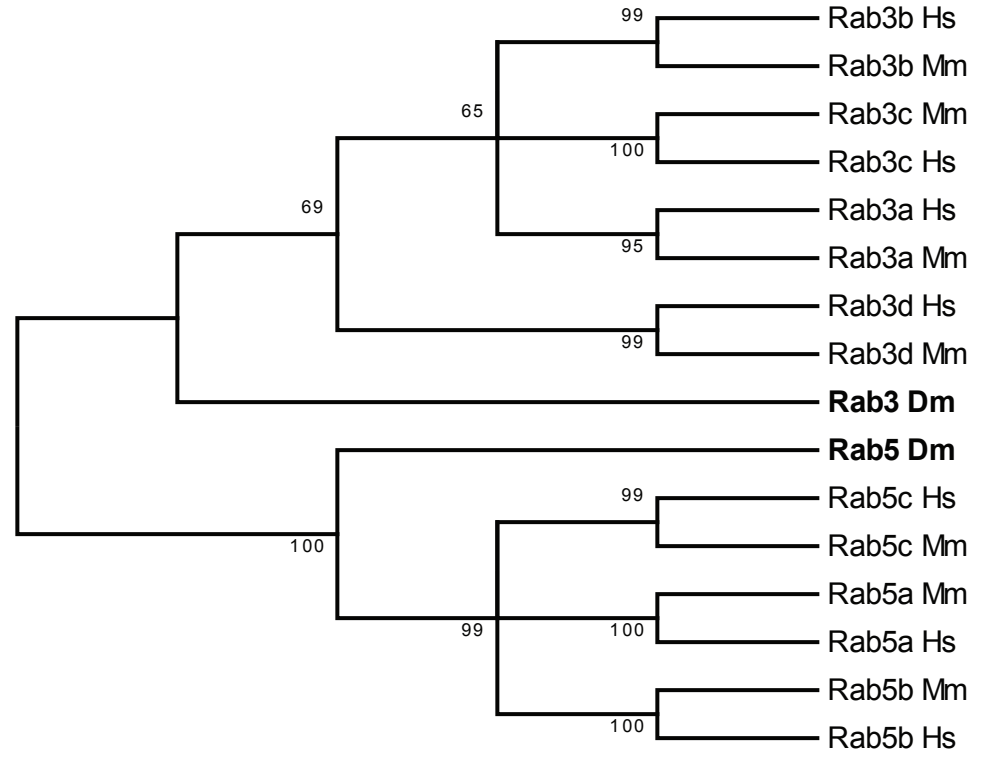

Supplement: Additional data file 3 — RAB phylogeny [file gb-2006-7-11-r105-S3.pdf]

## Neighbor-Joining

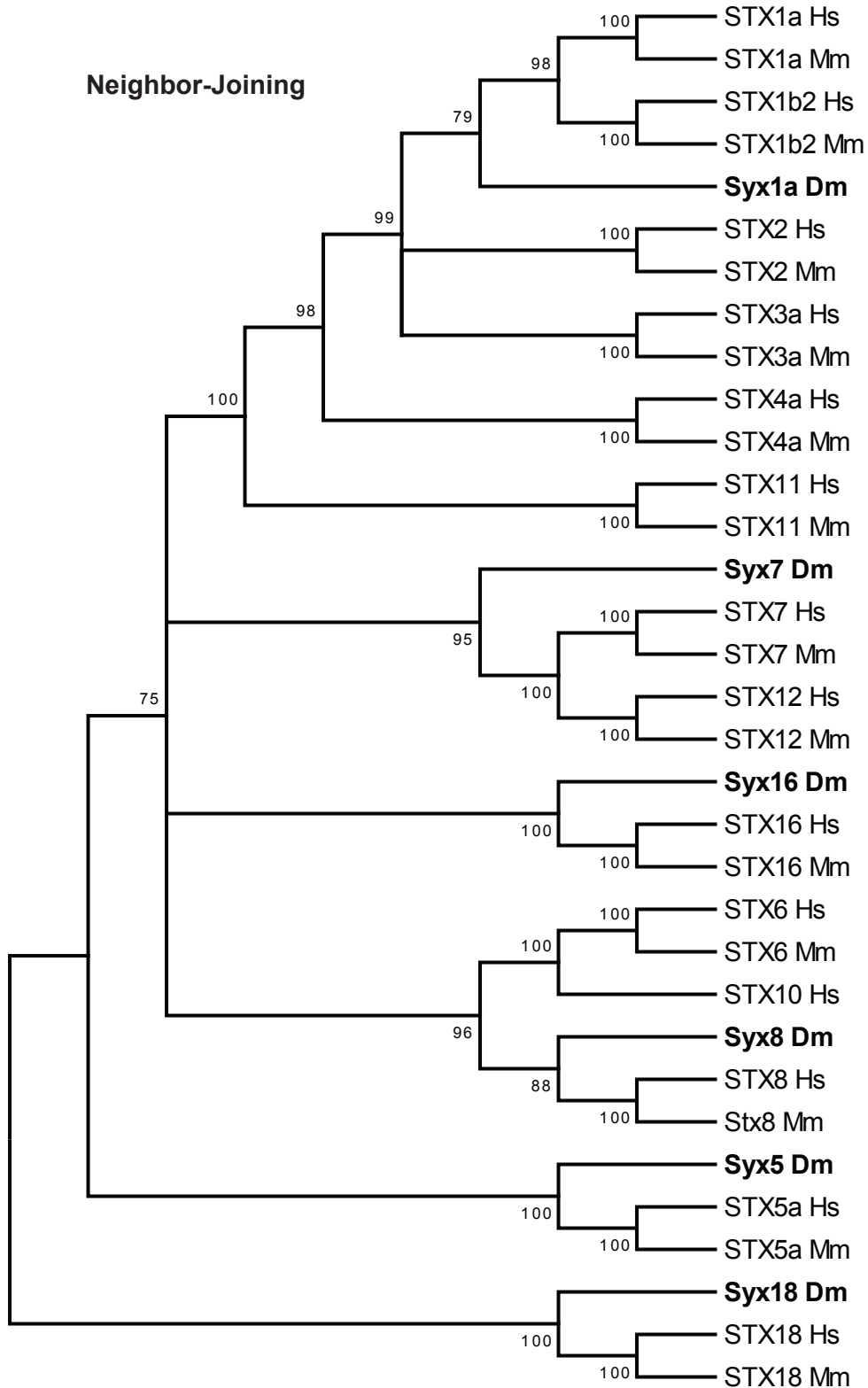

## Maximum Parsimony

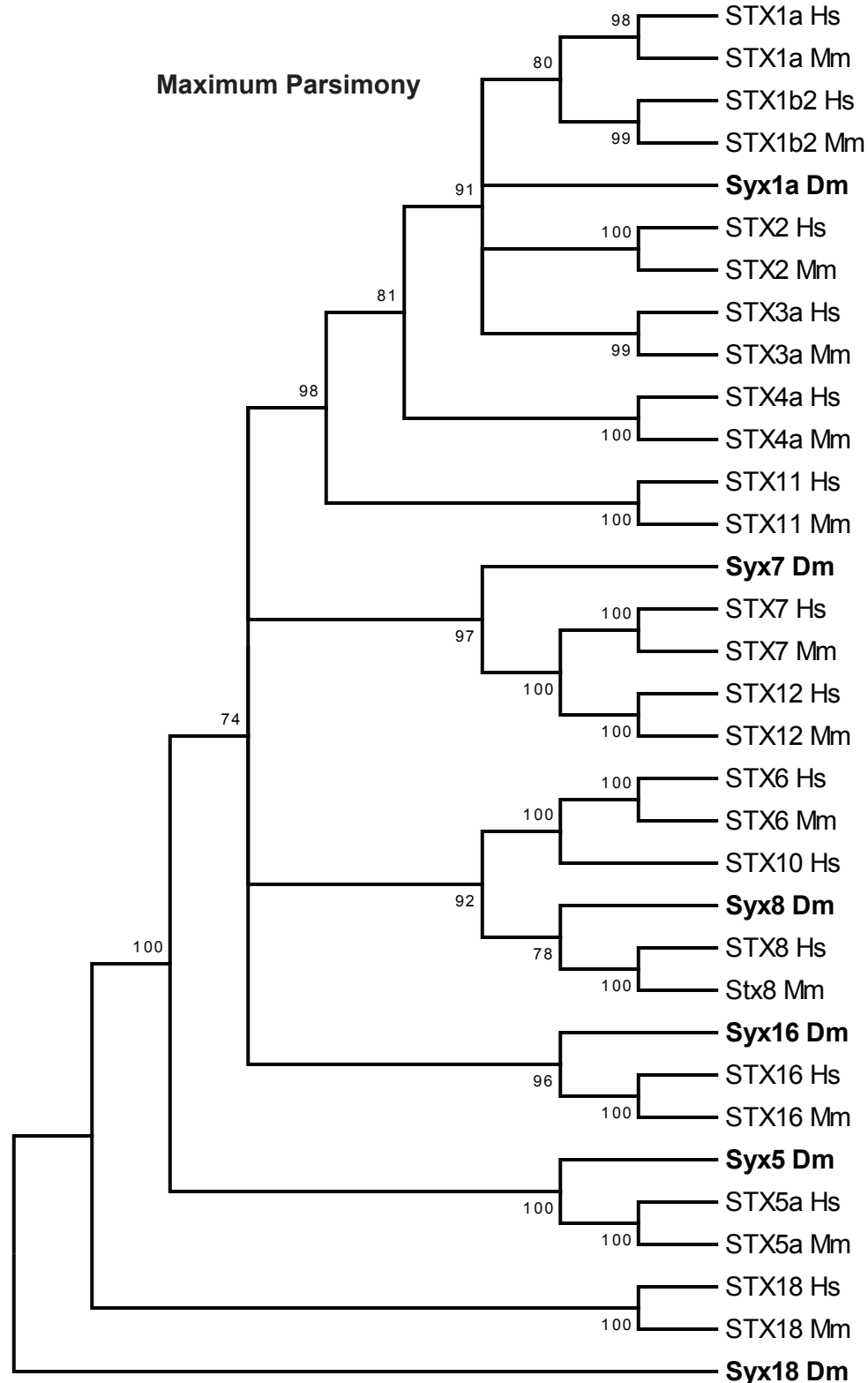

Supplement: Additional data file 4 — STX phylogeny [file gb-2006-7-11-r105-S4.pdf]

## Neighbor-Joining

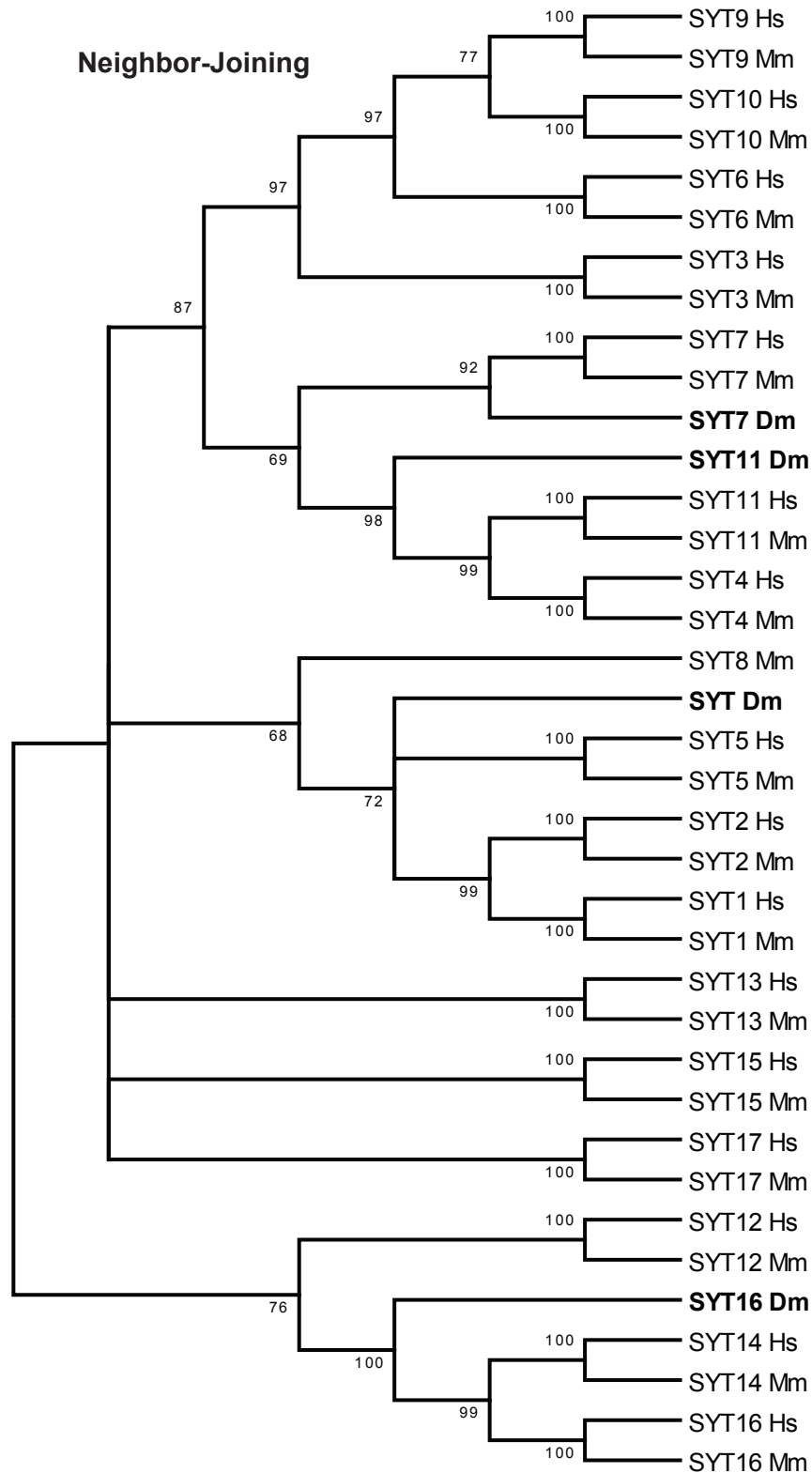

## Maximum Parsimony

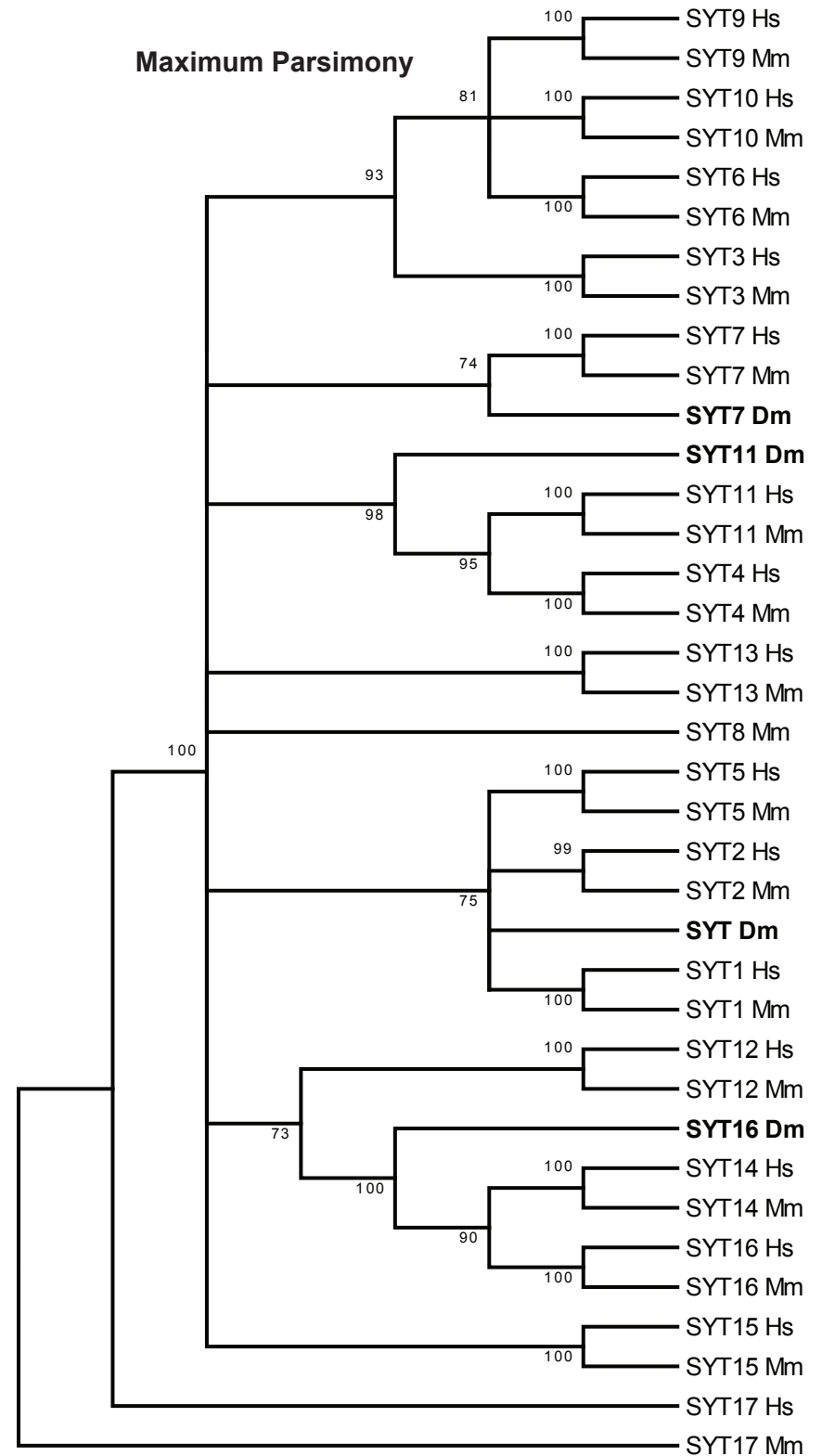

Supplement: Additional data file 5 — SYT phylogeny [file gb-2006-7-11-r105-S5.pdf]

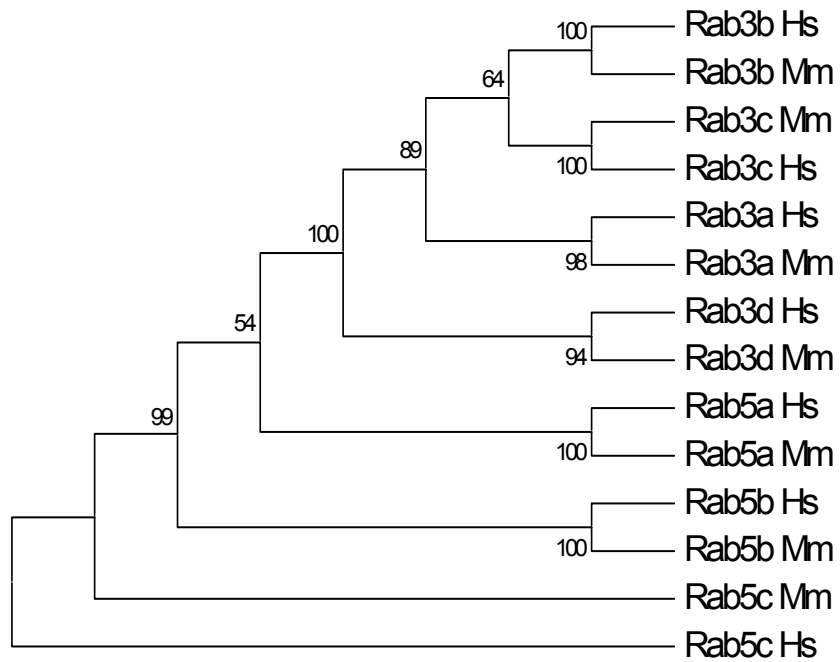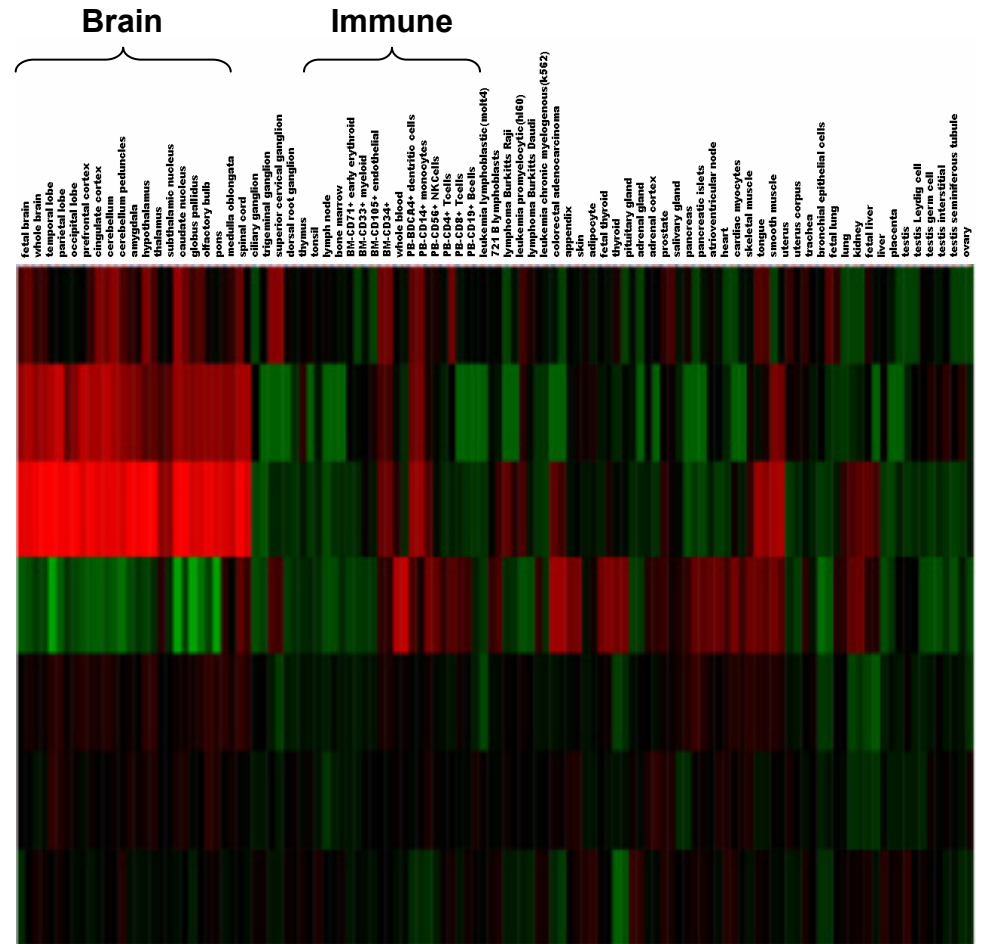

Supplement: Additional data file 6 — RAB tree with superimposed expression profiles [file gb-2006-7-11-r105-S6.pdf]

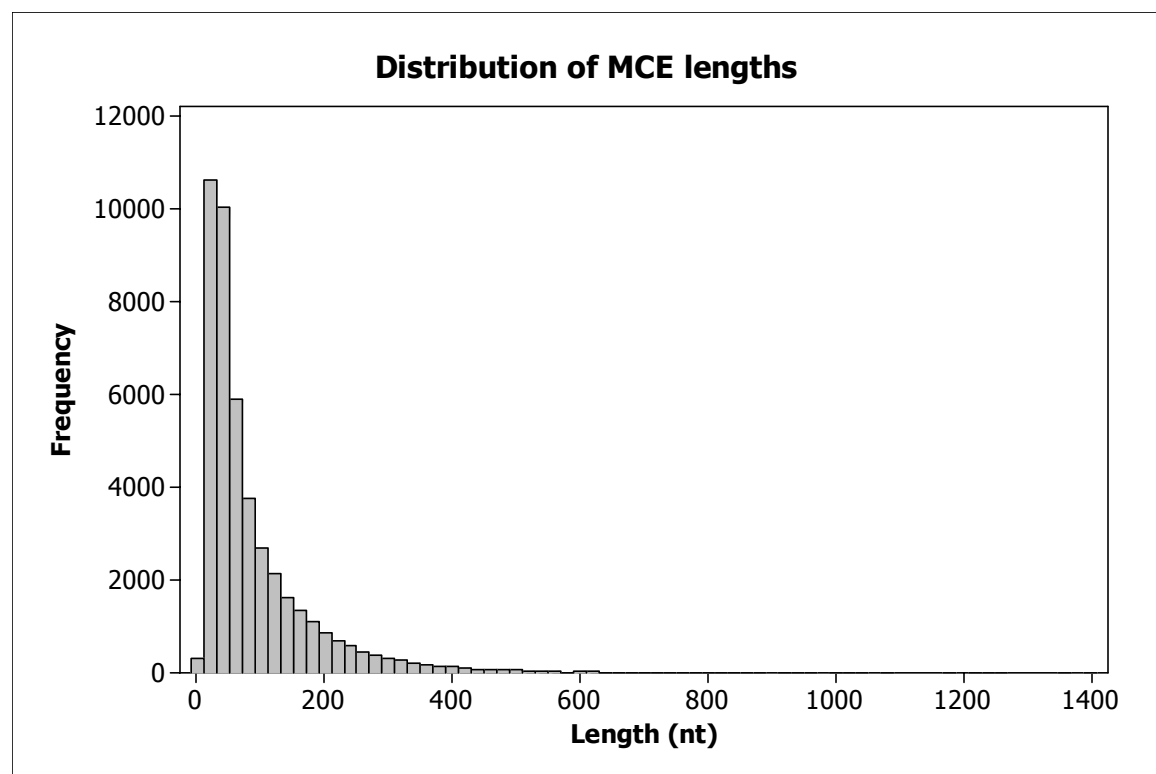

Supplement: Additional data file 7 — Distribution of most conserved element (MCE) lengths [file gb-2006-7-11-r105-S7.pdf]
