# Supplementary material for: Patterns of sequence conservation in presynaptic neural genes
Source: Genome Biol. 2006 Nov 10;7(11):R105. doi: 10.1186/gb-2006-7-11-r105 (PMC1794582; doi:10.1186/gb-2006-7-11-r105)
Supplement: Additional data file 8 — Palindromes found within most conserved element (MCE) subsequences [file gb-2006-7-11-r105-S8.doc]

# Palindromes found within MCE sub-sequences

The gene, genomic position, relative genic position (type), BLASTN score, expected value, and alignment of sequence reads in both directions are shown.

| **gene** | **position** | **type** | **score** | **expect** | **Alignment** |
| --- | --- | --- | --- | --- | --- |
| APBA1 | [chr9:69518108-69518125](http://genome.ucsc.edu/cgi-bin/hgTracks?db=hg17&position=chr9:69518108-69518125&hgt.customText=http://www.neurogenome.org/mcs/tracks/chr9.txt) | 2a (5’) | 18 | 7.23E-04 | AAAATTCATATGAATTTT  ||||||||||||||||||  AAAATTCATATGAATTTT |
| BZRAP1 | [chr17:53757173-53757190](http://genome.ucsc.edu/cgi-bin/hgTracks?db=hg17&position=chr17:53757173-53757190&hgt.customText=http://www.neurogenome.org/mcs/tracks/chr17.txt) | 1a | 18 | 1.13E-03 | CTCCCGGGGCCCCGGGAG  ||||||||||||||||||  CTCCCGGGGCCCCGGGAG |
| CAMK2N1 | [chr1:20472444-20472461](http://genome.ucsc.edu/cgi-bin/hgTracks?db=hg17&position=chr1:20472444-20472461&hgt.customText=http://www.neurogenome.org/mcs/tracks/chr1.txt) | 2a (3’) | 18 | 2.26E-03 | CACAGCCTGCAGGCTGTG  ||||||||||||||||||  CACAGCCTGCAGGCTGTG |
| CAST1 | [chr3:55644393-55644410](http://genome.ucsc.edu/cgi-bin/hgTracks?db=hg17&position=chr3:55644393-55644410&hgt.customText=http://www.neurogenome.org/mcs/tracks/chr3.txt) | 2b | 18 | 4.39E-03 | TAATGTTTATAAACATTA  ||||||||||||||||||  TAATGTTTATAAACATTA |
| EXOC4 | [chr7:132415595-132415620](http://genome.ucsc.edu/cgi-bin/hgTracks?db=hg17&position=chr7:132415595-132415620&hgt.customText=http://www.neurogenome.org/mcs/tracks/chr7.txt) | 2b | 18 | 3.49E-03 | CAAAAAGAATTTTACAATTCTTTTTG  ||||||||||| ||||||||||||||  CAAAAAGAATTGTAAAATTCTTTTTG |
| EXOC4 | [chr7:132620688-132620715](http://genome.ucsc.edu/cgi-bin/hgTracks?db=hg17&position=chr7:132620688-132620715&hgt.customText=http://www.neurogenome.org/mcs/tracks/chr7.txt) | 2b | 20 | 3.27E-04 | TTAATTAACCTTAGCTAAGGGTAATTAA  ||||||| |||||||||||| |||||||  TTAATTACCCTTAGCTAAGGTTAATTAA |
| EXOC5 | [chr14:56383802-56383821](http://genome.ucsc.edu/cgi-bin/hgTracks?db=hg17&position=chr14:56383802-56383821&hgt.customText=http://www.neurogenome.org/mcs/tracks/chr14.txt) | 2a (3’) | 20 | 1.14E-04 | TTTCTTAGGGCCCTAAGAAA  ||||||||||||||||||||  TTTCTTAGGGCCCTAAGAAA |
| NBEA | [chr13:33537136-33537153](http://genome.ucsc.edu/cgi-bin/hgTracks?db=hg17&position=chr13:33537136-33537153&hgt.customText=http://www.neurogenome.org/mcs/tracks/chr13.txt) | 2a (5’) | 18 | 1.73E-03 | TATTTGTAGCTACAAATA  ||||||||||||||||||  TATTTGTAGCTACAAATA |
| NCAM1 | [chr11:111909689-111909706](http://genome.ucsc.edu/cgi-bin/hgTracks?db=hg17&position=chr11:111909689-111909706&hgt.customText=http://www.neurogenome.org/mcs/tracks/chr11.txt) | 2a (5’) | 18 | 5.13E-03 | TTTCCATTTAAATGGAAA  ||||||||||||||||||  TTTCCATTTAAATGGAAA |
| NLGN1 | [chr3:175618405-175618422](http://genome.ucsc.edu/cgi-bin/hgTracks?db=hg17&position=chr3:175618405-175618422&hgt.customText=http://www.neurogenome.org/mcs/tracks/chr3.txt) | 2a (3’) | 18 | 6.50E-04 | TGTGTGTGTACACACACA  ||||||||||||||||||  TGTGTGTGTACACACACA |
| NRXN1 | [chr2:50727468-50727491](http://genome.ucsc.edu/cgi-bin/hgTracks?db=hg17&position=chr2:50727468-50727491&hgt.customText=http://www.neurogenome.org/mcs/tracks/chr2.txt) | 2b | 24 | 4.93E-07 | TCATCATTAGATATCTAATGATGA  ||||||||||||||||||||||||  TCATCATTAGATATCTAATGATGA |
| NRXN2 | [chr11:64166900-64166921](http://genome.ucsc.edu/cgi-bin/hgTracks?db=hg17&position=chr11:64166900-64166921&hgt.customText=http://www.neurogenome.org/mcs/tracks/chr11.txt) | 1c (5’) | 22 | 4.70E-05 | CGCGGGGCTGCGCAGCCCCGCG  ||||||||||||||||||||||  CGCGGGGCTGCGCAGCCCCGCG |
| PCLO | [chr7:82333088-82333110](http://genome.ucsc.edu/cgi-bin/hgTracks?db=hg17&position=chr7:82333088-82333110&hgt.customText=http://www.neurogenome.org/mcs/tracks/chr7.txt) | 2b | 19 | 1.65E-04 | TATAGTATATAGTATATACTATA  ||||||||||| |||||||||||  TATAGTATATACTATATACTATA |
| PCLO | [chr7:82333087-82333104](http://genome.ucsc.edu/cgi-bin/hgTracks?db=hg17&position=chr7:82333087-82333104&hgt.customText=http://www.neurogenome.org/mcs/tracks/chr7.txt) | 2b | 18 | 6.50E-04 | ATATAGTATATACTATAT  ||||||||||||||||||  ATATAGTATATACTATAT |
| RIMS1 | [chr6:72235008-72235033](http://genome.ucsc.edu/cgi-bin/hgTracks?db=hg17&position=chr6:72235008-72235033&hgt.customText=http://www.neurogenome.org/mcs/tracks/chr6.txt) | 2a (5’) | 26 | 1.20E-07 | ATTATTCATGCAATTGCATGAATAAT  ||||||||||||||||||||||||||  ATTATTCATGCAATTGCATGAATAAT |
| STX18 | [chr4:4978813-4978828](http://genome.ucsc.edu/cgi-bin/hgTracks?db=hg17&position=chr4:4978813-4978828&hgt.customText=http://www.neurogenome.org/mcs/tracks/chr4.txt) | 2a (5’) | 16 | 8.65E-03 | TTTACCTCGAGGTAAA  ||||||||||||||||  TTTACCTCGAGGTAAA |
| STX3A | [chr11:59327271-59327288](http://genome.ucsc.edu/cgi-bin/hgTracks?db=hg17&position=chr11:59327271-59327288&hgt.customText=http://www.neurogenome.org/mcs/tracks/chr11.txt) | 2a (3’) | 18 | 2.28E-03 | AGATCATGATCATGATCT  ||||||||||||||||||  AGATCATGATCATGATCT |
| STXBP5 | [chr6:147702219-147702236](http://genome.ucsc.edu/cgi-bin/hgTracks?db=hg17&position=chr6:147702219-147702236&hgt.customText=http://www.neurogenome.org/mcs/tracks/chr6.txt) | 2b | 18 | 3.44E-03 | AAATAGAAATTTCTATTT  ||||||||||||||||||  AAATAGAAATTTCTATTT |
| SYN3 | [chr22:31420954-31420971](http://genome.ucsc.edu/cgi-bin/hgTracks?db=hg17&position=chr22:31420954-31420971&hgt.customText=http://www.neurogenome.org/mcs/tracks/chr22.txt) | 2b | 18 | 2.87E-03 | GGAGGGTCATGACCCTCC  ||||||||||||||||||  GGAGGGTCATGACCCTCC |
| SYT1 | [chr12:77810719-77810736](http://genome.ucsc.edu/cgi-bin/hgTracks?db=hg17&position=chr12:77810719-77810736&hgt.customText=http://www.neurogenome.org/mcs/tracks/chr12.txt) | 2b | 18 | 9.63E-04 | AACAAATTATAATTTGTT  ||||||||||||||||||  AACAAATTATAATTTGTT |
| SYT3 | [chr19:55855059-55855091](http://genome.ucsc.edu/cgi-bin/hgTracks?db=hg17&position=chr19:55855059-55855091&hgt.customText=http://www.neurogenome.org/mcs/tracks/chr19.txt) | 2b | 21 | 1.04E-04 | TGAGGGGAGGGGGCTGCAAGCCCCCTCCCCTCA  ||||||||||||||| |||||||||||||||  TGAGGGGAGGGGGCTTGCAGCCCCCTCCCCTCA |
| SYTL2 | [chr11:85083349-85083366](http://genome.ucsc.edu/cgi-bin/hgTracks?db=hg17&position=chr11:85083349-85083366&hgt.customText=http://www.neurogenome.org/mcs/tracks/chr11.txt) | 1c (3’) | 18 | 1.20E-03 | AATATGGAATTCCATATT  ||||||||||||||||||  AATATGGAATTCCATATT |
| UNC13C | [chr15:53123950-53123969](http://genome.ucsc.edu/cgi-bin/hgTracks?db=hg17&position=chr15:53123950-53123969&hgt.customText=http://www.neurogenome.org/mcs/tracks/chr15.txt) | 2a (3’) | 20 | 1.07E-04 | TCATTTATAGCTATAAATGA  ||||||||||||||||||||  TCATTTATAGCTATAAATGA |
