# Supplementary material for: Patterns of sequence conservation in presynaptic neural genes
Source: Genome Biol. 2006 Nov 10;7(11):R105. doi: 10.1186/gb-2006-7-11-r105 (PMC1794582; doi:10.1186/gb-2006-7-11-r105)
Supplement: Additional data file 9 — Differential expression of genes [file gb-2006-7-11-r105-S9.doc]

**Differential expression of genes**

We used Michael Eisen’s Cluster v2.11 [62] and Java TreeView 1.0.13 [64] to respectively cluster and visualize expression data from the Genomics Institute of the Novartis Research Foundation (GNF) across 79 human tissues and cell lines. For 144 genes, there were 291 unique probes interrogating transcription. The expression levels of these probes were grouped by *k*-medians clustering into 5 significant clusters, thereby mapping genes directly to expression clusters as shown. The five clusters identified are:

1. transcripts with wide-spread and low level of expression in most tissues/cell-types (“Nonspecific”)
2. transcripts expressed in brain and immune tissues and cell types that are under-expressed in other tissues (“Brain & Immune Enriched”)
3. transcripts with enriched expression in brain tissues with low levels of expression in other tissues (“Brain Enriched”)
4. transcripts or splice forms enriched in hematopoetic derived immune cell-types (“Immune Enriched”)
5. transcripts or splice forms under-expressed in immune tissues and cell-types (“Immune Reduced”)

| Expression / Gene | “Nonspecific | “Brain & Immune Enriched” | “Brain Enriched” | “Immune Enriched” | “Immune Reduced” |
| --- | --- | --- | --- | --- | --- |
| AMPH |  |  | AMPH |  |  |
| APBA1 |  |  |  |  | APBA1 |
| APBA2 |  |  | APBA2 |  |  |
| APBA3 |  |  |  | APBA3 |  |
| ASPM |  |  |  | ASPM | ASPM |
| BSN |  |  | BSN |  |  |
| BZRAP1 |  | BZRAP1 |  |  |  |
| CALM1 |  | CALM1 | CALM1 |  |  |
| CALM2 |  | CALM2 |  |  |  |
| CALM3 |  | CALM3 |  |  |  |
| CALML3 | CALML3 |  |  |  |  |
| CALML4 | CALML4 |  |  | CALML4 | CALML4 |
| CALML5 |  |  |  |  | CALML5 |
| CAMK1 | CAMK1 |  |  |  |  |
| CAMK1D |  | CAMK1D |  |  | CAMK1D |
| CAMK1G |  |  | CAMK1G |  | CAMK1G |
| CAMK2A |  |  | CAMK2A |  | CAMK2A |
| CAMK2B | CAMK2B |  | CAMK2B |  |  |
| CAMK2D | CAMK2D |  |  |  | CAMK2D |
| CAMK2G |  | CAMK2G |  |  |  |
| CAMK2N1 |  |  | CAMK2N1 |  |  |
| CAMK2N2 |  |  |  |  | CAMK2N2 |
| CAMK4 | CAMK4 |  |  |  |  |
| CASK | CASK |  |  |  | CASK |
| CAST |  |  |  | CAST | CAST |
| CAST1 |  |  | CAST1 |  | CAST1 |
| DMXL2 |  | DMXL2 |  |  | DMXL2 |
| DNM1 |  |  | DNM1 |  |  |
| EPIM | EPIM |  |  |  |  |
| EXOC1 |  | EXOC1 |  |  |  |
| EXOC2 |  | EXOC2 |  |  | EXOC2 |
| EXOC3 | EXOC3 |  |  | EXOC3 |  |
| EXOC4 | EXOC4 | EXOC4 |  |  | EXOC4 |
| EXOC5 |  |  |  | EXOC5 |  |
| EXOC6 | EXOC6 |  |  |  |  |
| EXOC7 | EXOC7 | EXOC7 |  | EXOC7 | EXOC7 |
| GDI1 |  | GDI1 |  |  |  |
| GDI2 |  |  |  | GDI2 |  |
| GZMB |  |  |  | GZMB |  |
| NAPA | NAPA | NAPA |  |  |  |
| NAPB |  |  |  |  | NAPB |
| NAPG | NAPG |  |  |  |  |
| NBEA |  |  | NBEA |  |  |
| NCAM1 | NCAM1 |  | NCAM1 |  | NCAM1 |
| NLGN1 |  |  | NLGN1 |  | NLGN1 |
| NLGN2 |  |  |  |  | NLGN2 |
| NLGN3 |  |  |  |  | NLGN3 |
| NLGN4X |  |  | NLGN4X |  |  |
| NLGN4Y |  |  |  |  | NLGN4Y |
| NRXN1 |  |  | NRXN1 |  | NRXN1 |
| NRXN2 |  |  | NRXN2 |  |  |
| NRXN3 |  |  | NRXN3 |  | NRXN3 |
| NSF | NSF | NSF |  |  |  |
| PCLO |  |  | PCLO |  | PCLO |
| RAB3A |  |  | RAB3A |  |  |
| RAB3B | RAB3B |  |  |  | RAB3B |
| RAB3C | RAB3C |  |  |  | RAB3C |
| RAB3D | RAB3D |  |  |  |  |
| RAB3GAP1 | RAB3GAP1 | RAB3GAP1 |  |  |  |
| RAB5A | RAB5A | RAB5A |  |  | RAB5A |
| RAB5B | RAB5B |  |  |  |  |
| RAB5C | RAB5C |  |  |  |  |
| RAB6IP2 |  |  |  |  | RAB6IP2 |
| RABAC1 | RABAC1 |  |  |  |  |
| RABGEF1 | RABGEF1 | RABGEF1 |  |  |  |
| RABGGTA |  |  |  | RABGGTA |  |
| RABGGTB |  | RABGGTB |  | RABGGTB |  |
| RABIF | RABIF |  |  |  |  |
| RIMBP2 | RIMBP2 |  | RIMBP2 |  | RIMBP2 |
| RIMS1 |  |  |  |  | RIMS1 |
| RIMS2 |  |  | RIMS2 |  | RIMS2 |
| RIMS3 | RIMS3 |  | RIMS3 |  |  |
| RIMS4 | RIMS4 |  |  |  |  |
| RPH3A |  |  | RPH3A |  |  |
| SCAMP1 |  | SCAMP1 |  |  | SCAMP1 |
| SCAMP2 | SCAMP2 |  |  |  |  |
| SCAMP3 |  |  |  | SCAMP3 |  |
| SCAMP4 | SCAMP4 |  |  |  |  |
| SCAMP5 |  |  | SCAMP5 |  |  |
| SLC30A3 | SLC30A3 |  |  |  |  |
| SLC30A4 |  |  |  |  | SLC30A4 |
| SNAP25 |  |  | SNAP25 |  |  |
| SNCA |  | SNCA | SNCA |  | SNCA |
| STX10 |  |  |  | STX10 |  |
| STX11 |  |  |  | STX11 |  |
| STX12 |  | STX12 |  |  |  |
| STX16 |  | STX16 |  | STX16 |  |
| STX17 | STX17 |  |  |  |  |
| STX18 | STX18 |  |  |  |  |
| STX19 |  |  |  |  | STX19 |
| STX1A |  |  | STX1A |  |  |
| STX1B2 |  |  |  |  | STX1B2 |
| STX3A | STX3A |  |  |  |  |
| STX4A |  |  |  | STX4A |  |
| STX5A | STX5A |  |  | STX5A |  |
| STX6 | STX6 |  |  |  |  |
| STX7 | STX7 |  |  |  |  |
| STX8 | STX8 |  |  | STX8 |  |
| STXBP1 |  |  | STXBP1 |  |  |
| STXBP2 |  |  |  | STXBP2 |  |
| STXBP3 |  | STXBP3 |  |  | STXBP3 |
| STXBP5 |  |  |  |  | STXBP5 |
| STXBP6 | STXBP6 |  |  |  | STXBP6 |
| SV2A |  |  | SV2A |  |  |
| SV2B |  |  | SV2B |  |  |
| SV2C |  |  |  |  | SV2C |
| SVOP |  |  |  |  | SVOP |
| SYN1 |  |  | SYN1 |  |  |
| SYN2 |  |  | SYN2 |  | SYN2 |
| SYN3 |  |  |  |  | SYN3 |
| SYNGR1 | SYNGR1 |  | SYNGR1 | SYNGR1 |  |
| SYNGR2 |  |  |  | SYNGR2 |  |
| SYNGR3 |  |  | SYNGR3 |  |  |
| SYNGR4 |  |  |  |  | SYNGR4 |
| SYP |  |  | SYP |  |  |
| SYT1 |  |  | SYT1 |  |  |
| SYT10 |  |  |  |  | SYT10 |
| SYT11 |  | SYT11 | SYT11 |  | SYT11 |
| SYT12 |  |  |  |  | SYT12 |
| SYT13 |  |  |  |  | SYT13 |
| SYT14 |  |  |  |  | SYT14 |
| SYT15 |  |  |  |  | SYT15 |
| SYT16 |  |  |  |  | SYT16 |
| SYT17 |  | SYT17 |  |  |  |
| SYT2 |  |  |  |  | SYT2 |
| SYT3 | SYT3 |  |  |  | SYT3 |
| SYT4 |  |  | SYT4 |  |  |
| SYT5 |  |  | SYT5 |  | SYT5 |
| SYT8 |  |  |  |  | SYT8 |
| SYT9 |  |  |  |  | SYT9 |
| SYTL1 |  |  |  | SYTL1 |  |
| SYTL2 | SYTL2 | SYTL2 |  |  |  |
| SYTL3 | SYTL3 |  |  |  |  |
| SYTL4 |  |  |  |  | SYTL4 |
| UNC13A |  |  | UNC13A |  |  |
| UNC13B |  |  |  |  | UNC13B |
| UNC13C |  |  | UNC13C |  |  |
| UNC13D | UNC13D |  |  | UNC13D |  |
| VAMP1 | VAMP1 | VAMP1 |  |  |  |
| VAMP2 | VAMP2 | VAMP2 |  | VAMP2 |  |
| VAMP3 | VAMP3 | VAMP3 |  | VAMP3 |  |
| VAMP4 | VAMP4 | VAMP4 |  |  |  |
| VAMP5 |  |  |  | VAMP5 |  |
| VAMP8 |  |  |  | VAMP8 |  |
