# Supplementary material for: Patterns of sequence conservation in presynaptic neural genes
Source: Genome Biol. 2006 Nov 10;7(11):R105. doi: 10.1186/gb-2006-7-11-r105 (PMC1794582; doi:10.1186/gb-2006-7-11-r105)
Supplement: Additional data file 11 — Large most conserved elements (LMCEs) identified [file gb-2006-7-11-r105-S11.doc]

## Large most conserved elements (LMCEs) identified

The largest most conserved elements (LMCEs; ≥ 360bp) are shown with their respective annotations and conservation. Elements are marked as conserved if the human sequence aligned over the majority (ie more than half) of the sequence (disregarding indels) from at least one genome in the primate, tetrapod, bird and fish groups. In the primate group there was only chimpanzee, in the tetrapod group were dog, mouse, and rat, in the bird group there was only chicken, and in the fish group there were pufferfish and zebrafish. In consecutive order, the table lists the element internal name, its position, length, relative genic position, evidence of transcription from tiling array data, evidence of transcription from DoTS, stable RNA secondary structure, conserved in primates (prim), conserved in tetrapods (tpod), conserved in bird, and conserved in fish.

| **#** | **element** | **pos** | **len** | **type** | **annotation** | | | **Conservation** | | | |
| --- | --- | --- | --- | --- | --- | --- | --- | --- | --- | --- | --- |
|  |  |  |  |  | **TnFg** | **DoTS** | **RNA** | **prim** | **tpod** | **bird** | **fish** |
| 1 | AMPH.112 | [chr7:38197078-38197442](http://genome.ucsc.edu/cgi-bin/hgTracks?db=hg17&position=chr7:38197078-38197442&hgt.customText=http://www.neurogenome.org/mcs/tracks/chr7.txt) | 365 | 1c (3’) | ● | ● |  | ● | ● | ● |  |
| 2 | APBA1.10 | [chr9:69516579-69517013](http://genome.ucsc.edu/cgi-bin/hgTracks?db=hg17&position=chr9:69516579-69517013&hgt.customText=http://www.neurogenome.org/mcs/tracks/chr9.txt) | 435 | 1c (5’) | N/A | ● |  | ● | ● |  |  |
| 3 | APBA1.14 | [chr9:69515845-69516287](http://genome.ucsc.edu/cgi-bin/hgTracks?db=hg17&position=chr9:69515845-69516287&hgt.customText=http://www.neurogenome.org/mcs/tracks/chr9.txt) | 443 | 2b | N/A |  |  | ● | ● |  |  |
| 4 | APBA1.124 | [chr9:69360474-69361005](http://genome.ucsc.edu/cgi-bin/hgTracks?db=hg17&position=chr9:69360474-69361005&hgt.customText=http://www.neurogenome.org/mcs/tracks/chr9.txt) | 532 | 1a | N/A | ● |  | ● | ● | ● | ● |
| 5 | APBA2.11 | [chr15:26842336-26842809](http://genome.ucsc.edu/cgi-bin/hgTracks?db=hg17&position=chr15:26842336-26842809&hgt.customText=http://www.neurogenome.org/mcs/tracks/chr15.txt) | 474 | 2a (5’) | N/A |  | ● | ● | ● |  |  |
| 6 | BSN.11 | [chr3:49566941-49567366](http://genome.ucsc.edu/cgi-bin/hgTracks?db=hg17&position=chr3:49566941-49567366&hgt.customText=http://www.neurogenome.org/mcs/tracks/chr3.txt) | 426 | 1c (5’) | N/A | ● |  | ● | ● |  |  |
| 7 | BSN.98 | [chr3:49664769-49665414](http://genome.ucsc.edu/cgi-bin/hgTracks?db=hg17&position=chr3:49664769-49665414&hgt.customText=http://www.neurogenome.org/mcs/tracks/chr3.txt) | 646 | 1a | N/A | ● | ● | ● | ● | ● | ● |
| 8 | BSN.138 | [chr3:49669088-49669459](http://genome.ucsc.edu/cgi-bin/hgTracks?db=hg17&position=chr3:49669088-49669459&hgt.customText=http://www.neurogenome.org/mcs/tracks/chr3.txt) | 372 | 1a | N/A | ● | ● | ● | ● | ● | ● |
| 9 | BSN.143 | [chr3:49670085-49670616](http://genome.ucsc.edu/cgi-bin/hgTracks?db=hg17&position=chr3:49670085-49670616&hgt.customText=http://www.neurogenome.org/mcs/tracks/chr3.txt) | 532 | 1a | N/A | ● |  | ● | ● | ● | ● |
| 10 | CALM1.28 | [chr14:89932945-89933340](http://genome.ucsc.edu/cgi-bin/hgTracks?db=hg17&position=chr14:89932945-89933340&hgt.customText=http://www.neurogenome.org/mcs/tracks/chr14.txt) | 396 | 1c (5’) | ● | ● |  | ● | ● |  |  |
| 11 | CALM2.78 | [chr2:47329395-47330015](http://genome.ucsc.edu/cgi-bin/hgTracks?db=hg17&position=chr2:47329395-47330015&hgt.customText=http://www.neurogenome.org/mcs/tracks/chr2.txt) | 621 | 2a (5’) | N/A |  |  | ● | ● |  |  |
| 12 | CALM3.19 | [chr19:51754173-51754568](http://genome.ucsc.edu/cgi-bin/hgTracks?db=hg17&position=chr19:51754173-51754568&hgt.customText=http://www.neurogenome.org/mcs/tracks/chr19.txt) | 396 | 2a (5’) | ● |  | ● | ● | ● |  | ● |
| 13 | CALM3.53 | [chr19:51804219-51804647](http://genome.ucsc.edu/cgi-bin/hgTracks?db=hg17&position=chr19:51804219-51804647&hgt.customText=http://www.neurogenome.org/mcs/tracks/chr19.txt) | 429 | 1c (3’) | ● | ● |  | ● | ● |  |  |
| 14 | CALM3.59 | [chr19:51805331-51805783](http://genome.ucsc.edu/cgi-bin/hgTracks?db=hg17&position=chr19:51805331-51805783&hgt.customText=http://www.neurogenome.org/mcs/tracks/chr19.txt) | 453 | 1c (3’) | ● | ● |  | ● | ● |  |  |
| 15 | CALML3.3 | [chr10:5557044-5557473](http://genome.ucsc.edu/cgi-bin/hgTracks?db=hg17&position=chr10:5557044-5557473&hgt.customText=http://www.neurogenome.org/mcs/tracks/chr10.txt) | 430 | 1a | N/A | ● |  | ● | ● | ● | ● |
| 16 | CAMK1G.28 | [chr1:204826236-204826633](http://genome.ucsc.edu/cgi-bin/hgTracks?db=hg17&position=chr1:204826236-204826633&hgt.customText=http://www.neurogenome.org/mcs/tracks/chr1.txt) | 398 | 2a (5’) | N/A |  | ● | ● | ● |  |  |
| 17 | CAMK1G.40 | [chr1:204833976-204834375](http://genome.ucsc.edu/cgi-bin/hgTracks?db=hg17&position=chr1:204833976-204834375&hgt.customText=http://www.neurogenome.org/mcs/tracks/chr1.txt) | 400 | 2a (5’) | N/A |  | ● | ● | ● |  |  |
| 18 | CAMK1G.91 | [chr1:204885874-204886314](http://genome.ucsc.edu/cgi-bin/hgTracks?db=hg17&position=chr1:204885874-204886314&hgt.customText=http://www.neurogenome.org/mcs/tracks/chr1.txt) | 441 | 2a (5’) | N/A |  |  | ● | ● |  |  |
| 19 | CAMK1G.99 | [chr1:204894463-204894988](http://genome.ucsc.edu/cgi-bin/hgTracks?db=hg17&position=chr1:204894463-204894988&hgt.customText=http://www.neurogenome.org/mcs/tracks/chr1.txt) | 526 | 2a (5’) | N/A |  |  | ● | ● | ● |  |
| 20 | CAMK1G.158 | [chr1:204956522-204956914](http://genome.ucsc.edu/cgi-bin/hgTracks?db=hg17&position=chr1:204956522-204956914&hgt.customText=http://www.neurogenome.org/mcs/tracks/chr1.txt) | 393 | 2a (5’) | N/A |  |  | ● | ● | ● |  |
| 21 | CAMK1G.200 | [chr1:204996582-204997032](http://genome.ucsc.edu/cgi-bin/hgTracks?db=hg17&position=chr1:204996582-204997032&hgt.customText=http://www.neurogenome.org/mcs/tracks/chr1.txt) | 451 | 2a (5’) | N/A |  |  | ● | ● |  |  |
| 22 | CAMK1G.218 | [chr1:205012151-205012699](http://genome.ucsc.edu/cgi-bin/hgTracks?db=hg17&position=chr1:205012151-205012699&hgt.customText=http://www.neurogenome.org/mcs/tracks/chr1.txt) | 549 | 2a (5’) | N/A |  |  | ● | ● | ● |  |
| 23 | CAMK1G.230 | [chr1:205029249-205029890](http://genome.ucsc.edu/cgi-bin/hgTracks?db=hg17&position=chr1:205029249-205029890&hgt.customText=http://www.neurogenome.org/mcs/tracks/chr1.txt) | 642 | 2a (5’) | N/A |  |  | ● | ● |  |  |
| 24 | CAMK1G.234 | [chr1:205031788-205032177](http://genome.ucsc.edu/cgi-bin/hgTracks?db=hg17&position=chr1:205031788-205032177&hgt.customText=http://www.neurogenome.org/mcs/tracks/chr1.txt) | 390 | 2a (5’) | N/A |  |  | ● | ● |  |  |
| 25 | CAMK1G.267 | [chr1:205086831-205087212](http://genome.ucsc.edu/cgi-bin/hgTracks?db=hg17&position=chr1:205086831-205087212&hgt.customText=http://www.neurogenome.org/mcs/tracks/chr1.txt) | 382 | 2a (5’) | N/A |  | ● | ● | ● |  |  |
| 26 | CAMK1G.283 | [chr1:205099212-205099630](http://genome.ucsc.edu/cgi-bin/hgTracks?db=hg17&position=chr1:205099212-205099630&hgt.customText=http://www.neurogenome.org/mcs/tracks/chr1.txt) | 419 | 2a (5’) | N/A |  |  | ● | ● |  |  |
| 27 | CAMK1G.400 | [chr1:205225684-205226139](http://genome.ucsc.edu/cgi-bin/hgTracks?db=hg17&position=chr1:205225684-205226139&hgt.customText=http://www.neurogenome.org/mcs/tracks/chr1.txt) | 456 | 2a (5’) | N/A |  |  | ● | ● | ● |  |
| 28 | CAMK1G.418 | [chr1:205252809-205253356](http://genome.ucsc.edu/cgi-bin/hgTracks?db=hg17&position=chr1:205252809-205253356&hgt.customText=http://www.neurogenome.org/mcs/tracks/chr1.txt) | 548 | 2a (5’) | N/A |  |  | ● | ● | ● |  |
| 29 | CAMK1G.440 | [chr1:205309726-205310160](http://genome.ucsc.edu/cgi-bin/hgTracks?db=hg17&position=chr1:205309726-205310160&hgt.customText=http://www.neurogenome.org/mcs/tracks/chr1.txt) | 435 | 2a (5’) | N/A |  |  | ● | ● | ● |  |
| 30 | CAMK1G.451 | [chr1:205329632-205330102](http://genome.ucsc.edu/cgi-bin/hgTracks?db=hg17&position=chr1:205329632-205330102&hgt.customText=http://www.neurogenome.org/mcs/tracks/chr1.txt) | 471 | 2a (5’) | N/A |  |  | ● | ● | ● |  |
| 31 | CAMK1G.461 | [chr1:205336114-205336554](http://genome.ucsc.edu/cgi-bin/hgTracks?db=hg17&position=chr1:205336114-205336554&hgt.customText=http://www.neurogenome.org/mcs/tracks/chr1.txt) | 441 | 2a (5’) | N/A |  |  | ● | ● | ● |  |
| 32 | CAMK1G.482 | [chr1:205364863-205365272](http://genome.ucsc.edu/cgi-bin/hgTracks?db=hg17&position=chr1:205364863-205365272&hgt.customText=http://www.neurogenome.org/mcs/tracks/chr1.txt) | 410 | 2a (5’) | N/A |  |  | ● | ● |  |  |
| 33 | CAMK1G.496 | [chr1:205382355-205382862](http://genome.ucsc.edu/cgi-bin/hgTracks?db=hg17&position=chr1:205382355-205382862&hgt.customText=http://www.neurogenome.org/mcs/tracks/chr1.txt) | 508 | 2a (5’) | N/A |  |  | ● | ● |  |  |
| 34 | CAMK1G.501 | [chr1:205386383-205386855](http://genome.ucsc.edu/cgi-bin/hgTracks?db=hg17&position=chr1:205386383-205386855&hgt.customText=http://www.neurogenome.org/mcs/tracks/chr1.txt) | 473 | 2a (5’) | N/A |  |  | ● | ● |  |  |
| 35 | CAMK1G.547 | [chr1:205439135-205439510](http://genome.ucsc.edu/cgi-bin/hgTracks?db=hg17&position=chr1:205439135-205439510&hgt.customText=http://www.neurogenome.org/mcs/tracks/chr1.txt) | 376 | 2a (5’) | N/A |  |  | ● | ● |  |  |
| 36 | CAMK1G.572 | [chr1:205454872-205455355](http://genome.ucsc.edu/cgi-bin/hgTracks?db=hg17&position=chr1:205454872-205455355&hgt.customText=http://www.neurogenome.org/mcs/tracks/chr1.txt) | 484 | 2a (5’) | N/A |  |  | ● | ● |  |  |
| 37 | CAMK1G.579 | [chr1:205471145-205471701](http://genome.ucsc.edu/cgi-bin/hgTracks?db=hg17&position=chr1:205471145-205471701&hgt.customText=http://www.neurogenome.org/mcs/tracks/chr1.txt) | 557 | 2a (5’) | N/A |  | ● | ● | ● |  |  |
| 38 | CAMK1G.592 | [chr1:205485458-205485955](http://genome.ucsc.edu/cgi-bin/hgTracks?db=hg17&position=chr1:205485458-205485955&hgt.customText=http://www.neurogenome.org/mcs/tracks/chr1.txt) | 498 | 2a (5’) | N/A |  |  | ● | ● | ● |  |
| 39 | CAMK1G.656 | [chr1:205571867-205572449](http://genome.ucsc.edu/cgi-bin/hgTracks?db=hg17&position=chr1:205571867-205572449&hgt.customText=http://www.neurogenome.org/mcs/tracks/chr1.txt) | 583 | 2a (5’) | N/A |  |  | ● | ● |  |  |
| 40 | CAMK1G.664 | [chr1:205575184-205575551](http://genome.ucsc.edu/cgi-bin/hgTracks?db=hg17&position=chr1:205575184-205575551&hgt.customText=http://www.neurogenome.org/mcs/tracks/chr1.txt) | 368 | 2a (5’) | N/A |  |  | ● | ● |  |  |
| 41 | CAMK1G.668 | [chr1:205585294-205585760](http://genome.ucsc.edu/cgi-bin/hgTracks?db=hg17&position=chr1:205585294-205585760&hgt.customText=http://www.neurogenome.org/mcs/tracks/chr1.txt) | 467 | 2a (5’) | N/A |  |  | ● | ● | ● |  |
| 42 | CAMK1G.726 | [chr1:205664366-205664798](http://genome.ucsc.edu/cgi-bin/hgTracks?db=hg17&position=chr1:205664366-205664798&hgt.customText=http://www.neurogenome.org/mcs/tracks/chr1.txt) | 433 | 2a (5’) | N/A |  |  | ● | ● | ● |  |
| 43 | CAMK1G.754 | [chr1:205707846-205708219](http://genome.ucsc.edu/cgi-bin/hgTracks?db=hg17&position=chr1:205707846-205708219&hgt.customText=http://www.neurogenome.org/mcs/tracks/chr1.txt) | 374 | 2a (5’) | N/A |  |  | ● | ● | ● |  |
| 44 | CAMK1G.774 | [chr1:205719394-205719822](http://genome.ucsc.edu/cgi-bin/hgTracks?db=hg17&position=chr1:205719394-205719822&hgt.customText=http://www.neurogenome.org/mcs/tracks/chr1.txt) | 429 | 2a (5’) | N/A |  | ● | ● | ● |  |  |
| 45 | CAMK1G.818 | [chr1:205784523-205784889](http://genome.ucsc.edu/cgi-bin/hgTracks?db=hg17&position=chr1:205784523-205784889&hgt.customText=http://www.neurogenome.org/mcs/tracks/chr1.txt) | 367 | 2a (5’) | N/A |  | ● | ● | ● |  |  |
| 46 | CAMK1G.834 | [chr1:205795784-205796153](http://genome.ucsc.edu/cgi-bin/hgTracks?db=hg17&position=chr1:205795784-205796153&hgt.customText=http://www.neurogenome.org/mcs/tracks/chr1.txt) | 370 | 2a (5’) | N/A |  |  | ● | ● | ● |  |
| 47 | CAMK1G.859 | [chr1:205818473-205818914](http://genome.ucsc.edu/cgi-bin/hgTracks?db=hg17&position=chr1:205818473-205818914&hgt.customText=http://www.neurogenome.org/mcs/tracks/chr1.txt) | 442 | 2a (5’) | N/A |  |  | ● | ● |  |  |
| 48 | CAMK1G.865 | [chr1:205829394-205830029](http://genome.ucsc.edu/cgi-bin/hgTracks?db=hg17&position=chr1:205829394-205830029&hgt.customText=http://www.neurogenome.org/mcs/tracks/chr1.txt) | 636 | 2a (5’) | N/A |  | ● | ● | ● |  | ● |
| 49 | CAMK1G.868 | [chr1:205849868-205850322](http://genome.ucsc.edu/cgi-bin/hgTracks?db=hg17&position=chr1:205849868-205850322&hgt.customText=http://www.neurogenome.org/mcs/tracks/chr1.txt) | 455 | 2a (5’) | N/A |  |  | ● | ● |  |  |
| 50 | CAMK1G.880 | [chr1:205864699-205865201](http://genome.ucsc.edu/cgi-bin/hgTracks?db=hg17&position=chr1:205864699-205865201&hgt.customText=http://www.neurogenome.org/mcs/tracks/chr1.txt) | 503 | 2a (5’) | N/A |  | ● | ● | ● | ● |  |
| 51 | CAMK1G.939 | [chr1:205928998-205929383](http://genome.ucsc.edu/cgi-bin/hgTracks?db=hg17&position=chr1:205928998-205929383&hgt.customText=http://www.neurogenome.org/mcs/tracks/chr1.txt) | 386 | 2a (5’) | N/A |  |  | ● | ● |  |  |
| 52 | CAMK1G.1073 | [chr1:206084590-206085004](http://genome.ucsc.edu/cgi-bin/hgTracks?db=hg17&position=chr1:206084590-206085004&hgt.customText=http://www.neurogenome.org/mcs/tracks/chr1.txt) | 415 | 2a (5’) | N/A |  |  | ● | ● | ● |  |
| 53 | CAMK2B.11 | [chr7:44138031-44138446](http://genome.ucsc.edu/cgi-bin/hgTracks?db=hg17&position=chr7:44138031-44138446&hgt.customText=http://www.neurogenome.org/mcs/tracks/chr7.txt) | 416 | 1c (5’) | ● | ● |  | ● | ● |  |  |
| 54 | CAMK2D.164 | [chr4:114816477-114816859](http://genome.ucsc.edu/cgi-bin/hgTracks?db=hg17&position=chr4:114816477-114816859&hgt.customText=http://www.neurogenome.org/mcs/tracks/chr4.txt) | 383 | 2b | N/A |  | ● | ● | ● |  |  |
| 55 | CAMK2D.240 | [chr4:114731141-114731704](http://genome.ucsc.edu/cgi-bin/hgTracks?db=hg17&position=chr4:114731141-114731704&hgt.customText=http://www.neurogenome.org/mcs/tracks/chr4.txt) | 564 | 1c (3’) | N/A | ● |  | ● | ● | ● |  |
| 56 | CAMK2G.39 | [chr10:75303453-75303812](http://genome.ucsc.edu/cgi-bin/hgTracks?db=hg17&position=chr10:75303453-75303812&hgt.customText=http://www.neurogenome.org/mcs/tracks/chr10.txt) | 360 | 2b | N/A |  | ● |  | ● |  |  |
| 57 | CAMK2G.73 | [chr10:75289606-75290230](http://genome.ucsc.edu/cgi-bin/hgTracks?db=hg17&position=chr10:75289606-75290230&hgt.customText=http://www.neurogenome.org/mcs/tracks/chr10.txt) | 625 | 2b | N/A |  | ● | ● | ● |  |  |
| 58 | CAMK2G.113 | [chr10:75269169-75269550](http://genome.ucsc.edu/cgi-bin/hgTracks?db=hg17&position=chr10:75269169-75269550&hgt.customText=http://www.neurogenome.org/mcs/tracks/chr10.txt) | 382 | 1b | N/A |  |  | ● | ● |  | ● |
| 59 | CAMK2G.140 | [chr10:75249213-75249609](http://genome.ucsc.edu/cgi-bin/hgTracks?db=hg17&position=chr10:75249213-75249609&hgt.customText=http://www.neurogenome.org/mcs/tracks/chr10.txt) | 397 | 1b | N/A | ● |  | ● | ● |  |  |
| 60 | CAMK2G.155 | [chr10:75242805-75243413](http://genome.ucsc.edu/cgi-bin/hgTracks?db=hg17&position=chr10:75242805-75243413&hgt.customText=http://www.neurogenome.org/mcs/tracks/chr10.txt) | 609 | 1c (3’) | N/A | ● | ● | ● | ● |  |  |
| 61 | CAMK2G.156 | [chr10:75242242-75242784](http://genome.ucsc.edu/cgi-bin/hgTracks?db=hg17&position=chr10:75242242-75242784&hgt.customText=http://www.neurogenome.org/mcs/tracks/chr10.txt) | 543 | 1c (3’) | N/A | ● | ● | ● | ● | ● |  |
| 62 | CAMK4.15 | [chr5:110556085-110556643](http://genome.ucsc.edu/cgi-bin/hgTracks?db=hg17&position=chr5:110556085-110556643&hgt.customText=http://www.neurogenome.org/mcs/tracks/chr5.txt) | 559 | 2a (5’) | N/A | ● |  | ● | ● | ● | ● |
| 63 | CASK.7 | [chrX:41894236-41894619](http://genome.ucsc.edu/cgi-bin/hgTracks?db=hg17&position=chrX:41894236-41894619&hgt.customText=http://www.neurogenome.org/mcs/tracks/chrX.txt) | 384 | 2a (5’) | ● |  |  | ● | ● |  |  |
| 64 | CASK.18 | [chrX:41883493-41883866](http://genome.ucsc.edu/cgi-bin/hgTracks?db=hg17&position=chrX:41883493-41883866&hgt.customText=http://www.neurogenome.org/mcs/tracks/chrX.txt) | 374 | 2a (5’) |  |  | ● |  | ● |  |  |
| 65 | CASK.122 | [chrX:41759261-41759687](http://genome.ucsc.edu/cgi-bin/hgTracks?db=hg17&position=chrX:41759261-41759687&hgt.customText=http://www.neurogenome.org/mcs/tracks/chrX.txt) | 427 | 2a (5’) |  |  |  | ● | ● |  |  |
| 66 | CASK.150 | [chrX:41730343-41730722](http://genome.ucsc.edu/cgi-bin/hgTracks?db=hg17&position=chrX:41730343-41730722&hgt.customText=http://www.neurogenome.org/mcs/tracks/chrX.txt) | 380 | 2a (5’) |  |  |  |  | ● |  |  |
| 67 | CASK.244 | [chrX:41629035-41629409](http://genome.ucsc.edu/cgi-bin/hgTracks?db=hg17&position=chrX:41629035-41629409&hgt.customText=http://www.neurogenome.org/mcs/tracks/chrX.txt) | 375 | 2a (5’) |  |  |  | ● | ● |  |  |
| 68 | CASK.248 | [chrX:41616636-41617065](http://genome.ucsc.edu/cgi-bin/hgTracks?db=hg17&position=chrX:41616636-41617065&hgt.customText=http://www.neurogenome.org/mcs/tracks/chrX.txt) | 430 | 2a (5’) |  |  |  | ● | ● |  |  |
| 69 | CASK.332 | [chrX:41538665-41539262](http://genome.ucsc.edu/cgi-bin/hgTracks?db=hg17&position=chrX:41538665-41539262&hgt.customText=http://www.neurogenome.org/mcs/tracks/chrX.txt) | 598 | 2a (5’) | ● |  |  |  | ● |  |  |
| 70 | CASK.333 | [chrX:41537444-41538656](http://genome.ucsc.edu/cgi-bin/hgTracks?db=hg17&position=chrX:41537444-41538656&hgt.customText=http://www.neurogenome.org/mcs/tracks/chrX.txt) | 1213 | 1b | ● |  | ● | ● | ● |  |  |
| 71 | CASK.453 | [chrX:41395394-41395829](http://genome.ucsc.edu/cgi-bin/hgTracks?db=hg17&position=chrX:41395394-41395829&hgt.customText=http://www.neurogenome.org/mcs/tracks/chrX.txt) | 436 | 2b |  |  |  | ● | ● |  |  |
| 72 | CASK.562 | [chrX:41290825-41291262](http://genome.ucsc.edu/cgi-bin/hgTracks?db=hg17&position=chrX:41290825-41291262&hgt.customText=http://www.neurogenome.org/mcs/tracks/chrX.txt) | 438 | 1c (3’) | ● |  |  | ● | ● | ● |  |
| 73 | CASK.564 | [chrX:41290152-41290515](http://genome.ucsc.edu/cgi-bin/hgTracks?db=hg17&position=chrX:41290152-41290515&hgt.customText=http://www.neurogenome.org/mcs/tracks/chrX.txt) | 364 | 1c (3’) | ● |  |  | ● | ● |  |  |
| 74 | CASK.566 | [chrX:41289346-41289739](http://genome.ucsc.edu/cgi-bin/hgTracks?db=hg17&position=chrX:41289346-41289739&hgt.customText=http://www.neurogenome.org/mcs/tracks/chrX.txt) | 394 | 1c (3’) | ● | ● |  | ● | ● |  |  |
| 75 | CASK.691 | [chrX:41144809-41145250](http://genome.ucsc.edu/cgi-bin/hgTracks?db=hg17&position=chrX:41144809-41145250&hgt.customText=http://www.neurogenome.org/mcs/tracks/chrX.txt) | 442 | 2b |  |  |  | ● | ● |  |  |
| 76 | CASK.704 | [chrX:41135404-41135897](http://genome.ucsc.edu/cgi-bin/hgTracks?db=hg17&position=chrX:41135404-41135897&hgt.customText=http://www.neurogenome.org/mcs/tracks/chrX.txt) | 494 | 1c (3’) | ● | ● | ● | ● | ● | ● |  |
| 77 | CASK.705 | [chrX:41134672-41135381](http://genome.ucsc.edu/cgi-bin/hgTracks?db=hg17&position=chrX:41134672-41135381&hgt.customText=http://www.neurogenome.org/mcs/tracks/chrX.txt) | 710 | 1c (3’) | ● | ● |  | ● | ● | ● |  |
| 78 | CASK.716 | [chrX:41130395-41131031](http://genome.ucsc.edu/cgi-bin/hgTracks?db=hg17&position=chrX:41130395-41131031&hgt.customText=http://www.neurogenome.org/mcs/tracks/chrX.txt) | 637 | 2a (3’) | ● | ● |  | ● | ● |  |  |
| 79 | CAST.40 | [chr5:95927217-95927710](http://genome.ucsc.edu/cgi-bin/hgTracks?db=hg17&position=chr5:95927217-95927710&hgt.customText=http://www.neurogenome.org/mcs/tracks/chr5.txt) | 494 | 2a (5’) | N/A |  |  | ● | ● |  |  |
| 80 | CAST1.79 | [chr3:56443704-56444082](http://genome.ucsc.edu/cgi-bin/hgTracks?db=hg17&position=chr3:56443704-56444082&hgt.customText=http://www.neurogenome.org/mcs/tracks/chr3.txt) | 379 | 1a | N/A | ● |  | ● | ● | ● | ● |
| 81 | CAST1.88 | [chr3:56432746-56433161](http://genome.ucsc.edu/cgi-bin/hgTracks?db=hg17&position=chr3:56432746-56433161&hgt.customText=http://www.neurogenome.org/mcs/tracks/chr3.txt) | 416 | 2b | N/A |  |  | ● | ● |  |  |
| 82 | CAST1.155 | [chr3:56317507-56318061](http://genome.ucsc.edu/cgi-bin/hgTracks?db=hg17&position=chr3:56317507-56318061&hgt.customText=http://www.neurogenome.org/mcs/tracks/chr3.txt) | 555 | 2b | N/A |  |  | ● | ● |  |  |
| 83 | CAST1.206 | [chr3:56257755-56258219](http://genome.ucsc.edu/cgi-bin/hgTracks?db=hg17&position=chr3:56257755-56258219&hgt.customText=http://www.neurogenome.org/mcs/tracks/chr3.txt) | 465 | 2b | N/A |  |  | ● | ● |  |  |
| 84 | CAST1.221 | [chr3:56246386-56246863](http://genome.ucsc.edu/cgi-bin/hgTracks?db=hg17&position=chr3:56246386-56246863&hgt.customText=http://www.neurogenome.org/mcs/tracks/chr3.txt) | 478 | 2b | N/A |  |  | ● | ● |  |  |
| 85 | CAST1.247 | [chr3:56205604-56206069](http://genome.ucsc.edu/cgi-bin/hgTracks?db=hg17&position=chr3:56205604-56206069&hgt.customText=http://www.neurogenome.org/mcs/tracks/chr3.txt) | 466 | 2b | N/A |  | ● | ● | ● |  |  |
| 86 | CAST1.304 | [chr3:56120851-56121447](http://genome.ucsc.edu/cgi-bin/hgTracks?db=hg17&position=chr3:56120851-56121447&hgt.customText=http://www.neurogenome.org/mcs/tracks/chr3.txt) | 597 | 2b | N/A |  | ● | ● | ● | ● |  |
| 87 | CAST1.315 | [chr3:56101399-56101810](http://genome.ucsc.edu/cgi-bin/hgTracks?db=hg17&position=chr3:56101399-56101810&hgt.customText=http://www.neurogenome.org/mcs/tracks/chr3.txt) | 412 | 2b | N/A |  |  | ● | ● | ● |  |
| 88 | CAST1.351 | [chr3:56069624-56070243](http://genome.ucsc.edu/cgi-bin/hgTracks?db=hg17&position=chr3:56069624-56070243&hgt.customText=http://www.neurogenome.org/mcs/tracks/chr3.txt) | 620 | 2b | N/A |  | ● | ● | ● |  |  |
| 89 | CAST1.374 | [chr3:56028556-56029048](http://genome.ucsc.edu/cgi-bin/hgTracks?db=hg17&position=chr3:56028556-56029048&hgt.customText=http://www.neurogenome.org/mcs/tracks/chr3.txt) | 493 | 2b | N/A |  |  | ● | ● |  |  |
| 90 | CAST1.411 | [chr3:55997990-55998510](http://genome.ucsc.edu/cgi-bin/hgTracks?db=hg17&position=chr3:55997990-55998510&hgt.customText=http://www.neurogenome.org/mcs/tracks/chr3.txt) | 521 | 2b | N/A |  | ● | ● | ● |  |  |
| 91 | CAST1.435 | [chr3:55977714-55978416](http://genome.ucsc.edu/cgi-bin/hgTracks?db=hg17&position=chr3:55977714-55978416&hgt.customText=http://www.neurogenome.org/mcs/tracks/chr3.txt) | 703 | 2b | N/A |  | ● | ● | ● |  |  |
| 92 | CAST1.518 | [chr3:55898220-55898667](http://genome.ucsc.edu/cgi-bin/hgTracks?db=hg17&position=chr3:55898220-55898667&hgt.customText=http://www.neurogenome.org/mcs/tracks/chr3.txt) | 448 | 2b | N/A |  | ● | ● | ● |  |  |
| 93 | CAST1.554 | [chr3:55869031-55869419](http://genome.ucsc.edu/cgi-bin/hgTracks?db=hg17&position=chr3:55869031-55869419&hgt.customText=http://www.neurogenome.org/mcs/tracks/chr3.txt) | 389 | 2b | N/A |  |  | ● | ● | ● |  |
| 94 | CAST1.581 | [chr3:55828151-55828811](http://genome.ucsc.edu/cgi-bin/hgTracks?db=hg17&position=chr3:55828151-55828811&hgt.customText=http://www.neurogenome.org/mcs/tracks/chr3.txt) | 661 | 2b | N/A |  |  | ● | ● | ● |  |
| 95 | CAST1.593 | [chr3:55804206-55804622](http://genome.ucsc.edu/cgi-bin/hgTracks?db=hg17&position=chr3:55804206-55804622&hgt.customText=http://www.neurogenome.org/mcs/tracks/chr3.txt) | 417 | 2b | N/A |  | ● | ● | ● |  |  |
| 96 | CAST1.594 | [chr3:55803789-55804172](http://genome.ucsc.edu/cgi-bin/hgTracks?db=hg17&position=chr3:55803789-55804172&hgt.customText=http://www.neurogenome.org/mcs/tracks/chr3.txt) | 384 | 2b | N/A |  |  | ● | ● |  |  |
| 97 | CAST1.628 | [chr3:55764510-55765254](http://genome.ucsc.edu/cgi-bin/hgTracks?db=hg17&position=chr3:55764510-55765254&hgt.customText=http://www.neurogenome.org/mcs/tracks/chr3.txt) | 745 | 2b | N/A |  |  | ● | ● | ● |  |
| 98 | CAST1.693 | [chr3:55711095-55711530](http://genome.ucsc.edu/cgi-bin/hgTracks?db=hg17&position=chr3:55711095-55711530&hgt.customText=http://www.neurogenome.org/mcs/tracks/chr3.txt) | 436 | 2b | N/A |  |  | ● | ● | ● |  |
| 99 | CAST1.699 | [chr3:55708190-55708583](http://genome.ucsc.edu/cgi-bin/hgTracks?db=hg17&position=chr3:55708190-55708583&hgt.customText=http://www.neurogenome.org/mcs/tracks/chr3.txt) | 394 | 1b | N/A |  |  | ● | ● | ● |  |
| 100 | CAST1.732 | [chr3:55692284-55692759](http://genome.ucsc.edu/cgi-bin/hgTracks?db=hg17&position=chr3:55692284-55692759&hgt.customText=http://www.neurogenome.org/mcs/tracks/chr3.txt) | 476 | 2b | N/A |  |  | ● | ● |  |  |
| 101 | CAST1.777 | [chr3:55655025-55655430](http://genome.ucsc.edu/cgi-bin/hgTracks?db=hg17&position=chr3:55655025-55655430&hgt.customText=http://www.neurogenome.org/mcs/tracks/chr3.txt) | 406 | 2b | N/A |  |  | ● | ● |  |  |
| 102 | CAST1.782 | [chr3:55647196-55647730](http://genome.ucsc.edu/cgi-bin/hgTracks?db=hg17&position=chr3:55647196-55647730&hgt.customText=http://www.neurogenome.org/mcs/tracks/chr3.txt) | 535 | 2b | N/A |  |  | ● | ● |  |  |
| 103 | CAST1.790 | [chr3:55640035-55640433](http://genome.ucsc.edu/cgi-bin/hgTracks?db=hg17&position=chr3:55640035-55640433&hgt.customText=http://www.neurogenome.org/mcs/tracks/chr3.txt) | 399 | 2b | N/A |  | ● | ● | ● |  |  |
| 104 | CAST1.801 | [chr3:55629637-55630108](http://genome.ucsc.edu/cgi-bin/hgTracks?db=hg17&position=chr3:55629637-55630108&hgt.customText=http://www.neurogenome.org/mcs/tracks/chr3.txt) | 472 | 2b | N/A |  |  | ● | ● |  |  |
| 105 | CAST1.906 | [chr3:55552138-55552537](http://genome.ucsc.edu/cgi-bin/hgTracks?db=hg17&position=chr3:55552138-55552537&hgt.customText=http://www.neurogenome.org/mcs/tracks/chr3.txt) | 400 | 2b | N/A |  |  | ● | ● |  |  |
| 106 | CAST1.916 | [chr3:55547588-55548033](http://genome.ucsc.edu/cgi-bin/hgTracks?db=hg17&position=chr3:55547588-55548033&hgt.customText=http://www.neurogenome.org/mcs/tracks/chr3.txt) | 446 | 2b | N/A |  |  |  | ● | ● |  |
| 107 | CAST1.938 | [chr3:55537033-55537507](http://genome.ucsc.edu/cgi-bin/hgTracks?db=hg17&position=chr3:55537033-55537507&hgt.customText=http://www.neurogenome.org/mcs/tracks/chr3.txt) | 475 | 2b | N/A |  |  | ● | ● |  |  |
| 108 | CAST1.951 | [chr3:55527538-55528017](http://genome.ucsc.edu/cgi-bin/hgTracks?db=hg17&position=chr3:55527538-55528017&hgt.customText=http://www.neurogenome.org/mcs/tracks/chr3.txt) | 480 | 2b | N/A |  | ● | ● | ● | ● |  |
| 109 | CAST1.980 | [chr3:55517369-55517958](http://genome.ucsc.edu/cgi-bin/hgTracks?db=hg17&position=chr3:55517369-55517958&hgt.customText=http://www.neurogenome.org/mcs/tracks/chr3.txt) | 590 | 1c (3’) | N/A | ● |  | ● | ● | ● |  |
| 110 | CAST1.989 | [chr3:55505351-55505804](http://genome.ucsc.edu/cgi-bin/hgTracks?db=hg17&position=chr3:55505351-55505804&hgt.customText=http://www.neurogenome.org/mcs/tracks/chr3.txt) | 454 | 2a (3’) | N/A |  |  | ● | ● |  |  |
| 111 | CAST1.991 | [chr3:55500450-55500928](http://genome.ucsc.edu/cgi-bin/hgTracks?db=hg17&position=chr3:55500450-55500928&hgt.customText=http://www.neurogenome.org/mcs/tracks/chr3.txt) | 479 | 2a (3’) | N/A |  |  | ● | ● |  |  |
| 112 | CAST1.993 | [chr3:55497293-55497734](http://genome.ucsc.edu/cgi-bin/hgTracks?db=hg17&position=chr3:55497293-55497734&hgt.customText=http://www.neurogenome.org/mcs/tracks/chr3.txt) | 442 | 2a (3’) | N/A |  | ● | ● | ● |  |  |
| 113 | DMXL2.86 | [chr15:49578869-49579316](http://genome.ucsc.edu/cgi-bin/hgTracks?db=hg17&position=chr15:49578869-49579316&hgt.customText=http://www.neurogenome.org/mcs/tracks/chr15.txt) | 448 | 1a | N/A | ● |  | ● | ● | ● | ● |
| 114 | DMXL2.158 | [chr15:49527274-49527674](http://genome.ucsc.edu/cgi-bin/hgTracks?db=hg17&position=chr15:49527274-49527674&hgt.customText=http://www.neurogenome.org/mcs/tracks/chr15.txt) | 401 | 2a (3’) | N/A | ● |  | ● | ● | ● |  |
| 115 | DNM1.101 | [chr9:128094829-128095257](http://genome.ucsc.edu/cgi-bin/hgTracks?db=hg17&position=chr9:128094829-128095257&hgt.customText=http://www.neurogenome.org/mcs/tracks/chr9.txt) | 429 | 1c (5’) | N/A |  |  | ● | ● |  |  |
| 116 | DNM1.108 | [chr9:128096482-128096918](http://genome.ucsc.edu/cgi-bin/hgTracks?db=hg17&position=chr9:128096482-128096918&hgt.customText=http://www.neurogenome.org/mcs/tracks/chr9.txt) | 437 | 1c (3’) | N/A | ● |  | ● | ● |  |  |
| 117 | EXOC2.39 | [chr6:562480-562841](http://genome.ucsc.edu/cgi-bin/hgTracks?db=hg17&position=chr6:562480-562841&hgt.customText=http://www.neurogenome.org/mcs/tracks/chr6.txt) | 362 | 1b | ● |  | ● | ● | ● |  |  |
| 118 | EXOC3.5 | [chr5:506483-506857](http://genome.ucsc.edu/cgi-bin/hgTracks?db=hg17&position=chr5:506483-506857&hgt.customText=http://www.neurogenome.org/mcs/tracks/chr5.txt) | 375 | 1a | N/A | ● |  | ● | ● | ● | ● |
| 119 | EXOC4.60 | [chr7:132291429-132291831](http://genome.ucsc.edu/cgi-bin/hgTracks?db=hg17&position=chr7:132291429-132291831&hgt.customText=http://www.neurogenome.org/mcs/tracks/chr7.txt) | 403 | 2a (5’) |  |  |  | ● | ● |  |  |
| 120 | EXOC4.70 | [chr7:132303181-132303571](http://genome.ucsc.edu/cgi-bin/hgTracks?db=hg17&position=chr7:132303181-132303571&hgt.customText=http://www.neurogenome.org/mcs/tracks/chr7.txt) | 391 | 2a (5’) |  |  |  | ● | ● |  |  |
| 121 | EXOC4.72 | [chr7:132306918-132307430](http://genome.ucsc.edu/cgi-bin/hgTracks?db=hg17&position=chr7:132306918-132307430&hgt.customText=http://www.neurogenome.org/mcs/tracks/chr7.txt) | 513 | 2a (5’) |  |  | ● | ● | ● | ● |  |
| 122 | EXOC4.84 | [chr7:132311675-132312145](http://genome.ucsc.edu/cgi-bin/hgTracks?db=hg17&position=chr7:132311675-132312145&hgt.customText=http://www.neurogenome.org/mcs/tracks/chr7.txt) | 471 | 2a (5’) | ● | ● | ● | ● | ● | ● | ● |
| 123 | EXOC4.119 | [chr7:132377156-132377564](http://genome.ucsc.edu/cgi-bin/hgTracks?db=hg17&position=chr7:132377156-132377564&hgt.customText=http://www.neurogenome.org/mcs/tracks/chr7.txt) | 409 | 2a (5’) |  |  |  | ● | ● |  |  |
| 124 | EXOC4.155 | [chr7:132415765-132416155](http://genome.ucsc.edu/cgi-bin/hgTracks?db=hg17&position=chr7:132415765-132416155&hgt.customText=http://www.neurogenome.org/mcs/tracks/chr7.txt) | 391 | 2b |  |  |  | ● | ● |  |  |
| 125 | EXOC4.185 | [chr7:132435648-132436283](http://genome.ucsc.edu/cgi-bin/hgTracks?db=hg17&position=chr7:132435648-132436283&hgt.customText=http://www.neurogenome.org/mcs/tracks/chr7.txt) | 636 | 2b |  |  |  | ● | ● | ● |  |
| 126 | EXOC4.196 | [chr7:132446062-132446472](http://genome.ucsc.edu/cgi-bin/hgTracks?db=hg17&position=chr7:132446062-132446472&hgt.customText=http://www.neurogenome.org/mcs/tracks/chr7.txt) | 411 | 2b |  |  |  | ● | ● |  |  |
| 127 | EXOC4.230 | [chr7:132504742-132505247](http://genome.ucsc.edu/cgi-bin/hgTracks?db=hg17&position=chr7:132504742-132505247&hgt.customText=http://www.neurogenome.org/mcs/tracks/chr7.txt) | 506 | 2b |  |  |  | ● | ● |  |  |
| 128 | EXOC4.331 | [chr7:132633049-132633462](http://genome.ucsc.edu/cgi-bin/hgTracks?db=hg17&position=chr7:132633049-132633462&hgt.customText=http://www.neurogenome.org/mcs/tracks/chr7.txt) | 414 | 2b |  |  | ● | ● | ● |  |  |
| 129 | EXOC4.334 | [chr7:132643343-132643796](http://genome.ucsc.edu/cgi-bin/hgTracks?db=hg17&position=chr7:132643343-132643796&hgt.customText=http://www.neurogenome.org/mcs/tracks/chr7.txt) | 454 | 2b |  | ● |  | ● | ● | ● |  |
| 130 | EXOC4.344 | [chr7:132664263-132664661](http://genome.ucsc.edu/cgi-bin/hgTracks?db=hg17&position=chr7:132664263-132664661&hgt.customText=http://www.neurogenome.org/mcs/tracks/chr7.txt) | 399 | 2b |  |  |  | ● | ● |  |  |
| 131 | EXOC4.381 | [chr7:132697977-132698373](http://genome.ucsc.edu/cgi-bin/hgTracks?db=hg17&position=chr7:132697977-132698373&hgt.customText=http://www.neurogenome.org/mcs/tracks/chr7.txt) | 397 | 2b |  | ● |  | ● | ● | ● |  |
| 132 | EXOC4.417 | [chr7:132744221-132744718](http://genome.ucsc.edu/cgi-bin/hgTracks?db=hg17&position=chr7:132744221-132744718&hgt.customText=http://www.neurogenome.org/mcs/tracks/chr7.txt) | 498 | 2b |  |  |  | ● | ● | ● |  |
| 133 | EXOC4.453 | [chr7:132808036-132808417](http://genome.ucsc.edu/cgi-bin/hgTracks?db=hg17&position=chr7:132808036-132808417&hgt.customText=http://www.neurogenome.org/mcs/tracks/chr7.txt) | 382 | 2b | ● |  |  | ● | ● | ● |  |
| 134 | EXOC4.481 | [chr7:132853063-132853545](http://genome.ucsc.edu/cgi-bin/hgTracks?db=hg17&position=chr7:132853063-132853545&hgt.customText=http://www.neurogenome.org/mcs/tracks/chr7.txt) | 483 | 2b | ● |  |  | ● | ● | ● |  |
| 135 | EXOC4.498 | [chr7:132874702-132875220](http://genome.ucsc.edu/cgi-bin/hgTracks?db=hg17&position=chr7:132874702-132875220&hgt.customText=http://www.neurogenome.org/mcs/tracks/chr7.txt) | 519 | 2b | ● |  |  | ● | ● | ● | ● |
| 136 | EXOC4.531 | [chr7:132913381-132913821](http://genome.ucsc.edu/cgi-bin/hgTracks?db=hg17&position=chr7:132913381-132913821&hgt.customText=http://www.neurogenome.org/mcs/tracks/chr7.txt) | 441 | 2b |  |  |  | ● | ● | ● |  |
| 137 | EXOC4.532 | [chr7:132913831-132914226](http://genome.ucsc.edu/cgi-bin/hgTracks?db=hg17&position=chr7:132913831-132914226&hgt.customText=http://www.neurogenome.org/mcs/tracks/chr7.txt) | 396 | 2b |  |  |  | ● | ● | ● |  |
| 138 | EXOC4.548 | [chr7:132924196-132924605](http://genome.ucsc.edu/cgi-bin/hgTracks?db=hg17&position=chr7:132924196-132924605&hgt.customText=http://www.neurogenome.org/mcs/tracks/chr7.txt) | 410 | 2b |  |  | ● | ● | ● |  |  |
| 139 | EXOC4.601 | [chr7:132978607-132979139](http://genome.ucsc.edu/cgi-bin/hgTracks?db=hg17&position=chr7:132978607-132979139&hgt.customText=http://www.neurogenome.org/mcs/tracks/chr7.txt) | 533 | 2b |  |  |  | ● | ● | ● |  |
| 140 | EXOC4.667 | [chr7:133076580-133076996](http://genome.ucsc.edu/cgi-bin/hgTracks?db=hg17&position=chr7:133076580-133076996&hgt.customText=http://www.neurogenome.org/mcs/tracks/chr7.txt) | 417 | 2b |  |  |  | ● | ● |  |  |
| 141 | EXOC4.682 | [chr7:133091340-133091933](http://genome.ucsc.edu/cgi-bin/hgTracks?db=hg17&position=chr7:133091340-133091933&hgt.customText=http://www.neurogenome.org/mcs/tracks/chr7.txt) | 594 | 2b |  |  | ● | ● | ● | ● |  |
| 142 | EXOC4.689 | [chr7:133104660-133105022](http://genome.ucsc.edu/cgi-bin/hgTracks?db=hg17&position=chr7:133104660-133105022&hgt.customText=http://www.neurogenome.org/mcs/tracks/chr7.txt) | 363 | 2b |  |  | ● | ● | ● |  |  |
| 143 | EXOC4.752 | [chr7:133183281-133183875](http://genome.ucsc.edu/cgi-bin/hgTracks?db=hg17&position=chr7:133183281-133183875&hgt.customText=http://www.neurogenome.org/mcs/tracks/chr7.txt) | 595 | 2b |  |  |  | ● | ● | ● |  |
| 144 | EXOC4.767 | [chr7:133196659-133197110](http://genome.ucsc.edu/cgi-bin/hgTracks?db=hg17&position=chr7:133196659-133197110&hgt.customText=http://www.neurogenome.org/mcs/tracks/chr7.txt) | 452 | 2b |  |  |  | ● | ● |  |  |
| 145 | EXOC4.784 | [chr7:133212060-133212496](http://genome.ucsc.edu/cgi-bin/hgTracks?db=hg17&position=chr7:133212060-133212496&hgt.customText=http://www.neurogenome.org/mcs/tracks/chr7.txt) | 437 | 2a (3’) |  |  |  | ● | ● |  |  |
| 146 | EXOC4.792 | [chr7:133240104-133240542](http://genome.ucsc.edu/cgi-bin/hgTracks?db=hg17&position=chr7:133240104-133240542&hgt.customText=http://www.neurogenome.org/mcs/tracks/chr7.txt) | 439 | 2a (3’) |  |  | ● | ● | ● |  |  |
| 147 | EXOC5.56 | [chr14:56726503-56726881](http://genome.ucsc.edu/cgi-bin/hgTracks?db=hg17&position=chr14:56726503-56726881&hgt.customText=http://www.neurogenome.org/mcs/tracks/chr14.txt) | 379 | 2a (3’) |  |  | ● | ● | ● | ● |  |
| 148 | EXOC5.60 | [chr14:56725291-56725789](http://genome.ucsc.edu/cgi-bin/hgTracks?db=hg17&position=chr14:56725291-56725789&hgt.customText=http://www.neurogenome.org/mcs/tracks/chr14.txt) | 499 | 2a (3’) |  |  |  | ● | ● | ● |  |
| 149 | EXOC5.69 | [chr14:56715373-56715779](http://genome.ucsc.edu/cgi-bin/hgTracks?db=hg17&position=chr14:56715373-56715779&hgt.customText=http://www.neurogenome.org/mcs/tracks/chr14.txt) | 407 | 2a (3’) |  |  |  | ● | ● |  |  |
| 150 | EXOC5.70 | [chr14:56710961-56711361](http://genome.ucsc.edu/cgi-bin/hgTracks?db=hg17&position=chr14:56710961-56711361&hgt.customText=http://www.neurogenome.org/mcs/tracks/chr14.txt) | 401 | 2a (3’) |  |  |  | ● | ● | ● |  |
| 151 | EXOC5.74 | [chr14:56707806-56708182](http://genome.ucsc.edu/cgi-bin/hgTracks?db=hg17&position=chr14:56707806-56708182&hgt.customText=http://www.neurogenome.org/mcs/tracks/chr14.txt) | 377 | 2a (3’) |  |  |  | ● | ● |  |  |
| 152 | EXOC5.84 | [chr14:56696256-56696663](http://genome.ucsc.edu/cgi-bin/hgTracks?db=hg17&position=chr14:56696256-56696663&hgt.customText=http://www.neurogenome.org/mcs/tracks/chr14.txt) | 408 | 2a (3’) |  |  | ● | ● | ● | ● |  |
| 153 | EXOC5.105 | [chr14:56657783-56658145](http://genome.ucsc.edu/cgi-bin/hgTracks?db=hg17&position=chr14:56657783-56658145&hgt.customText=http://www.neurogenome.org/mcs/tracks/chr14.txt) | 363 | 2a (3’) |  |  | ● | ● | ● | ● |  |
| 154 | EXOC5.126 | [chr14:56623027-56623548](http://genome.ucsc.edu/cgi-bin/hgTracks?db=hg17&position=chr14:56623027-56623548&hgt.customText=http://www.neurogenome.org/mcs/tracks/chr14.txt) | 522 | 2a (3’) |  |  |  | ● | ● | ● |  |
| 155 | EXOC5.129 | [chr14:56622195-56622570](http://genome.ucsc.edu/cgi-bin/hgTracks?db=hg17&position=chr14:56622195-56622570&hgt.customText=http://www.neurogenome.org/mcs/tracks/chr14.txt) | 376 | 2a (3’) |  |  | ● | ● | ● |  |  |
| 156 | EXOC5.133 | [chr14:56617102-56617551](http://genome.ucsc.edu/cgi-bin/hgTracks?db=hg17&position=chr14:56617102-56617551&hgt.customText=http://www.neurogenome.org/mcs/tracks/chr14.txt) | 450 | 2a (3’) |  |  |  | ● | ● | ● |  |
| 157 | EXOC5.136 | [chr14:56608101-56608480](http://genome.ucsc.edu/cgi-bin/hgTracks?db=hg17&position=chr14:56608101-56608480&hgt.customText=http://www.neurogenome.org/mcs/tracks/chr14.txt) | 380 | 2a (3’) |  |  | ● | ● | ● | ● |  |
| 158 | EXOC5.168 | [chr14:56582111-56582623](http://genome.ucsc.edu/cgi-bin/hgTracks?db=hg17&position=chr14:56582111-56582623&hgt.customText=http://www.neurogenome.org/mcs/tracks/chr14.txt) | 513 | 2a (3’) |  |  |  | ● | ● |  |  |
| 159 | EXOC5.184 | [chr14:56545923-56546464](http://genome.ucsc.edu/cgi-bin/hgTracks?db=hg17&position=chr14:56545923-56546464&hgt.customText=http://www.neurogenome.org/mcs/tracks/chr14.txt) | 542 | 2a (3’) |  |  |  | ● | ● | ● | ● |
| 160 | EXOC5.187 | [chr14:56545026-56545508](http://genome.ucsc.edu/cgi-bin/hgTracks?db=hg17&position=chr14:56545026-56545508&hgt.customText=http://www.neurogenome.org/mcs/tracks/chr14.txt) | 483 | 2a (3’) |  |  |  | ● | ● | ● |  |
| 161 | EXOC5.192 | [chr14:56539836-56540345](http://genome.ucsc.edu/cgi-bin/hgTracks?db=hg17&position=chr14:56539836-56540345&hgt.customText=http://www.neurogenome.org/mcs/tracks/chr14.txt) | 510 | 2a (3’) |  |  | ● | ● | ● | ● |  |
| 162 | EXOC5.199 | [chr14:56533474-56533933](http://genome.ucsc.edu/cgi-bin/hgTracks?db=hg17&position=chr14:56533474-56533933&hgt.customText=http://www.neurogenome.org/mcs/tracks/chr14.txt) | 460 | 2a (3’) |  |  |  | ● | ● | ● |  |
| 163 | EXOC5.211 | [chr14:56518513-56518948](http://genome.ucsc.edu/cgi-bin/hgTracks?db=hg17&position=chr14:56518513-56518948&hgt.customText=http://www.neurogenome.org/mcs/tracks/chr14.txt) | 436 | 2a (3’) |  |  |  | ● | ● | ● |  |
| 164 | EXOC5.217 | [chr14:56514882-56515499](http://genome.ucsc.edu/cgi-bin/hgTracks?db=hg17&position=chr14:56514882-56515499&hgt.customText=http://www.neurogenome.org/mcs/tracks/chr14.txt) | 618 | 2a (3’) | ● |  | ● | ● | ● | ● | ● |
| 165 | EXOC5.222 | [chr14:56500959-56501738](http://genome.ucsc.edu/cgi-bin/hgTracks?db=hg17&position=chr14:56500959-56501738&hgt.customText=http://www.neurogenome.org/mcs/tracks/chr14.txt) | 780 | 2a (3’) |  |  |  | ● | ● | ● |  |
| 166 | EXOC5.224 | [chr14:56498964-56499551](http://genome.ucsc.edu/cgi-bin/hgTracks?db=hg17&position=chr14:56498964-56499551&hgt.customText=http://www.neurogenome.org/mcs/tracks/chr14.txt) | 588 | 2a (3’) |  |  |  | ● | ● | ● |  |
| 167 | EXOC5.240 | [chr14:56490228-56490836](http://genome.ucsc.edu/cgi-bin/hgTracks?db=hg17&position=chr14:56490228-56490836&hgt.customText=http://www.neurogenome.org/mcs/tracks/chr14.txt) | 609 | 2a (3’) |  |  |  | ● | ● | ● |  |
| 168 | EXOC5.270 | [chr14:56439941-56440348](http://genome.ucsc.edu/cgi-bin/hgTracks?db=hg17&position=chr14:56439941-56440348&hgt.customText=http://www.neurogenome.org/mcs/tracks/chr14.txt) | 408 | 2a (3’) |  |  | ● | ● | ● | ● |  |
| 169 | EXOC5.272 | [chr14:56438798-56439410](http://genome.ucsc.edu/cgi-bin/hgTracks?db=hg17&position=chr14:56438798-56439410&hgt.customText=http://www.neurogenome.org/mcs/tracks/chr14.txt) | 613 | 2a (3’) |  |  |  | ● | ● | ● |  |
| 170 | EXOC5.277 | [chr14:56436213-56436614](http://genome.ucsc.edu/cgi-bin/hgTracks?db=hg17&position=chr14:56436213-56436614&hgt.customText=http://www.neurogenome.org/mcs/tracks/chr14.txt) | 402 | 2a (3’) |  |  |  | ● | ● | ● |  |
| 171 | EXOC5.279 | [chr14:56435450-56436027](http://genome.ucsc.edu/cgi-bin/hgTracks?db=hg17&position=chr14:56435450-56436027&hgt.customText=http://www.neurogenome.org/mcs/tracks/chr14.txt) | 578 | 2a (3’) |  |  | ● | ● | ● | ● |  |
| 172 | EXOC5.286 | [chr14:56427605-56428214](http://genome.ucsc.edu/cgi-bin/hgTracks?db=hg17&position=chr14:56427605-56428214&hgt.customText=http://www.neurogenome.org/mcs/tracks/chr14.txt) | 610 | 2a (3’) |  |  | ● | ● | ● | ● |  |
| 173 | EXOC5.290 | [chr14:56424726-56425591](http://genome.ucsc.edu/cgi-bin/hgTracks?db=hg17&position=chr14:56424726-56425591&hgt.customText=http://www.neurogenome.org/mcs/tracks/chr14.txt) | 866 | 2a (3’) |  |  | ● | ● | ● | ● |  |
| 174 | EXOC5.294 | [chr14:56422733-56423319](http://genome.ucsc.edu/cgi-bin/hgTracks?db=hg17&position=chr14:56422733-56423319&hgt.customText=http://www.neurogenome.org/mcs/tracks/chr14.txt) | 587 | 2a (3’) |  |  |  | ● | ● |  |  |
| 175 | EXOC5.318 | [chr14:56392073-56392564](http://genome.ucsc.edu/cgi-bin/hgTracks?db=hg17&position=chr14:56392073-56392564&hgt.customText=http://www.neurogenome.org/mcs/tracks/chr14.txt) | 492 | 2a (3’) |  |  | ● | ● | ● | ● |  |
| 176 | EXOC5.322 | [chr14:56390455-56390868](http://genome.ucsc.edu/cgi-bin/hgTracks?db=hg17&position=chr14:56390455-56390868&hgt.customText=http://www.neurogenome.org/mcs/tracks/chr14.txt) | 414 | 2a (3’) |  |  |  | ● | ● | ● |  |
| 177 | EXOC5.330 | [chr14:56382466-56383006](http://genome.ucsc.edu/cgi-bin/hgTracks?db=hg17&position=chr14:56382466-56383006&hgt.customText=http://www.neurogenome.org/mcs/tracks/chr14.txt) | 541 | 2a (3’) |  |  | ● | ● | ● |  |  |
| 178 | EXOC5.338 | [chr14:56365466-56366003](http://genome.ucsc.edu/cgi-bin/hgTracks?db=hg17&position=chr14:56365466-56366003&hgt.customText=http://www.neurogenome.org/mcs/tracks/chr14.txt) | 538 | 2a (3’) |  |  |  | ● | ● |  |  |
| 179 | EXOC5.349 | [chr14:56351303-56351828](http://genome.ucsc.edu/cgi-bin/hgTracks?db=hg17&position=chr14:56351303-56351828&hgt.customText=http://www.neurogenome.org/mcs/tracks/chr14.txt) | 526 | 2a (3’) |  | ● |  | ● | ● |  |  |
| 180 | EXOC5.359 | [chr14:56349318-56349993](http://genome.ucsc.edu/cgi-bin/hgTracks?db=hg17&position=chr14:56349318-56349993&hgt.customText=http://www.neurogenome.org/mcs/tracks/chr14.txt) | 676 | 2a (3’) |  | ● |  | ● | ● | ● |  |
| 181 | EXOC6.5 | [chr10:94447448-94447966](http://genome.ucsc.edu/cgi-bin/hgTracks?db=hg17&position=chr10:94447448-94447966&hgt.customText=http://www.neurogenome.org/mcs/tracks/chr10.txt) | 519 | 2a (5’) | N/A |  |  | ● | ● | ● |  |
| 182 | EXOC6.56 | [chr10:94549614-94550049](http://genome.ucsc.edu/cgi-bin/hgTracks?db=hg17&position=chr10:94549614-94550049&hgt.customText=http://www.neurogenome.org/mcs/tracks/chr10.txt) | 436 | 2a (5’) | N/A |  | ● | ● | ● | ● |  |
| 183 | EXOC6.107 | [chr10:94627593-94628062](http://genome.ucsc.edu/cgi-bin/hgTracks?db=hg17&position=chr10:94627593-94628062&hgt.customText=http://www.neurogenome.org/mcs/tracks/chr10.txt) | 470 | 2b | N/A |  |  | ● | ● | ● | ● |
| 184 | EXOC6.121 | [chr10:94644177-94644586](http://genome.ucsc.edu/cgi-bin/hgTracks?db=hg17&position=chr10:94644177-94644586&hgt.customText=http://www.neurogenome.org/mcs/tracks/chr10.txt) | 410 | 1b | N/A |  |  | ● | ● | ● | ● |
| 185 | EXOC6.163 | [chr10:94743447-94744183](http://genome.ucsc.edu/cgi-bin/hgTracks?db=hg17&position=chr10:94743447-94744183&hgt.customText=http://www.neurogenome.org/mcs/tracks/chr10.txt) | 737 | 2b | N/A |  |  | ● | ● | ● |  |
| 186 | EXOC6.183 | [chr10:94772750-94773212](http://genome.ucsc.edu/cgi-bin/hgTracks?db=hg17&position=chr10:94772750-94773212&hgt.customText=http://www.neurogenome.org/mcs/tracks/chr10.txt) | 463 | 2b | N/A |  |  | ● | ● | ● |  |
| 187 | EXOC6.197 | [chr10:94801619-94802036](http://genome.ucsc.edu/cgi-bin/hgTracks?db=hg17&position=chr10:94801619-94802036&hgt.customText=http://www.neurogenome.org/mcs/tracks/chr10.txt) | 418 | 2b | N/A |  | ● | ● | ● |  |  |
| 188 | EXOC8.5 | [chr1:227778045-227779248](http://genome.ucsc.edu/cgi-bin/hgTracks?db=hg17&position=chr1:227778045-227779248&hgt.customText=http://www.neurogenome.org/mcs/tracks/chr1.txt) | 1204 | 1a | N/A | ● | ● | ● | ● | ● | ● |
| 189 | GZMB.36 | [chr14:24251094-24251464](http://genome.ucsc.edu/cgi-bin/hgTracks?db=hg17&position=chr14:24251094-24251464&hgt.customText=http://www.neurogenome.org/mcs/tracks/chr14.txt) | 371 | 2a (5’) |  |  |  | ● | ● |  |  |
| 190 | NAPG.38 | [chr18:10613870-10614303](http://genome.ucsc.edu/cgi-bin/hgTracks?db=hg17&position=chr18:10613870-10614303&hgt.customText=http://www.neurogenome.org/mcs/tracks/chr18.txt) | 434 | 2a (3’) | N/A |  |  | ● | ● |  | ● |
| 191 | NAPG.40 | [chr18:10626953-10627352](http://genome.ucsc.edu/cgi-bin/hgTracks?db=hg17&position=chr18:10626953-10627352&hgt.customText=http://www.neurogenome.org/mcs/tracks/chr18.txt) | 400 | 2a (3’) | N/A |  | ● | ● | ● |  | ● |
| 192 | NBEA.67 | [chr13:33636556-33636956](http://genome.ucsc.edu/cgi-bin/hgTracks?db=hg17&position=chr13:33636556-33636956&hgt.customText=http://www.neurogenome.org/mcs/tracks/chr13.txt) | 401 | 2a (5’) |  |  |  | ● | ● |  |  |
| 193 | NBEA.83 | [chr13:33682957-33683329](http://genome.ucsc.edu/cgi-bin/hgTracks?db=hg17&position=chr13:33682957-33683329&hgt.customText=http://www.neurogenome.org/mcs/tracks/chr13.txt) | 373 | 2a (5’) |  |  |  | ● | ● |  |  |
| 194 | NBEA.352 | [chr13:34210703-34211210](http://genome.ucsc.edu/cgi-bin/hgTracks?db=hg17&position=chr13:34210703-34211210&hgt.customText=http://www.neurogenome.org/mcs/tracks/chr13.txt) | 508 | 2a (5’) | ● |  | ● | ● | ● |  |  |
| 195 | NBEA.500 | [chr13:34567967-34568358](http://genome.ucsc.edu/cgi-bin/hgTracks?db=hg17&position=chr13:34567967-34568358&hgt.customText=http://www.neurogenome.org/mcs/tracks/chr13.txt) | 392 | 2b |  |  |  | ● | ● | ● |  |
| 196 | NBEA.550 | [chr13:34670832-34671301](http://genome.ucsc.edu/cgi-bin/hgTracks?db=hg17&position=chr13:34670832-34671301&hgt.customText=http://www.neurogenome.org/mcs/tracks/chr13.txt) | 470 | 2b |  |  |  | ● | ● | ● | ● |
| 197 | NBEA.619 | [chr13:34860666-34861078](http://genome.ucsc.edu/cgi-bin/hgTracks?db=hg17&position=chr13:34860666-34861078&hgt.customText=http://www.neurogenome.org/mcs/tracks/chr13.txt) | 413 | 2b |  |  | ● | ● | ● | ● |  |
| 198 | NBEA.631 | [chr13:34914717-34915169](http://genome.ucsc.edu/cgi-bin/hgTracks?db=hg17&position=chr13:34914717-34915169&hgt.customText=http://www.neurogenome.org/mcs/tracks/chr13.txt) | 453 | 2b |  |  |  | ● | ● | ● | ● |
| 199 | NBEA.682 | [chr13:34956587-34956992](http://genome.ucsc.edu/cgi-bin/hgTracks?db=hg17&position=chr13:34956587-34956992&hgt.customText=http://www.neurogenome.org/mcs/tracks/chr13.txt) | 406 | 2b |  |  |  | ● | ● | ● |  |
| 200 | NBEA.694 | [chr13:34971557-34972104](http://genome.ucsc.edu/cgi-bin/hgTracks?db=hg17&position=chr13:34971557-34972104&hgt.customText=http://www.neurogenome.org/mcs/tracks/chr13.txt) | 548 | 2b |  |  |  | ● | ● | ● | ● |
| 201 | NBEA.695 | [chr13:34972115-34972843](http://genome.ucsc.edu/cgi-bin/hgTracks?db=hg17&position=chr13:34972115-34972843&hgt.customText=http://www.neurogenome.org/mcs/tracks/chr13.txt) | 729 | 2b |  |  |  | ● | ● | ● |  |
| 202 | NBEA.702 | [chr13:34986252-34986629](http://genome.ucsc.edu/cgi-bin/hgTracks?db=hg17&position=chr13:34986252-34986629&hgt.customText=http://www.neurogenome.org/mcs/tracks/chr13.txt) | 378 | 2b |  |  |  | ● | ● | ● |  |
| 203 | NBEA.704 | [chr13:34986907-34987444](http://genome.ucsc.edu/cgi-bin/hgTracks?db=hg17&position=chr13:34986907-34987444&hgt.customText=http://www.neurogenome.org/mcs/tracks/chr13.txt) | 538 | 2b |  |  |  | ● | ● | ● | ● |
| 204 | NBEA.715 | [chr13:35003739-35004135](http://genome.ucsc.edu/cgi-bin/hgTracks?db=hg17&position=chr13:35003739-35004135&hgt.customText=http://www.neurogenome.org/mcs/tracks/chr13.txt) | 397 | 2b |  |  |  | ● | ● | ● | ● |
| 205 | NBEA.763 | [chr13:35068826-35069498](http://genome.ucsc.edu/cgi-bin/hgTracks?db=hg17&position=chr13:35068826-35069498&hgt.customText=http://www.neurogenome.org/mcs/tracks/chr13.txt) | 673 | 2b |  |  |  | ● | ● | ● |  |
| 206 | NBEA.817 | [chr13:35131971-35132431](http://genome.ucsc.edu/cgi-bin/hgTracks?db=hg17&position=chr13:35131971-35132431&hgt.customText=http://www.neurogenome.org/mcs/tracks/chr13.txt) | 461 | 2b |  |  |  | ● | ● | ● |  |
| 207 | NBEA.826 | [chr13:35143035-35143918](http://genome.ucsc.edu/cgi-bin/hgTracks?db=hg17&position=chr13:35143035-35143918&hgt.customText=http://www.neurogenome.org/mcs/tracks/chr13.txt) | 884 | 1c (3’) | ● | ● |  | ● | ● | ● |  |
| 208 | NBEA.830 | [chr13:35144210-35144877](http://genome.ucsc.edu/cgi-bin/hgTracks?db=hg17&position=chr13:35144210-35144877&hgt.customText=http://www.neurogenome.org/mcs/tracks/chr13.txt) | 668 | 1c (3’) | ● | ● |  | ● | ● | ● | ● |
| 209 | NBEA.856 | [chr13:35195147-35195536](http://genome.ucsc.edu/cgi-bin/hgTracks?db=hg17&position=chr13:35195147-35195536&hgt.customText=http://www.neurogenome.org/mcs/tracks/chr13.txt) | 390 | 2a (3’) |  |  |  | ● | ● |  |  |
| 210 | NCAM1.4 | [chr11:111643233-111643865](http://genome.ucsc.edu/cgi-bin/hgTracks?db=hg17&position=chr11:111643233-111643865&hgt.customText=http://www.neurogenome.org/mcs/tracks/chr11.txt) | 633 | 2a (5’) | N/A |  |  | ● | ● | ● | ● |
| 211 | NCAM1.26 | [chr11:111682322-111682778](http://genome.ucsc.edu/cgi-bin/hgTracks?db=hg17&position=chr11:111682322-111682778&hgt.customText=http://www.neurogenome.org/mcs/tracks/chr11.txt) | 457 | 2a (5’) | N/A |  | ● | ● | ● |  |  |
| 212 | NCAM1.95 | [chr11:111796732-111797168](http://genome.ucsc.edu/cgi-bin/hgTracks?db=hg17&position=chr11:111796732-111797168&hgt.customText=http://www.neurogenome.org/mcs/tracks/chr11.txt) | 437 | 2a (5’) | N/A |  |  | ● | ● |  |  |
| 213 | NCAM1.134 | [chr11:111864267-111864660](http://genome.ucsc.edu/cgi-bin/hgTracks?db=hg17&position=chr11:111864267-111864660&hgt.customText=http://www.neurogenome.org/mcs/tracks/chr11.txt) | 394 | 2a (5’) | N/A |  |  | ● | ● | ● |  |
| 214 | NCAM1.284 | [chr11:112027301-112027805](http://genome.ucsc.edu/cgi-bin/hgTracks?db=hg17&position=chr11:112027301-112027805&hgt.customText=http://www.neurogenome.org/mcs/tracks/chr11.txt) | 505 | 2a (5’) | N/A |  | ● | ● | ● |  |  |
| 215 | NCAM1.296 | [chr11:112041225-112041588](http://genome.ucsc.edu/cgi-bin/hgTracks?db=hg17&position=chr11:112041225-112041588&hgt.customText=http://www.neurogenome.org/mcs/tracks/chr11.txt) | 364 | 2a (5’) | N/A |  |  | ● | ● |  |  |
| 216 | NCAM1.308 | [chr11:112073354-112073713](http://genome.ucsc.edu/cgi-bin/hgTracks?db=hg17&position=chr11:112073354-112073713&hgt.customText=http://www.neurogenome.org/mcs/tracks/chr11.txt) | 360 | 2a (5’) | N/A |  | ● | ● | ● |  |  |
| 217 | NCAM1.369 | [chr11:112153231-112153665](http://genome.ucsc.edu/cgi-bin/hgTracks?db=hg17&position=chr11:112153231-112153665&hgt.customText=http://www.neurogenome.org/mcs/tracks/chr11.txt) | 435 | 2a (5’) | N/A |  |  | ● | ● |  |  |
| 218 | NCAM1.373 | [chr11:112154613-112154991](http://genome.ucsc.edu/cgi-bin/hgTracks?db=hg17&position=chr11:112154613-112154991&hgt.customText=http://www.neurogenome.org/mcs/tracks/chr11.txt) | 379 | 2a (5’) | N/A |  |  | ● | ● | ● |  |
| 219 | NCAM1.415 | [chr11:112190005-112190377](http://genome.ucsc.edu/cgi-bin/hgTracks?db=hg17&position=chr11:112190005-112190377&hgt.customText=http://www.neurogenome.org/mcs/tracks/chr11.txt) | 373 | 2a (5’) | N/A |  |  | ● | ● | ● |  |
| 220 | NCAM1.512 | [chr11:112286984-112287439](http://genome.ucsc.edu/cgi-bin/hgTracks?db=hg17&position=chr11:112286984-112287439&hgt.customText=http://www.neurogenome.org/mcs/tracks/chr11.txt) | 456 | 2a (5’) | N/A |  |  | ● | ● |  |  |
| 221 | NCAM1.552 | [chr11:112337140-112337605](http://genome.ucsc.edu/cgi-bin/hgTracks?db=hg17&position=chr11:112337140-112337605&hgt.customText=http://www.neurogenome.org/mcs/tracks/chr11.txt) | 466 | 1c (5’) | N/A | ● |  | ● | ● |  |  |
| 222 | NCAM1.555 | [chr11:112338117-112338588](http://genome.ucsc.edu/cgi-bin/hgTracks?db=hg17&position=chr11:112338117-112338588&hgt.customText=http://www.neurogenome.org/mcs/tracks/chr11.txt) | 472 | 2b | N/A |  |  | ● | ● |  |  |
| 223 | NCAM1.582 | [chr11:112353553-112353966](http://genome.ucsc.edu/cgi-bin/hgTracks?db=hg17&position=chr11:112353553-112353966&hgt.customText=http://www.neurogenome.org/mcs/tracks/chr11.txt) | 414 | 2b | N/A |  |  | ● | ● | ● |  |
| 224 | NCAM1.651 | [chr11:112458094-112458606](http://genome.ucsc.edu/cgi-bin/hgTracks?db=hg17&position=chr11:112458094-112458606&hgt.customText=http://www.neurogenome.org/mcs/tracks/chr11.txt) | 513 | 2b | N/A |  |  | ● | ● |  |  |
| 225 | NCAM1.717 | [chr11:112521860-112522386](http://genome.ucsc.edu/cgi-bin/hgTracks?db=hg17&position=chr11:112521860-112522386&hgt.customText=http://www.neurogenome.org/mcs/tracks/chr11.txt) | 527 | 2b | N/A |  |  | ● | ● |  |  |
| 226 | NCAM1.718 | [chr11:112523154-112523575](http://genome.ucsc.edu/cgi-bin/hgTracks?db=hg17&position=chr11:112523154-112523575&hgt.customText=http://www.neurogenome.org/mcs/tracks/chr11.txt) | 422 | 2b | N/A |  |  | ● | ● |  |  |
| 227 | NCAM1.743 | [chr11:112548433-112548940](http://genome.ucsc.edu/cgi-bin/hgTracks?db=hg17&position=chr11:112548433-112548940&hgt.customText=http://www.neurogenome.org/mcs/tracks/chr11.txt) | 508 | 2b | N/A |  |  | ● | ● |  |  |
| 228 | NCAM1.843 | [chr11:112621951-112622325](http://genome.ucsc.edu/cgi-bin/hgTracks?db=hg17&position=chr11:112621951-112622325&hgt.customText=http://www.neurogenome.org/mcs/tracks/chr11.txt) | 375 | 2b | N/A |  |  | ● | ● | ● | ● |
| 229 | NLGN1.171 | [chr3:174710984-174711351](http://genome.ucsc.edu/cgi-bin/hgTracks?db=hg17&position=chr3:174710984-174711351&hgt.customText=http://www.neurogenome.org/mcs/tracks/chr3.txt) | 368 | 2b | N/A |  |  | ● | ● | ● |  |
| 230 | NLGN1.216 | [chr3:174789008-174789569](http://genome.ucsc.edu/cgi-bin/hgTracks?db=hg17&position=chr3:174789008-174789569&hgt.customText=http://www.neurogenome.org/mcs/tracks/chr3.txt) | 562 | 2b | N/A |  |  | ● | ● | ● |  |
| 231 | NLGN1.238 | [chr3:174805224-174805612](http://genome.ucsc.edu/cgi-bin/hgTracks?db=hg17&position=chr3:174805224-174805612&hgt.customText=http://www.neurogenome.org/mcs/tracks/chr3.txt) | 389 | 1a | N/A | ● |  | ● | ● | ● | ● |
| 232 | NLGN1.317 | [chr3:174904120-174904582](http://genome.ucsc.edu/cgi-bin/hgTracks?db=hg17&position=chr3:174904120-174904582&hgt.customText=http://www.neurogenome.org/mcs/tracks/chr3.txt) | 463 | 2b | N/A |  |  | ● | ● | ● |  |
| 233 | NLGN1.320 | [chr3:174908166-174908625](http://genome.ucsc.edu/cgi-bin/hgTracks?db=hg17&position=chr3:174908166-174908625&hgt.customText=http://www.neurogenome.org/mcs/tracks/chr3.txt) | 460 | 2b | N/A |  |  | ● | ● | ● |  |
| 234 | NLGN1.417 | [chr3:175091026-175091481](http://genome.ucsc.edu/cgi-bin/hgTracks?db=hg17&position=chr3:175091026-175091481&hgt.customText=http://www.neurogenome.org/mcs/tracks/chr3.txt) | 456 | 2b | N/A |  |  | ● | ● |  |  |
| 235 | NLGN1.431 | [chr3:175111846-175112407](http://genome.ucsc.edu/cgi-bin/hgTracks?db=hg17&position=chr3:175111846-175112407&hgt.customText=http://www.neurogenome.org/mcs/tracks/chr3.txt) | 562 | 2b | N/A |  |  | ● | ● |  |  |
| 236 | NLGN1.459 | [chr3:175145759-175146186](http://genome.ucsc.edu/cgi-bin/hgTracks?db=hg17&position=chr3:175145759-175146186&hgt.customText=http://www.neurogenome.org/mcs/tracks/chr3.txt) | 428 | 2b | N/A |  |  | ● | ● |  |  |
| 237 | NLGN1.468 | [chr3:175163219-175163615](http://genome.ucsc.edu/cgi-bin/hgTracks?db=hg17&position=chr3:175163219-175163615&hgt.customText=http://www.neurogenome.org/mcs/tracks/chr3.txt) | 397 | 2b | N/A |  |  | ● | ● |  |  |
| 238 | NLGN1.503 | [chr3:175223999-175224393](http://genome.ucsc.edu/cgi-bin/hgTracks?db=hg17&position=chr3:175223999-175224393&hgt.customText=http://www.neurogenome.org/mcs/tracks/chr3.txt) | 395 | 2b | N/A | ● | ● | ● | ● |  |  |
| 239 | NLGN1.530 | [chr3:175261569-175261983](http://genome.ucsc.edu/cgi-bin/hgTracks?db=hg17&position=chr3:175261569-175261983&hgt.customText=http://www.neurogenome.org/mcs/tracks/chr3.txt) | 415 | 2b | N/A |  |  | ● | ● | ● |  |
| 240 | NLGN1.588 | [chr3:175332927-175333286](http://genome.ucsc.edu/cgi-bin/hgTracks?db=hg17&position=chr3:175332927-175333286&hgt.customText=http://www.neurogenome.org/mcs/tracks/chr3.txt) | 360 | 2b | N/A |  |  | ● | ● |  |  |
| 241 | NLGN1.598 | [chr3:175355643-175356006](http://genome.ucsc.edu/cgi-bin/hgTracks?db=hg17&position=chr3:175355643-175356006&hgt.customText=http://www.neurogenome.org/mcs/tracks/chr3.txt) | 364 | 2b | N/A |  |  | ● | ● | ● |  |
| 242 | NLGN1.605 | [chr3:175378678-175379066](http://genome.ucsc.edu/cgi-bin/hgTracks?db=hg17&position=chr3:175378678-175379066&hgt.customText=http://www.neurogenome.org/mcs/tracks/chr3.txt) | 389 | 2b | N/A |  |  | ● | ● |  |  |
| 243 | NLGN1.614 | [chr3:175390024-175390535](http://genome.ucsc.edu/cgi-bin/hgTracks?db=hg17&position=chr3:175390024-175390535&hgt.customText=http://www.neurogenome.org/mcs/tracks/chr3.txt) | 512 | 2b | N/A |  |  | ● | ● | ● |  |
| 244 | NLGN1.630 | [chr3:175418423-175418795](http://genome.ucsc.edu/cgi-bin/hgTracks?db=hg17&position=chr3:175418423-175418795&hgt.customText=http://www.neurogenome.org/mcs/tracks/chr3.txt) | 373 | 2b | N/A |  |  | ● | ● |  |  |
| 245 | NLGN1.634 | [chr3:175421383-175421760](http://genome.ucsc.edu/cgi-bin/hgTracks?db=hg17&position=chr3:175421383-175421760&hgt.customText=http://www.neurogenome.org/mcs/tracks/chr3.txt) | 378 | 2b | N/A |  |  | ● | ● | ● |  |
| 246 | NLGN1.653 | [chr3:175450087-175450490](http://genome.ucsc.edu/cgi-bin/hgTracks?db=hg17&position=chr3:175450087-175450490&hgt.customText=http://www.neurogenome.org/mcs/tracks/chr3.txt) | 404 | 2b | N/A |  |  | ● | ● |  |  |
| 247 | NLGN1.657 | [chr3:175453804-175454305](http://genome.ucsc.edu/cgi-bin/hgTracks?db=hg17&position=chr3:175453804-175454305&hgt.customText=http://www.neurogenome.org/mcs/tracks/chr3.txt) | 502 | 2b | N/A |  |  | ● | ● |  |  |
| 248 | NLGN1.667 | [chr3:175479351-175480144](http://genome.ucsc.edu/cgi-bin/hgTracks?db=hg17&position=chr3:175479351-175480144&hgt.customText=http://www.neurogenome.org/mcs/tracks/chr3.txt) | 794 | 1a | N/A | ● |  | ● | ● | ● | ● |
| 249 | NLGN1.668 | [chr3:175480970-175481798](http://genome.ucsc.edu/cgi-bin/hgTracks?db=hg17&position=chr3:175480970-175481798&hgt.customText=http://www.neurogenome.org/mcs/tracks/chr3.txt) | 829 | 1a | N/A | ● |  | ● | ● | ● | ● |
| 250 | NLGN1.673 | [chr3:175482977-175483609](http://genome.ucsc.edu/cgi-bin/hgTracks?db=hg17&position=chr3:175482977-175483609&hgt.customText=http://www.neurogenome.org/mcs/tracks/chr3.txt) | 633 | 1c (3’) | N/A | ● |  | ● | ● | ● |  |
| 251 | NLGN1.688 | [chr3:175514535-175514956](http://genome.ucsc.edu/cgi-bin/hgTracks?db=hg17&position=chr3:175514535-175514956&hgt.customText=http://www.neurogenome.org/mcs/tracks/chr3.txt) | 422 | 2a (3’) | N/A |  | ● | ● | ● |  |  |
| 252 | NLGN1.697 | [chr3:175536374-175536733](http://genome.ucsc.edu/cgi-bin/hgTracks?db=hg17&position=chr3:175536374-175536733&hgt.customText=http://www.neurogenome.org/mcs/tracks/chr3.txt) | 360 | 2a (3’) | N/A |  |  | ● | ● |  |  |
| 253 | NLGN1.716 | [chr3:175577864-175578496](http://genome.ucsc.edu/cgi-bin/hgTracks?db=hg17&position=chr3:175577864-175578496&hgt.customText=http://www.neurogenome.org/mcs/tracks/chr3.txt) | 633 | 2a (3’) | N/A |  |  | ● | ● | ● | ● |
| 254 | NLGN2.2 | [chr17:7248627-7249031](http://genome.ucsc.edu/cgi-bin/hgTracks?db=hg17&position=chr17:7248627-7249031&hgt.customText=http://www.neurogenome.org/mcs/tracks/chr17.txt) | 405 | 2a (5’) | N/A |  |  | ● | ● |  |  |
| 255 | NLGN2.8 | [chr17:7249595-7250084](http://genome.ucsc.edu/cgi-bin/hgTracks?db=hg17&position=chr17:7249595-7250084&hgt.customText=http://www.neurogenome.org/mcs/tracks/chr17.txt) | 490 | 2a (5’) | N/A |  | ● |  | ● |  |  |
| 256 | NLGN2.19 | [chr17:7252115-7252642](http://genome.ucsc.edu/cgi-bin/hgTracks?db=hg17&position=chr17:7252115-7252642&hgt.customText=http://www.neurogenome.org/mcs/tracks/chr17.txt) | 528 | 1c (5’) | N/A | ● | ● |  | ● | ● | ● |
| 257 | NLGN2.26 | [chr17:7255901-7256454](http://genome.ucsc.edu/cgi-bin/hgTracks?db=hg17&position=chr17:7255901-7256454&hgt.customText=http://www.neurogenome.org/mcs/tracks/chr17.txt) | 554 | 1b | N/A |  |  | ● | ● |  |  |
| 258 | NLGN2.37 | [chr17:7259524-7260155](http://genome.ucsc.edu/cgi-bin/hgTracks?db=hg17&position=chr17:7259524-7260155&hgt.customText=http://www.neurogenome.org/mcs/tracks/chr17.txt) | 632 | 1a | N/A | ● | ● | ● | ● | ● | ● |
| 259 | NLGN3.9 | [chrX:70148342-70148974](http://genome.ucsc.edu/cgi-bin/hgTracks?db=hg17&position=chrX:70148342-70148974&hgt.customText=http://www.neurogenome.org/mcs/tracks/chrX.txt) | 633 | 2b |  |  | ● | ● | ● |  |  |
| 260 | NLGN3.18 | [chrX:70150744-70151489](http://genome.ucsc.edu/cgi-bin/hgTracks?db=hg17&position=chrX:70150744-70151489&hgt.customText=http://www.neurogenome.org/mcs/tracks/chrX.txt) | 746 | 1b | ● | ● |  | ● | ● | ● | ● |
| 261 | NLGN3.19 | [chrX:70151520-70151898](http://genome.ucsc.edu/cgi-bin/hgTracks?db=hg17&position=chrX:70151520-70151898&hgt.customText=http://www.neurogenome.org/mcs/tracks/chrX.txt) | 379 | 1b | ● |  |  | ● | ● | ● |  |
| 262 | NLGN3.31 | [chrX:70156026-70156632](http://genome.ucsc.edu/cgi-bin/hgTracks?db=hg17&position=chrX:70156026-70156632&hgt.customText=http://www.neurogenome.org/mcs/tracks/chrX.txt) | 607 | 1b | ● |  | ● |  | ● | ● |  |
| 263 | NLGN3.51 | [chrX:70165626-70166262](http://genome.ucsc.edu/cgi-bin/hgTracks?db=hg17&position=chrX:70165626-70166262&hgt.customText=http://www.neurogenome.org/mcs/tracks/chrX.txt) | 637 | 2b |  |  |  | ● | ● |  |  |
| 264 | NLGN3.61 | [chrX:70169872-70170515](http://genome.ucsc.edu/cgi-bin/hgTracks?db=hg17&position=chrX:70169872-70170515&hgt.customText=http://www.neurogenome.org/mcs/tracks/chrX.txt) | 644 | 1a | ● | ● | ● | ● | ● | ● | ● |
| 265 | NLGN3.63 | [chrX:70172112-70172662](http://genome.ucsc.edu/cgi-bin/hgTracks?db=hg17&position=chrX:70172112-70172662&hgt.customText=http://www.neurogenome.org/mcs/tracks/chrX.txt) | 551 | 1a | ● | ● |  | ● | ● | ● | ● |
| 266 | NLGN3.66 | [chrX:70172792-70173446](http://genome.ucsc.edu/cgi-bin/hgTracks?db=hg17&position=chrX:70172792-70173446&hgt.customText=http://www.neurogenome.org/mcs/tracks/chrX.txt) | 655 | 1c (3’) |  | ● | ● | ● | ● |  |  |
| 267 | NLGN4X.24 | [chrX:5680851-5681681](http://genome.ucsc.edu/cgi-bin/hgTracks?db=hg17&position=chrX:5680851-5681681&hgt.customText=http://www.neurogenome.org/mcs/tracks/chrX.txt) | 831 | 1a | N/A | ● | ● | ● | ● | ● | ● |
| 268 | NLGN4X.34 | [chrX:5669661-5670033](http://genome.ucsc.edu/cgi-bin/hgTracks?db=hg17&position=chrX:5669661-5670033&hgt.customText=http://www.neurogenome.org/mcs/tracks/chrX.txt) | 373 | 1c (3’) | N/A | ● | ● | ● | ● | ● |  |
| 269 | NLGN4X.35 | [chrX:5667848-5668313](http://genome.ucsc.edu/cgi-bin/hgTracks?db=hg17&position=chrX:5667848-5668313&hgt.customText=http://www.neurogenome.org/mcs/tracks/chrX.txt) | 466 | 1c (3’) | N/A | ● |  | ● | ● | ● | ● |
| 270 | NLGN4X.54 | [chrX:5431152-5431644](http://genome.ucsc.edu/cgi-bin/hgTracks?db=hg17&position=chrX:5431152-5431644&hgt.customText=http://www.neurogenome.org/mcs/tracks/chrX.txt) | 493 | 2a (3’) | N/A |  |  | ● |  |  | ● |
| 271 | NLGN4Y.38 | [chr●:15379728-15380533](http://genome.ucsc.edu/cgi-bin/hgTracks?db=hg17&position=chrY:15379728-15380533&hgt.customText=http://www.neurogenome.org/mcs/tracks/chrY.txt) | 806 | 1c (3’) |  | ● | ● | ● | ● | ● | ● |
| 272 | NRXN1.7 | [chr2:53774921-53775463](http://genome.ucsc.edu/cgi-bin/hgTracks?db=hg17&position=chr2:53774921-53775463&hgt.customText=http://www.neurogenome.org/mcs/tracks/chr2.txt) | 543 | 2a (5’) | N/A |  |  | ● | ● |  |  |
| 273 | NRXN1.14 | [chr2:53743841-53744235](http://genome.ucsc.edu/cgi-bin/hgTracks?db=hg17&position=chr2:53743841-53744235&hgt.customText=http://www.neurogenome.org/mcs/tracks/chr2.txt) | 395 | 2a (5’) | N/A |  |  | ● | ● |  |  |
| 274 | NRXN1.26 | [chr2:53684274-53684933](http://genome.ucsc.edu/cgi-bin/hgTracks?db=hg17&position=chr2:53684274-53684933&hgt.customText=http://www.neurogenome.org/mcs/tracks/chr2.txt) | 660 | 2a (5’) | N/A |  | ● | ● | ● |  |  |
| 275 | NRXN1.37 | [chr2:53658918-53659473](http://genome.ucsc.edu/cgi-bin/hgTracks?db=hg17&position=chr2:53658918-53659473&hgt.customText=http://www.neurogenome.org/mcs/tracks/chr2.txt) | 556 | 2a (5’) | N/A |  |  | ● | ● |  |  |
| 276 | NRXN1.38 | [chr2:53654607-53655090](http://genome.ucsc.edu/cgi-bin/hgTracks?db=hg17&position=chr2:53654607-53655090&hgt.customText=http://www.neurogenome.org/mcs/tracks/chr2.txt) | 484 | 2a (5’) | N/A |  | ● | ● | ● |  |  |
| 277 | NRXN1.44 | [chr2:53624130-53624549](http://genome.ucsc.edu/cgi-bin/hgTracks?db=hg17&position=chr2:53624130-53624549&hgt.customText=http://www.neurogenome.org/mcs/tracks/chr2.txt) | 420 | 2a (5’) | N/A |  | ● | ● | ● |  |  |
| 278 | NRXN1.63 | [chr2:53573100-53573635](http://genome.ucsc.edu/cgi-bin/hgTracks?db=hg17&position=chr2:53573100-53573635&hgt.customText=http://www.neurogenome.org/mcs/tracks/chr2.txt) | 536 | 2a (5’) | N/A |  |  | ● | ● |  |  |
| 279 | NRXN1.85 | [chr2:53348891-53349267](http://genome.ucsc.edu/cgi-bin/hgTracks?db=hg17&position=chr2:53348891-53349267&hgt.customText=http://www.neurogenome.org/mcs/tracks/chr2.txt) | 377 | 2a (5’) | N/A |  |  | ● | ● |  |  |
| 280 | NRXN1.92 | [chr2:53327737-53328209](http://genome.ucsc.edu/cgi-bin/hgTracks?db=hg17&position=chr2:53327737-53328209&hgt.customText=http://www.neurogenome.org/mcs/tracks/chr2.txt) | 473 | 2a (5’) | N/A |  |  | ● | ● |  |  |
| 281 | NRXN1.109 | [chr2:53206242-53206623](http://genome.ucsc.edu/cgi-bin/hgTracks?db=hg17&position=chr2:53206242-53206623&hgt.customText=http://www.neurogenome.org/mcs/tracks/chr2.txt) | 382 | 2a (5’) | N/A |  |  | ● | ● |  |  |
| 282 | NRXN1.113 | [chr2:53175381-53175740](http://genome.ucsc.edu/cgi-bin/hgTracks?db=hg17&position=chr2:53175381-53175740&hgt.customText=http://www.neurogenome.org/mcs/tracks/chr2.txt) | 360 | 2a (5’) | N/A |  |  | ● | ● |  |  |
| 283 | NRXN1.157 | [chr2:52920087-52920878](http://genome.ucsc.edu/cgi-bin/hgTracks?db=hg17&position=chr2:52920087-52920878&hgt.customText=http://www.neurogenome.org/mcs/tracks/chr2.txt) | 792 | 2a (5’) | N/A |  | ● | ● | ● |  |  |
| 284 | NRXN1.406 | [chr2:51478676-51479035](http://genome.ucsc.edu/cgi-bin/hgTracks?db=hg17&position=chr2:51478676-51479035&hgt.customText=http://www.neurogenome.org/mcs/tracks/chr2.txt) | 360 | 2a (5’) | N/A |  |  | ● | ● |  |  |
| 285 | NRXN1.483 | [chr2:51252335-51252699](http://genome.ucsc.edu/cgi-bin/hgTracks?db=hg17&position=chr2:51252335-51252699&hgt.customText=http://www.neurogenome.org/mcs/tracks/chr2.txt) | 365 | 2a (5’) | N/A |  |  | ● | ● |  |  |
| 286 | NRXN1.507 | [chr2:51222734-51223117](http://genome.ucsc.edu/cgi-bin/hgTracks?db=hg17&position=chr2:51222734-51223117&hgt.customText=http://www.neurogenome.org/mcs/tracks/chr2.txt) | 384 | 2a (5’) | N/A |  |  | ● | ● |  |  |
| 287 | NRXN1.528 | [chr2:51170539-51171121](http://genome.ucsc.edu/cgi-bin/hgTracks?db=hg17&position=chr2:51170539-51171121&hgt.customText=http://www.neurogenome.org/mcs/tracks/chr2.txt) | 583 | 2a (5’) | N/A | ● | ● | ● | ● |  |  |
| 288 | NRXN1.685 | [chr2:50933034-50933436](http://genome.ucsc.edu/cgi-bin/hgTracks?db=hg17&position=chr2:50933034-50933436&hgt.customText=http://www.neurogenome.org/mcs/tracks/chr2.txt) | 403 | 2b | N/A |  |  | ● | ● |  |  |
| 289 | NRXN1.750 | [chr2:50845942-50846443](http://genome.ucsc.edu/cgi-bin/hgTracks?db=hg17&position=chr2:50845942-50846443&hgt.customText=http://www.neurogenome.org/mcs/tracks/chr2.txt) | 502 | 2b | N/A |  |  | ● | ● |  |  |
| 290 | NRXN1.801 | [chr2:50762037-50762409](http://genome.ucsc.edu/cgi-bin/hgTracks?db=hg17&position=chr2:50762037-50762409&hgt.customText=http://www.neurogenome.org/mcs/tracks/chr2.txt) | 373 | 1a | N/A | ● |  | ● | ● | ● | ● |
| 291 | NRXN1.812 | [chr2:50759606-50760289](http://genome.ucsc.edu/cgi-bin/hgTracks?db=hg17&position=chr2:50759606-50760289&hgt.customText=http://www.neurogenome.org/mcs/tracks/chr2.txt) | 684 | 1b | N/A |  |  | ● | ● | ● | ● |
| 292 | NRXN1.814 | [chr2:50759086-50759461](http://genome.ucsc.edu/cgi-bin/hgTracks?db=hg17&position=chr2:50759086-50759461&hgt.customText=http://www.neurogenome.org/mcs/tracks/chr2.txt) | 376 | 2b | N/A |  |  | ● | ● | ● | ● |
| 293 | NRXN1.859 | [chr2:50691373-50691826](http://genome.ucsc.edu/cgi-bin/hgTracks?db=hg17&position=chr2:50691373-50691826&hgt.customText=http://www.neurogenome.org/mcs/tracks/chr2.txt) | 454 | 1a | N/A | ● | ● | ● | ● | ● | ● |
| 294 | NRXN1.865 | [chr2:50685698-50686088](http://genome.ucsc.edu/cgi-bin/hgTracks?db=hg17&position=chr2:50685698-50686088&hgt.customText=http://www.neurogenome.org/mcs/tracks/chr2.txt) | 391 | 2b | N/A |  |  | ● | ● | ● |  |
| 295 | NRXN1.871 | [chr2:50677037-50677402](http://genome.ucsc.edu/cgi-bin/hgTracks?db=hg17&position=chr2:50677037-50677402&hgt.customText=http://www.neurogenome.org/mcs/tracks/chr2.txt) | 366 | 1a | N/A | ● |  | ● | ● | ● | ● |
| 296 | NRXN1.916 | [chr2:50636115-50636505](http://genome.ucsc.edu/cgi-bin/hgTracks?db=hg17&position=chr2:50636115-50636505&hgt.customText=http://www.neurogenome.org/mcs/tracks/chr2.txt) | 391 | 1a | N/A | ● |  | ● | ● | ● | ● |
| 297 | NRXN1.1055 | [chr2:50485471-50485914](http://genome.ucsc.edu/cgi-bin/hgTracks?db=hg17&position=chr2:50485471-50485914&hgt.customText=http://www.neurogenome.org/mcs/tracks/chr2.txt) | 444 | 1c (5’) | N/A | ● |  | ● | ● | ● |  |
| 298 | NRXN1.1201 | [chr2:50265322-50265739](http://genome.ucsc.edu/cgi-bin/hgTracks?db=hg17&position=chr2:50265322-50265739&hgt.customText=http://www.neurogenome.org/mcs/tracks/chr2.txt) | 418 | 2b | N/A |  | ● | ● | ● |  |  |
| 299 | NRXN1.1221 | [chr2:50235170-50235602](http://genome.ucsc.edu/cgi-bin/hgTracks?db=hg17&position=chr2:50235170-50235602&hgt.customText=http://www.neurogenome.org/mcs/tracks/chr2.txt) | 433 | 2b | N/A |  |  | ● | ● |  |  |
| 300 | NRXN1.1242 | [chr2:50193547-50194341](http://genome.ucsc.edu/cgi-bin/hgTracks?db=hg17&position=chr2:50193547-50194341&hgt.customText=http://www.neurogenome.org/mcs/tracks/chr2.txt) | 795 | 2b | N/A |  |  | ● | ● | ● | ● |
| 301 | NRXN1.1277 | [chr2:50148323-50148732](http://genome.ucsc.edu/cgi-bin/hgTracks?db=hg17&position=chr2:50148323-50148732&hgt.customText=http://www.neurogenome.org/mcs/tracks/chr2.txt) | 410 | 2b | N/A |  |  | ● | ● |  |  |
| 302 | NRXN1.1291 | [chr2:50133591-50134031](http://genome.ucsc.edu/cgi-bin/hgTracks?db=hg17&position=chr2:50133591-50134031&hgt.customText=http://www.neurogenome.org/mcs/tracks/chr2.txt) | 441 | 2b | N/A |  | ● | ● | ● |  |  |
| 303 | NRXN1.1300 | [chr2:50112721-50113106](http://genome.ucsc.edu/cgi-bin/hgTracks?db=hg17&position=chr2:50112721-50113106&hgt.customText=http://www.neurogenome.org/mcs/tracks/chr2.txt) | 386 | 1c (5’) | N/A | ● |  | ● | ● | ● | ● |
| 304 | NRXN1.1304 | [chr2:50112008-50112464](http://genome.ucsc.edu/cgi-bin/hgTracks?db=hg17&position=chr2:50112008-50112464&hgt.customText=http://www.neurogenome.org/mcs/tracks/chr2.txt) | 457 | 2b | N/A |  |  | ● | ● |  |  |
| 305 | NRXN1.1334 | [chr2:50076508-50076929](http://genome.ucsc.edu/cgi-bin/hgTracks?db=hg17&position=chr2:50076508-50076929&hgt.customText=http://www.neurogenome.org/mcs/tracks/chr2.txt) | 422 | 2b | N/A |  |  | ● | ● |  |  |
| 306 | NRXN1.1404 | [chr2:49929079-49929463](http://genome.ucsc.edu/cgi-bin/hgTracks?db=hg17&position=chr2:49929079-49929463&hgt.customText=http://www.neurogenome.org/mcs/tracks/chr2.txt) | 385 | 2a (3’) | N/A |  |  | ● | ● | ● |  |
| 307 | NRXN1.1406 | [chr2:49920962-49921382](http://genome.ucsc.edu/cgi-bin/hgTracks?db=hg17&position=chr2:49920962-49921382&hgt.customText=http://www.neurogenome.org/mcs/tracks/chr2.txt) | 421 | 2a (3’) | N/A |  |  | ● | ● |  |  |
| 308 | NRXN1.1407 | [chr2:49913436-49913804](http://genome.ucsc.edu/cgi-bin/hgTracks?db=hg17&position=chr2:49913436-49913804&hgt.customText=http://www.neurogenome.org/mcs/tracks/chr2.txt) | 369 | 2a (3’) | N/A |  | ● | ● | ● |  |  |
| 309 | NRXN1.1467 | [chr2:49755824-49756279](http://genome.ucsc.edu/cgi-bin/hgTracks?db=hg17&position=chr2:49755824-49756279&hgt.customText=http://www.neurogenome.org/mcs/tracks/chr2.txt) | 456 | 2a (3’) | N/A |  |  | ● | ● |  |  |
| 310 | NRXN1.1508 | [chr2:49655365-49655796](http://genome.ucsc.edu/cgi-bin/hgTracks?db=hg17&position=chr2:49655365-49655796&hgt.customText=http://www.neurogenome.org/mcs/tracks/chr2.txt) | 432 | 2a (3’) | N/A |  | ● | ● | ● |  |  |
| 311 | NRXN1.1611 | [chr2:49407066-49407444](http://genome.ucsc.edu/cgi-bin/hgTracks?db=hg17&position=chr2:49407066-49407444&hgt.customText=http://www.neurogenome.org/mcs/tracks/chr2.txt) | 379 | 2a (3’) | N/A |  |  | ● | ● |  |  |
| 312 | NRXN1.1632 | [chr2:49376720-49377248](http://genome.ucsc.edu/cgi-bin/hgTracks?db=hg17&position=chr2:49376720-49377248&hgt.customText=http://www.neurogenome.org/mcs/tracks/chr2.txt) | 529 | 2a (3’) | N/A |  | ● | ● | ● | ● |  |
| 313 | NRXN2.78 | [chr11:64216810-64217175](http://genome.ucsc.edu/cgi-bin/hgTracks?db=hg17&position=chr11:64216810-64217175&hgt.customText=http://www.neurogenome.org/mcs/tracks/chr11.txt) | 366 | 1b | N/A |  |  |  | ● |  |  |
| 314 | NRXN2.143 | [chr11:64177684-64178240](http://genome.ucsc.edu/cgi-bin/hgTracks?db=hg17&position=chr11:64177684-64178240&hgt.customText=http://www.neurogenome.org/mcs/tracks/chr11.txt) | 557 | 1b | N/A |  | ● | ● | ● |  |  |
| 315 | NRXN2.173 | [chr11:64166580-64167083](http://genome.ucsc.edu/cgi-bin/hgTracks?db=hg17&position=chr11:64166580-64167083&hgt.customText=http://www.neurogenome.org/mcs/tracks/chr11.txt) | 504 | 1c (5’) | N/A | ● | ● |  | ● |  |  |
| 316 | NRXN2.188 | [chr11:64160183-64160679](http://genome.ucsc.edu/cgi-bin/hgTracks?db=hg17&position=chr11:64160183-64160679&hgt.customText=http://www.neurogenome.org/mcs/tracks/chr11.txt) | 497 | 2b | N/A |  | ● | ● | ● |  |  |
| 317 | NRXN2.198 | [chr11:64156672-64157066](http://genome.ucsc.edu/cgi-bin/hgTracks?db=hg17&position=chr11:64156672-64157066&hgt.customText=http://www.neurogenome.org/mcs/tracks/chr11.txt) | 395 | 2b | N/A |  | ● | ● | ● |  |  |
| 318 | NRXN2.215 | [chr11:64149258-64149774](http://genome.ucsc.edu/cgi-bin/hgTracks?db=hg17&position=chr11:64149258-64149774&hgt.customText=http://www.neurogenome.org/mcs/tracks/chr11.txt) | 517 | 2b | N/A |  | ● | ● | ● |  |  |
| 319 | NRXN2.245 | [chr11:64132143-64132519](http://genome.ucsc.edu/cgi-bin/hgTracks?db=hg17&position=chr11:64132143-64132519&hgt.customText=http://www.neurogenome.org/mcs/tracks/chr11.txt) | 377 | 2b | N/A | ● |  | ● | ● |  |  |
| 320 | NRXN2.251 | [chr11:64131106-64131595](http://genome.ucsc.edu/cgi-bin/hgTracks?db=hg17&position=chr11:64131106-64131595&hgt.customText=http://www.neurogenome.org/mcs/tracks/chr11.txt) | 490 | 1c (3’) | N/A | ● |  | ● | ● | ● | ● |
| 321 | NRXN3.32 | [chr14:77526043-77526418](http://genome.ucsc.edu/cgi-bin/hgTracks?db=hg17&position=chr14:77526043-77526418&hgt.customText=http://www.neurogenome.org/mcs/tracks/chr14.txt) | 376 | 2a (5’) |  |  |  | ● | ● |  |  |
| 322 | NRXN3.109 | [chr14:77681877-77682369](http://genome.ucsc.edu/cgi-bin/hgTracks?db=hg17&position=chr14:77681877-77682369&hgt.customText=http://www.neurogenome.org/mcs/tracks/chr14.txt) | 493 | 2a (5’) |  |  |  | ● | ● |  |  |
| 323 | NRXN3.139 | [chr14:77706310-77706802](http://genome.ucsc.edu/cgi-bin/hgTracks?db=hg17&position=chr14:77706310-77706802&hgt.customText=http://www.neurogenome.org/mcs/tracks/chr14.txt) | 493 | 1c (5’) | ● |  | ● | ● | ● |  |  |
| 324 | NRXN3.153 | [chr14:77708213-77708726](http://genome.ucsc.edu/cgi-bin/hgTracks?db=hg17&position=chr14:77708213-77708726&hgt.customText=http://www.neurogenome.org/mcs/tracks/chr14.txt) | 514 | 2b | ● |  |  | ● | ● |  |  |
| 325 | NRXN3.156 | [chr14:77709012-77709602](http://genome.ucsc.edu/cgi-bin/hgTracks?db=hg17&position=chr14:77709012-77709602&hgt.customText=http://www.neurogenome.org/mcs/tracks/chr14.txt) | 591 | 2b | ● |  |  | ● | ● | ● |  |
| 326 | NRXN3.231 | [chr14:77779211-77779578](http://genome.ucsc.edu/cgi-bin/hgTracks?db=hg17&position=chr14:77779211-77779578&hgt.customText=http://www.neurogenome.org/mcs/tracks/chr14.txt) | 368 | 1a |  | ● |  | ● | ● | ● | ● |
| 327 | NRXN3.322 | [chr14:77834436-77834833](http://genome.ucsc.edu/cgi-bin/hgTracks?db=hg17&position=chr14:77834436-77834833&hgt.customText=http://www.neurogenome.org/mcs/tracks/chr14.txt) | 398 | 2b |  |  |  | ● | ● | ● |  |
| 328 | NRXN3.399 | [chr14:77881257-77881625](http://genome.ucsc.edu/cgi-bin/hgTracks?db=hg17&position=chr14:77881257-77881625&hgt.customText=http://www.neurogenome.org/mcs/tracks/chr14.txt) | 369 | 2b |  |  |  | ● | ● | ● |  |
| 329 | NRXN3.443 | [chr14:77939369-77940141](http://genome.ucsc.edu/cgi-bin/hgTracks?db=hg17&position=chr14:77939369-77940141&hgt.customText=http://www.neurogenome.org/mcs/tracks/chr14.txt) | 773 | 1c (5’) |  |  |  | ● | ● |  |  |
| 330 | NRXN3.475 | [chr14:77976442-77976838](http://genome.ucsc.edu/cgi-bin/hgTracks?db=hg17&position=chr14:77976442-77976838&hgt.customText=http://www.neurogenome.org/mcs/tracks/chr14.txt) | 397 | 2b |  |  |  | ● | ● |  |  |
| 331 | NRXN3.513 | [chr14:78013494-78013927](http://genome.ucsc.edu/cgi-bin/hgTracks?db=hg17&position=chr14:78013494-78013927&hgt.customText=http://www.neurogenome.org/mcs/tracks/chr14.txt) | 434 | 2b |  |  |  | ● | ● |  |  |
| 332 | NRXN3.728 | [chr14:78245279-78245753](http://genome.ucsc.edu/cgi-bin/hgTracks?db=hg17&position=chr14:78245279-78245753&hgt.customText=http://www.neurogenome.org/mcs/tracks/chr14.txt) | 475 | 1a |  | ● |  | ● | ● | ● | ● |
| 333 | NRXN3.733 | [chr14:78250849-78251217](http://genome.ucsc.edu/cgi-bin/hgTracks?db=hg17&position=chr14:78250849-78251217&hgt.customText=http://www.neurogenome.org/mcs/tracks/chr14.txt) | 369 | 1a | ● | ● |  | ● | ● | ● | ● |
| 334 | NRXN3.736 | [chr14:78253285-78253663](http://genome.ucsc.edu/cgi-bin/hgTracks?db=hg17&position=chr14:78253285-78253663&hgt.customText=http://www.neurogenome.org/mcs/tracks/chr14.txt) | 379 | 2b |  |  |  | ● | ● | ● |  |
| 335 | NRXN3.891 | [chr14:78402348-78402747](http://genome.ucsc.edu/cgi-bin/hgTracks?db=hg17&position=chr14:78402348-78402747&hgt.customText=http://www.neurogenome.org/mcs/tracks/chr14.txt) | 400 | 2b |  |  |  | ● | ● |  |  |
| 336 | NRXN3.945 | [chr14:78502119-78502510](http://genome.ucsc.edu/cgi-bin/hgTracks?db=hg17&position=chr14:78502119-78502510&hgt.customText=http://www.neurogenome.org/mcs/tracks/chr14.txt) | 392 | 1a |  | ● |  | ● | ● | ● | ● |
| 337 | NRXN3.966 | [chr14:78521779-78522175](http://genome.ucsc.edu/cgi-bin/hgTracks?db=hg17&position=chr14:78521779-78522175&hgt.customText=http://www.neurogenome.org/mcs/tracks/chr14.txt) | 397 | 2b |  |  |  | ● | ● |  |  |
| 338 | NRXN3.994 | [chr14:78563287-78563682](http://genome.ucsc.edu/cgi-bin/hgTracks?db=hg17&position=chr14:78563287-78563682&hgt.customText=http://www.neurogenome.org/mcs/tracks/chr14.txt) | 396 | 2b |  |  |  | ● | ● |  |  |
| 339 | NRXN3.1112 | [chr14:78673210-78673685](http://genome.ucsc.edu/cgi-bin/hgTracks?db=hg17&position=chr14:78673210-78673685&hgt.customText=http://www.neurogenome.org/mcs/tracks/chr14.txt) | 476 | 2b |  |  | ● | ● | ● |  |  |
| 340 | NRXN3.1149 | [chr14:78713203-78713628](http://genome.ucsc.edu/cgi-bin/hgTracks?db=hg17&position=chr14:78713203-78713628&hgt.customText=http://www.neurogenome.org/mcs/tracks/chr14.txt) | 426 | 2b |  |  |  | ● | ● | ● |  |
| 341 | NRXN3.1173 | [chr14:78734225-78734602](http://genome.ucsc.edu/cgi-bin/hgTracks?db=hg17&position=chr14:78734225-78734602&hgt.customText=http://www.neurogenome.org/mcs/tracks/chr14.txt) | 378 | 2b |  |  |  | ● | ● |  |  |
| 342 | NRXN3.1207 | [chr14:78780479-78780878](http://genome.ucsc.edu/cgi-bin/hgTracks?db=hg17&position=chr14:78780479-78780878&hgt.customText=http://www.neurogenome.org/mcs/tracks/chr14.txt) | 400 | 2b |  |  |  | ● | ● |  |  |
| 343 | NRXN3.1241 | [chr14:78816295-78816703](http://genome.ucsc.edu/cgi-bin/hgTracks?db=hg17&position=chr14:78816295-78816703&hgt.customText=http://www.neurogenome.org/mcs/tracks/chr14.txt) | 409 | 1c (5’) | ● | ● |  | ● | ● | ● | ● |
| 344 | NRXN3.1263 | [chr14:78837350-78837778](http://genome.ucsc.edu/cgi-bin/hgTracks?db=hg17&position=chr14:78837350-78837778&hgt.customText=http://www.neurogenome.org/mcs/tracks/chr14.txt) | 429 | 2b |  |  |  | ● | ● |  |  |
| 345 | NRXN3.1282 | [chr14:78862098-78862461](http://genome.ucsc.edu/cgi-bin/hgTracks?db=hg17&position=chr14:78862098-78862461&hgt.customText=http://www.neurogenome.org/mcs/tracks/chr14.txt) | 364 | 2b |  |  |  | ● | ● |  |  |
| 346 | NRXN3.1306 | [chr14:78887900-78888333](http://genome.ucsc.edu/cgi-bin/hgTracks?db=hg17&position=chr14:78887900-78888333&hgt.customText=http://www.neurogenome.org/mcs/tracks/chr14.txt) | 434 | 2b |  |  |  | ● | ● |  |  |
| 347 | NRXN3.1337 | [chr14:78909597-78909985](http://genome.ucsc.edu/cgi-bin/hgTracks?db=hg17&position=chr14:78909597-78909985&hgt.customText=http://www.neurogenome.org/mcs/tracks/chr14.txt) | 389 | 2b |  |  |  | ● | ● | ● |  |
| 348 | NRXN3.1369 | [chr14:78942861-78943254](http://genome.ucsc.edu/cgi-bin/hgTracks?db=hg17&position=chr14:78942861-78943254&hgt.customText=http://www.neurogenome.org/mcs/tracks/chr14.txt) | 394 | 2b |  |  | ● | ● | ● |  |  |
| 349 | NRXN3.1458 | [chr14:79049445-79049889](http://genome.ucsc.edu/cgi-bin/hgTracks?db=hg17&position=chr14:79049445-79049889&hgt.customText=http://www.neurogenome.org/mcs/tracks/chr14.txt) | 445 | 2b |  |  | ● | ● | ● |  |  |
| 350 | NRXN3.1812 | [chr14:79397101-79398063](http://genome.ucsc.edu/cgi-bin/hgTracks?db=hg17&position=chr14:79397101-79398063&hgt.customText=http://www.neurogenome.org/mcs/tracks/chr14.txt) | 963 | 1c (3’) | ● | ● | ● | ● | ● | ● | ● |
| 351 | NRXN3.1825 | [chr14:79399260-79399673](http://genome.ucsc.edu/cgi-bin/hgTracks?db=hg17&position=chr14:79399260-79399673&hgt.customText=http://www.neurogenome.org/mcs/tracks/chr14.txt) | 414 | 1c (3’) |  | ● | ● | ● | ● | ● |  |
| 352 | NRXN3.1901 | [chr14:79551953-79552315](http://genome.ucsc.edu/cgi-bin/hgTracks?db=hg17&position=chr14:79551953-79552315&hgt.customText=http://www.neurogenome.org/mcs/tracks/chr14.txt) | 363 | 2a (3’) |  |  |  | ● | ● | ● |  |
| 353 | NRXN3.1925 | [chr14:79636808-79637195](http://genome.ucsc.edu/cgi-bin/hgTracks?db=hg17&position=chr14:79636808-79637195&hgt.customText=http://www.neurogenome.org/mcs/tracks/chr14.txt) | 388 | 2a (3’) |  |  |  | ● | ● |  |  |
| 354 | NRXN3.1968 | [chr14:79703988-79704357](http://genome.ucsc.edu/cgi-bin/hgTracks?db=hg17&position=chr14:79703988-79704357&hgt.customText=http://www.neurogenome.org/mcs/tracks/chr14.txt) | 370 | 2a (3’) |  |  |  | ● | ● |  |  |
| 355 | NSF.205 | [chr17:42152967-42153393](http://genome.ucsc.edu/cgi-bin/hgTracks?db=hg17&position=chr17:42152967-42153393&hgt.customText=http://www.neurogenome.org/mcs/tracks/chr17.txt) | 427 | 2b | N/A |  |  | ● | ● |  |  |
| 356 | NSF.209 | [chr17:42154208-42154722](http://genome.ucsc.edu/cgi-bin/hgTracks?db=hg17&position=chr17:42154208-42154722&hgt.customText=http://www.neurogenome.org/mcs/tracks/chr17.txt) | 515 | 2b | N/A | ● |  | ● | ● | ● | ● |
| 357 | NSF.222 | [chr17:42164750-42165764](http://genome.ucsc.edu/cgi-bin/hgTracks?db=hg17&position=chr17:42164750-42165764&hgt.customText=http://www.neurogenome.org/mcs/tracks/chr17.txt) | 1015 | 2b | N/A |  | ● | ● | ● | ● |  |
| 358 | NSF.230 | [chr17:42176946-42177479](http://genome.ucsc.edu/cgi-bin/hgTracks?db=hg17&position=chr17:42176946-42177479&hgt.customText=http://www.neurogenome.org/mcs/tracks/chr17.txt) | 534 | 2b | N/A |  |  | ● | ● |  |  |
| 359 | NSF.242 | [chr17:42186467-42186851](http://genome.ucsc.edu/cgi-bin/hgTracks?db=hg17&position=chr17:42186467-42186851&hgt.customText=http://www.neurogenome.org/mcs/tracks/chr17.txt) | 385 | 2b | N/A |  |  | ● | ● | ● |  |
| 360 | PCLO.43 | [chr7:82435934-82436604](http://genome.ucsc.edu/cgi-bin/hgTracks?db=hg17&position=chr7:82435934-82436604&hgt.customText=http://www.neurogenome.org/mcs/tracks/chr7.txt) | 671 | 1b |  |  | ● | ● | ● |  |  |
| 361 | PCLO.119 | [chr7:82229222-82229849](http://genome.ucsc.edu/cgi-bin/hgTracks?db=hg17&position=chr7:82229222-82229849&hgt.customText=http://www.neurogenome.org/mcs/tracks/chr7.txt) | 628 | 1a | ● | ● | ● | ● | ● | ● | ● |
| 362 | PCLO.155 | [chr7:82224584-82224956](http://genome.ucsc.edu/cgi-bin/hgTracks?db=hg17&position=chr7:82224584-82224956&hgt.customText=http://www.neurogenome.org/mcs/tracks/chr7.txt) | 373 | 1a | ● | ● |  | ● | ● | ● | ● |
| 363 | PCLO.162 | [chr7:82223371-82224024](http://genome.ucsc.edu/cgi-bin/hgTracks?db=hg17&position=chr7:82223371-82224024&hgt.customText=http://www.neurogenome.org/mcs/tracks/chr7.txt) | 654 | 1a | ● | ● |  | ● | ● | ● | ● |
| 364 | PCLO.175 | [chr7:82189972-82190518](http://genome.ucsc.edu/cgi-bin/hgTracks?db=hg17&position=chr7:82189972-82190518&hgt.customText=http://www.neurogenome.org/mcs/tracks/chr7.txt) | 547 | 1a | ● | ● |  | ● | ● | ● | ● |
| 365 | PCLO.215 | [chr7:82094727-82095436](http://genome.ucsc.edu/cgi-bin/hgTracks?db=hg17&position=chr7:82094727-82095436&hgt.customText=http://www.neurogenome.org/mcs/tracks/chr7.txt) | 710 | 2a (3’) | ● | ● |  | ● | ● | ● |  |
| 366 | PCLO.259 | [chr7:81991063-81991438](http://genome.ucsc.edu/cgi-bin/hgTracks?db=hg17&position=chr7:81991063-81991438&hgt.customText=http://www.neurogenome.org/mcs/tracks/chr7.txt) | 376 | 2a (3’) |  |  |  | ● | ● |  |  |
| 367 | PCLO.265 | [chr7:81966197-81966630](http://genome.ucsc.edu/cgi-bin/hgTracks?db=hg17&position=chr7:81966197-81966630&hgt.customText=http://www.neurogenome.org/mcs/tracks/chr7.txt) | 434 | 2a (3’) |  |  |  |  | ● |  |  |
| 368 | RAB3C.131 | [chr5:58154156-58154824](http://genome.ucsc.edu/cgi-bin/hgTracks?db=hg17&position=chr5:58154156-58154824&hgt.customText=http://www.neurogenome.org/mcs/tracks/chr5.txt) | 669 | 2b | N/A |  | ● | ● | ● | ● | ● |
| 369 | RAB3C.156 | [chr5:58188476-58188873](http://genome.ucsc.edu/cgi-bin/hgTracks?db=hg17&position=chr5:58188476-58188873&hgt.customText=http://www.neurogenome.org/mcs/tracks/chr5.txt) | 398 | 2a (3’) | N/A | ● |  | ● | ● | ● |  |
| 370 | RAB5A.17 | [chr3:20000979-20001413](http://genome.ucsc.edu/cgi-bin/hgTracks?db=hg17&position=chr3:20000979-20001413&hgt.customText=http://www.neurogenome.org/mcs/tracks/chr3.txt) | 435 | 1c (3’) | N/A | ● |  | ● | ● | ● |  |
| 371 | RAB6IP2.20 | [chr12:1007322-1008005](http://genome.ucsc.edu/cgi-bin/hgTracks?db=hg17&position=chr12:1007322-1008005&hgt.customText=http://www.neurogenome.org/mcs/tracks/chr12.txt) | 684 | 1a | N/A | ● | ● | ● | ● | ● | ● |
| 372 | RAB6IP2.59 | [chr12:1131993-1132352](http://genome.ucsc.edu/cgi-bin/hgTracks?db=hg17&position=chr12:1131993-1132352&hgt.customText=http://www.neurogenome.org/mcs/tracks/chr12.txt) | 360 | 2b | N/A |  |  | ● | ● | ● | ● |
| 373 | RAB6IP2.93 | [chr12:1257370-1257749](http://genome.ucsc.edu/cgi-bin/hgTracks?db=hg17&position=chr12:1257370-1257749&hgt.customText=http://www.neurogenome.org/mcs/tracks/chr12.txt) | 380 | 2b | N/A |  |  | ● | ● |  |  |
| 374 | RABAC1.27 | [chr19:47129083-47129542](http://genome.ucsc.edu/cgi-bin/hgTracks?db=hg17&position=chr19:47129083-47129542&hgt.customText=http://www.neurogenome.org/mcs/tracks/chr19.txt) | 460 | 2a (3’) |  |  |  |  | ● |  | ● |
| 375 | RABAC1.37 | [chr19:47122529-47122910](http://genome.ucsc.edu/cgi-bin/hgTracks?db=hg17&position=chr19:47122529-47122910&hgt.customText=http://www.neurogenome.org/mcs/tracks/chr19.txt) | 382 | 2a (3’) |  |  | ● |  | ● |  |  |
| 376 | RABAC1.65 | [chr19:47104278-47104671](http://genome.ucsc.edu/cgi-bin/hgTracks?db=hg17&position=chr19:47104278-47104671&hgt.customText=http://www.neurogenome.org/mcs/tracks/chr19.txt) | 394 | 2a (3’) |  | ● |  | ● | ● |  | ● |
| 377 | RIMBP2.55 | [chr12:129451268-129451644](http://genome.ucsc.edu/cgi-bin/hgTracks?db=hg17&position=chr12:129451268-129451644&hgt.customText=http://www.neurogenome.org/mcs/tracks/chr12.txt) | 377 | 1a | N/A | ● | ● |  | ● | ● | ● |
| 378 | RIMS1.37 | [chr6:72245292-72245942](http://genome.ucsc.edu/cgi-bin/hgTracks?db=hg17&position=chr6:72245292-72245942&hgt.customText=http://www.neurogenome.org/mcs/tracks/chr6.txt) | 651 | 2a (5’) |  |  | ● | ● | ● |  |  |
| 379 | RIMS1.208 | [chr6:72653011-72653806](http://genome.ucsc.edu/cgi-bin/hgTracks?db=hg17&position=chr6:72653011-72653806&hgt.customText=http://www.neurogenome.org/mcs/tracks/chr6.txt) | 796 | 1c (5’) |  | ● |  | ● | ● | ● |  |
| 380 | RIMS1.324 | [chr6:72839613-72840086](http://genome.ucsc.edu/cgi-bin/hgTracks?db=hg17&position=chr6:72839613-72840086&hgt.customText=http://www.neurogenome.org/mcs/tracks/chr6.txt) | 474 | 2b |  |  |  | ● | ● |  |  |
| 381 | RIMS1.437 | [chr6:73057063-73057461](http://genome.ucsc.edu/cgi-bin/hgTracks?db=hg17&position=chr6:73057063-73057461&hgt.customText=http://www.neurogenome.org/mcs/tracks/chr6.txt) | 399 | 1b | ● | ● | ● | ● | ● | ● |  |
| 382 | RIMS1.502 | [chr6:73168775-73169254](http://genome.ucsc.edu/cgi-bin/hgTracks?db=hg17&position=chr6:73168775-73169254&hgt.customText=http://www.neurogenome.org/mcs/tracks/chr6.txt) | 480 | 1c (3’) |  | ● |  | ● | ● | ● |  |
| 383 | RIMS2.9 | [chr8:104531098-104531467](http://genome.ucsc.edu/cgi-bin/hgTracks?db=hg17&position=chr8:104531098-104531467&hgt.customText=http://www.neurogenome.org/mcs/tracks/chr8.txt) | 370 | 2a (5’) | N/A |  |  | ● | ● |  |  |
| 384 | RIMS2.14 | [chr8:104534098-104534480](http://genome.ucsc.edu/cgi-bin/hgTracks?db=hg17&position=chr8:104534098-104534480&hgt.customText=http://www.neurogenome.org/mcs/tracks/chr8.txt) | 383 | 2a (5’) | N/A |  |  | ● | ● |  |  |
| 385 | RIMS2.48 | [chr8:104581879-104582262](http://genome.ucsc.edu/cgi-bin/hgTracks?db=hg17&position=chr8:104581879-104582262&hgt.customText=http://www.neurogenome.org/mcs/tracks/chr8.txt) | 384 | 1c (5’) | N/A |  |  | ● | ● |  |  |
| 386 | RIMS2.49 | [chr8:104582288-104582698](http://genome.ucsc.edu/cgi-bin/hgTracks?db=hg17&position=chr8:104582288-104582698&hgt.customText=http://www.neurogenome.org/mcs/tracks/chr8.txt) | 411 | 1b | N/A | ● |  | ● | ● | ● |  |
| 387 | RIMS2.81 | [chr8:104649950-104650354](http://genome.ucsc.edu/cgi-bin/hgTracks?db=hg17&position=chr8:104649950-104650354&hgt.customText=http://www.neurogenome.org/mcs/tracks/chr8.txt) | 405 | 2b | N/A |  |  | ● | ● |  | ● |
| 388 | RIMS2.90 | [chr8:104740591-104740988](http://genome.ucsc.edu/cgi-bin/hgTracks?db=hg17&position=chr8:104740591-104740988&hgt.customText=http://www.neurogenome.org/mcs/tracks/chr8.txt) | 398 | 2b | N/A |  | ● | ● | ● |  | ● |
| 389 | RIMS2.118 | [chr8:104849763-104850245](http://genome.ucsc.edu/cgi-bin/hgTracks?db=hg17&position=chr8:104849763-104850245&hgt.customText=http://www.neurogenome.org/mcs/tracks/chr8.txt) | 483 | 2b | N/A |  |  | ● | ● | ● | ● |
| 390 | RIMS2.173 | [chr8:104966883-104967310](http://genome.ucsc.edu/cgi-bin/hgTracks?db=hg17&position=chr8:104966883-104967310&hgt.customText=http://www.neurogenome.org/mcs/tracks/chr8.txt) | 428 | 1a | N/A | ● |  | ● | ● | ● | ● |
| 391 | RIMS2.193 | [chr8:105009080-105009489](http://genome.ucsc.edu/cgi-bin/hgTracks?db=hg17&position=chr8:105009080-105009489&hgt.customText=http://www.neurogenome.org/mcs/tracks/chr8.txt) | 410 | 1b | N/A |  |  |  | ● | ● | ● |
| 392 | RIMS2.214 | [chr8:105094768-105095322](http://genome.ucsc.edu/cgi-bin/hgTracks?db=hg17&position=chr8:105094768-105095322&hgt.customText=http://www.neurogenome.org/mcs/tracks/chr8.txt) | 555 | 1b | N/A |  | ● | ● | ● |  |  |
| 393 | RIMS2.241 | [chr8:105174563-105175052](http://genome.ucsc.edu/cgi-bin/hgTracks?db=hg17&position=chr8:105174563-105175052&hgt.customText=http://www.neurogenome.org/mcs/tracks/chr8.txt) | 490 | 2b | N/A |  |  | ● | ● |  | ● |
| 394 | RIMS2.306 | [chr8:105335384-105335831](http://genome.ucsc.edu/cgi-bin/hgTracks?db=hg17&position=chr8:105335384-105335831&hgt.customText=http://www.neurogenome.org/mcs/tracks/chr8.txt) | 448 | 2a (3’) | N/A | ● |  | ● | ● | ● |  |
| 395 | RIMS3.114 | [chr1:40755409-40755781](http://genome.ucsc.edu/cgi-bin/hgTracks?db=hg17&position=chr1:40755409-40755781&hgt.customText=http://www.neurogenome.org/mcs/tracks/chr1.txt) | 373 | 1c (3’) | N/A | ● |  | ● | ● |  |  |
| 396 | RIMS4.52 | [chr20:42865513-42865927](http://genome.ucsc.edu/cgi-bin/hgTracks?db=hg17&position=chr20:42865513-42865927&hgt.customText=http://www.neurogenome.org/mcs/tracks/chr20.txt) | 415 | 2b |  |  | ● | ● | ● |  |  |
| 397 | RPH3A.71 | [chr12:111692007-111692369](http://genome.ucsc.edu/cgi-bin/hgTracks?db=hg17&position=chr12:111692007-111692369&hgt.customText=http://www.neurogenome.org/mcs/tracks/chr12.txt) | 363 | 2a (5’) | N/A | ● |  | ● | ● |  |  |
| 398 | SCAMP5.8 | [chr15:73055565-73055949](http://genome.ucsc.edu/cgi-bin/hgTracks?db=hg17&position=chr15:73055565-73055949&hgt.customText=http://www.neurogenome.org/mcs/tracks/chr15.txt) | 385 | 2a (5’) | N/A |  | ● | ● | ● | ● | ● |
| 399 | SLC30A3.13 | [chr2:27397222-27397664](http://genome.ucsc.edu/cgi-bin/hgTracks?db=hg17&position=chr2:27397222-27397664&hgt.customText=http://www.neurogenome.org/mcs/tracks/chr2.txt) | 443 | 1c (5’) | N/A | ● |  | ● | ● |  |  |
| 400 | SNAP25.145 | [chr20:10206910-10207329](http://genome.ucsc.edu/cgi-bin/hgTracks?db=hg17&position=chr20:10206910-10207329&hgt.customText=http://www.neurogenome.org/mcs/tracks/chr20.txt) | 420 | 2b |  |  |  | ● | ● |  |  |
| 401 | SNAP25.159 | [chr20:10221453-10222192](http://genome.ucsc.edu/cgi-bin/hgTracks?db=hg17&position=chr20:10221453-10222192&hgt.customText=http://www.neurogenome.org/mcs/tracks/chr20.txt) | 740 | 1b | ● | ● | ● | ● | ● | ● | ● |
| 402 | SNAP25.168 | [chr20:10224631-10225035](http://genome.ucsc.edu/cgi-bin/hgTracks?db=hg17&position=chr20:10224631-10225035&hgt.customText=http://www.neurogenome.org/mcs/tracks/chr20.txt) | 405 | 2b | ● | ● |  | ● | ● | ● |  |
| 403 | SNAP25.229 | [chr20:10314790-10315410](http://genome.ucsc.edu/cgi-bin/hgTracks?db=hg17&position=chr20:10314790-10315410&hgt.customText=http://www.neurogenome.org/mcs/tracks/chr20.txt) | 621 | 2a (3’) | ● | ● |  | ● | ● | ● | ● |
| 404 | SNCA.51 | [chr4:91004386-91004996](http://genome.ucsc.edu/cgi-bin/hgTracks?db=hg17&position=chr4:91004386-91004996&hgt.customText=http://www.neurogenome.org/mcs/tracks/chr4.txt) | 611 | 1c (3’) | N/A | ● | ● | ● | ● | ● |  |
| 405 | SNCA.90 | [chr4:90851177-90851553](http://genome.ucsc.edu/cgi-bin/hgTracks?db=hg17&position=chr4:90851177-90851553&hgt.customText=http://www.neurogenome.org/mcs/tracks/chr4.txt) | 377 | 2a (3’) | N/A |  | ● | ● | ● |  |  |
| 406 | STX16.33 | [chr20:56665713-56666207](http://genome.ucsc.edu/cgi-bin/hgTracks?db=hg17&position=chr20:56665713-56666207&hgt.customText=http://www.neurogenome.org/mcs/tracks/chr20.txt) | 495 | 2b | ● |  |  | ● | ● |  |  |
| 407 | STX16.57 | [chr20:56685285-56685843](http://genome.ucsc.edu/cgi-bin/hgTracks?db=hg17&position=chr20:56685285-56685843&hgt.customText=http://www.neurogenome.org/mcs/tracks/chr20.txt) | 559 | 1c (3’) | ● | ● |  | ● | ● |  |  |
| 408 | STX16.58 | [chr20:56687566-56688037](http://genome.ucsc.edu/cgi-bin/hgTracks?db=hg17&position=chr20:56687566-56688037&hgt.customText=http://www.neurogenome.org/mcs/tracks/chr20.txt) | 472 | 1c (3’) | ● | ● |  | ● | ● | ● |  |
| 409 | STX17.38 | [chr9:99716301-99717127](http://genome.ucsc.edu/cgi-bin/hgTracks?db=hg17&position=chr9:99716301-99717127&hgt.customText=http://www.neurogenome.org/mcs/tracks/chr9.txt) | 827 | 2a (5’) | N/A |  |  | ● | ● |  | ● |
| 410 | STX17.48 | [chr9:99720389-99720771](http://genome.ucsc.edu/cgi-bin/hgTracks?db=hg17&position=chr9:99720389-99720771&hgt.customText=http://www.neurogenome.org/mcs/tracks/chr9.txt) | 383 | 2a (5’) | N/A |  | ● | ● | ● |  | ● |
| 411 | STX17.301 | [chr9:99809272-99809777](http://genome.ucsc.edu/cgi-bin/hgTracks?db=hg17&position=chr9:99809272-99809777&hgt.customText=http://www.neurogenome.org/mcs/tracks/chr9.txt) | 506 | 1b | N/A | ● |  | ● | ● | ● | ● |
| 412 | STX18.30 | [chr4:4937999-4938507](http://genome.ucsc.edu/cgi-bin/hgTracks?db=hg17&position=chr4:4937999-4938507&hgt.customText=http://www.neurogenome.org/mcs/tracks/chr4.txt) | 509 | 2a (5’) | N/A |  |  | ● | ● | ● | ● |
| 413 | STX18.32 | [chr4:4930179-4930591](http://genome.ucsc.edu/cgi-bin/hgTracks?db=hg17&position=chr4:4930179-4930591&hgt.customText=http://www.neurogenome.org/mcs/tracks/chr4.txt) | 413 | 2a (5’) | N/A |  | ● | ● | ● | ● |  |
| 414 | STX18.54 | [chr4:4883191-4883568](http://genome.ucsc.edu/cgi-bin/hgTracks?db=hg17&position=chr4:4883191-4883568&hgt.customText=http://www.neurogenome.org/mcs/tracks/chr4.txt) | 378 | 2a (5’) | N/A |  |  | ● | ● | ● |  |
| 415 | STX18.69 | [chr4:4874537-4874923](http://genome.ucsc.edu/cgi-bin/hgTracks?db=hg17&position=chr4:4874537-4874923&hgt.customText=http://www.neurogenome.org/mcs/tracks/chr4.txt) | 387 | 2a (5’) | N/A |  | ● | ● | ● |  |  |
| 416 | STX18.138 | [chr4:4727129-4727572](http://genome.ucsc.edu/cgi-bin/hgTracks?db=hg17&position=chr4:4727129-4727572&hgt.customText=http://www.neurogenome.org/mcs/tracks/chr4.txt) | 444 | 2a (5’) | N/A |  |  | ● | ● |  |  |
| 417 | STX18.183 | [chr4:4646729-4647111](http://genome.ucsc.edu/cgi-bin/hgTracks?db=hg17&position=chr4:4646729-4647111&hgt.customText=http://www.neurogenome.org/mcs/tracks/chr4.txt) | 383 | 2b | N/A |  |  | ● | ● |  |  |
| 418 | STX18.190 | [chr4:4641320-4642053](http://genome.ucsc.edu/cgi-bin/hgTracks?db=hg17&position=chr4:4641320-4642053&hgt.customText=http://www.neurogenome.org/mcs/tracks/chr4.txt) | 734 | 2b | N/A |  |  | ● | ● | ● | ● |
| 419 | STX18.204 | [chr4:4635528-4636254](http://genome.ucsc.edu/cgi-bin/hgTracks?db=hg17&position=chr4:4635528-4636254&hgt.customText=http://www.neurogenome.org/mcs/tracks/chr4.txt) | 727 | 2b | N/A |  |  | ● | ● | ● |  |
| 420 | STX18.215 | [chr4:4618876-4619293](http://genome.ucsc.edu/cgi-bin/hgTracks?db=hg17&position=chr4:4618876-4619293&hgt.customText=http://www.neurogenome.org/mcs/tracks/chr4.txt) | 418 | 2b | N/A |  |  | ● | ● |  |  |
| 421 | STX18.220 | [chr4:4611690-4612102](http://genome.ucsc.edu/cgi-bin/hgTracks?db=hg17&position=chr4:4611690-4612102&hgt.customText=http://www.neurogenome.org/mcs/tracks/chr4.txt) | 413 | 2b | N/A |  | ● | ● | ● | ● |  |
| 422 | STX1B2.7 | [chr16:30929069-30929602](http://genome.ucsc.edu/cgi-bin/hgTracks?db=hg17&position=chr16:30929069-30929602&hgt.customText=http://www.neurogenome.org/mcs/tracks/chr16.txt) | 534 | 1c (5’) | N/A |  |  | ● | ● |  |  |
| 423 | STX1B2.39 | [chr16:30908012-30908635](http://genome.ucsc.edu/cgi-bin/hgTracks?db=hg17&position=chr16:30908012-30908635&hgt.customText=http://www.neurogenome.org/mcs/tracks/chr16.txt) | 624 | 1c (3’) | N/A | ● | ● | ● | ● |  |  |
| 424 | STX6.30 | [chr1:177701820-177702180](http://genome.ucsc.edu/cgi-bin/hgTracks?db=hg17&position=chr1:177701820-177702180&hgt.customText=http://www.neurogenome.org/mcs/tracks/chr1.txt) | 361 | 2b | N/A |  |  | ● | ● |  |  |
| 425 | STX6.39 | [chr1:177689002-177689405](http://genome.ucsc.edu/cgi-bin/hgTracks?db=hg17&position=chr1:177689002-177689405&hgt.customText=http://www.neurogenome.org/mcs/tracks/chr1.txt) | 404 | 1b | N/A | ● |  | ● | ● | ● |  |
| 426 | STX8.81 | [chr17:9276598-9276967](http://genome.ucsc.edu/cgi-bin/hgTracks?db=hg17&position=chr17:9276598-9276967&hgt.customText=http://www.neurogenome.org/mcs/tracks/chr17.txt) | 370 | 2b | N/A |  |  | ● | ● | ● |  |
| 427 | STX8.140 | [chr17:9174687-9175169](http://genome.ucsc.edu/cgi-bin/hgTracks?db=hg17&position=chr17:9174687-9175169&hgt.customText=http://www.neurogenome.org/mcs/tracks/chr17.txt) | 483 | 2b | N/A |  |  | ● | ● | ● |  |
| 428 | STX8.142 | [chr17:9174070-9174429](http://genome.ucsc.edu/cgi-bin/hgTracks?db=hg17&position=chr17:9174070-9174429&hgt.customText=http://www.neurogenome.org/mcs/tracks/chr17.txt) | 360 | 2b | N/A |  |  | ● | ● |  |  |
| 429 | STX8.148 | [chr17:9166282-9166881](http://genome.ucsc.edu/cgi-bin/hgTracks?db=hg17&position=chr17:9166282-9166881&hgt.customText=http://www.neurogenome.org/mcs/tracks/chr17.txt) | 600 | 2b | N/A | ● | ● | ● | ● |  |  |
| 430 | STX8.195 | [chr17:9091527-9091939](http://genome.ucsc.edu/cgi-bin/hgTracks?db=hg17&position=chr17:9091527-9091939&hgt.customText=http://www.neurogenome.org/mcs/tracks/chr17.txt) | 413 | 2a (3’) | N/A |  |  | ● | ● |  |  |
| 431 | STXBP1.67 | [chr9:127531721-127532102](http://genome.ucsc.edu/cgi-bin/hgTracks?db=hg17&position=chr9:127531721-127532102&hgt.customText=http://www.neurogenome.org/mcs/tracks/chr9.txt) | 382 | 2b | N/A |  |  | ● | ● |  |  |
| 432 | STXBP4.99 | [chr17:50573040-50573900](http://genome.ucsc.edu/cgi-bin/hgTracks?db=hg17&position=chr17:50573040-50573900&hgt.customText=http://www.neurogenome.org/mcs/tracks/chr17.txt) | 861 | 1b | N/A |  | ● | ● | ● |  |  |
| 433 | STXBP5.247 | [chr6:147726162-147726539](http://genome.ucsc.edu/cgi-bin/hgTracks?db=hg17&position=chr6:147726162-147726539&hgt.customText=http://www.neurogenome.org/mcs/tracks/chr6.txt) | 378 | 1a | ● | ● |  | ● | ● | ● | ● |
| 434 | STXBP6.10 | [chr14:24786221-24786613](http://genome.ucsc.edu/cgi-bin/hgTracks?db=hg17&position=chr14:24786221-24786613&hgt.customText=http://www.neurogenome.org/mcs/tracks/chr14.txt) | 393 | 2a (5’) |  |  |  | ● | ● |  |  |
| 435 | STXBP6.19 | [chr14:24761427-24761801](http://genome.ucsc.edu/cgi-bin/hgTracks?db=hg17&position=chr14:24761427-24761801&hgt.customText=http://www.neurogenome.org/mcs/tracks/chr14.txt) | 375 | 2a (5’) |  |  | ● | ● | ● |  |  |
| 436 | STXBP6.86 | [chr14:24588897-24589282](http://genome.ucsc.edu/cgi-bin/hgTracks?db=hg17&position=chr14:24588897-24589282&hgt.customText=http://www.neurogenome.org/mcs/tracks/chr14.txt) | 386 | 1c (5’) | ● | ● |  |  | ● |  |  |
| 437 | STXBP6.301 | [chr14:24251094-24251464](http://genome.ucsc.edu/cgi-bin/hgTracks?db=hg17&position=chr14:24251094-24251464&hgt.customText=http://www.neurogenome.org/mcs/tracks/chr14.txt) | 371 | 2a (3’) |  |  |  | ● | ● |  |  |
| 438 | SV2A.16 | [chr1:146697838-146698279](http://genome.ucsc.edu/cgi-bin/hgTracks?db=hg17&position=chr1:146697838-146698279&hgt.customText=http://www.neurogenome.org/mcs/tracks/chr1.txt) | 442 | 1a | N/A | ● |  | ● | ● | ● | ● |
| 439 | SV2B.47 | [chr15:89463192-89463600](http://genome.ucsc.edu/cgi-bin/hgTracks?db=hg17&position=chr15:89463192-89463600&hgt.customText=http://www.neurogenome.org/mcs/tracks/chr15.txt) | 409 | 2b | N/A |  |  | ● | ● |  |  |
| 440 | SV2C.10 | [chr5:75084452-75085082](http://genome.ucsc.edu/cgi-bin/hgTracks?db=hg17&position=chr5:75084452-75085082&hgt.customText=http://www.neurogenome.org/mcs/tracks/chr5.txt) | 631 | 2a (5’) | N/A |  | ● | ● | ● | ● | ● |
| 441 | SV2C.122 | [chr5:75410867-75411352](http://genome.ucsc.edu/cgi-bin/hgTracks?db=hg17&position=chr5:75410867-75411352&hgt.customText=http://www.neurogenome.org/mcs/tracks/chr5.txt) | 486 | 2a (5’) | N/A |  |  | ● | ● | ● | ● |
| 442 | SV2C.199 | [chr5:75573428-75573882](http://genome.ucsc.edu/cgi-bin/hgTracks?db=hg17&position=chr5:75573428-75573882&hgt.customText=http://www.neurogenome.org/mcs/tracks/chr5.txt) | 455 | 2b | N/A | ● | ● | ● | ● | ● |  |
| 443 | SYN1.11 | [chrX:47235428-47235839](http://genome.ucsc.edu/cgi-bin/hgTracks?db=hg17&position=chrX:47235428-47235839&hgt.customText=http://www.neurogenome.org/mcs/tracks/chrX.txt) | 412 | 1c (5’) | ● |  |  |  | ● |  |  |
| 444 | SYN1.73 | [chrX:47188299-47188790](http://genome.ucsc.edu/cgi-bin/hgTracks?db=hg17&position=chrX:47188299-47188790&hgt.customText=http://www.neurogenome.org/mcs/tracks/chrX.txt) | 492 | 1c (3’) | ● | ● | ● | ● | ● |  |  |
| 445 | SYN2.152 | [chr3:12086843-12087536](http://genome.ucsc.edu/cgi-bin/hgTracks?db=hg17&position=chr3:12086843-12087536&hgt.customText=http://www.neurogenome.org/mcs/tracks/chr3.txt) | 694 | 2b | N/A |  |  | ● | ● | ● | ● |
| 446 | SYN2.189 | [chr3:12136779-12137207](http://genome.ucsc.edu/cgi-bin/hgTracks?db=hg17&position=chr3:12136779-12137207&hgt.customText=http://www.neurogenome.org/mcs/tracks/chr3.txt) | 429 | 2b | N/A |  | ● | ● | ● |  |  |
| 447 | SYN2.235 | [chr3:12165417-12165801](http://genome.ucsc.edu/cgi-bin/hgTracks?db=hg17&position=chr3:12165417-12165801&hgt.customText=http://www.neurogenome.org/mcs/tracks/chr3.txt) | 385 | 1a | N/A | ● | ● | ● | ● |  |  |
| 448 | SYN2.276 | [chr3:12194529-12194920](http://genome.ucsc.edu/cgi-bin/hgTracks?db=hg17&position=chr3:12194529-12194920&hgt.customText=http://www.neurogenome.org/mcs/tracks/chr3.txt) | 392 | 2b | N/A |  | ● | ● | ● |  |  |
| 449 | SYN2.295 | [chr3:12201746-12202294](http://genome.ucsc.edu/cgi-bin/hgTracks?db=hg17&position=chr3:12201746-12202294&hgt.customText=http://www.neurogenome.org/mcs/tracks/chr3.txt) | 549 | 1c (3’) | N/A | ● | ● | ● | ● | ● |  |
| 450 | SYN3.67 | [chr22:31688006-31688452](http://genome.ucsc.edu/cgi-bin/hgTracks?db=hg17&position=chr22:31688006-31688452&hgt.customText=http://www.neurogenome.org/mcs/tracks/chr22.txt) | 447 | 2b |  |  | ● | ● | ● |  |  |
| 451 | SYN3.69 | [chr22:31677748-31678316](http://genome.ucsc.edu/cgi-bin/hgTracks?db=hg17&position=chr22:31677748-31678316&hgt.customText=http://www.neurogenome.org/mcs/tracks/chr22.txt) | 569 | 2b |  |  |  | ● | ● |  |  |
| 452 | SYN3.310 | [chr22:31338832-31339293](http://genome.ucsc.edu/cgi-bin/hgTracks?db=hg17&position=chr22:31338832-31339293&hgt.customText=http://www.neurogenome.org/mcs/tracks/chr22.txt) | 462 | 2b |  |  |  | ● | ● |  |  |
| 453 | SYT1.48 | [chr12:77224038-77224398](http://genome.ucsc.edu/cgi-bin/hgTracks?db=hg17&position=chr12:77224038-77224398&hgt.customText=http://www.neurogenome.org/mcs/tracks/chr12.txt) | 361 | 2a (5’) | N/A |  |  | ● | ● |  |  |
| 454 | SYT1.270 | [chr12:77651243-77651636](http://genome.ucsc.edu/cgi-bin/hgTracks?db=hg17&position=chr12:77651243-77651636&hgt.customText=http://www.neurogenome.org/mcs/tracks/chr12.txt) | 394 | 2a (5’) | N/A |  |  | ● | ● |  |  |
| 455 | SYT1.286 | [chr12:77689512-77690249](http://genome.ucsc.edu/cgi-bin/hgTracks?db=hg17&position=chr12:77689512-77690249&hgt.customText=http://www.neurogenome.org/mcs/tracks/chr12.txt) | 738 | 2a (5’) | N/A |  |  | ● | ● | ● | ● |
| 456 | SYT1.299 | [chr12:77698959-77699432](http://genome.ucsc.edu/cgi-bin/hgTracks?db=hg17&position=chr12:77698959-77699432&hgt.customText=http://www.neurogenome.org/mcs/tracks/chr12.txt) | 474 | 2a (5’) | N/A |  |  | ● | ● |  |  |
| 457 | SYT1.512 | [chr12:78044147-78044587](http://genome.ucsc.edu/cgi-bin/hgTracks?db=hg17&position=chr12:78044147-78044587&hgt.customText=http://www.neurogenome.org/mcs/tracks/chr12.txt) | 441 | 2b | N/A |  |  | ● | ● | ● |  |
| 458 | SYT1.627 | [chr12:78199728-78200132](http://genome.ucsc.edu/cgi-bin/hgTracks?db=hg17&position=chr12:78199728-78200132&hgt.customText=http://www.neurogenome.org/mcs/tracks/chr12.txt) | 405 | 2b | N/A |  | ● | ● | ● |  |  |
| 459 | SYT1.635 | [chr12:78205651-78206018](http://genome.ucsc.edu/cgi-bin/hgTracks?db=hg17&position=chr12:78205651-78206018&hgt.customText=http://www.neurogenome.org/mcs/tracks/chr12.txt) | 368 | 2b | N/A |  |  | ● | ● | ● |  |
| 460 | SYT1.778 | [chr12:78345382-78345835](http://genome.ucsc.edu/cgi-bin/hgTracks?db=hg17&position=chr12:78345382-78345835&hgt.customText=http://www.neurogenome.org/mcs/tracks/chr12.txt) | 454 | 1c (3’) | N/A | ● |  | ● | ● | ● | ● |
| 461 | SYT1.781 | [chr12:78346164-78347011](http://genome.ucsc.edu/cgi-bin/hgTracks?db=hg17&position=chr12:78346164-78347011&hgt.customText=http://www.neurogenome.org/mcs/tracks/chr12.txt) | 848 | 1c (3’) | N/A | ● |  | ● | ● | ● |  |
| 462 | SYT1.785 | [chr12:78347874-78348264](http://genome.ucsc.edu/cgi-bin/hgTracks?db=hg17&position=chr12:78347874-78348264&hgt.customText=http://www.neurogenome.org/mcs/tracks/chr12.txt) | 391 | 1c (3’) | N/A | ● |  | ● | ● | ● | ● |
| 463 | SYT10.78 | [chr12:33450984-33451476](http://genome.ucsc.edu/cgi-bin/hgTracks?db=hg17&position=chr12:33450984-33451476&hgt.customText=http://www.neurogenome.org/mcs/tracks/chr12.txt) | 493 | 1a | N/A | ● |  | ● | ● | ● | ● |
| 464 | SYT11.16 | [chr1:152651275-152651660](http://genome.ucsc.edu/cgi-bin/hgTracks?db=hg17&position=chr1:152651275-152651660&hgt.customText=http://www.neurogenome.org/mcs/tracks/chr1.txt) | 386 | 1a | N/A | ● |  | ● | ● | ● | ● |
| 465 | SYT13.4 | [chr11:45344804-45345185](http://genome.ucsc.edu/cgi-bin/hgTracks?db=hg17&position=chr11:45344804-45345185&hgt.customText=http://www.neurogenome.org/mcs/tracks/chr11.txt) | 382 | 2a (5’) | N/A |  |  | ● | ● |  |  |
| 466 | SYT13.7 | [chr11:45341924-45342289](http://genome.ucsc.edu/cgi-bin/hgTracks?db=hg17&position=chr11:45341924-45342289&hgt.customText=http://www.neurogenome.org/mcs/tracks/chr11.txt) | 366 | 2a (5’) | N/A |  |  | ● | ● |  |  |
| 467 | SYT13.26 | [chr11:45264147-45264557](http://genome.ucsc.edu/cgi-bin/hgTracks?db=hg17&position=chr11:45264147-45264557&hgt.customText=http://www.neurogenome.org/mcs/tracks/chr11.txt) | 411 | 1c (5’) | N/A | ● |  | ● | ● |  |  |
| 468 | SYT13.83 | [chr11:45205845-45206237](http://genome.ucsc.edu/cgi-bin/hgTracks?db=hg17&position=chr11:45205845-45206237&hgt.customText=http://www.neurogenome.org/mcs/tracks/chr11.txt) | 393 | 2a (3’) | N/A | ● |  | ● | ● | ● | ● |
| 469 | SYT13.85 | [chr11:45204899-45205775](http://genome.ucsc.edu/cgi-bin/hgTracks?db=hg17&position=chr11:45204899-45205775&hgt.customText=http://www.neurogenome.org/mcs/tracks/chr11.txt) | 877 | 2a (3’) | N/A | ● | ● | ● | ● | ● | ● |
| 470 | SYT13.87 | [chr11:45204253-45204842](http://genome.ucsc.edu/cgi-bin/hgTracks?db=hg17&position=chr11:45204253-45204842&hgt.customText=http://www.neurogenome.org/mcs/tracks/chr11.txt) | 590 | 2a (3’) | N/A | ● | ● | ● | ● | ● | ● |
| 471 | SYT14.12 | [chr1:206441472-206441838](http://genome.ucsc.edu/cgi-bin/hgTracks?db=hg17&position=chr1:206441472-206441838&hgt.customText=http://www.neurogenome.org/mcs/tracks/chr1.txt) | 367 | 2a (5’) | N/A |  | ● | ● | ● |  |  |
| 472 | SYT14.112 | [chr1:206721025-206721397](http://genome.ucsc.edu/cgi-bin/hgTracks?db=hg17&position=chr1:206721025-206721397&hgt.customText=http://www.neurogenome.org/mcs/tracks/chr1.txt) | 373 | 2b | N/A |  |  | ● | ● | ● | ● |
| 473 | SYT15.27 | [chr10:46381280-46381843](http://genome.ucsc.edu/cgi-bin/hgTracks?db=hg17&position=chr10:46381280-46381843&hgt.customText=http://www.neurogenome.org/mcs/tracks/chr10.txt) | 564 | 1c (3’) | N/A | ● | ● | ● | ● |  | ● |
| 474 | SYT16.62 | [chr14:61448083-61448459](http://genome.ucsc.edu/cgi-bin/hgTracks?db=hg17&position=chr14:61448083-61448459&hgt.customText=http://www.neurogenome.org/mcs/tracks/chr14.txt) | 377 | 2a (5’) |  |  |  | ● | ● |  |  |
| 475 | SYT16.136 | [chr14:61542875-61543266](http://genome.ucsc.edu/cgi-bin/hgTracks?db=hg17&position=chr14:61542875-61543266&hgt.customText=http://www.neurogenome.org/mcs/tracks/chr14.txt) | 392 | 2b |  |  |  | ● | ● | ● |  |
| 476 | SYT16.164 | [chr14:61609515-61609925](http://genome.ucsc.edu/cgi-bin/hgTracks?db=hg17&position=chr14:61609515-61609925&hgt.customText=http://www.neurogenome.org/mcs/tracks/chr14.txt) | 411 | 2b |  |  |  | ● | ● | ● |  |
| 477 | SYT16.215 | [chr14:61647465-61647831](http://genome.ucsc.edu/cgi-bin/hgTracks?db=hg17&position=chr14:61647465-61647831&hgt.customText=http://www.neurogenome.org/mcs/tracks/chr14.txt) | 367 | 2a (3’) | ● | ● |  | ● | ● |  |  |
| 478 | SYT17.42 | [chr16:19091064-19091444](http://genome.ucsc.edu/cgi-bin/hgTracks?db=hg17&position=chr16:19091064-19091444&hgt.customText=http://www.neurogenome.org/mcs/tracks/chr16.txt) | 381 | 2b | N/A | ● |  | ● | ● |  |  |
| 479 | SYT3.15 | [chr19:55856806-55857651](http://genome.ucsc.edu/cgi-bin/hgTracks?db=hg17&position=chr19:55856806-55857651&hgt.customText=http://www.neurogenome.org/mcs/tracks/chr19.txt) | 846 | 1c (3’) |  | ● |  |  | ● |  | ● |
| 480 | SYT3.75 | [chr19:55762864-55763235](http://genome.ucsc.edu/cgi-bin/hgTracks?db=hg17&position=chr19:55762864-55763235&hgt.customText=http://www.neurogenome.org/mcs/tracks/chr19.txt) | 372 | 2a (3’) | ● | ● |  | ● | ● |  |  |
| 481 | SYT4.4 | [chr18:39301545-39301907](http://genome.ucsc.edu/cgi-bin/hgTracks?db=hg17&position=chr18:39301545-39301907&hgt.customText=http://www.neurogenome.org/mcs/tracks/chr18.txt) | 363 | 2a (5’) | N/A |  |  | ● | ● |  |  |
| 482 | SYT4.15 | [chr18:39226161-39226549](http://genome.ucsc.edu/cgi-bin/hgTracks?db=hg17&position=chr18:39226161-39226549&hgt.customText=http://www.neurogenome.org/mcs/tracks/chr18.txt) | 389 | 2a (5’) | N/A |  |  | ● | ● |  |  |
| 483 | SYT6.40 | [chr1:114545517-114545979](http://genome.ucsc.edu/cgi-bin/hgTracks?db=hg17&position=chr1:114545517-114545979&hgt.customText=http://www.neurogenome.org/mcs/tracks/chr1.txt) | 463 | 2a (5’) | N/A |  |  | ● | ● |  |  |
| 484 | SYT6.151 | [chr1:114410250-114410710](http://genome.ucsc.edu/cgi-bin/hgTracks?db=hg17&position=chr1:114410250-114410710&hgt.customText=http://www.neurogenome.org/mcs/tracks/chr1.txt) | 461 | 2a (5’) | N/A | ● | ● | ● | ● |  |  |
| 485 | SYT7.53 | [chr11:61104686-61105336](http://genome.ucsc.edu/cgi-bin/hgTracks?db=hg17&position=chr11:61104686-61105336&hgt.customText=http://www.neurogenome.org/mcs/tracks/chr11.txt) | 651 | 1b | N/A |  |  |  | ● |  |  |
| 486 | SYT7.95 | [chr11:61090594-61090971](http://genome.ucsc.edu/cgi-bin/hgTracks?db=hg17&position=chr11:61090594-61090971&hgt.customText=http://www.neurogenome.org/mcs/tracks/chr11.txt) | 378 | 2b | N/A |  |  | ● | ● |  |  |
| 487 | SYT7.136 | [chr11:61070415-61071085](http://genome.ucsc.edu/cgi-bin/hgTracks?db=hg17&position=chr11:61070415-61071085&hgt.customText=http://www.neurogenome.org/mcs/tracks/chr11.txt) | 671 | 2b | N/A |  |  | ● | ● |  |  |
| 488 | SYT7.147 | [chr11:61066214-61066698](http://genome.ucsc.edu/cgi-bin/hgTracks?db=hg17&position=chr11:61066214-61066698&hgt.customText=http://www.neurogenome.org/mcs/tracks/chr11.txt) | 485 | 2b | N/A |  |  | ● | ● | ● | ● |
| 489 | SYT7.204 | [chr11:61034661-61035112](http://genome.ucsc.edu/cgi-bin/hgTracks?db=hg17&position=chr11:61034661-61035112&hgt.customText=http://www.neurogenome.org/mcs/tracks/chr11.txt) | 452 | 2a (3’) | N/A | ● | ● | ● | ● |  |  |
| 490 | SYT7.208 | [chr11:61032900-61033648](http://genome.ucsc.edu/cgi-bin/hgTracks?db=hg17&position=chr11:61032900-61033648&hgt.customText=http://www.neurogenome.org/mcs/tracks/chr11.txt) | 749 | 2a (3’) | N/A | ● |  |  | ● | ● | ● |
| 491 | SYT9.61 | [chr11:7200283-7200691](http://genome.ucsc.edu/cgi-bin/hgTracks?db=hg17&position=chr11:7200283-7200691&hgt.customText=http://www.neurogenome.org/mcs/tracks/chr11.txt) | 409 | 2a (5’) | N/A |  | ● | ● | ● |  |  |
| 492 | SYT9.152 | [chr11:7291348-7291757](http://genome.ucsc.edu/cgi-bin/hgTracks?db=hg17&position=chr11:7291348-7291757&hgt.customText=http://www.neurogenome.org/mcs/tracks/chr11.txt) | 410 | 1c (5’) | N/A | ● |  | ● | ● | ● | ● |
| 493 | SYT9.193 | [chr11:7396332-7396824](http://genome.ucsc.edu/cgi-bin/hgTracks?db=hg17&position=chr11:7396332-7396824&hgt.customText=http://www.neurogenome.org/mcs/tracks/chr11.txt) | 493 | 2b | N/A |  |  | ● | ● |  |  |
| 494 | SYTL2.110 | [chr11:85107582-85108198](http://genome.ucsc.edu/cgi-bin/hgTracks?db=hg17&position=chr11:85107582-85108198&hgt.customText=http://www.neurogenome.org/mcs/tracks/chr11.txt) | 617 | 1c (5’) | N/A | ● | ● | ● | ● |  |  |
| 495 | SYTL4.29 | [chrX:99807091-99807679](http://genome.ucsc.edu/cgi-bin/hgTracks?db=hg17&position=chrX:99807091-99807679&hgt.customText=http://www.neurogenome.org/mcs/tracks/chrX.txt) | 589 | 2a (5’) |  |  |  | ● | ● |  |  |
| 496 | UNC13A.87 | [chr19:17561402-17561902](http://genome.ucsc.edu/cgi-bin/hgTracks?db=hg17&position=chr19:17561402-17561902&hgt.customText=http://www.neurogenome.org/mcs/tracks/chr19.txt) | 501 | 2a (3’) | ● |  | ● | ● | ● | ● | ● |
| 497 | UNC13B.104 | [chr9:35296758-35297137](http://genome.ucsc.edu/cgi-bin/hgTracks?db=hg17&position=chr9:35296758-35297137&hgt.customText=http://www.neurogenome.org/mcs/tracks/chr9.txt) | 380 | 2b | N/A |  |  | ● | ● |  |  |
| 498 | UNC13B.148 | [chr9:35330001-35330430](http://genome.ucsc.edu/cgi-bin/hgTracks?db=hg17&position=chr9:35330001-35330430&hgt.customText=http://www.neurogenome.org/mcs/tracks/chr9.txt) | 430 | 2b | N/A |  |  | ● | ● |  |  |
| 499 | UNC13C.53 | [chr15:52092824-52093382](http://genome.ucsc.edu/cgi-bin/hgTracks?db=hg17&position=chr15:52092824-52093382&hgt.customText=http://www.neurogenome.org/mcs/tracks/chr15.txt) | 559 | 2a (5’) | N/A | ● | ● | ● | ● | ● | ● |
| 500 | UNC13C.59 | [chr15:52094505-52094989](http://genome.ucsc.edu/cgi-bin/hgTracks?db=hg17&position=chr15:52094505-52094989&hgt.customText=http://www.neurogenome.org/mcs/tracks/chr15.txt) | 485 | 2a (5’) | N/A | ● | ● | ● | ● | ● | ● |
| 501 | UNC13C.82 | [chr15:52128690-52129287](http://genome.ucsc.edu/cgi-bin/hgTracks?db=hg17&position=chr15:52128690-52129287&hgt.customText=http://www.neurogenome.org/mcs/tracks/chr15.txt) | 598 | 2a (5’) | N/A |  |  | ● | ● | ● | ● |
| 502 | VAMP2.22 | [chr17:8003117-8003508](http://genome.ucsc.edu/cgi-bin/hgTracks?db=hg17&position=chr17:8003117-8003508&hgt.customText=http://www.neurogenome.org/mcs/tracks/chr17.txt) | 392 | 1c (3’) | N/A | ● | ● | ● | ● |  |  |
| 503 | VAMP4.40 | [chr1:168384233-168385264](http://genome.ucsc.edu/cgi-bin/hgTracks?db=hg17&position=chr1:168384233-168385264&hgt.customText=http://www.neurogenome.org/mcs/tracks/chr1.txt) | 1032 | 2a (3’) | N/A |  |  | ● | ● | ● | ● |
| 504 | VAMP4.51 | [chr1:168371134-168371737](http://genome.ucsc.edu/cgi-bin/hgTracks?db=hg17&position=chr1:168371134-168371737&hgt.customText=http://www.neurogenome.org/mcs/tracks/chr1.txt) | 604 | 2a (3’) | N/A | ● |  | ● | ● |  |  |
